# Aminocatalytic enantioselective [2 + 2] cycloaddition of Bicyclo[1.1.0]butanes and α,β-unsaturated aldehydes

**DOI:** 10.1039/d5sc05477j

**Published:** 2025-08-12

**Authors:** René Slot Bitsch, Enrico Marcantonio, Erlaitz Basabe Obregón, Ida Rygaard Kocemba, Jonas Faghtmann, Karl Anker Jørgensen

**Affiliations:** a Department of Chemistry, Aarhus University 8000 Aarhus C Denmark kaj@chem.au.dk

## Abstract

Bicyclo[1.1.0]butanes have opened a new area of chemical space for construction of bicyclo[2.1.1]hexanes – a scaffold showing promise as *ortho*- and *meta*-aryl bioisosteres. Herein, we present the first aminocatalytic concept that enables the enantioselective [2 + 2] cycloaddition of bicyclo[1.1.0]butanes with α,β-unsaturated aldehydes. The reaction is general for α,β-unsaturated aldehydes, substituted at the γ-position with aromatic functionalities, esters and ketones, by applying a secondary aminocatalyst and Yb(OTf)_3_ as a Lewis acid to activate the bicyclo[1.1.0]butane. For cinnamaldehydes, bicyclo[2.1.1]hexane cycloadducts are obtained in moderate to good yields, and up to 96.5 : 3.5 e.r. Pleasingly, α,β-unsaturated aldehydes containing ester and ketone functionalities in the γ-position provided high yields and enantioselectivities up to 98.5 : 1.5 e.r. For all three classes of [2 + 2] cycloadditions, a range of α,β-unsaturated aldehydes and bicyclo[1.1.0]butanes were successfully tolerated. Several transformations adding further complexity to the bicyclo[2.1.1]hexane scaffolds are disclosed. Finally, a reaction mechanism is proposed.

## Abstract 2

An aminocatalytic asymmetric [2 + 2] cycloaddition for the formation of bicyclo[2.1.1]hexanes is presented. The bicyclo[2.1.1]hexane scaffold is a potential aryl bioisostere and its complexity can be increased by several synthetic elaborations.

## Graphical abstract

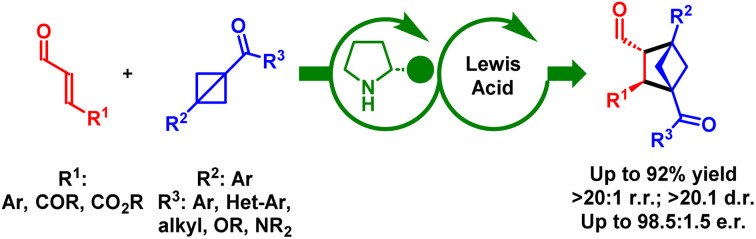

## Introduction

**Scheme 1:**
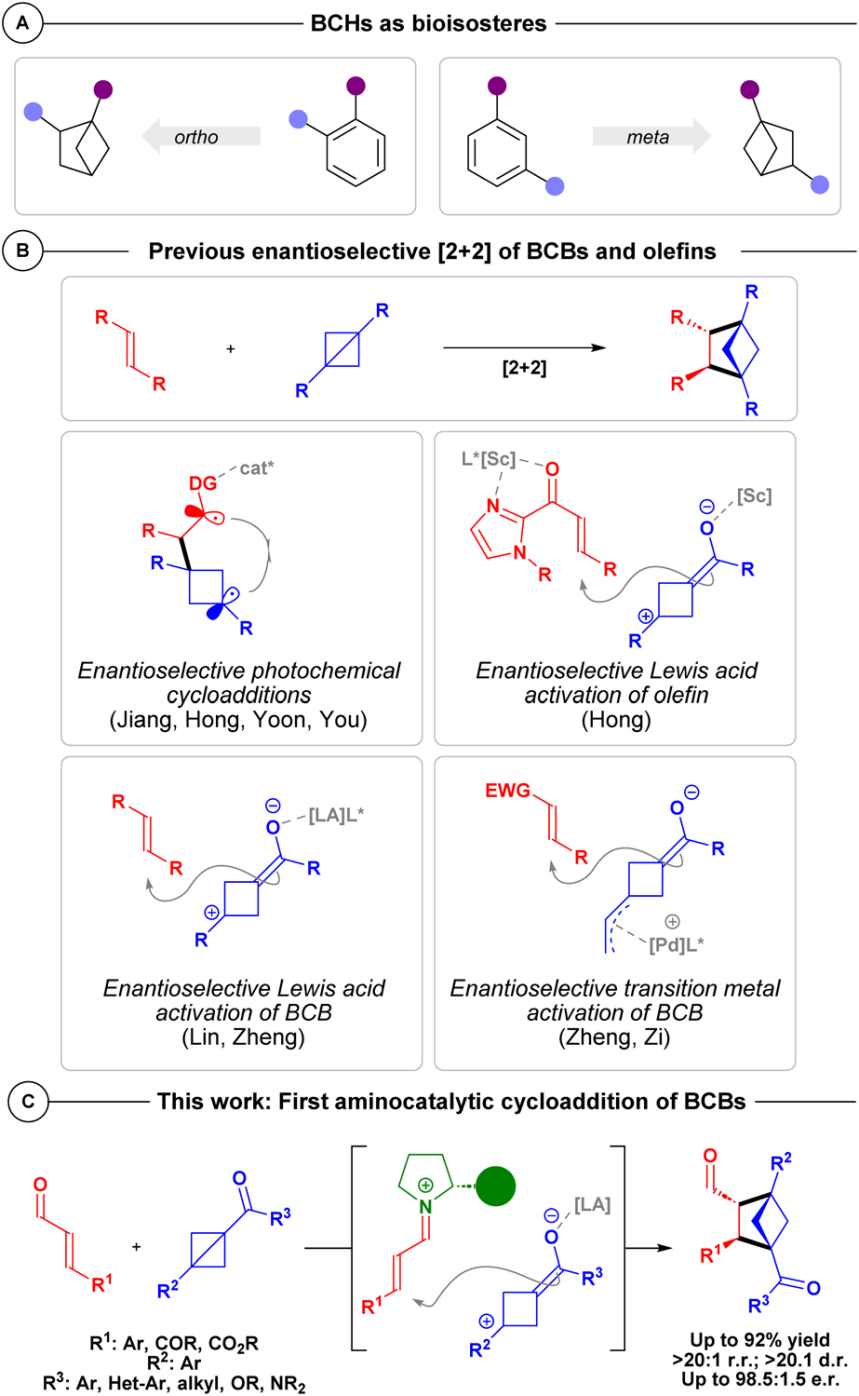
(A) BCHs as bioisosteres. (B) Asymmetric activation strategies to form BCHs. (C) Envisioned enantioselective aminocatalytic activation strategy.

Recently, small carbocyclic rings have received considerable attention due to their beneficial physicochemical properties and prospective use as bioisosteres.^1^ In particular, bicyclo[2.1.1]hexanes (BCHs) have been in focus due to their potential as *ortho*- or *meta*-aryl replacements within the “Escape from Flatland” paradigm (Scheme 1A).^2^

The most facile approach for the generation of decorated BCHs is the strain-release driven [2 + 2] cycloaddition between the central bond of bicyclo[1.1.0]butanes (BCBs) and olefins. This was first accomplished in a racemic fashion by leveraging a photocatalytic energy-transfer process by the group of Glorius,^3^ or by utilizing a SmI_2_ catalyzed radical relay process as demonstrated by Procter *et al.*^4^

The first entry into enantioenriched BCHs from BCBs was developed by the group of Bach using a stoichiometric chiral template as photosensitizer to promote an energy-transfer event to enable the [2 + 2] cycloaddition.^5^ Since then, several catalytic enantioselective methodologies have been developed to access carbon-based BCHs, mainly relying on four different catalytic strategies.^6^

The first is based on photocatalytic [2 + 2] cycloadditions between BCBs and an olefin bearing a directing-group in the presence of a chiral Brønsted acid or Lewis acid (Scheme 1B, top left).^7^ Similarly, chiral Lewis acid catalysis has played a pivotal role in the formation of BCHs from BCBs in asymmetric [2 + 2] cycloadditions, and have been accomplished either by activating the olefin (Scheme 1B, top right)^7*b*^ or the BCB (Scheme 1B, bottom left).^8^ Lastly enantioenriched BCHs have also been accessed from transition-metal catalysis of vinyl-BCBs with electron-deficient olefins (Scheme 1B, bottom right).^9^ While not yet having been utilized for the formation of all-carbon BCH scaffolds, chiral Brønsted-acid catalysts have shown success for the formation of enantioenriched *aza*-BCHs.^10^ To the best of our knowledge, aminocatalysis has not been explored in the context of BCB chemistry for the enantioselective synthesis of BCHs.

Aminocatalysis has proven compatible with multiple external catalytic systems in synergistic strategies,^11^ including metal complexes such as palladium,^12^ and iridium,^13^ but also Lewis acids,^14^ and photoredox systems.^15^ In light of previous works regarding the formation of BCHs from BCBs, and the high tolerance of aminocatalysis towards external catalytic systems, we anticipated that a synergistic aminocatalytic methodology might be possible. Therefore, the BCB requires activation by a Lewis acid, meanwhile the aminocatalyst should activate the α,β-unsaturated aldehyde by *in situ* formation of an iminium ion (Scheme 1C).^11,16^

## Results and discussion

The envisioned [2 + 2] cycloaddition was unlocked by reacting 4-bromocinnamaldehyde 1a and disubstituted phenyl ketone BCB 2a in the presence of trimethylsilyl diaryl prolinol C1 and HFIP (Table 1, entry 1). We were pleased to observe that the envisioned BCH 3a was formed in 30% yield as a single regio- and diastereoisomer in 13 : 87 e.r. Next, we replaced HFIP with a catalytic amount of Yb(OTf)_3_ and we were pleased that the enantiomeric ratio of 3a was improved to 3 : 97 e.r. (entry 2). Two control experiments demonstrated the necessity of both aminocatalyst and Lewis acid for reactivity (entry 3 and 4). A series of Lewis acids were investigated (Table 1, entry 5, 6 and SI). The presence of a catalytic amount of Sc(OTf)_3_ or BF_3_·OEt_2_ both provided the same enantiomeric ratio of 3a in 3 : 97 e.r. similar to the application of Yb(OTf)_3_. Interestingly, in the presence of BF_3_·OEt_2_, a by-product speculated to originate from the *oxa*-[2 + 2] between the BCB and aldehyde carbonyl, as demonstrated by Glorius utilizing the same Lewis acid, was observed in 25% NMR yield.^17^ Applying THF or PhCl as the solvent, the enantiomeric ratio improved up to 0.5 : 99.5; however, at the expense of the yield of 3a (entry 7 and 8). During the optimization, we observed that water was crucial for reactivity, as its absence impaired the reaction (entry 9 and 10). A beneficial effect of water has also been observed by *e.g.* Feringa and Kobayashi who proposed that water coordinates to Yb(iii), thereby loosening the coordination sphere and making it more accessible for coordination.^18^

**Table 1:** Optimization of reaction conditions^a^

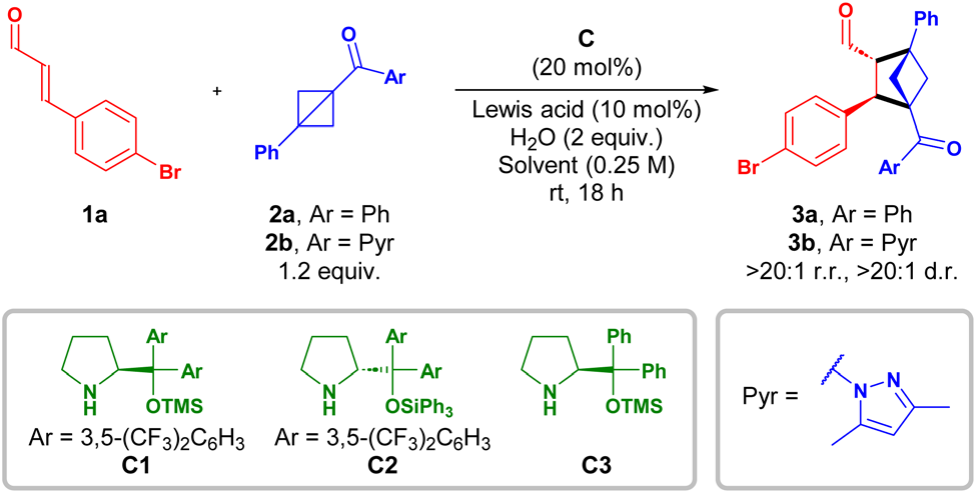						
Entry	Solv.	BCB	LA	Cat.	Yield^b^ [%]	e.r.
1^c^	CH_2_Cl_2_	2a	—	C1	30	13 : 87
2	CH_2_Cl_2_	2a	Yb(OTf)_3_	C1	31	3 : 97
3	CH_2_Cl_2_	2a	—	C1	<5	—
4	CH_2_Cl_2_	2a	Yb(OTf)_3_	—	<5	—
5	CH_2_Cl_2_	2a	Sc(OTf)_3_	C1	29	3 : 97
6	CH_2_Cl_2_	2a	BF_3_·OEt_2_	C1	30	4 : 96
7	THF	2a	Yb(OTf)_3_	C1	13	1.5 : 98.5
8	PhCl	2a	Yb(OTf)_3_	C1	23	0.5 : 99.5
9^d^	CH_2_Cl_2_	2a	Yb(OTf)_3_	C1	16	3 : 97
10^e^	CH_2_Cl_2_	2a	Yb(OTf)	C1	<5	—
11^f^	CH_2_Cl_2_	2a	Yb(OTf)_3_	C1	35	3 : 97
12^f^^,^^g^	CH_2_Cl_2_	2a	Yb(OTf)_3_	C1	63^h^	3.5 : 96.5
13^f^	CH_2_Cl_2_	2b	Yb(OTf)_3_	C1	56	10 : 90
14^f^	CH_2_Cl_2_	2b	Yb(OTf)_3_	C2	62^h^	93 : 7
15^f^	CH_2_Cl_2_	2b	Yb(OTf)_3_	C3	<5	—

a Reaction conditions: 1a (0.050 mmol), 2a or 2b (0.060 mmol), C (20 mol%), Lewis acid (10 mol%), H_2_O (0.10 mmol) in solvent (0.2 mL) for 18 h at rt. Regio- and diastereoisomeric ratios determined by ^1^H NMR spectroscopy of the reaction crude. e.r. determined by chiral-phase ultra performance convergence chromatography (UPC^2^) analysis.

b Determined by ^1^H NMR spectroscopy of the reaction crude using 1,3,5-trimethoxybenzene as internal standard.

c Using 10 equiv. of HFIP, and no addition of H_2_O.

d Using 10 equiv. of H_2_O.

e Using 2 spheres of 4 Å MS and no addition of H_2_O.

f Using 3 equiv. of 1a (0.15 mmol) and 1 equiv. of 2a or 2b (0.050 mmol).

g Using 40 mol% C1 and stirred for 48 h.

h Isolated yield.

The reaction was dependent on the stoichiometry, with a modest increase in yield applying 3 equiv. of 1a relative to 2a (entry 11). To achieve full conversion of BCB 2a increased aminocatalyst loading and reaction time were required, affording 3a in good yield and excellent enantioselectivity (entry 12 and SI). Replacing the phenyl ketone in the BCB to a pyrazole containing BCB 2b improved the conversion and cycloadduct 3b was furnished in 56% yield and 10 : 90 e.r. (entry 13 and SI). Screening of aminocatalysts revealed enhanced enantiomeric ratio with catalyst C2 (entry 14, 15 and SI). The reaction was incompatible with monosubstituted BCBs, presumably due to lack of the electronic push–pull system present in disubstituted BCBs 2 (see SI).^1*b*,19^

With the optimal conditions in hand (Table 1, entry 10 and 12), the scope of the [2 + 2] cycloaddition of substituted cinnamaldehydes 1 with different BCBs 2 was investigated (Table 2).

**Table 2:** Scope of the [2 + 2] cycloaddition between cinnamaldehydes 1 and BCBs 2^a^

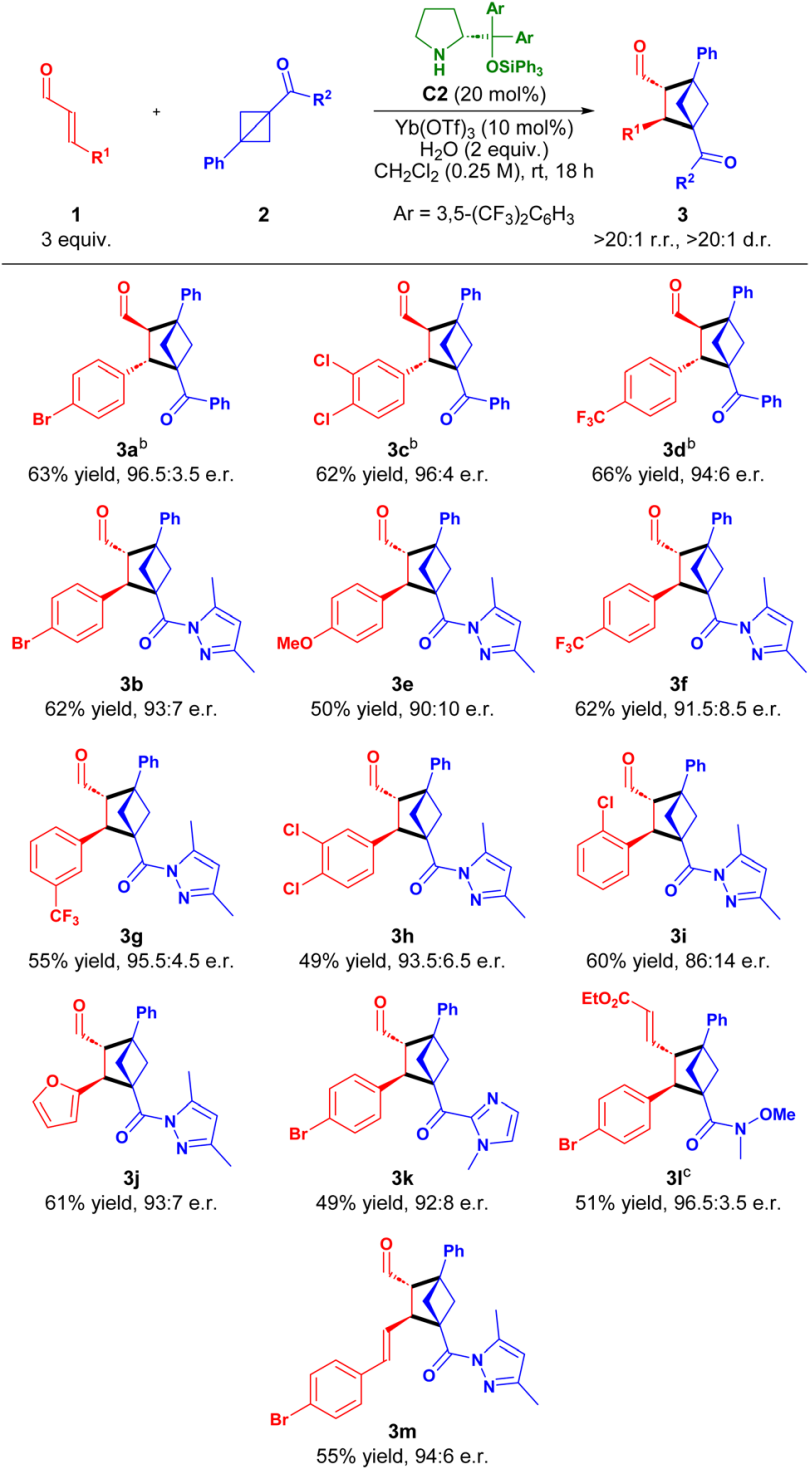

a Reaction conditions: 1 (3 equiv.), 2 (1 equiv.), C2 (20 mol%), Yb(OTf)_3_ (10 mol%), H_2_O (2 equiv.) in CH_2_Cl_2_ (0.25 M) for 18 h. Yield of isolated product. Regio- and diastereoisomeric ratios determined by ^1^H NMR spectroscopy of the reaction crude. e.r. determined by UPC^2^ analysis.

b With C1 (40 mol%) and stirred for 48 h.

c Isolated after *in situ* Wittig olefination.

For the [2 + 2] cycloaddition of *para*-substituted cinnamaldehydes 1a–c with disubstituted phenyl ketone BCB 2a, good yields of BCH-cycloadducts 3a,c,d were obtained with excellent enantiomeric ratio (up to 96.5 : 3.5 e.r.). Replacing the phenyl substituent of the BCB with a pyrazole (2b) afforded similar yields of the cycloadducts, without increasing catalyst loadings. For *para*- and *meta*-substituted cinnamaldehydes, cycloadducts 3b,e–h were formed in up to 62% yield; however, with a slight decrease in the enantiomeric ratio – 95.5 : 4.5 to 90 : 10 e.r. – compared to 3a (96.5 : 3.5 e.r.). For *ortho*-chloro cycloadduct 3i, the enantioselectivity was reduced to 86 : 14 e.r., presumably due to steric constraint. The furanyl-substituted α,β-unsaturated aldehyde reacted smoothly, yielding 3j in 61% yield and 93 : 7 e.r. *N*-methyl imidazole and Weinreb amide containing BCBs, 2c and 2d were also suitable substrates providing the corresponding BCH-cycloadducts 3k and 3l (after *in situ* Wittig olefination) in moderate yields with high enantiomeric ratios. Interestingly, dienal 1h reacted exclusively at the proximal olefin, leaving the distant one untouched, thereby generating 3m in 55% yield and 96 : 4 e.r.^20^ Replacing the aromatic functionality in the β-position with an alkyl (2-pentenal) only afforded the corresponding BCH-cycloadduct in trace amount (see SI).

The results for the [2 + 2] cycloaddition of cinnamic aldehydes with BCBs in Table 2 reveal the reactions to proceed in moderate to good yields and with high to excellent enantiomeric excess. We were therefore pleased to find that more activated α,β-unsaturated aldehydes, such as γ-ester-enals 4 reacted faster for the phenyl-ketone derived BCBs compared to cinnamaldehydes 1 without necessitating increased aminocatalyst loading in the [2 + 2] cycloaddition (Table 3). The reaction proved more efficient under more dilute conditions (0.1 M) (see SI).

**Table 3:** Scope of the [2 + 2] cycloaddition between γ-ester-enals 4 and BCBs 2^a^

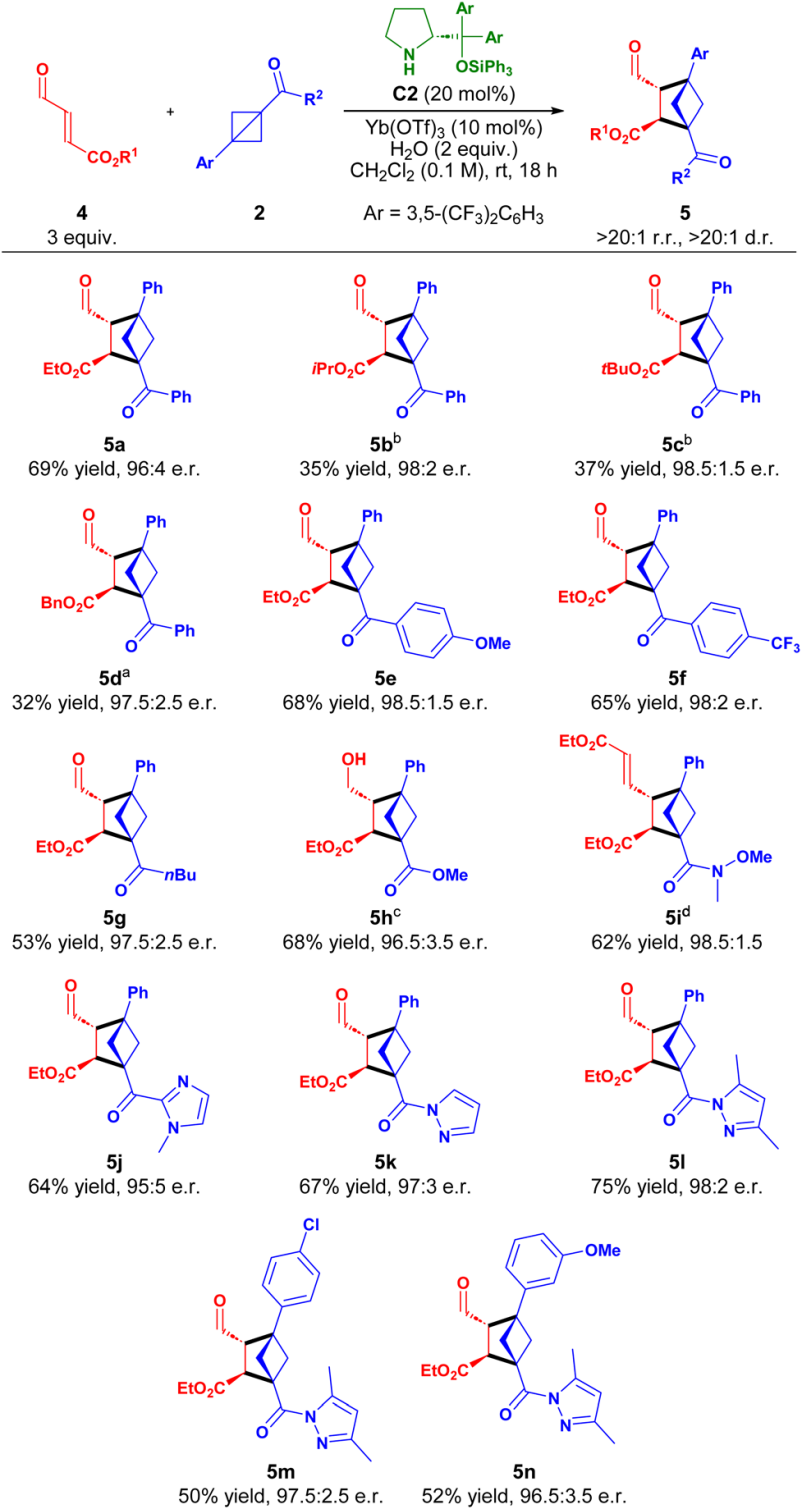

a Reaction conditions: 4 (3 equiv.), 2 (1 equiv.), C2 (20 mol%), Yb(OTf)_3_ (10 mol%), H_2_O (2 equiv.) in CH_2_Cl_2_ (0.1 M) for 18 h. Yield of isolated product. Regio- and diastereoisomeric ratios determined by ^1^H NMR spectroscopy of the reaction crude. e.r. determined by UPC^2^ analysis.

b Isolated at ∼40% conversion.

c Isolated after *in situ* NaBH_4_ reduction.

d Isolated after *in situ* Wittig olefination.

The [2 + 2] cycloaddition of ethyl γ-ester-enals 4a with BCB 2a efficiently afforded cycloadduct 5a in 69% yield and 96 : 4 e.r. For the different γ-ester-enals 4b–d a lower conversion (∼40%) was observed; thus, using the optimized reaction conditions for 5a provided 5b–d in reduced yields (32–37%), but maintaining excellent enantioselectivity (97.5 : 2.5 to 98.5 : 1.5 e.r.). Derivatization of the phenyl ketone moiety of 2a was also permitted, as both electron-donating and -withdrawing substituents reacted smoothly, affording 5e,f in good yields and excellent enantiomeric ratios (98 : 2 to 98.5 : 1.5 e.r.). Additionally, an aliphatic ketone proved viable, forming 5g in 53% yield with 97.5 : 2.5 e.r. Other carbonyl functionalities, such as esters and amides, on the BCB were also well-tolerated, yielding 5h,i in high yields and excellent enantiomeric ratios after NaBH_4_ reduction or Wittig olefination (up to 68% yield, 98.5 : 1.5 e.r.). Heteroaromatic carbonyls embedding an imidazole or pyrazoles were also feasible, furnishing 5j–l in 64–75% yield and 95 : 5–98 : 2 e.r. Finally, derivatization of the aryl group of 2b was possible, forming 5m,n in moderate yield and excellent enantioselectivity. Introducing a CF_3_-group at the *meta*-position of the aryl of 2b shut down reactivity, probably due to the destabilization of the benzylic carbocation of the zwitterionic intermediate (*vide infra* and see SI).

Encouraged by the generality and positive results for the reaction of γ-ester-enals 4, we next explored the reactivity of γ-keto-enals 6 in the [2 + 2] cycloaddition (Table 4).

**Table 4:** Scope of the [2 + 2] cycloaddition between γ-keto-enals 6 and BCBs 2^a^

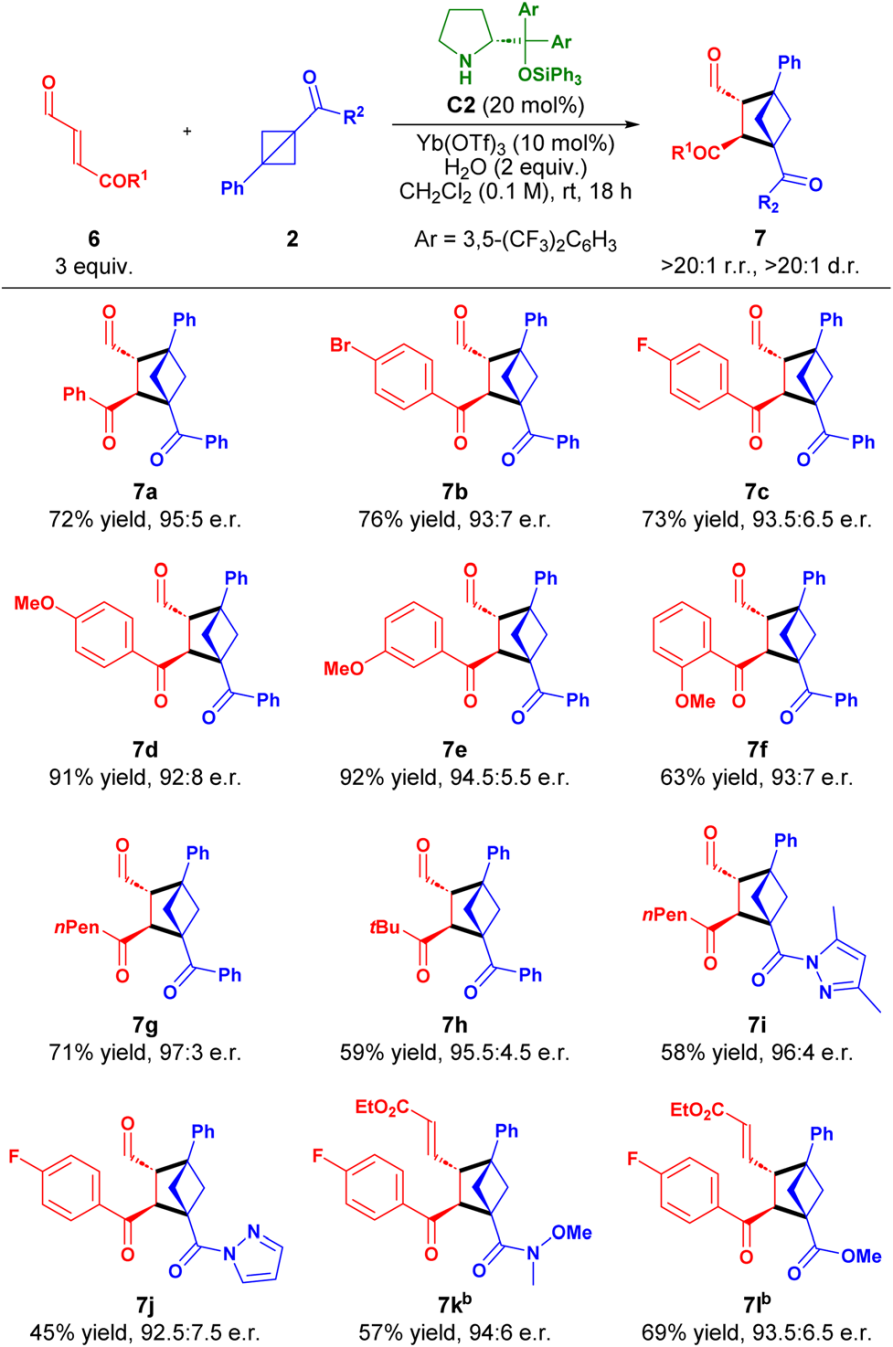

a Reaction conditions: 6 (3 equiv.), 2 (1 equiv.), C2 (20 mol%), Yb(OTf)_3_ (10 mol%), H_2_O (2 equiv.) in CH_2_Cl_2_ (0.1 M) for 18 h. Yield of isolated product. Regio- and diastereoisomeric ratios determined by ^1^H NMR spectroscopy of the reaction crude. e.r. determined by UPC^2^ analysis.

b Isolated after *in situ* Wittig olefination.

A series of substituted γ-keto-enals 6a–f bearing both electron-donating and -withdrawing substituents in *ortho*-, *meta*- and *para*-positions were well-tolerated, delivering BCHs 7a–f in high yields (63–92%) with high enantioselectivities (95 : 5–92 : 8 e.r.). Both linear and branched aliphatic γ-keto-enals 6g,h reacted efficiently, yielding cycloadducts 7g and 7h in 71% yield with 97 : 3 e.r. and 59% yield with 95.5 : 4.5 e.r., respectively. Additionally, pyrazole substituted BCB reacted with aliphatic and aromatic γ-keto-enals in a similar fashion affording 7i and 7j in 58 and 45% yield and 96 : 4 and 92.5 : 7.5 e.r., respectively. Furthermore, BCBs bearing a Weinreb amide and methyl ester functionality were well-tolerated after *in situ* Wittig transformation providing 7k,l in good yields and high enantioselectivities.

**Scheme 2:**
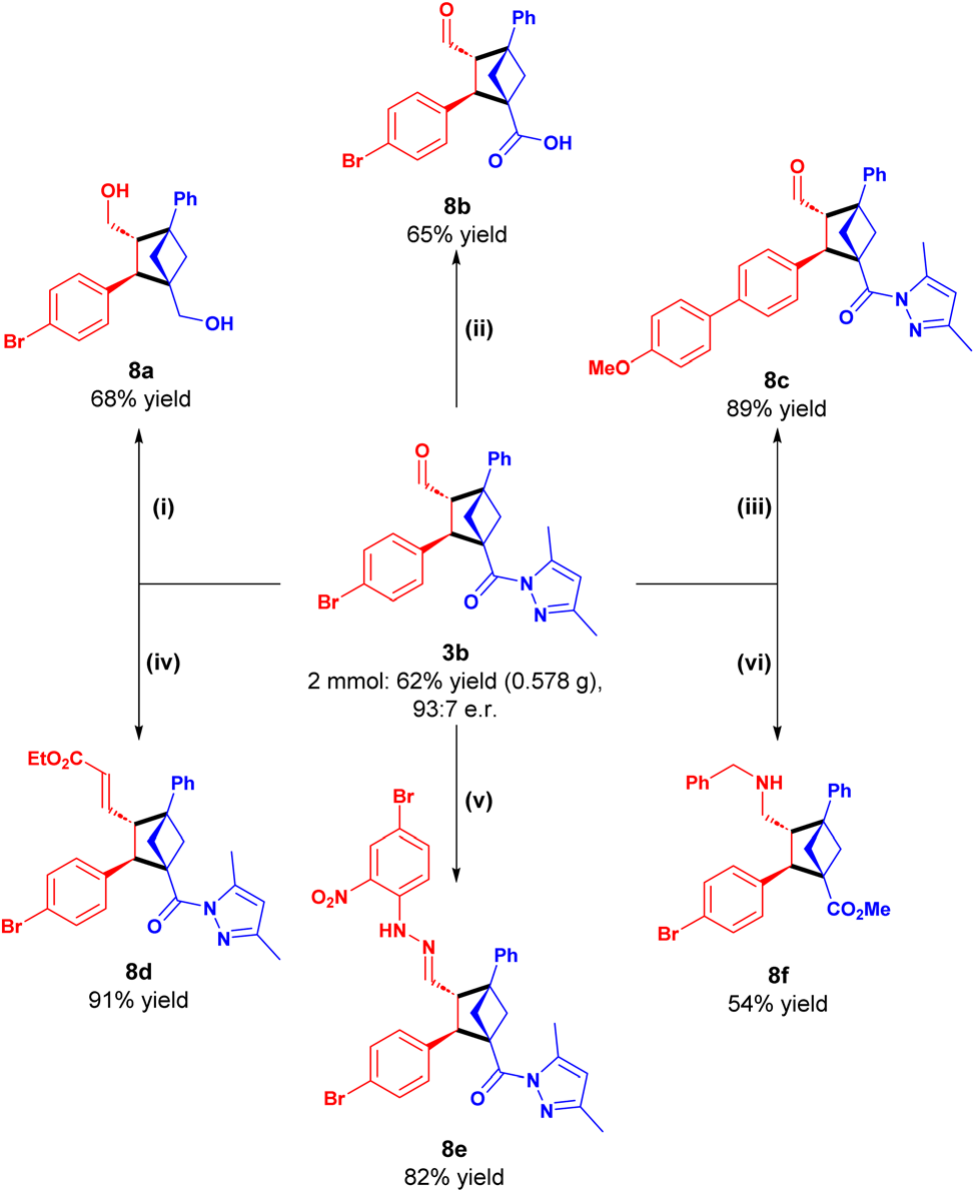
Scale-up and synthetic transformations of BCH 3b. Reaction conditions: (i) NaBH_4_ in CH_2_Cl_2_/MeOH; (ii) LiOH in THF/H_2_O; (iii) 4-MeO-Ph-B(OH)_2_, P(*o*-Tol)_3_, K_2_CO_3_ and Pd_2_dba_3_ in 1,4-dioxane/H_2_O at 50 °C; (iv) EtO_2_CCH=PPh_3_ in CH_2_Cl_2_; (v) 4-bromo-2-nitrophenylhydrazine hydrochloride in CH_2_Cl_2_/MeOH; (vi) BnNH_2_ and Et_3_N in CH_2_Cl_2_ then NaBH_4_ and MeOH. See SI for detailed reaction conditions.

Subsequently, to probe the synthetic utility of the methodology, several synthetic elaborations were performed to further decorate the BCHs (Scheme 2).

The acyl-pyrazole, aldehyde and phenyl-bromine moieties proved to be valuable handles for derivatization. Both aldehyde and acyl-pyrazole were efficiently and simultaneously reduced to diol 8a by treatment with NaBH_4_. The acyl-pyrazole was hydrolyzed to the carboxylic acid by subjection to aqueous LiOH, while leaving the aldehyde untouched, affording 8b in good yield. Additionally, 3b proved receptive towards a Suzuki–Miyaura cross-coupling in excellent yield to provide 8c. The aldehyde motif was also efficiently transformed into the corresponding alkene by a Wittig olefination (8d) or hydrazone (8e) by treatment with a hydrazine. Finally, to our surprise, when 3b was subjected to a one-pot imine formation and reduction by NaBH_4_ in the presence of MeOH, it simultaneously underwent acyl-substitution to the methyl ester, thereby affording 8f.

The relative configuration of the products was determined by X-ray analysis of 8e, and the absolute configuration of the BCHs was determined through comparison of the calculated electronic circular dichroism spectrum with the experimentally obtained spectrum for 3b (see SI).

**Scheme 3:**
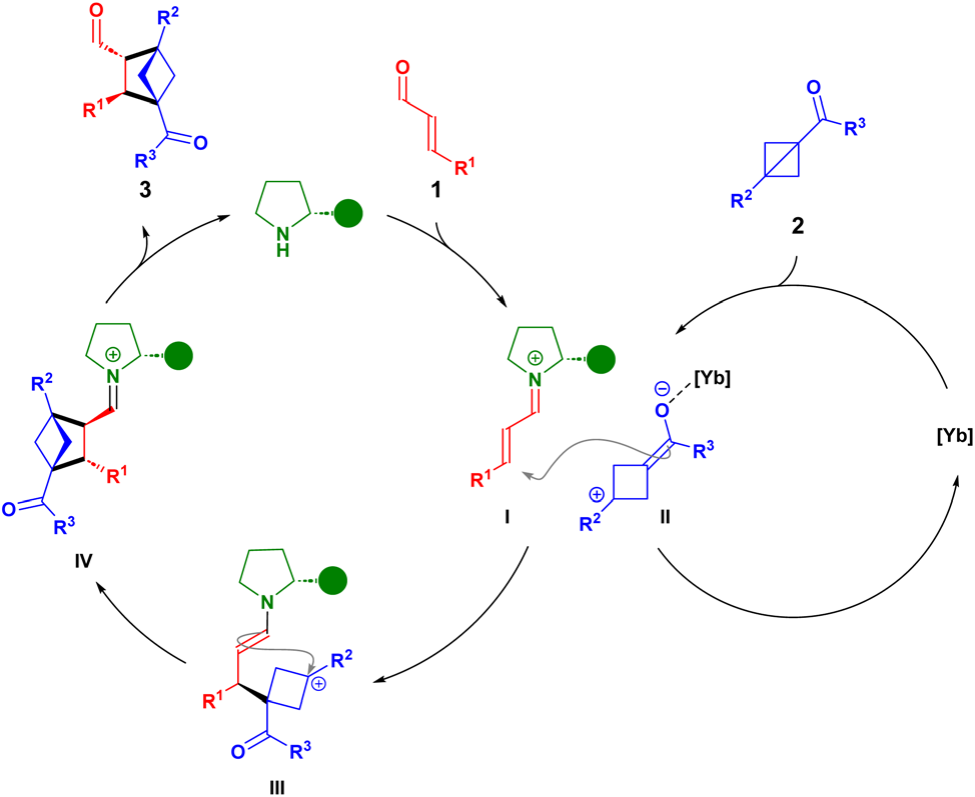
Proposed reaction mechanism.

Scheme 3 presents a proposal for the reaction mechanism, where the organocatalyst and Yb(iii) catalyst activate the α,β-unsaturated aldehyde and BCB, respectively, in a synergistic manner. In the first step, the aminocatalyst condenses with α,β-unsaturated aldehyde 1 forming iminium ion I. Simultaneously, BCB 2 is activated by Yb(OTf)_3_ thereby generating a zwitterionic intermediate II, where the nucleophilic carbon of the enolate attacks the electrophilic β-carbon of I in the stereo-determining step. This generates a transient enamine III which undergoes a highly diastereoselective intramolecular cyclization affording IV, which after hydrolysis liberates the aminocatalyst providing BCH 3. The proposed mechanism is supported by the observation that the introduction of an electron-withdrawing substituent in *R*^2^ shuts down reactivity (see SI). Furthermore, the transient nature of III is supported by the excellent diastereoselectivities obtained for the three classes of α,β-unsaturated aldehydes. The introduction of a step-wise mechanism is based on the observation by Hong *et al.* where it was demonstrated that a (*Z*)-olefin exclusively provides the same diastereoisomer as applying the (*E*)-isomer of the olefin.^7*b*^

## Conclusions

In conclusion, we have developed the first aminocatalytic enantioselective strategy achieving enantioenriched BCHs, based on the activation of α,β-unsaturated aldehydes. This is based on the *in situ* generation of an iminium-ion reacting with Yb(iii)-activated BCBs in a stereoselective [2 + 2] cycloaddition. The reaction is demonstrated to be tolerant towards multiple classes of both α,β-unsaturated aldehydes and BCBs affording BCHs in good-to-high yields and with high to excellent enantioselectivities. The formed BCHs contain multiple chemical handles that can be exploited to further expand the chemical complexity, such as a one-pot reductive amination of the aldehyde and acyl-substitution of the pyrazole functionality to the methyl ester. A mechanistic model is proposed to explain the developed methodology.

## Author contributions

R. S. B. conceived the project and devised the experiments with K. A. J., and E. M. R. S. B. and E. M. optimized the reaction conditions. R. S. B., E. M., E. B. O., I. R. K. and J. F. executed the experiments. R. S. B., E. M., E. B. O., I. R. K., J. F. and K. A. J. rationalized the experimental results. R. S. B. performed XRD analysis and measured ECD spectra. R. S. B. and K. A. J. wrote the initial draft of the manuscript. All authors participated in editing the manuscript and agreed on the final version.

## Conflicts of interest

There are no conflicts to declare.

## Supplementary Material

SC-016-D5SC05477J-s001

## Data Availability

CCDC 2463774 contains the supplementary crystallographic data for this paper.^21^ The SI contains experimental procedures, characterization data, NMR spectra, UPC^2^ chromatograms, X-ray crystallographic data for 8e (CCDC 2463774) and ECD measurements and calculations for 3b. See DOI: https://doi.org/10.1039/d5sc05477j.
