# Supplementary material for: Aminocatalytic enantioselective [2 + 2] cycloaddition of Bicyclo[1.1.0]butanes and α,β-unsaturated aldehydes
Source: Chem Sci. 2025 Aug 12;16(36):16567–72. doi: 10.1039/d5sc05477j (PMC12360369; doi:10.1039/d5sc05477j)
Supplement: SC-016-D5SC05477J-s001 [file SC-016-D5SC05477J-s001.pdf]

Supplementary Information (SI) for Chemical Science.
This journal is © The Royal Society of Chemistry 2025

Supplementary Information for

Aminocatalytic Enantioselective [2+2] Cycloaddition of Bicyclo[1.1.0]butanes and α,β-Unsaturated Aldehydes

René Slot Bitsch, Enrico Marcantonio, Erlaitz Basabe Obregón‡, Ida Rygaard Kocemba‡, Jonas Faghtmann‡ and Karl Anker Jørgensen*

Department of Chemistry, Aarhus University, DK-8000 Aarhus C, Denmark

* Corresponding author. E-mail: kaj@chem.au.dk

Table of Contents

1. General Information **S3**

2. Reaction Optimization **S4**

2.1 Summary of Optimizations S4

2.2 Initial Catalyst Screening S5

2.3 Lewis Acid Screening S6

2.4 Water Additive Screening S7

2.5 Solvent Screening S8

2.6 Stoichiometry Screening S9

2.7 γ-Ester-enal Optimization S10

3. Synthesis and Characterization of Starting Materials **S11**

3.1 Overview of Cinnamaldehydes **1**, γ-ester-enals **4** and γ-keto-enals **6** S11

3.2 Overview of BCBs **2** S14

4. Chiral Entries **S16**

4.1 General Procedures S16

4.1.1 General procedure 1 (GP1) for synthesis of BCHs **3** S16

4.1.1 General procedure 2 (GP2) for synthesis of BCHs **5** S16

4.1.1 General procedure 3 (GP3) for synthesis of BCHs **7** S16

4.3 Characterization of Chiral BCHs **3**, **5** and **7** S17

4.4 Unsuccessful substrates S37

4.5 Synthetic Transformations S38

5. Crystallographic Data **S42**

6. ECD and UV-vis Calculations for Determination of Absolute Stereochemistry of 3b **S44**

7. NMR **S48**

8. UPC2-Chromatograms **S107**

9. Geometries XYZ **S147**

References **S155**

1. General Information

All solvents were of high-performance liquid chromatography grade and were used as received except for solvents specified as dry, which were distilled prior to use. Commercially available reagents were used as received without further purification, unless specified. Moisture sensitive reactions were performed in flame-dried glassware. Analytical thin layer chromatography (TLC) was performed using pre-coated aluminum-backed plates (Merck® silica gel 60 F_{254}) and visualized by ultraviolet irradiation (254 nm) or $KMnO_4$ stain. Flash chromatography (FC) was performed using Sigma-Aldrich® silica gel high purity-grade (9385) (SiO_2 60, 230-400 mesh) or Iatrobeads. The NMR spectra were acquired on a Varian AS 400 MHz spectrometer (running at 400 MHz for ^{1}H, 100 MHz for ^{13}C and 376 MHz for ^{19}F). Chemical shifts (δ) are reported in ppm relative to residual solvent signal ($CHCl_3$: 7.26 ppm for ^{1}H NMR, and $CDCl_3$: 77.16 ppm for ^{13}C NMR, $CHDCl_2$: 5.32 ppm for ^{1}H NMR, and CD_2Cl_2: 54.00 ppm for ^{13}C NMR) The following abbreviations are used to indicate the multiplicity in NMR spectra: s = singlet, bs = broad singlet, d = doublet, dd = double doublet, dt = double triplet, dq = double quartet, ddd = double double doublet, dddd = double double double doublet, ddt = double double triplet, dtd = double triple doublet, t = triplet, td = triple doublet, tt = triple triplet, tdd = triple double doublet, q = quartet, qd = quartet of doublets, qt = quartet of triplets, hept = heptet and m = multiplet. Coupling constants are reported in Hertz (Hz) as the mean value between coupled hydrogen atoms. ^{13}C NMR spectra were acquired on a broadband decoupled mode. ^{19}F NMR spectra were acquired in decoupled mode. Mass spectra were recorded on a Bruker Maxis Impact mass spectrometer using electrospray ionization (ESI+) and with a TOF analyzer. Optical rotations were measured on a Bellingham+Stanley ADP440+ polarimeter, and $[\alpha]_D^T$ values are given in deg cm^3 g^{-1} dm^{-1}; concentrations, c, are listed in g 100 mL^{-1}. The enantiomeric ratio (e.r.) of the products was determined by Ultra Performance Convergence Chromatography (UPC^2) using Daicel Chiralpak IA-3, IB-3, IC-3 or ID-3 columns as chiral stationary phases. Racemic samples of compounds were prepared following the general procedures described below using (±)-**C1** as aminocatalyst (obtained by mixing the two pure enantiomers together). Therefore, a deviation from an expected 50:50 ratio of enantiomers in the UPC^2 may be observed. Crystallographic analysis was performed on a Bruker D8 VENTURE diffractometer with MoKα radiation (λ = 0.71073 Å) at 100 K.

2. Reaction Optimization

2.1 Summary of Optimizations

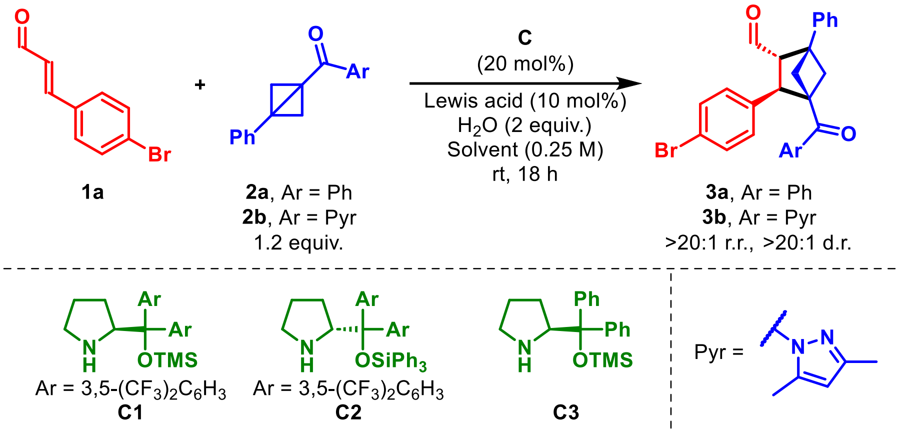

1a

2a, Ar = Ph
2b, Ar = Pyr
1.2 equiv.

3a, Ar = Ph
3b, Ar = Pyr
>20:1 r.r., >20:1 d.r.

Ar = 3,5-$(CF_3)_2C_6H_3$ **C1**

Ar = 3,5-$(CF_3)_2C_6H_3$ **C2**

C3

Entry	Solvent	BCB	LA	Catalyst	Conv. limiting reagent [%]	Yield [%][a]	e.r.
1[b]	CH_2Cl_2	**2a**	HFIP	**C1**	50	30	13:87
2	CH_2Cl_2	**2a**	$Yb(OTf)_3$	**C1**	50	31	3:97
3	CH_2Cl_2	**2a**	$Sc(OTf)_3$	**C1**	46	29	3:97
4	CH_2Cl_2	**2a**	$BF_3{\cdot}OEt_2$	**C1**	85	30	4:96
5	THF	**2a**	$Yb(OTf)_3$	**C1**	20	13	1.5:98.5
6	PhCl	**2a**	$Yb(OTf)_3$	**C1**	39	23	0.5:99.5
7[c]	CH_2Cl_2	**2a**	$Yb(OTf)_3$	**C1**	35	16	3:97
8[d]	CH_2Cl_2	**2a**	$Yb(OTf)_3$	**C1**	17	<5	-
9[e]	CH_2Cl_2	**2a**	$Yb(OTf)_3$	**C1**	81	41	3:97
10[f]	CH_2Cl_2	**2a**	$Yb(OTf)_3$	**C1**	55	35	3:97
11[f,g]	CH_2Cl_2	**2a**	$Yb(OTf)_3$	**C1**	>95	63[h]	3:97
12[f]	CH_2Cl_2	**2b**	$Yb(OTf)_3$	**C1**	>95	56	10:90
13[f]	CH_2Cl_2	**2b**	$Yb(OTf)_3$	**C2**	>95	62[h]	93:7
14[f]	CH_2Cl_2	**2b**	$Yb(OTf)_3$	**C3**	19	<5	-
15	CH_2Cl_2	**2a**	-	**C1**	5	<5	-
16	CH_2Cl_2	**2a**	$Yb(OTf)_3$	-	15	<5	-

Reaction conditions: **1a** (0.050 mmol), **2a** or **2b** (0.060 mmol), **C** (20 mol%), Lewis acid (10 mol%), H_2O (0.1 mmol) in solvent (0.2 mL) for 18 h. Regio- and diastereoisomeric ratios determined by ^1H NMR spectroscopy of the reaction crude. e.r. determined by chiral-phase ultraperformance convergence chromatography (UPC^2) analysis. [a] Determined by ^1H NMR spectroscopy of the reaction crude using 1,3,5-trimethoxybenzene as internal standard. [b] Using 10 equiv. of HFIP, and no addition of H_2O. [c] Using 10 equiv. of H_2O. [d] Using 2 spheres of 4Å MS and no addition of H_2O. [e] Using 40 mol% **C1**. [f] Using 3 equiv. of **1a** (0.15 mmol) and 1 equiv. of **2a** or **2b** (0.05 mmol). [g] Using 40 mol% **C1** and stirred for 48 h. [h] Isolated yield.

2.2 Initial Catalyst Screening

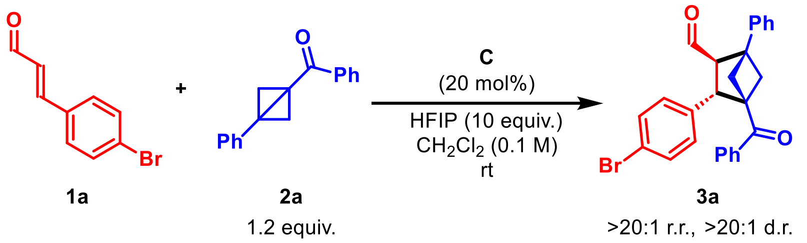

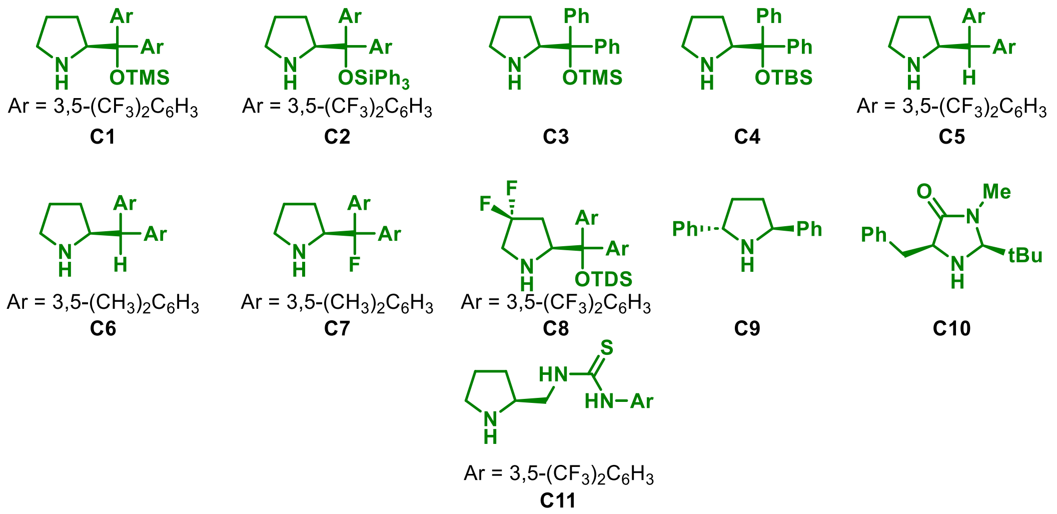

Entry	Catalyst	Reaction time (h)	Conv. **1a** [%]	Yield [%][a]	e.r.
1	**C1**	18	29	17	-
2[b]	**C1**	18	28	19	86:14
3[c]	**C1**	18	50	30	87:13
4	**C1**	72	67	31	86:14
5	**C2**	18	23	19	87:13
6	**C3**	18	40	-	-
7	**C4**	72	40	4	-
8	**C5**	18	42	25	61:39
9	**C6**	72	46	2	-
10	**C7**	18	16	2	-
11	**C8**	18	14	14	84:16
12	**C9**	72	93	34	84:16
13	**C10**	18	26	25	56:44
14	**C11**	18	10	-	-

Reaction conditions: **1a** (0.050 mmol), **2a** (0.060 mmol), **C** (20 mol%) and HFIP (0.5 mmol) in CH_2Cl_2 (0.5 mL). Regio- and diastereoisomeric ratios determined by ^1H NMR spectroscopy of the reaction crude. e.r. determined by chiral-phase ultraperformance convergence chromatography (UPC^2) analysis. [a] Determined

by ^1H NMR spectroscopy of the reaction crude using 1,3,5-trimethoxybenzene as internal standard. [b] At 40 °C. [c] In CH_2Cl_2 (0.25 M).

2.3 Lewis Acid Screening

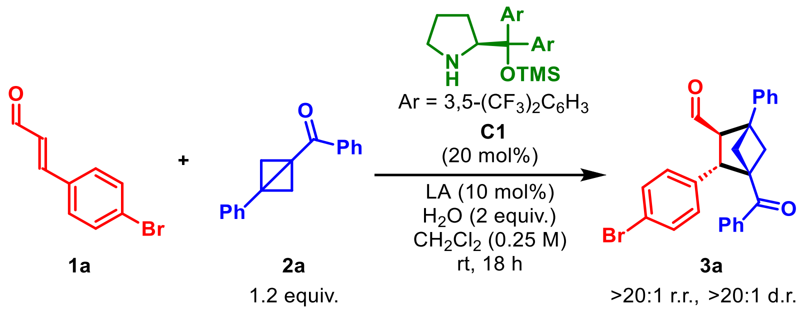

Entry	Lewis acid	Conv. **1a** [%]	Yield [%][a]	e.r.
1	$Yb(OTf)_3$	50	31	97:3
2	$Sc(OTf)_3$	46	29	97:3
3	AgOTf	45	18	93:7
5	$Zn(OTf)_2$	47	27	97:3
6	$Cu(OTf)_2$	60	25	97:3
7	$NiBr_2$	25	15	99:1
8	$Ni(OTf)_2$	28	18	98:2
9[b]	TMSOTf	75	26	97:3
10[b]	$BF_3{\cdot}OEt_2$	85	30	96:4
11[c]	$Yb(OTf)_3$	20	5	-
12[b]	$Sc(OTf)_3$	75	5	-
13[b]	$Zn(OTf)_2$	5	1	-
14[d]	$Yb(OTf)_3$	27	20	96:4
15[e]	$Yb(OTf)_3$	57	29	95: 5

Reaction conditions: **1a** (0.050 mmol), **2a** (0.060 mmol), **C1** (20 mol%), Lewis acid (10 mol%), H_2O (0.1 mmol) in CH_2Cl_2 (0.2 mL) for 18 h. Regio- and diastereoisomeric ratios determined by ^1H NMR spectroscopy of the reaction crude. e.r. determined by chiral-phase ultraperformance convergence chromatography (UPC^2) analysis. [a] Determined by ^1H NMR spectroscopy of the reaction crude using 1,3,5-trimethoxybenzene as internal standard. [b] Without addition of water. [c] With 2 spheres of 4 Å MS. [d] Using 30 mol% $Yb(OTf)_3$. [e] Using 5 mol% $Yb(OTf)_3$.

We realized the importance of water upon the purchase of a new batch of $Yb(OTf)_3$, which performance was vastly inferior to the previously used batch. This forced us to re-evaluate multiple Lewis acids that initially proved non-productive (*e.g.* $Sc(OTf)_3$ and $Zn(OTf)_2$) in the presence of water, which revealed similar reactivity compared to $Yb(OTf)_3$.

2.4 Water Additive Screening

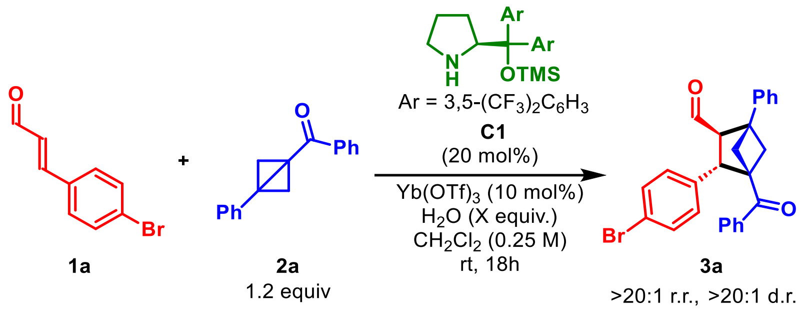

Entry	H_2O equiv.	Conv. **1a** [%]	Yield [%][a]	e.r.
1	0.5	14	5	-
2	1.0	42	20	97.5:2.5
3	2.0	60	30	97.5:2.5
4	3.0	65	30	97.5:2.5
5	5.0	60	27	97.5:2.5
6	10.0	35	16	97.5:2.5
7	20.0	40	18	97.5:2.5
8[b]	222	15	0	-

Reaction conditions: **1a** (0.050 mmol), **2a** (0.060 mmol), **C1** (20 mol%), $Yb(OTf)_3$ (10 mol%), in CH_2Cl_2 (0.2 mL) for 18 h. Regio- and diastereoisomeric ratios determined by ^1H NMR spectroscopy of the reaction crude. e.r. determined by chiral-phase ultraperformance convergence chromatography (UPC^2) analysis. [a] Determined by ^1H NMR spectroscopy of the reaction crude using 1,3,5-trimethoxybenzene as internal standard. [b] No CH_2Cl_2, run in 222 equiv. H_2O (0.2 mL).

2.5 Solvent Screening

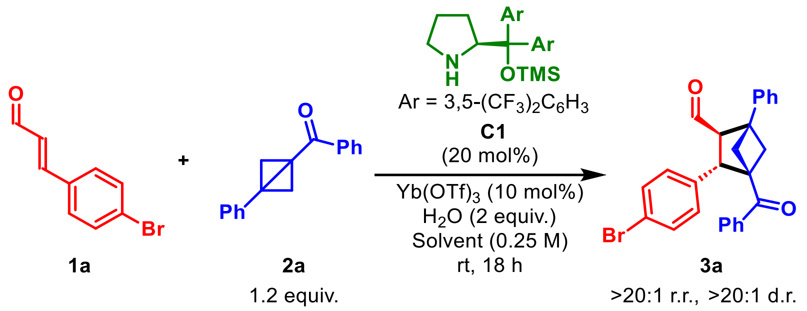

Entry	Solvent	Conv. **1a** [%]	Yield [%][a]	e.r.
1	CH_2Cl_2	60	30	97.5:2.5
2	MeCN	22	9	91.5:8.5
3	THF	20	13	98.5:1.5
4	Toluene	23	16	99.5:0.5
5	PhCl	39	23	99:1
6	EtOAc	10	10	97.5:2.5
7	$CDCl_3$	45	21	99:1
8	1,2-DCE	43	22	96:4
9	MeOH	90	<1	-
10	DMF	12	<1	-

Reaction conditions: **1a** (0.050 mmol), **2a** (0.060 mmol), **C1** (20 mol%), $Yb(OTf)_3$ (10 mol%), H_2O (0.1 mmol) in solvent (0.2 mL) for 18 h. Regio- and diastereoisomeric ratios determined by ^1H NMR spectroscopy of the reaction crude. e.r. determined by chiral-phase ultraperformance convergence chromatography (UPC^2) analysis. [a] Determined by ^1H NMR spectroscopy of the reaction crude using 1,3,5-trimethoxybenzene as internal standard.

2.6 Stoichiometry Screening

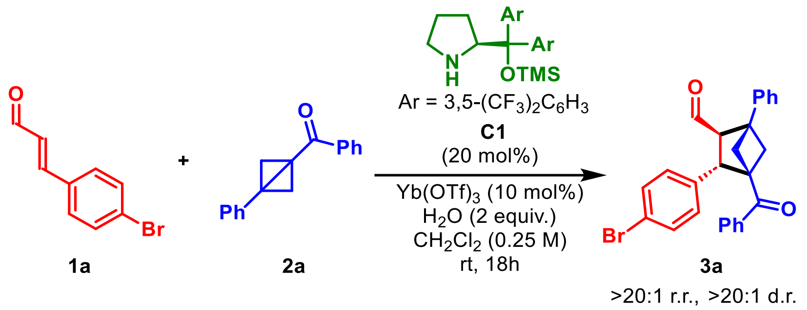

Entry	Equiv. **1a:2a**	Conv. **1a** [%]	Conv. **2a** [%]	Yield [%][a]	e.r.
1	1:2	30	15	18	97.5:2.5
2	2:1	55	50	28	98:2
3	3:1	50	55	35	97.5:2.5
4	5:1	40	70	36	98:2

Reaction conditions: **1a**, **2a**, **C1** (20 mol%), $Yb(OTf)_3$ (10 mol%), H_2O (0.1 mmol) in CH_2Cl_2 (0.2 mL) for 18 h. Regio- and diastereoisomeric ratios determined by ^1H NMR spectroscopy of the reaction crude. e.r. determined by chiral-phase ultraperformance convergence chromatography (UPC^2) analysis. [a] Determined by ^1H NMR spectroscopy of the reaction crude using 1,3,5-trimethoxybenzene as internal standard.

2.7 γ-Ester-enal Optimization

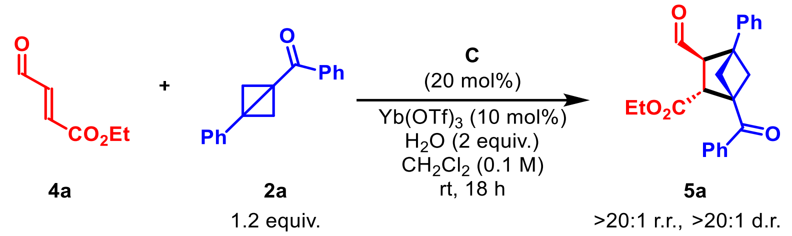

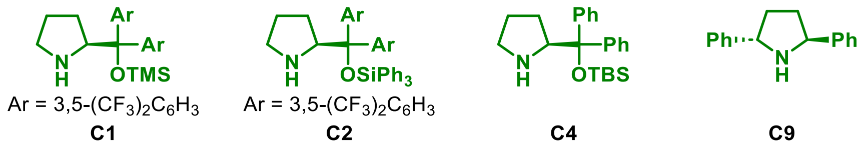

Entry	Deviation from above	Yield [%][a]	e.r.
1	None	42	95:5
2	HFIP (10 equiv.) instead of Yb	45	70:30
3	0.05 M instead of 0.1 M	54	94:6
4	4 °C instead of rt	40	96:4
5	-18 °C instead of rt	34	98:2
6	**C2** instead of **C1**	43	98:2
7	**C4** instead of **C1**	10	85:15
8	**C9** instead of **C1**	40	91:9
9	**4a**:**2a** 1.5:1 using **C2**	59	96:4
10	**4a**:**2a** 2:1 using **C2**	64	97:3
11	**4a**:**2a** 3:1 using **C2**	73 (69)	96:4
12	**4a**:**2a** 5:1 using **C2**	69	96:4
13	0.25 M using **C2** (3 equiv. **4a**)	63	96.5:3.5
14	0.5 M using **C2** (3 equiv. **4a**)	59	97:3

Regio- and diastereoisomeric ratios determined by ^1H NMR spectroscopy of the reaction crude. e.r. determined by chiral-phase ultraperformance convergence chromatography (UPC^2) analysis. [a] Determined by ^1H NMR spectroscopy of the reaction crude using 1,3,5-trimethoxybenzene as internal standard.

3. Synthesis and Characterization of Starting Materials

3.1 Overview of Cinnamaldehydes 1, γ-ester-enals 4 and γ-keto-enals 6

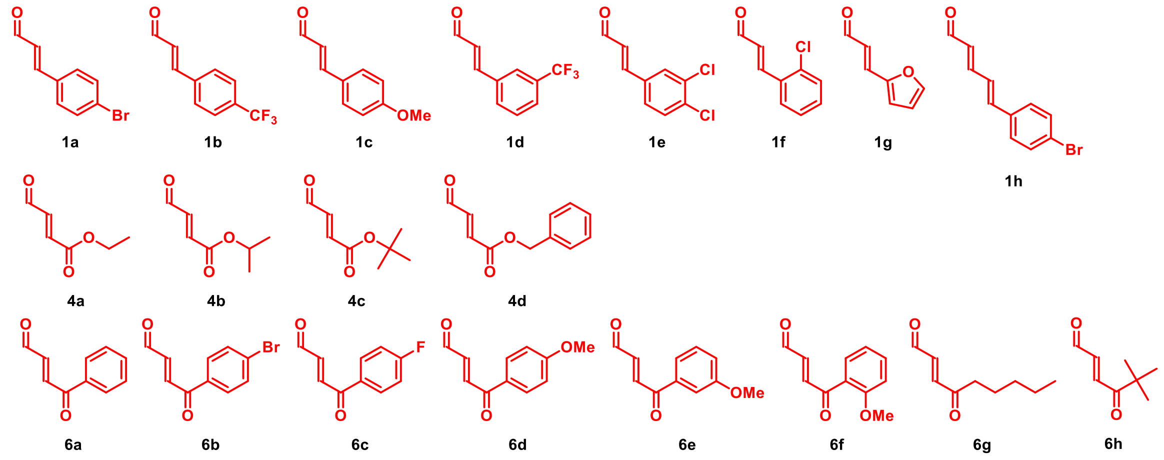

Compound	Reference
**1a**	Commercially available.
**1b**	Commercially available.
**1c**	Commercially available.
**1d**	Commercially available.
**1e**	*Org. Lett.* 2021, **23**, 6573-6577.
**1f**	*Synth. Commun.* 2018, **48**, 336-343.
**1g**	Commercially available.
**1h**	*Angew. Chem. Int. Ed.* 2015, **54**, 8203-8207.
**4a**	Commercially available.
**4b**	Described and characterized below.
**4c**	*Chem. Commun.* 2015, **51**, 10062-10065.
**4d**	*Angew. Chem. Int. Ed.* 2019, **58**, 12409-12414.
**6a**	*Chem. Eur. J.* 2016, **22**, 16810-16818.
**6b**	*Chem. Eur. J.* 2016, **22**, 16810-16818.
**6c**	*Chem. Eur. J.* 2016, **22**, 16810-16818.
**6d**	Described and characterized below.
**6e**	Described and characterized below.
**6f**	Described and characterized below.
**6g**	*Chem. Eur. J.* 2016, **22**, 16810-16818.
**6h**	*Chem. Eur. J.* 2016, **22**, 16810-16818.

Isopropyl (*E*)-4-oxobut-2-enoate, 4b

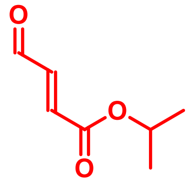

4b

Triethylphosphite (1 equiv. 8.0 mmol, 1.12 mL) and isopropyl 2-bromoacetate were (1 equiv. 8.0 mmol, 1.45 g) were mixed and stirred at 50 °C for 2 h. The solution was put under vacuum and left at 50 °C for a further 2 h, giving the phosphite as a colorless oil which was used without further purification. the crude isopropyl diethyl-phosphonoacetate was added to K_2CO_3 (1.3 equiv. 10.4 mmol, 1.44 g) in cyclohexane (7 mL) and the solution was stirred at 60 °C for 30 min. 2,2-dimethoxyacetaldehyde (1 equiv. 60 wt. % in H_2O, 8.0 mmol, 1.21 mL) was added dropwise and the reaction stirred a further 1 h at 60 °C. The reaction was cooled to rt and quenched with a sat. NH_4Cl solution. The crude was extracted with Et_2O (3 × 5 mL), washed with brine (5 mL), dried over $MgSO_4$ and concentrated *in vacuo* yielding the acetal as a colorless oil which was used without further purification.

p-Toluene sulfonic acid monohydrate (5 mol%, 0.4 mmol, 76 mg) was added to the crude dimethyl acetal and dissolved in a mixture of acetone (8 mL) and water (5 mL). The solution was then stirred at 80 °C for 30 min and cooled to rt. The reaction was neutralized with a sat. $NaHCO_3$ solution (30 mL), extracted with Et_2O (3 × 8 mL), washed with brine (5 mL), dried over $MgSO_4$ and concentrated *in vacuo*. The crude was purified by FC (Iatrobeads, 100:1 to 50:1 pentane:EtOAc) yielding **4b** as a yellow oil (269 mg, 1.89 mmol, 24%).

^{1}H NMR ($CDCl_3$, 400 MHz): δ [ppm] 9.69 (dd, *J* = 7.6, 1.2 Hz, 1H), 6.93 – 6.84 (m, 1H), 6.66 (dd, *J* = 16.0, 1.0 Hz, 1H), 5.14 – 4.99 (m, 1H), 1.24 (dd, *J* = 6.3, 1.3 Hz, 6H).

^{13}C NMR ($CDCl_3$, 100 MHz): δ [ppm] 192.7, 164.4, 140.1 (2C), 69.5, 21.7 (2C).

HRMS (ESI+): *m/z* calcd. for $C_7H_{11}O_3$ $[M+H]^+$: 143.0703 found: 143.0703.

(*E*)-4-(4-Methoxyphenyl)-4-oxobut-2-enal, 6d

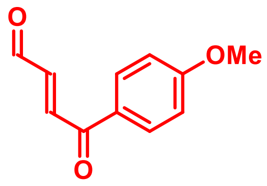

6d

To a solution of 2-bromo-1-(4-methoxyphenyl)ethan-1-one (1.0 equiv. 3 mmol, 687 mg) in MeCN (0.2 M) was added triphenylphosphine (1.05 equiv. 3.15 mmol, 826 mg) in one portion. The mixture was stirred at rt for 3 h. Afterwards, K_2CO_3 (1.2 equiv. 3.6 mmol, 498 mg) was added in one portion and the reaction mixture was stirred for an additional 3 h. The solid was removed by filtration and the crude filtrate was concentrated *in vacuo*. The crude ylide was dissolved in THF (0.2 M) and glyoxal was added (3.0 equiv., 40% aq. solution, 9 mmol, 1.10 mL) and the reaction mixture was stirred at rt overnight. The crude mixture was concentrated *in vacuo* and purified by FC (Iatrobeads, 4:1 to 2:1 pentane:EtOAc) to obtain **6d** as an orange solid (218 mg, 1.15 mmol, 38%).

^{1}H NMR ($CDCl_3$, 400 MHz): δ [ppm] 9.87 (d, *J* = 7.5 Hz, 1H), 8.06 – 7.95 (m, 2H), 7.72 (d, *J* = 15.7 Hz, 1H), 7.03 – 6.98 (m, 2H), 6.97 (dd, *J* = 15.8, 7.5 Hz, 1H), 3.90 (s, 3H).

^{13}C NMR ($CDCl_3$, 100 MHz): δ [ppm] 193.1, 188.0, 164.6, 142.6, 138.7, 131.5 (2C), 129.5, 114.4 (2C), 55.8.

HRMS (ESI+): *m/z* calcd. for $C_{11}H_{11}O_3$ $[M+H]^+$: 191.0703 found: 191.0705.

(*E*)-4-(3-Methoxyphenyl)-4-oxobut-2-enal, 6e

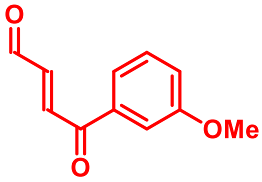

6e

1-(3-Methoxyphenyl)-2-(triphenyl-l5-phosphaneylidene)ethan-1-one (1 equiv., 2.0 mmol, 821 mg) was dissolved in THF (0.2 M) and glyoxal was added (3.0 equiv., 40% aq. solution, 6 mmol, 0.73 mL) and the reaction mixture was stirred at rt overnight. The crude mixture was concentrated *in vacuo* and purified by FC (Iatrobeads, 4:1 to 2:1 pentane:EtOAc) to obtain **6e** as a yellow solid (270 mg, 1.42 mmol, 71%).

^{1}H NMR ($CDCl_3$, 400 MHz): δ [ppm] 9.88 (d, *J* = 7.5 Hz, 1H), 7.69 (d, *J* = 15.8 Hz, 1H), 7.55 (ddd, *J* = 7.5, 1.6, 1.0 Hz, 1H), 7.50 (dd, *J* = 2.6, 1.6 Hz, 1H), 7.44 (t, *J* = 7.9 Hz, 1H), 7.19 (ddd, *J* = 8.2, 2.6, 1.0 Hz, 1H), 6.98 (dd, *J* = 15.8, 7.5 Hz, 1H), 3.88 (s, 3H).

^{13}C NMR ($CDCl_3$, 100 MHz): δ [ppm] 192.9, 189.6, 160.3, 142.2, 139.2, 137.7, 130.1, 121.7, 121.0, 112.9, 55.7.

HRMS (ESI+): *m/z* calcd. for $C_{11}H_{11}O_3$ $[M+H]^+$: 191.0703 found: 191.0699.

(*E*)-4-(2-Methoxyphenyl)-4-oxobut-2-enal, 6f

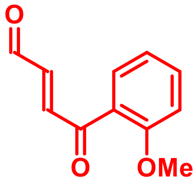

6f

To a solution of 2-bromo-1-(2-methoxyphenyl)ethan-1-one (1.0 equiv. 3 mmol, 687 mg) in MeCN (0.2 M) was added triphenylphosphine (1.05 equiv. 3.15 mmol, 826 mg) in one portion. The mixture was stirred at rt for 3 h. Afterwards, K_2CO_3 (1.2 equiv. 3.6 mmol, 498 mg) was added in one portion and the reaction mixture was stirred for an additional 3 h. The solid was removed by filtration and the crude filtrate was concentrated *in vacuo*. The crude ylide was dissolved in THF (0.2 M) and glyoxal was added (3.0 equiv., 40% aq. solution, 9 mmol, 1.10 mL) and the reaction mixture was stirred at rt overnight. The crude mixture was concentrated *in vacuo* and purified by FC (Iatrobeads, 4:1 to 2:1 pentane:EtOAc) to obtain **6f** as an orange solid (296 mg, 1.55 mmol, 52%).

^{1}H NMR ($CDCl_3$, 400 MHz): δ [ppm] 9.83 (d, *J* = 7.8 Hz, 1H), 7.72 (dd, *J* = 7.7, 1.8 Hz, 1H), 7.66 (d, *J* = 15.7 Hz, 1H), 7.55 (ddd, *J* = 8.4, 7.3, 1.8 Hz, 1H), 7.06 (td, *J* = 7.5, 0.9 Hz, 1H), 7.01 (d, *J* = 8.4 Hz, 1H), 6.86 (dd, *J* = 15.7, 7.7 Hz, 1H), 3.92 (s, 3H).

^{13}C NMR ($CDCl_3$, 100 MHz): δ [ppm] 193.7, 191.2, 159.1, 147.0, 136.9, 135.0, 131.1, 127.2, 121.3, 111.9, 55.8.

HRMS (ESI+): *m/z* calcd. for $C_{11}H_{11}O_3$ $[M+H]^+$: 191.0703 found: 191.0698.

3.2 Overview of BCBs 2

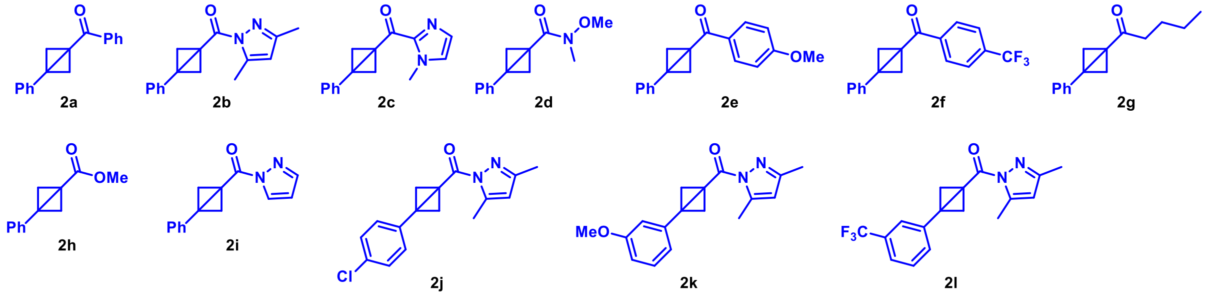

Compound	Reference
**2a**	*ACS Catal.* 2024, **14**, 13987-13998.
**2b**	*Angew. Chem. Int. Ed.* 2023, **62**, e202305043.
**2c**	*Angew. Chem. Int. Ed.* 2024, **63**, e202406548.
**2d**	*Angew. Chem. Int. Ed.* 2023, **62**, e202305043.
**2e**	*Org. Lett.* 2024, **26**, 1745-1750.
**2f**	*Org. Lett.* 2024, **26**, 1745-1750.
**2g**	*Nat. Commun.* 2024, **15**, 4374.
**2h**	*Angew. Chem. Int. Ed.* 2023, **62**, e202305043.
**2i**	*Angew. Chem. Int. Ed.* 2025, **64**, e202416781.
**2j**	*J. Am. Chem. Soc.* 2024, **146**, 34427-34441.
**2k**	*J. Am. Chem. Soc.* 2024, **146**, 34427-34441.
**2l**	Described and characterized below.

7-Oxocyclohepta-1,3,5-trien-1-yl trifluoromethanesulfonate, 2a

(3,5-Dimethyl-1*H*-pyrazol-1-yl)(3-(3-(trifluoromethyl)phenyl)bicyclo[1.1.0]butan-1-yl)methanone, 2l

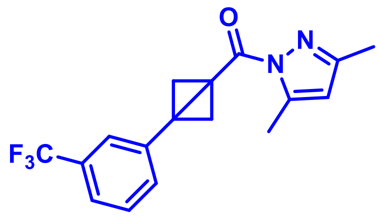

2l

Synthesized in four steps from 1-bromo-3-(trifluoromethyl)benzene as described by Lin *et al.*[1] After the final step, the crude product was purified by FC (silica, 10:1 pentane:Et_2O) to give **2l** as a white solid in 38% yield over four steps.

^{1}H NMR (400 MHz, $CDCl_3$): δ [ppm] 7.58-7.54 (m, 1H), 7.54-7.51 (m, 2H), 7.41-7.35 (m, 1H), 5.87-5.84 (m, 1H), 3.43 (t, *J* = 1.1 Hz, 2H), 2.24-2.22 (m, 6H), 1.89 (t, *J* = 1.1 Hz, 2H).

^{13}C NMR (100 MHz, $CDCl_3$): δ [ppm] 168.7, 151.9, 144.0, 135.0, 130.9 (q, *J* = 32.3 Hz), 129.7 (d, *J* = 1.0 Hz), 124.0 (q, *J* = 272.2 Hz), 123.9 (q, *J* = 3.8 Hz), 123.4 (q, *J* = 3.8 Hz) 40.4, 38.9 (2C), 37.1, 27.3, 14.1, 13.9.

^{19}F NMR (376 MHz, $CDCl_3$): δ [ppm] -62.8.

HRMS (ESI+) m/z calcd. for $C_{17}H_{16}F_3N_2O$ $[M+H]^+$: 321.1210; found: 321.1217.

4. Chiral Entries

4.1 General Procedures

4.1.1 General procedure 1 (GP1) for synthesis of BCHs 3

A 1 mL screw-capped vial equipped with a magnetic stirring-bar was charged with cinnamaldehyde **1** (3 equiv.), BCB **2** (1 equiv.), catalyst **C2** (20 mol%) and $Yb(OTf)_3$ (10 mol%). To this was added CH_2Cl_2 (0.25 M) and H_2O (2 equiv.). The vial was capped and stirred at rt. After full conversion was achieved, the crude was directly purified by FC to yield BCH **3**.

4.1.1 General procedure 2 (GP2) for synthesis of BCHs 5

A 4 mL screw-capped vial equipped with a magnetic stirring-bar was charged with γ-ester-enal **4** (3 equiv.), BCB **2** (1 equiv.), catalyst **C2** (20 mol%) and $Yb(OTf)_3$ (10 mol%). To this was added CH_2Cl_2 (0.10 M) and H_2O (2 equiv.). The vial was capped and stirred at rt. After full conversion was achieved, the crude was directly purified by FC to yield BCH **5**.

4.1.1 General procedure 3 (GP3) for synthesis of BCHs 7

A 4 mL screw-capped vial equipped with a magnetic stirring-bar was charged with γ-keto-enal **6** (3 equiv.), BCB **2** (1 equiv.), catalyst **C2** (20 mol%) and $Yb(OTf)_3$ (10 mol%). To this was added CH_2Cl_2 (0.10 M) and H_2O (2 equiv.). The vial was capped and stirred at rt. After full conversion was achieved, the crude was directly purified by FC to yield BCH **7**.

4.3 Characterization of Chiral BCHs 3, 5 and 7

(2*R*,3*S*)-4-Benzoyl-3-(4-bromophenyl)-1-phenylbicyclo[2.1.1]hexane-2-carbaldehyde, 3a

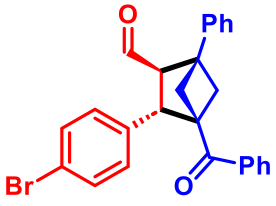

3a

Performed on 0.10 mmol scale following GP1, however using **C1** (40 mol%) instead. Full conversion was achieved in 2 d. Purified by FC (silica gel, 20:2:1 pentane:CH_2Cl_2:Et_2O) to obtain **3a** (63% yield, 27.9 mg, 0.063 mmol, >20:1 d.r., >20:1 r.r., 96.5:3.5 e.r.) as a colorless oil.

^{1}H NMR ($CDCl_3$, 400 MHz): δ [ppm] 9.75 (d, *J* = 1.3 Hz, 1H), 7.85 – 7.80 (m, 2H), 7.58 – 7.52 (m, 1H), 7.47 – 7.41 (m, 2H), 7.41 – 7.35 (m, 2H), 7.35 – 7.27 (m, 3H), 7.27 – 7.23 (m, 2H), 7.07 – 7.02 (m, 2H), 4.54 (dd, *J* = 4.6, 2.1 Hz, 1H), 3.47 (dt, *J* = 4.5, 1.8 Hz, 1H), 2.75 – 2.66 (m, 2H), 2.44 (dt, *J* = 5.7, 2.0 Hz, 1H), 2.35 (dt, *J* = 5.9, 2.0 Hz, 1H).

^{13}C NMR ($CDCl_3$, 100 MHz): δ [ppm] 202.8, 200.5, 139.3, 139.2, 136.3, 133.3, 131.8 (2C), 129.4 (2C), 129.0 (2C), 128.9 (2C), 128.4 (2C), 127.6, 125.9 (2C), 121.0, 64.6, 58.8, 53.2, 49.4, 46.4, 45.3.

HRMS (ESI+): *m/z* calcd. for $C_{26}H_{22}{}^{79}BrO_2$ $[M+H]^+$: 445.0798; found: 445.0802. *m/z* calcd. for $C_{26}H_{22}{}^{81}BrO_2$ $[M+H]^+$: 447.0778; found: 447.0784.

UPC^2: ID-3, CO_2:MeCN, gradient [99:1 (0.5 min), 99:1 to 60:40 (over 4 min), 60:40 (1.5 min)], 120 bar, 40 °C, 3.0 mL min^{-1}; t_{major} = 4.04 min; t_{minor} = 4.47 min, 96.5:3.5 e.r.

$[\alpha]_D^{298\,K}$ = +10.4 (*c* 1.07, CH_2Cl_2) for 96.5:3.5 e.r.

(2*S*,3*R*)-3-(4-Bromophenyl)-4-(3,5-dimethyl-1*H*-pyrazole-1-carbonyl)-1-phenylbicyclo[2.1.1]hexane-2-carbaldehyde, 3b

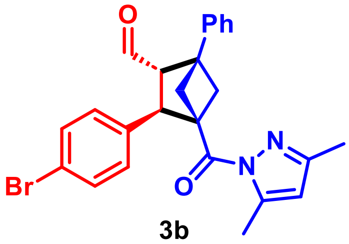

3b

Performed on 0.050 mmol scale following GP1. Full conversion was achieved in 18 h. Purified by FC (silica gel, 20:1 pentane:Et_2O) to obtain **3b** (62% yield, 14.2 mg, 0.031 mmol, >20:1 d.r., >20:1 r.r., 93:7 e.r.) as a white solid.

Scale-up: In a 50-mL round bottom flash **C2** (313 mg, 0.4 mmol, 0.2 equiv.), **1a** (1266 mg, 6 mmol, 3.0 equiv.) and $Yb(OTf)_3$ (124 mg, 0.2 mmol, 0.1 equiv.) was dissolved in CH_2Cl_2 (8 mL). Water (72 µL, 4 mmol, 2.0 equiv.) was added, and finally the bicyclobutane **2b** (505 mg, 2.0 mmol, 1.0 equiv.) was added. The mixture was stirred at rt for 25 h. The solvent was removed by a flow of N_2 gas, and the crude residue was purified by FC (silica, pentane to 20:1 pentane:Et_2O) which afforded **2b** as a white solid (62% yield, 578 mg, 1.25 mmol, >20:1 d.r., >20:1 r.r., 93:7 e.r.).

^{1}H NMR ($CDCl_3$, 400 MHz): δ [ppm] 9.76 (d, *J* = 2.3 Hz, 1H), 7.40 – 7.33 (m, 4H), 7.30 – 7.27 (m, 1H), 7.26 – 7.23 (m, 2H), 7.11 – 7.06 (m, 2H), 5.93 (d, *J* = 1.1 Hz, 1H), 4.78 (dd, *J* = 4.9, 2.0 Hz, 1H), 3.30 (dt, *J* = 4.8, 2.3 Hz, 1H), 2.85 (dd, *J* = 9.4, 7.6 Hz, 1H), 2.61 (dd, *J* = 9.4, 7.7 Hz, 1H), 2.48 (dd, *J* = 7.6, 2.1 Hz, 1H), 2.44 (d, *J* = 1.1 Hz, 3H), 2.27 (dd, *J* = 7.7, 1.8 Hz, 1H), 2.25 (s, 3H).

^{13}C NMR ($CDCl_3$, 100 MHz): δ [ppm] 202.7, 172.2, 152.8, 144.2, 139.7, 139.6, 131.8 (2C), 129.3 (2C), 128.9 (2C), 127.4, 126.1 (2C), 120.9, 111.0, 63.9, 55.5, 52.5, 49.9, 47.0, 44.8, 14.4, 14.2.

HRMS (ESI+): *m/z* calcd. for $C_{25}H_{24}{}^{79}BrN_2O_2$ $[M+H]^+$: 463.1016; found: 463.1024. *m/z* calcd. for $C_{25}H_{24}{}^{81}BrN_2O_2$ $[M+H]^+$: 465.0996; found: 465.1006.

UPC^2: IB-3, CO_2:MeCN, gradient [99:1 (0.5 min), 99:1 to 60:40 (over 4 min), 60:40 (1.5 min)], 120 bar, 40 °C, 3.0 mL min^{-1}; t_{major} = 3.84 min; t_{minor} = 3.65 min, 93:7 e.r.

$[\alpha]_D^{298\,K}$ = -12.2 (*c* 1.0, CH_2Cl_2) for 93:7 e.r.

(2*R*,3*S*)-4-Benzoyl-3-(3,4-dichlorophenyl)-1-phenylbicyclo[2.1.1]hexane-2-carbaldehyde, 3c

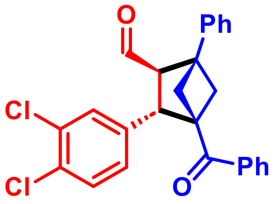

3c

Performed on 0.10 mmol scale following GP1, however using **C1** (40 mol%) instead. Full conversion was achieved in 2 d. Purified by FC (silica gel, 20:2:1 pentane:CH_2Cl_2:Et_2O) to obtain **3c** (62% yield, 27.0 mg, 0.062 mmol, >20:1 d.r., >20:1 r.r., 96:4 e.r.) as a colorless oil.

^{1}H NMR ($CDCl_3$, 400 MHz): δ [ppm] 9.74 (d, *J* = 1.1 Hz, 1H), 7.88 – 7.80 (m, 2H), 7.62 – 7.53 (m, 1H), 7.49 – 7.43 (m, 2H), 7.42 – 7.36 (m, 2H), 7.34 – 7.29 (m, 1H), 7.29 – 7.24 (m, 4H), 7.00 (ddd, *J* = 8.3, 2.3, 0.6 Hz, 1H), 4.53 (dd, *J* = 4.5, 2.1 Hz, 1H), 3.49 – 3.42 (m, 1H), 2.69 (dd, *J* = 5.1, 2.3 Hz, 2H), 2.46 (dt, *J* = 4.9, 2.0 Hz, 1H), 2.38 (dt, *J* = 5.3, 2.1 Hz, 1H).

^{13}C NMR ($CDCl_3$, 100 MHz): δ [ppm] 202.5, 200.2, 140.5, 139.1, 136.3, 133.5, 132.8, 131.2, 130.6, 129.8, 129.0 (2C), 129.0 (2C), 128.4 (2C), 127.7, 127.1, 125.9 (2C), 64.6, 58.8, 53.2, 48.9, 46.3, 45.4.

HRMS (ESI+): *m/z* calcd. for $C_{26}H_{21}Cl_2O_2$ $[M+H]^+$: 435.0913; found: 435.0910.

UPC^2: ID-3, CO_2:MeCN, gradient [99:1 (0.5 min), 99:1 to 60:40 (over 4 min), 60:40 (1.5 min)], 120 bar, 40 °C, 3.0 mL min^{-1}; t_{major} = 3.81 min; t_{minor} = 4.04 min, 96:4 e.r.

$[\alpha]_D^{298\,K}$ = +23.8 (*c* 1.67, CH_2Cl_2) for 96:4 e.r.

(2*R*,3*S*)-4-Benzoyl-3-(4-(trifluoromethyl)phenyl)-1-phenylbicyclo[2.1.1]hexane-2-carbaldehyde, 3d

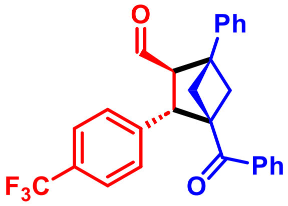

3d

Performed on 0.10 mmol scale following GP1, however using **C1** (40 mol%) instead. Full conversion was achieved in 2 d. Purified by FC (silica gel, 20:2:1 pentane:CH_2Cl_2:Et_2O) to obtain **3d** (66% yield, 28.6 mg, 0.066 mmol, >20:1 d.r., >20:1 r.r., 94:6 e.r.) as a colorless oil.

^{1}H NMR ($CDCl_3$, 400 MHz): δ [ppm] 9.76 (d, *J* = 1.2 Hz, 1H), 7.87 – 7.81 (m, 2H), 7.58 – 7.52 (m, 1H), 7.50 – 7.41 (m, 5H), 7.41 – 7.36 (m, 2H), 7.34 – 7.24 (m, 7H), 4.69 – 4.62 (m, 1H), 3.49 (dq, *J* = 4.5, 1.4 Hz, 1H), 2.77 – 2.66 (m, 2H), 2.47 (dt, *J* = 4.4, 2.0 Hz, 1H), 2.40 (dt, *J* = 4.9, 2.0 Hz, 1H).

^{13}C NMR ($CDCl_3$, 100 MHz): δ [ppm] 202.6, 200.4, 144.4, 139.2, 136.3, 133.4, 129.3 (d, *J* = 32.3 Hz), 129.0 (2C), 129.0 (2C), 128.4 (2C), 128.1 (2C), 127.7, 125.9 (2C), 125.7 (q, *J* = 3.7 Hz, 2C), 124.1 (d, *J* = 271.8 Hz), 64.8, 58.7, 53.3, 49.5, 46.4, 45.5.

^{19}F NMR ($CDCl_3$, 376 MHz): δ [ppm] – 62.6.

HRMS (ESI+): *m/z* calcd. for $C_{27}H_{22}F_3O_2$ $[M+H]^+$: 435.1566; found: 435.1560.

UPC^2: ID-3, CO_2:MeCN, gradient [99:1 (0.5 min), 99:1 to 60:40 (over 4 min), 60:40 (1.5 min)], 120 bar, 40 °C, 3.0 mL min^{-1}; t_{major} = 2.92 min; t_{minor} = 3.20 min, 94:6 e.r.

$[\alpha]_D^{298\,K}$ = +15.4 (*c* 0.83, CH_2Cl_2) for 94:6 e.r.

(2*S*,3*R*)-3-(4-Methoxyphenyl)-4-(3,5-dimethyl-1*H*-pyrazole-1-carbonyl)-1-phenylbicyclo[2.1.1]hexane-2-carbaldehyde, 3e

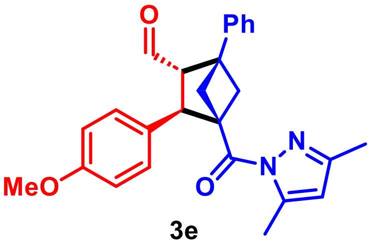

3e

Performed on 0.050 mmol scale following GP1. Full conversion was achieved in 24 h. Purified by FC (silica gel, 20:1 to 10:1 pentane:Et_2O) to obtain **3e** (50% yield, 10.4 mg, 0.025 mmol, >20:1 d.r., >20:1 r.r., 90:10 e.r.) as a colorless oil.

^{1}H NMR ($CDCl_3$, 400 MHz): δ [ppm] 9.79 (d, *J* = 2.7 Hz, 1H), 7.38 – 7.33 (m, 2H), 7.29 – 7.24 (m, 3H), 7.13 – 7.08 (m, 2H), 6.82 – 6.76 (m, 2H), 5.93 (d, *J* = 1.1 Hz, 1H), 4.75 (dd, *J* = 4.9, 2.0 Hz, 1H), 3.76 (s, 3H), 3.34 (dt, *J* = 4.8, 2.4 Hz, 1H), 2.90 (dd, *J* = 9.4, 7.5 Hz, 1H), 2.64 (dd, *J* = 9.4, 7.7 Hz, 1H), 2.48 (dd, *J* = 7.5, 2.1 Hz, 1H), 2.43 (d, *J* = 1.1 Hz, 3H), 2.26 (s, 3H), 2.23 (dd, *J* = 7.6, 1.8 Hz, 1H).

^{13}C NMR ($CDCl_3$, 100 MHz): δ [ppm] 203.1, 172.5, 158.5, 152.6, 144.2, 140.0, 132.4, 128.8 (2C), 128.6 (2C), 127.3, 126.2 (2C), 114.1 (2C), 110.9, 63.8, 55.9, 55.3, 52.5, 50.0, 47.1, 44.6, 14.4, 14.2.

HRMS (ESI+): *m/z* calcd. for $C_{26}H_{27}N_2O_3$ $[M+H]^+$: 415.2016; found: 415.2020.

UPC^2: IB-3, CO_2:MeCN, gradient [99:1 (0.5 min), 99:1 to 60:40 (over 4 min), 60:40 (1.5 min)], 120 bar, 40 °C, 3.0 mL min^{-1}; t_{major} = 3.70 min; t_{minor} = 3.56 min, 90:10 e.r.

$[\alpha]_D^{298\,K}$ = -26.9 (*c* 0.83, CH_2Cl_2) for 90:10 e.r.

(2*S*,3*R*)-3-(4-(Trifluoromethyl)phenyl)-4-(3,5-dimethyl-1*H*-pyrazole-1-carbonyl)-1-phenylbicyclo[2.1.1]hexane-2-carbaldehyde, 3f

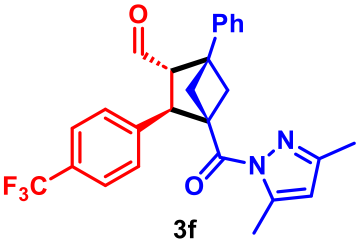

3f

Performed on 0.050 mmol scale following GP1. Full conversion was achieved in 18 h. Purified by FC (silica gel, 20:1 pentane:Et_2O) to obtain **3f** (62% yield, 14.1 mg, 0.031 mmol, >20:1 d.r., >20:1 r.r., 92:8 e.r.) as a colorless oil.

^{1}H NMR ($CDCl_3$, 400 MHz): δ [ppm] 9.77 (d, *J* = 2.2 Hz, 1H), 7.54 – 7.49 (m, 2H), 7.39 – 7.32 (m, 4H), 7.31 – 7.28 (m, 1H), 7.26 (d, *J* = 1.5 Hz, 1H), 7.25 – 7.23 (m, 1H), 5.93 (d, *J* = 1.2 Hz, 1H), 4.91 (d, *J* = 4.5 Hz, 1H), 3.32 (dt, *J* = 4.5, 2.2 Hz, 1H), 2.85 (dd, *J* = 9.4, 7.5 Hz, 1H), 2.64 (dd, *J* = 9.5, 7.8 Hz, 1H), 2.50 (dd, *J* = 7.6, 2.1 Hz, 1H), 2.45 (d, *J* = 1.1 Hz, 3H), 2.33 (dd, *J* = 7.7, 1.8 Hz, 1H), 2.24 (s, 3H).

^{13}C NMR ($CDCl_3$, 100 MHz): δ [ppm] 202.5, 172.2, 152.9, 145.0 (d, *J* = 1.5 Hz), 144.3, 139.5, 129.1 (d, *J* = 32.2 Hz), 128.9 (2C), 127.9 (2C), 127.5, 126.1 (2C), 125.7 (q, *J* = 4.0 Hz, 2C), 111.1, 64.1, 55.3, 52.6, 50.0, 47.0, 45.0, 14.4, 14.1.

^{19}F NMR ($CDCl_3$, 376 MHz): δ [ppm] – 62.5.

HRMS (ESI+): *m/z* calcd. for $C_{26}H_{24}F_3N_2O_2$ $[M+H]^+$: 453.1784; found: 453.1790.

UPC^2: IB-3, CO_2:MeCN, gradient [99:1 (0.5 min), 99:1 to 60:40 (over 4 min), 60:40 (1.5 min)], 120 bar, 40 °C, 3.0 mL min^{-1}; t_{major} = 3.05 min; t_{minor} = 2.84 min, 92:8 e.r.

$[\alpha]_D^{298\,K}$ = -38.3 (*c* 1.0, CH_2Cl_2) for 92:8 e.r.

(2S,3R)-4-(3,5-Dimethyl-1H-pyrazole-1-carbonyl)-1-phenyl-3-(3-(trifluoromethyl)phenyl)bicyclo[2.1.1]hexane-2-carbaldehyde, 3g

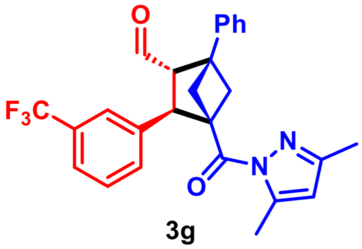

3g

Performed on 0.050 mmol scale following GP1. Full conversion was achieved in 18 h. Purified by FC (silica gel, 10:1 pentane :Et_2O) to obtain **3g** (55% yield, 12.4 mg, 0.028 mmol, >20:1 d.r., >20:1 r.r., 95.5:4.5 e.r.) as a yellow oil.

^{1}H NMR ($CDCl_3$, 400 MHz): δ [ppm] 9.93 (d, J = 2.2 Hz, 1H), 7.64 – 7.49 (m, 6H), 7.49 – 7.40 (m, 3H), 6.10 (d, J = 1.1 Hz, 1H), 5.02 (dd, J = 4.8, 1.7 Hz, 1H), 3.54 (dt, J = 4.5, 2.1 Hz, 1H), 3.02 (dd, J = 9.5, 7.5 Hz, 1H), 2.78 (dd, J = 9.5, 7.8 Hz, 1H), 2.66 (dd, J = 7.6, 2.1 Hz, 1H), 2.58 (d, J = 1.0 Hz, 3H), 2.46 (dd, J = 7.8, 1.8 Hz, 1H), 2.41 (s, 3H).

^{13}C NMR ($CDCl_3$, 100 MHz): δ [ppm] 202.3, 171.9, 152.8, 144.2, 141.4, 139.4, 130.9 (q, J = 32 Hz), 130.7, 129.1, 128.8 (2C), 127.4, 126.0 (2C), 124.4 (q, J = 4 Hz), 124.1 (q, J = 273 Hz), 123.7 (q, J = 4 Hz), 110.9, 63.2, 55.5, 52.6, 49.9, 46.8, 44.7, 14.2, 14.0.

^{19}F NMR ($CDCl_3$, 376 MHz): δ – 62.7 (s).

HRMS (ESI+): m/z calcd. for $C_{26}H_{24}F_3N_2O_2$ $[M+H]^+$: 453.1784; found: 453.1780.

Note: Since no suitable conditions were found to separate the two enantiomers of **3g** using UPC^2, **3g** was reduced with $NaBH_4$ into the corresponding diol which was filtered over a silica plug and directly analyzed at the UPC^2.

UPC^2: IB-3, CO_2:MeCN, gradient [99:1 (0.5 min), 99:1 to 60:40 (over 4 min), 60:40 (5.5 min)], 120 bar, 40 °C, 2.0 mL min^{-1}; t_{minor} = 4.45 min; t_{major} = 4.83 min, 95.5:4.5 e.r.

$[\alpha]_D^{298\,K}$ (**3g**) = -42.8 (c 0.77, CH_2Cl_2) for 95.5:4.5 e.r.

(2S,3R)-3-(3,4-Dichlorophenyl)-4-(3,5-dimethyl-1H-pyrazole-1-carbonyl)-1-phenylbicyclo[2.1.1]hexane-2-carbaldehyde, 3h

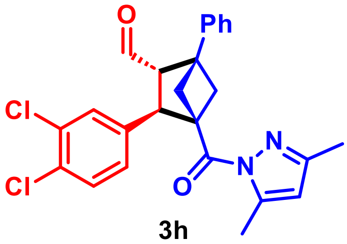

3h

Performed on 0.050 mmol scale following GP1. Full conversion was achieved in 18 h. Purified by FC (silica gel, 10:1 pentane:Et_2O) to obtain **3h** (49% yield, 11.1 mg, 0.024 mmol, >20:1 d.r., >20:1 r.r., 93.5:6.5 e.r.) as a yellow oil.

^{1}H NMR ($CDCl_3$, 400 MHz): δ [ppm] δ 9.74 (d, J = 2.1 Hz, 1H), 7.37 (tt, J = 6.9, 1.0 Hz, 2H), 7.33 – 7.23 (m, 6H), 7.04 (dd, J = 8.3, 2.2 Hz, 1H), 5.95 (d, J = 1.1 Hz, 1H), 4.77 (dd, J = 5.0, 1.7 Hz, 1H), 3.29 (dt, J = 4.5, 2.1 Hz, 1H), 2.81 (dd, J = 9.4, 7.5 Hz, 1H), 2.60 (dd, J = 9.4, 7.8 Hz, 1H), 2.51 – 2.43 (m, 4H), 2.29 (dd, J = 7.8, 1.8 Hz, 1H), 2.25 (s, 3H).

^{13}C NMR ($CDCl_3$, 100 MHz): δ [ppm] 202.3, 172.0, 153.0, 144.3, 140.9, 139.4, 132.8, 131.1, 130.6, 129.8, 128.9 (2C), 127.5, 126.9, 126.1 (2C), 111.1, 63.7, 55.4, 52.6, 49.4, 46.9, 44.9, 14.4, 14.2.

HRMS (ESI+): m/z calcd. for $C_{25}H_{23}Cl_2N_2O_2$ $[M+H]^+$: 453.1131; found: 453.1129.

UPC^2: IB-3, CO_2:MeCN, gradient [99:1 (0.5 min), 99:1 to 60:40 (over 4 min), 60:40 (5.5 min)], 120 bar, 40 °C, 2.0 mL min^{-1}; t_{minor} = 3.62 min; t_{minor} = 3.71 min, 93.5:6.5 e.r.

$[\alpha]_D^{298\,K}$ = -15.6 (c 0.90, CH_2Cl_2) for 93.5:6.5 e.r.

(2*S*,3*S*)-3-(2-Chlorophenyl)-4-(3,5-dimethyl-1*H*-pyrazole-1-carbonyl)-1-phenylbicyclo[2.1.1]hexane-2-carbaldehyde, 3i

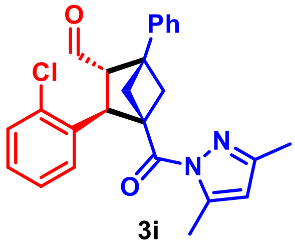

3i

Performed on 0.050 mmol scale following GP1. Full conversion was achieved in 18 h. Purified by FC (silica gel, 10:1 pentane:Et_2O) to obtain **3i** (60% yield, 12.6 mg, 0.030 mmol, >20:1 d.r., >20:1 r.r., 86:14 e.r.) as a yellow oil.

^{1}H NMR ($CDCl_3$, 400 MHz): δ [ppm] 9.92 (d, *J* = 3.5 Hz, 1H), 7.59 (dd, *J* = 7.8, 1.6 Hz, 1H), 7.31 (td, *J* = 7.6, 1.1 Hz, 3H), 7.26 – 7.12 (m, 5H), 5.88 (d, *J* = 1.1 Hz, 1H), 5.11 (dd, *J* = 4.7, 1.8 Hz, 1H), 3.15 – 3.05 (m, 2H), 2.72 (dd, *J* = 9.5, 7.6 Hz, 1H), 2.50 – 2.43 (m, 5H), 2.21 (s, 3H).

^{13}C NMR ($CDCl_3$, 100 MHz): δ [ppm] 202.8, 172.7, 152.6, 144.2, 139.6, 139.1, 134.8, 130.1, 128.7 (2C), 128.0, 127.5, 127.3, 127.0, 126.1 (2C), 111.0, 64.5, 54.8, 53.9, 49.0, 46.1 (2C), 14.5, 14.1.

HRMS (ESI+): *m/z* calcd. for $C_{25}H_{24}ClN_2O_2$ $[M+H]^+$: 419.1521; found: 419.1526.

UPC^2: IB-3, CO_2:MeCN, gradient [99:1 (0.5 min), 99:1 to 60:40 (over 4 min), 60:40 (5.5 min)], 120 bar, 40 °C, 2.0 mL min^{-1}; t_{minor} = 3.55 min; t_{minor} = 3.91 min, 86:14 e.r.

$[\alpha]_D^{298\,K}$ = -44.2 (*c* 0.85, CH_2Cl_2) for 86:14 e.r.

(2*S*,3*S*)-4-(3,5-Dimethyl-1*H*-pyrazole-1-carbonyl)-3-(furan-2-yl)-1-phenylbicyclo[2.1.1]hexane-2-carbaldehyde, 3j

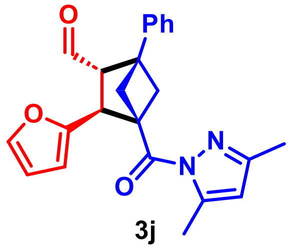

3j

Performed on 0.050 mmol scale following GP1. Full conversion was achieved in 18 h. Purified by FC (silica gel, 20:1 pentane:Et_2O) to obtain **3j** (61% yield, 11.5 mg, 0.031 mmol, >20:1 d.r., >20:1 r.r., 93:7 e.r.) as a colorless oil.

^{1}H NMR ($CDCl_3$, 400 MHz): δ [ppm] 9.74 (d, *J* = 2.2 Hz, 1H), 7.39 – 7.32 (m, 2H), 7.30 – 7.24 (m, 4H), 6.25 (dd, *J* = 3.2, 1.9 Hz, 1H), 6.04 (dt, *J* = 3.3, 0.9 Hz, 1H), 5.94 (d, *J* = 1.1 Hz, 1H), 4.76 (dd, *J* = 4.6, 2.6 Hz, 1H), 3.47 (dt, *J* = 4.5, 2.1 Hz, 1H), 2.81 (dd, *J* = 9.5, 7.5 Hz, 1H), 2.63 (dd, *J* = 9.5, 7.5 Hz, 1H), 2.49 (d, *J* = 1.1 Hz, 3H), 2.44 (dd, *J* = 7.5, 2.0 Hz, 1H), 2.25 (s, 3H), 2.19 (dd, *J* = 7.5, 1.8 Hz, 1H).

^{13}C NMR ($CDCl_3$, 100 MHz): δ [ppm] 202.2, 171.9, 154.1, 152.5, 144.1, 142.1, 139.7, 128.8 (2C), 127.3, 126.2 (2C), 110.8, 110.3, 106.7, 61.8, 55.1, 52.5, 46.0, 45.3, 44.5, 14.4, 14.2.

HRMS (ESI+): *m/z* calcd. for $C_{23}H_{23}N_2O_3$ $[M+H]^+$: 375.1703; found: 375.1701.

UPC^2: IC-3, CO_2:MeCN, gradient [99:1 (0.5 min), 99:1 to 60:40 (over 4 min), 60:40 (1.5 min)], 120 bar, 40 °C, 3.0 mL min^{-1}; t_{major} = 3.03 min; t_{minor} = 3.15 min, 93:7 e.r.

$[\alpha]_D^{298\,K}$ = -32.9 (*c* 0.5, CH_2Cl_2) for 93:7 e.r.

(2*S*,3*R*)-3-(4-Bromophenyl)-4-(1-methyl-1*H*-imidazole-2-carbonyl)-1-phenylbicyclo[2.1.1]hexane-2-carbaldehyde, 3k

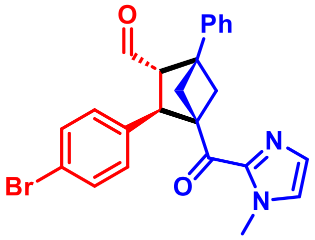

3k

Performed on 0.050 mmol scale following GP1. Full conversion was achieved in 18 h. Purified by FC (silica gel, 3:2 pentane:Et_2O) to obtain **3k** (49% yield, 10.9 mg, 0.025 mmol, >20:1 d.r., >20:1 r.r., 92:8 e.r.) as a colorless oil.

^{1}H NMR ($CDCl_3$, 400 MHz): δ [ppm] 9.80 (d, *J* = 2.6 Hz, 1H), 7.35 (ddd, *J* = 7.7, 5.2, 2.2 Hz, 4H), 7.28 – 7.22 (m, 3H), 7.15 (d, *J* = 0.9 Hz, 1H), 7.11 (m, 2H), 6.99 (d, *J* = 0.9 Hz, 1H), 4.89 (dd, *J* = 4.8, 1.7 Hz, 1H), 3.92 (s, 3H), 3.22 (dt, *J* = 4.8, 2.3 Hz, 1H), 2.92 – 2.88 (dd, *J* = 9.4, 7.3 Hz, 1H), 2.61 – 2.57 (dd, *J* = 9.4, 7.7 Hz, 1H), 2.45 – 2.37 (ddd, *J* = 21.3, 7.5, 2.0 Hz, 2H).

^{13}C NMR ($CDCl_3$, 100 MHz): δ [ppm] 202.8, 191.5, 142.1, 140.4, 139.8, 131.8 (2C), 129.7, 129.4 (2C), 128.9 (2C), 127.3, 126.9, 126.1 (2C), 120.6, 65.1, 58.5, 52.9, 50.4, 47.4, 44.3, 36.3.

HRMS (ESI+): *m/z* calcd. for $C_{24}H_{22}{}^{79}BrN_2O_2$ $[M+H]^+$: 449.0859; found: 449.0856. *m/z* calcd. for $C_{24}H_{22}{}^{81}BrN_2O_2$ $[M+H]^+$: 451.0839; found: 451.0838.

UPC^2: IC-3, CO_2/MeCN, gradient [99:1 (0.5 min), 99:1 to 60:40 (over 4 min), 60:40 (1.5 min)], 120 bar, 40 °C, 2.0 mL min^{-1}; t_{major} = 4.35 min; t_{minor} = 4.14 min, 92:8 e.r.

$[\alpha]_D^{298\,K}$ = -39.5 (*c* 0.48, CH_2Cl_2) for 92:8 e.r.

Ethyl (*E*)-3-((2*S*,3*R*)-3-(4-bromophenyl)-4-(methoxy(methyl)carbamoyl)-1-phenylbicyclo[2.1.1]hexan-2-yl)acrylate, 3l

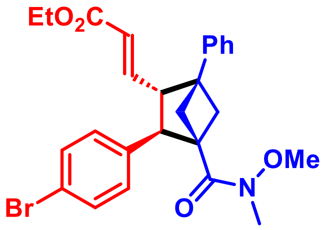

3l

Performed on 0.050 mmol scale following GP1. Full conversion was achieved in 18 h after which Wittig reagent (5.0 equiv.) was added and the mixture was stirred for a further 6 h. Purified by FC (silica gel, 3:2 Et_2O:pentane) to obtain **3l** (51% yield, 12.7 mg, 0.026 mmol, >20:1 d.r., >20:1 r.r., 96.5:3.5 e.r.) as a colorless oil.

^{1}H NMR ($CDCl_3$, 400 MHz): δ [ppm] 7.44 (m, 2H), 7.32 – 7.28 (m, 2H), 7.21 (m, 3H), 7.12 (m, 2H), 7.04 – 6.95 (dd, *J* = 15.6, 9.4 Hz, 1H), 5.72 – 5.68 (dd, *J* = 15.5, 0.9 Hz, 1H), 4.17 – 4.08 (m, 2H), 3.80 – 3.69 (m, 1H), 3.59 (s, 3H), 3.12 (s, 2H), 3.10 – 3.06 (dt, *J* = 9.3, 2.7 Hz, 1H), 2.48 (m, 2H), 2.31 – 2.20 (m, 2H), 1.26 – 1.22 (m, 3H).

^{13}C NMR ($CDCl_3$, 100 MHz): δ [ppm] 166.4, 148.6, 148.5, 140.3, 139.9, 131.9 (2C), 129.3 (2C), 128.5 (2C), 126.9, 126.1 (2C), 123.3, 120.9, 61.7, 60.5, 56.3, 54.3, 54.0, 53.4, 45.0, 44.4, 32.7, 14.3.

HRMS (ESI+): *m/z* calcd. for $C_{26}H_{29}{}^{79}BrNO_4$ $[M+H]^+$: 498.1274; found: 498.1275. *m/z* calcd. for $C_{26}H_{29}{}^{81}BrNO_4$ $[M+H]^+$: 500.1254; found: 500.1258.

UPC^2: IB-3, CO_2/iPrOH, gradient [99:1 (0.5 min), 99:1 to 60:40 (over 4 min), 60:40 (1.5 min)], 120 bar, 40 °C, 2.0 mL min^{-1}; t_{major} = 3.88 min; t_{minor} = 3.79 min, 96.5:3.5 e.r.

$[\alpha]_D^{298\,K}$ = -49.0 (*c* 0.49, CH_2Cl_2) for 96.5:3.5 e.r.

(2*S*,3*S*)-3-((*E*)-4-Bromostyryl)-4-(3,5-dimethyl-1*H*-pyrazole-1-carbonyl)-1-phenylbicyclo[2.1.1]hexane-2-carbaldehyde, 3m

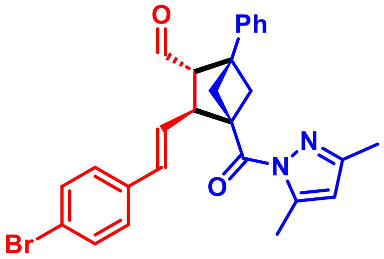

3m

Performed on 0.050 mmol scale following GP1. Full conversion was achieved in 18 h. Purified by FC (silica gel, 20:2:1 pentane:CH_2Cl_2:Et_2O) to obtain **3m** (55% yield, 13.5 mg, 0.028 mmol, >20:1 d.r., >20:1 r.r., 94:6 e.r.) as a yellow oil.

^{1}H NMR ($CDCl_3$, 400 MHz): δ [ppm] 9.73 (d, *J* = 2.7 Hz, 1H), 7.41 – 7.32 (m, 4H), 7.29 – 7.26 (m, 1H), 7.26 – 7.22 (m, 2H), 7.17 – 7.12 (m, 2H), 6.32 (d, *J* = 2.7 Hz, 1H), 6.31 (s, 1H), 5.93 (d, *J* = 1.1 Hz, 1H), 4.13 (tt, *J* = 4.3, 1.9 Hz, 1H), 3.08 (dt, *J* = 4.5, 2.4 Hz, 1H), 2.76 (dd, *J* = 9.4, 7.6 Hz, 1H), 2.54 (dd, *J* = 9.4, 7.7 Hz, 1H), 2.47 (d, *J* = 1.0 Hz, 3H), 2.42 (dd, *J* = 7.6, 2.0 Hz, 1H), 2.27 (s, 3H), 2.23 (dd, *J* = 7.6, 1.8 Hz, 1H).

^{13}C NMR ($CDCl_3$, 100 MHz): δ [ppm] 202.5, 172.2, 152.6, 144.2, 139.7, 135.9, 131.7 (2C), 131.6, 129.4, 128.8 (2C), 128.0 (2C), 127.3, 126.2 (2C), 121.4, 110.9, 63.7, 55.4, 52.7, 49.8, 45.7, 45.5, 14.4, 14.2.

HRMS (ESI+): *m/z* calcd. for $C_{27}H_{26}{}^{79}BrN_2O_2$ $[M+H]^+$: 489.1173; found: 489.1169. *m/z* calcd. for $C_{27}H_{26}{}^{81}BrN_2O_2$ $[M+H]^+$: 491.1152; found: 491.1151.

UPC^2: IB-3, CO_2:MeCN, gradient [99:1 (0.5 min), 99:1 to 60:40 (over 4 min), 60:40 (1.5 min)], 120 bar, 40 °C, 3.0 mL min^{-1}; t_{major} = 4.26 min; t_{minor} = 4.18 min, 94:6 e.r.

$[\alpha]_D^{298\,K}$ = +26.9 (*c* 1.0, CH_2Cl_2) for 94:6 e.r.

Ethyl (2*S*,3*S*)-1-Benzoyl-3-formyl-4-phenylbicyclo[2.1.1]hexane-2-carboxylate, 5a

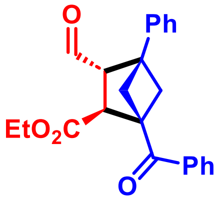

5a

Performed on 0.050 mmol scale following GP2. Full conversion was achieved in 18 h. Purified by FC (silica gel, 9:1 pentane:Et_2O) to obtain **5a** (69% yield, 12.5 mg, 0.034 mmol, >20:1 d.r., >20:1 r.r., 96:4 e.r.) as a yellow oil.

^{1}H NMR ($CDCl_3$, 400 MHz): δ [ppm] ^{1}H NMR (400 MHz, $CDCl_3$) δ 9.66 (d, *J* = 0.9 Hz, 1H), 7.94 – 7.87 (m, 2H), 7.61 – 7.54 (m, 1H), 7.53 – 7.45 (m, 2H), 7.42 – 7.34 (m, 2H), 7.32 – 7.27 (m, 1H), 7.26 – 7.23 (m, 2H), 4.09 (dd, *J* = 4.1, 2.0 Hz, 1H), 3.97 (q, *J* = 7.1 Hz, 2H), 3.84 (ddd, *J* = 4.1, 2.0, 0.9 Hz, 1H), 2.58 – 2.48 (m, 2H), 2.37 – 2.26 (m, 2H), 1.01 (t, *J* = 7.1 Hz, 3H).

^{13}C NMR ($CDCl_3$, 100 MHz): δ [ppm] 201.9, 199.0, 171.9, 139.0, 136.5, 133.1, 129.0 (2C), 128.8 (2C), 128.5 (2C), 127.6, 125.9 (2C), 61.5, 59.8, 56.9, 53.1, 48.9, 46.2, 45.2, 13.9.

HRMS (ESI+): *m/z* calcd. for $C_{23}H_{23}O_4$ $[M+H]^+$: 363.1591; found: 363.1590.

UPC^2: IB-3, CO_2:MeCN, gradient [99:1 (0.5 min), 99:1 to 60:40 (over 4 min), 60:40 (5.5 min)], 120 bar, 40 °C, 2.0 mL min^{-1}; t_{major} = 3.16 min; t_{minor} = 3.23 min, 96:4 e.r.

$[\alpha]_D^{298\,K}$ = -15,2 (*c* 1.07, CH_2Cl_2) for 96:4 e.r.

Isopropyl (2*S*,3*S*)-1-benzoyl-3-formyl-4-phenylbicyclo[2.1.1]hexane-2-carboxylate, 5b

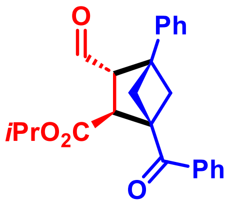

5b

Performed on 0.10 mmol scale following GP3. 40% conversion was achieved in 18 h. Purified by FC (silica gel, 10:1 pentane:Et_2O) to obtain **5b** (35% yield, 13.1 mg, 0.0348 mmol, >20:1 d.r., >20:1 r.r., 98:2 e.r.) as a yellow solid.

^{1}H NMR (CD_2Cl_2, 400 MHz): δ [ppm] 9.65 (d, *J* = 1.0 Hz, 1H), 7.94 – 7.87 (m, 2H), 7.65 – 7.55 (m, 1H), 7.50 (tt, *J* = 7.0, 1.4 Hz, 2H), 7.38 (tt, *J* = 7.0, 1.0 Hz, 2H), 7.33 – 7.23 (m, 3H), 4.84 (hept, *J* = 6.2 Hz, 1H), 4.00 (dd, *J* = 4.1, 2.1 Hz, 1H), 3.78 (ddd, *J* = 4.2, 2.0, 1.1 Hz, 1H), 2.56 – 2.41 (m, 2H), 2.35 (dd, *J* = 6.9, 2.0 Hz, 1 H), 2.25 (dd, *J* = 7.0, 2.0 Hz, 1H), 1.03 (d, *J* = 6.2 Hz, 3H), 0.98 (d, *J* = 6.3 Hz, 3H).

^{13}C NMR (CD_2Cl_2, 100 MHz): δ [ppm] 202.1, 198.9, 171.5, 139.5, 136.9, 133.2, 129.1 (2C), 129.0 (2C), 128.7 (2C), 127.7, 126.3 (2C), 69.5, 60.1, 57.1, 53.1, 49.4, 46.3, 45.6, 21.7, 21.5.

HRMS (ESI+): *m/z* calcd. for $C_{24}H_{25}O_4$ $[M+H]^+$: 377.1747; found: 377.1747.

UPC^2: IB-3, CO_2/CH_2Cl_2, gradient [99:1 (0.5 min), 99:1 to 60:40 (over 4 min), 60:40 (5.5 min)], 120 bar, 40 °C, 2.0 mL min^{-1}; t_{major} = 3.34 min; t_{minor} = 3.27 min, 98:2 e.r.

$[\alpha]_D^{298\,K}$ = –25.9 (*c* 1.0, CH_2Cl_2) for 98:2 e.r.

tert-Butyl (2*S*,3*S*)-1-benzoyl-3-formyl-4-phenylbicyclo[2.1.1]hexane-2-carboxylate, 5c

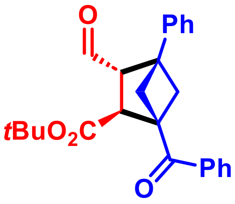

5c

Performed on 0.10 mmol scale following GP3. 40% conversion was achieved in 18 h. Purified by FC (silica gel, 10:1 pentane:Et_2O) to obtain **5c** (37% yield, 14.3 mg, 0.0366 mmol, >20:1 d.r., >20:1 r.r., 98.5:1.5 e.r.) as a yellow solid.

^{1}H NMR (CD_2Cl_2, 400 MHz): δ [ppm] 9.65 (d, *J* = 1.0 Hz, 1H), 7.98 – 7.89 (m, 2H), 7.66 – 7.55 (m, 1H), 7.51 (ddt, *J* = 8.2, 6.6, 1.2 Hz, 2H), 7.37 (tt, *J* = 7.1, 1.0 Hz, 2H), 7.33 – 7.23 (m, 3H), 4.00 (dd, *J* = 4.0, 2.1 Hz, 1H), 3.78 (ddd, *J* = 4.1, 2.0, 1.0 Hz, 1H), 2.52 (dd, *J* = 9.5, 7.2 Hz, 1H), 2.44 (dd, *J* = 9.5, 7.4 Hz, 1H), 2.34 (dd, *J* = 7.2, 2.0, Hz, 1H), 2.21 (dd, *J* = 7.4, 2.1 Hz, 1H), 1.23 (s, 9H).

^{13}C NMR (CD_2Cl_2, 100 MHz): δ [ppm] 202.3, 198.7, 171.0, 139.7, 136.6, 133.2, 129.0 (2C), 129.0 (2C), 128.8 (2C), 127.6, 126.3 (2C), 82.3, 60.0, 57.2, 53.0, 50.2, 46.0, 45.8, 27.8 (3C).

HRMS (ESI+): *m/z* calcd. for $C_{25}H_{27}O_4$ $[M+H]^+$: 391.1904; found: 391.1904.

UPC^2: IB-3, CO_2/CH_2Cl_2, gradient [99:1 (0.5 min), 99:1 to 60:40 (over 4 min), 60:40 (5.5 min)], 120 bar, 40 °C, 2.0 mL min^{-1}; t_{major} = 3.23 min; t_{minor} = 3.13 min, 98.5:1.5 e.r.

$[\alpha]_D^{298\,K}$ = –32.6 (*c* 1.0, CH_2Cl_2) for 98.5:1.5 e.r.

Benzyl (2*S*,3*S*)-1-benzoyl-3-formyl-4-phenylbicyclo[2.1.1]hexane-2-carboxylate, 5d

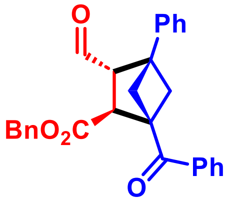

5d

Performed on 0.10 mmol scale following GP3. 40% conversion was achieved in 18 h. Purified by FC (silica gel, 10:2 pentane:Et_2O) to obtain **5d** (32% yield, 13.6mg, 0.0320 mmol, >20:1 d.r., >20:1 r.r., 97.5:2.5 e.r.) as a yellow solid.

^{1}H NMR (CD_2Cl_2, 400 MHz): δ [ppm] 9.64 (d, *J* = 1.0 Hz, 1H), 7.84 – 7.76 (m, 2H), 7.61 – 7.52 (m, 1H), 7.48 – 7.34 (m, 4H), 7.30 – 7.23 (m, 5H), 7.11 – 7.06 (m, 2H), 4.99 (d, *J* = 12.3 Hz, 1H), 4.88 (d, *J* = 12.3, 1H), 4.10 (dd, *J* =) 4.2, 2.0 Hz, 1H), 3.80 (ddd, *J* = 4.1, 2.0, 1.0 Hz, 1H), 2.58 – 2.46 (m, 2H), 2.41 – 2.33 (m, 1H), 2.32 – 2.22 (m, 1H).

^{13}C NMR (CD_2Cl_2, 100 MHz): δ [ppm] 201.8, 199.0, 172.0, 139.4, 136.8, 135.6, 133.2, 129.1 (2C), 129.0 (2C), 128.7 (2C), 128.7 (2C), 128.5 (2C), 128.5 (2C), 127.8, 67.4, 60.2, 57.1, 53.2, 49.4, 46.5, 45.5.

HRMS (ESI+): *m/z* calcd. for $C_{28}H_{25}O_4$ $[M+H]^+$: 425.1747; found: 425.1750.

UPC^2: IB-3, CO_2/CH_2Cl_2, gradient [99:1 (0.5 min), 99:1 to 60:40 (over 4 min), 60:40 (5.5 min)], 120 bar, 40 °C, 2.0 mL min^{-1}; t_{major} = 4.04 min; t_{minor} = 3.96 min, 97.5:2.5 e.r.

$[\alpha]_D^{298\,K}$ = –18.7 (*c* 1.0, CH_2Cl_2) for 97.5:2.5 e.r.

Ethyl (2*S*,3*S*)-3-formyl-1-(4-methoxybenzoyl)-4-phenylbicyclo[2.1.1]hexane-2-carboxylate, 5e

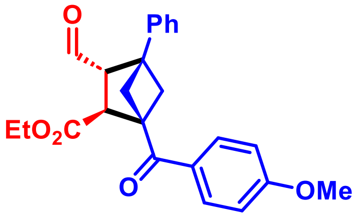

5e

Performed on 0.050 mmol scale following GP2. Full conversion was achieved in 18 h. Purified by FC (silica gel, 2:1 pentane:Et_2O) to obtain **5e** (68% yield, 13.4 mg, 0.034 mmol, >20:1 d.r., >20:1 r.r., 98.5:1.5 e.r.) as a colorless oil.

^{1}H NMR ($CDCl_3$, 400 MHz): δ [ppm] 9.65 (s, 1H), 7.93 – 7.89 (m, 2H), 7.39 – 7.35 (tt, *J* = 7.9, 1.4 Hz, 2H), 7.29 (m, 1H), 7.27 – 7.23 (m, 2H), 6.98 – 6.94 (m, 2H), 4.06 (dd, *J* = 4.1, 2.0 Hz, 1H), 4.01 – 3.93 (qd, *J* = 7.2, 2.8 Hz, 2H), 3.89 (s, 3H), 3.87 – 3.81 (m, 1H), 2.58 – 2.47 (m, 2H), 2.32 – 2.24 (ddd, *J* = 23.9, 7.3, 2.0 Hz, 2H), 1.03 – 0.99 (t, *J* = 7.1 Hz, 3H).

^{13}C NMR ($CDCl_3$, 100 MHz): δ [ppm] 202.1, 197.3, 172.0, 163.5, 139.1, 130.9 (2C), 129.4, 128.9 (2C), 127.6, 126.0 (2C), 114.0 (2C), 61.4, 59.8, 56.8, 55.6, 52.9, 49.0, 46.2, 45.2, 13.9.

HRMS (ESI+): *m/z* calcd. for $C_{24}H_{25}O_5$ $[M+H]^+$: 393.1697; found: 393.1688.

UPC^2: IB-3, CO_2/MeCN, gradient [99:1 (0.5 min), 99:1 to 60:40 (over 4 min), 60:40 (1.5 min)], 120 bar, 40 °C, 2.0 mL min^{-1}; t_{major} = 3.70 min; t_{minor} = 3.63 min, 98.5:1.5 e.r.

$[\alpha]_D^{298\,K}$ = -15.2 (*c* 0.46, CH_2Cl_2) for 98.5:1.5 e.r.

Ethyl (2*S*,3*S*)-3-formyl-4-phenyl-1-(4-(trifluoromethyl)benzoyl)bicyclo[2.1.1]hexane-2-carboxylate, 5f

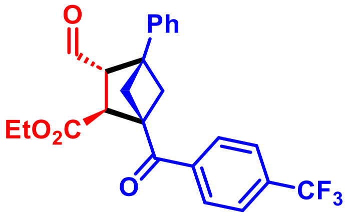

5f

Performed on 0.050 mmol scale following GP2. Full conversion was achieved in 18 h. Purified by FC (silica gel, 5:1 pentane:Et_2O) to obtain **5f** (65% yield, 14.0 mg, 0.033 mmol, >20:1 d.r., >20:1 r.r., 98:2 e.r.) as a white solid.

^{1}H NMR ($CDCl_3$, 400 MHz): δ [ppm] 9.66 (s, 1H), 8.00 (d, *J* = 8.1 Hz, 2H), 7.76 (d, *J* = 8.2 Hz, 2H), 7.40 – 7.36 (m, 2H), 7.33 – 7.28 (m, 1H), 7.25 – 7.21 (m, 2H), 4.10 (dd, *J* = 4.0, 2.1 Hz, 1H), 4.06 – 3.95 (qd, *J* = 7.2, 2.8 Hz, 2H), 3.84 (m, 1H), 2.54 – 2.47 (m, 2H), 2.35 – 2.29 (m, 2H), 1.06 – 1.03 (t, *J* = 7.1 Hz, 3H).

^{13}C NMR ($CDCl_3$, 100 MHz): δ [ppm] 201.7, 198.3, 171.7, 139.3, 138.7, 134.3 (d, *J* = 33 Hz), 129.0 (2C), 128.7 (2C), 127.8, 125.9 (4C), 123.7 (d, *J* = 273 Hz), 61.6, 59.8, 56.7, 53.3, 48.8, 46.1, 45.1, 14.0.

^{19}F NMR ($CDCl_3$, 376 MHz): δ [ppm] – 63.12 (s).

HRMS (ESI+): *m/z* calcd. for $C_{24}H_{22}F_3O_4$ $[M+H]^+$: 431.1465; found: 431.1457.

UPC^2: IB-3, CO_2/MeCN, gradient [99:1 (0.5 min), 99:1 to 60:40 (over 4 min), 60:40 (1.5 min)], 120 bar, 40 °C, 2.0 mL min^{-1}; t_{major} = 2.78 min; t_{minor} = 2.64 min, 98:2 e.r.

$[\alpha]_D^{298\,K}$ = -12.9 (*c* 0.45, CH_2Cl_2) for 98:2 e.r.

Ethyl (2*S*,3*S*)-3-formyl-1-pentanoyl-4-phenylbicyclo[2.1.1]hexane-2-carboxylate, 5g

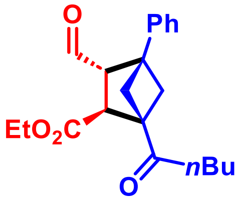

5g

Performed on 0.050 mmol scale following GP2. Full conversion was achieved in 18 h. Purified by FC (silica gel, 4:1 pentane:Et_2O) to obtain **5g** (53% yield, 9.1 mg, 0.027 mmol, >20:1 d.r., >20:1 r.r., 97.5:2.5 e.r.) as a colorless oil.

^{1}H NMR ($CDCl_3$, 400 MHz): δ [ppm] 9.59 (s, 1H), 7.37 – 7.33 (t, *J* = 7.4 Hz, 1H), 7.29 (d, *J* = 7.1 Hz, 1H), 7.20 (d, *J* = 7.4 Hz, 2H), 4.20 – 4.09 (qt, *J* = 6.8, 3.4 Hz, 2H), 3.89 (dd, *J* = 4.3, 2.0 Hz, 1H), 3.61 (d, *J* = 4.1 Hz, 1H), 2.66 – 2.47 (m, 2H), 2.33 – 2.27 (dd, *J* = 9.5, 7.4 Hz, 1H), 2.19 – 2.11 (m, 2H), 2.11 – 2.06 (dd, *J* = 7.0, 2.0 Hz, 1H), 1.63 – 1.55 (m, 2H), 1.38 – 1.29 (m, 2H), 1.26 – 1.23 (t, *J* = 7.1 Hz, 1H), 0.94 – 0.90 (t, *J* = 7.3 Hz, 3H).

^{13}C NMR ($CDCl_3$, 100 MHz): δ [ppm] 208.6, 201.5, 172.2, 139.0, 128.9 (2C), 127.6, 125.9 (2C), 61.5, 59.9, 57.3, 53.0, 47.2, 44.6, 43.7, 38.5, 25.4, 22.5, 14.2, 14.1.

HRMS (ESI+): *m/z* calcd. for $C_{21}H_{27}O_4$ $[M+H]^+$: 343.1904; found: 343.1901.

UPC^2: IB-3, CO_2/MeOH, gradient [99:1 (0.5 min), 99:1 to 60:40 (over 4 min), 60:40 (1.5 min)], 120 bar, 40 °C, 2.0 mL min^{-1}; t_{major} = 2.28 min; t_{minor} = 2.22 min, 97.5:2.5 e.r.

$[\alpha]_D^{298\,K}$ = -3.3 (*c* 0.34, CH_2Cl_2) for 97.5:2.5 e.r.

2-Ethyl 1-methyl (2*S*,3*S*)-3-(hydroxymethyl)-4-phenylbicyclo[2.1.1]hexane-1,2-dicarboxylate, 5h

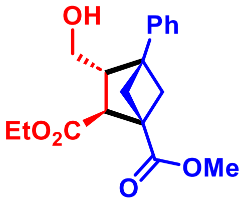

5h

Performed on 0.050 mmol scale following GP2. Full conversion was achieved in 18 h after which $NaBH_4$ (4.0 equiv.) and MeOH (0.2 mL) was added and stirred for a further 1 h. Purified by FC (silica gel, 1:1 pentane:Et_2O) to obtain **5h** (68% yield, 10.8 mg, 0.034 mmol, >20:1 d.r., >20:1 r.r., 96.5:3.5 e.r.) as a colorless oil.

^{1}H NMR ($CDCl_3$, 400 MHz): δ [ppm] 7.34 – 7.30 (m, 2H), 7.26 – 7.22 (m, 1H), 7.14 (m, 2H), 4.23 – 4.18 (q, *J* = 7.1 Hz, 2H), 3.72 (s, 3H), 3.71 – 3.57 (m, 2H), 3.13 (dd, *J* = 4.8, 2.1 Hz, 1H), 2.66 – 2.61 (dtd, *J* = 7.5, 5.7, 1.8 Hz, 1H), 2.35 –2.21 (ddd, *J* = 36.4, 9.4, 6.9 Hz, 1H), 2.18 – 2.14 (ddd, *J* = 8.5, 6.9, 2.0 Hz, 2H), 1.30 – 1.26 (t, *J* = 7.1 Hz, 3H).

^{13}C NMR ($CDCl_3$, 100 MHz): δ [ppm] 173.4, 171.9, 140.4, 128.8 (2C), 127.1, 125.7 (2C), 63.9, 61.2, 52.6, 52.0, 51.0, 50.5, 45.5, 43.1, 14.3.

HRMS (ESI+): *m/z* calcd. for $C_{18}H_{23}O_5$ $[M+H]^+$: 319.1540; found: 319.1592.

UPC²: IB-3, CO_2/iPrOH, gradient [99:1 (0.5 min), 99:1 to 60:40 (over 4 min), 60:40 (1.5 min)], 120 bar, 40 °C, 2.0 mL min^{-1}; t_{major} = 3.23 min; t_{minor} = 3.09 min, 96.5:3.5 e.r.

$[\alpha]_D^{298\,K}$ = -22.9 (*c* 0.45, CH_2Cl_2) for 96.5:3.5 e.r.

Ethyl (2*S*,3*S*)-3-((*E*)-3-ethoxy-3-oxoprop-1-en-1-yl)-1-(methoxy(methyl)carbamoyl)-4-phenylbicyclo[2.1.1]hexane-2-carboxylate, 5i

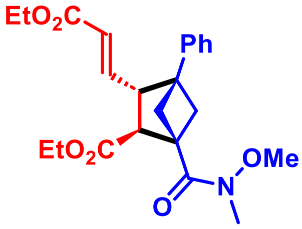

5i

Performed on 0.050 mmol scale following GP2. Full conversion was achieved in 18 h after which the mixture was reduced approximately by half and Wittig reagent (5.0 equiv.) was added and the mixture was stirred for a further 6 h. Purified by FC (silica gel, 2:1 Et_2O:pentane) to obtain **5i** (62% yield, 12.8 mg, 0.031 mmol, >20:1 d.r., >20:1 r.r., 98.5:1.5 e.r.) as a colorless oil.

^{1}H NMR ($CDCl_3$, 400 MHz): δ [ppm] 7.32 – 7.27 (m, 2H), 7.24 – 7.19 (m, 1H), 7.13 (m, 2H), 6.89 – 6.82 (dd, *J* = 15.6, 8.5 Hz, 1H), 5.81 – 5.76 (dd, *J* = 15.6, 1.1 Hz, 1H), 4.18 – 4.09 (m, 4H), 3.70 (s, 3H), 3.52 – 3.48 (m, 1H), 3.30 (dd, *J* = 4.3, 1.8 Hz, 1H), 3.22 (s, 3H), 2.40 – 2.29 (ddd, *J* = 30.9, 9.3, 7.2 Hz, 1H), 2.26 (m, 1H), 2.11 (dd, *J* = 7.3, 1.8 Hz, 1H), 1.24 (q, *J* = 7.2 Hz, 6H).

^{13}C NMR ($CDCl_3$, 100 MHz): δ [ppm] 172.2, 166.3, 147.5, 139.9, 128.5 (2C), 127.0, 126.3 (2C), 123.9, 61.6, 61.3, 60.5, 53.6, 53.1, 52.7, 50.8, 44.9, 44.4, 32.9, 14.4, 14.3.

HRMS (ESI+): *m/z* calcd. for $C_{23}H_{30}NO_6$ $[M+H]^+$: 416.2068; found: 416.2072.

UPC²: IB-3, CO_2/MeCN, gradient [99:1 (0.5 min), 99:1 to 60:40 (over 4 min), 60:40 (1.5 min)], 120 bar, 40 °C, 2.0 mL min^{-1}; t_{major} = 3.10 min; t_{minor} = 3.21 min, 98.5:1.5 e.r.

$[\alpha]_D^{298\,K}$ = -13.5 (*c* 0.46, CH_2Cl_2) for 98.5:1.5 e.r.

Ethyl (2*S*,3*S*)-3-formyl-1-(1-methyl-1*H*-imidazole-2-carbonyl)-4-phenylbicyclo[2.1.1]hexane-2-carboxylate, 5j

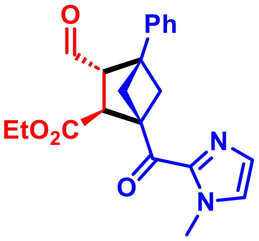

5j

Performed on 0.050 mmol scale following GP2. Full conversion was achieved in 18 h. Purified by FC (silica gel, 3:2 pentane:Et_2O) to obtain **5j** (64% yield, 11.7 mg, 0.032 mmol, >20:1 d.r., >20:1 r.r., 95:5 e.r.) as a colorless oil.

^{1}H NMR ($CDCl_3$, 400 MHz): δ [ppm] 9.68 (d, *J* = 1.4 Hz, 1H), 7.37 – 7.33 (m, 2H), 7.28 (m, 1H), 7.22 (m, 2H), 7.16 (s, 1H), 7.01 (s, 1H), 4.51 (dd, *J* = 4.5, 1.5 Hz, 1H), 4.08 – 4.03 (td, *J* = 7.2, 1.1 Hz, 2H), 4.01 (s, 3H), 3.63 (dt, *J* = 3.9, 1.8 Hz, 1H), 2.65 – 2.61 (td, *J* = 7.7, 1.7 Hz, 1H), 2.39 – 2.33 (m, 3H), 1.11 – 1.07 (t, *J* = 7.1 Hz, 3H).

^{13}C NMR ($CDCl_3$, 100 MHz): δ [ppm] 201.7, 190.0, 172.5, 142.3, 139.6, 129.5, 128.8 (2C), 127.4, 126.5, 126.1 (2C), 61.1, 60.1, 56.7, 52.8, 49.8, 45.6, 45.3, 36.2, 14.1.

HRMS (ESI+): *m/z* calcd. for $C_{21}H_{23}N_2O_4$ $[M+H]^+$: 367.1652; found: 367.1655.

UPC^2: IC-3, CO_2/iPrOH, gradient [99:1 (0.5 min), 99:1 to 60:40 (over 4 min), 60:40 (1.5 min)], 120 bar, 40 °C, 2.0 mL min^{-1}; t_{major} = 4.29 min; t_{minor} = 3.10 min, 95:5 e.r.

$[\alpha]_D^{298\,K}$ = -24.2 (*c* 0.48, CH_2Cl_2) for 95:5 e.r.

Ethyl (2*S*,3*S*)-3-formyl-4-phenyl-1-(1*H*-pyrazole-1-carbonyl)bicyclo[2.1.1]hexane-2-carboxylate, 5k

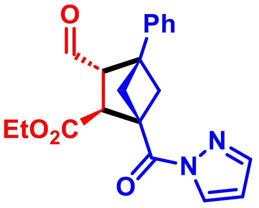

5k

Performed on 0.050 mmol scale following GP2. Full conversion was achieved in 18 h. Purified by FC (silica gel, 10:1 to 4:1 pentane:Et_2O) to obtain **5k** (67% yield, 11.8 mg, 0.034 mmol, >20:1 d.r., >20:1 r.r., 97:3 e.r.) as a colorless oil.

^{1}H NMR ($CDCl_3$, 400 MHz): δ [ppm] 9.65 (d, *J* = 1.1 Hz, 1H), 8.26 (dd, *J* = 2.9, 0.8 Hz, 1H), 7.75 (dd, *J* = 1.5, 0.7 Hz, 1H), 7.40 – 7.34 (m, 2H), 7.32 – 7.27 (m, 1H), 7.25 – 7.22 (m, 2H), 6.45 (dd, *J* = 2.8, 1.5 Hz, 1H), 4.51 (dd, *J* = 4.3, 2.0 Hz, 1H), 4.03 (qd, *J* = 7.1, 4.8 Hz, 2H), 3.73 (ddd, *J* = 4.4, 2.1, 1.2 Hz, 1H), 2.62 (dd, *J* = 9.3, 7.5 Hz, 1H), 2.45 – 2.36 (m, 2H), 2.32 (dd, *J* = 7.6, 2.0 Hz, 1H), 1.06 (t, *J* = 7.2 Hz, 3H).

^{13}C NMR ($CDCl_3$, 100 MHz): δ [ppm] 201.4, 171.9, 169.9, 144.4, 139.0, 128.9 (2C), 128.6, 127.6, 126.0 (2C), 109.4, 61.4, 59.4, 53.0, 52.5, 49.5, 45.9, 45.5, 13.9.

HRMS (ESI+): *m/z* calcd. for $C_{20}H_{21}N_2O_4$ $[M+H]^+$: 353.1496; found: 353.1488.

UPC^2: IB-3, CO_2:MeCN, gradient [99:1 (0.5 min), 99:1 to 60:40 (over 4 min), 60:40 (1.5 min)], 120 bar, 40 °C, 3.0 mL min^{-1}; t_{major} = 2.80 min; t_{minor} = 3.00 min, 97:3 e.r.

$[\alpha]_D^{298\,K}$ = -23.3 (*c* 0.83, CH_2Cl_2) for 97:3 e.r.

Ethyl (2*S*,3*S*)-1-(3,5-dimethyl-1*H*-pyrazole-1-carbonyl)-3-formyl-4-phenylbicyclo[2.1.1]hexane-2-carboxylate, 5l

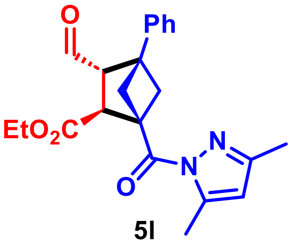

5l

Performed on 0.050 mmol scale following GP2. Full conversion was achieved in 18 h. Purified by FC (silica gel, 10:1 pentane:Et_2O) to obtain **5l** (75% yield, 14.3 mg, 0.038 mmol, >20:1 d.r., >20:1 r.r., 98:2 e.r.) as a yellow oil.

^{1}H NMR ($CDCl_3$, 400 MHz): δ 9.65 (d, J = 1.2 Hz, 1H), 7.39 – 7.33 (m, 2H), 7.32 – 7.21 (m, 3H), 5.95 (d, J = 1.1 Hz, 1H), 4.46 (dd, J = 4.3, 1.9 Hz, 1H), 4.04 (qd, J = 7.1, 2.8 Hz, 2H), 3.70 (ddd, J = 4.3, 2.1, 1.2 Hz, 1H), 2.60 (dd, J = 9.4, 7.5 Hz, 1H), 2.55 (d, J = 1.0 Hz, 3H), 2.41 – 2.33 (m, 2H), 2.25 (s, 4H), 1.10 (t, J = 7.1 Hz, 3H).

^{13}C NMR ($CDCl_3$, 100 MHz): δ [ppm] 201.7, 172.2, 171.4, 152.4, 144.0, 139.4, 128.9 (2C), 127.4, 126.1 (2C), 110.7, 61.3, 59.6, 53.6, 52.3, 49.4, 45.9, 45.7, 14.4, 14.1, 14.0.

HRMS (ESI+): *m/z* calcd. for $C_{22}H_{25}N_2O_4$ $[M+H]^+$: 381.1809; found: 381.1816.

UPC^2: IB-3, CO_2:MeCN IB, gradient [99:1 (0.5 min), 99:1 to 60:40 (over 4 min), 60:40 (5.5 min)], 120 bar, 40 °C, 2.0 mL min^{-1}; t_{major} = 2.71 min; t_{minor} = 2.84 min, 98:2 e.r.

$[\alpha]_D^{298\,K}$ = -25,5 (*c* 1.10, CH_2Cl_2) for 98:2 e.r.

Ethyl (2*S*,3*S*)-4-(4-Chlorophenyl)-1-(3,5-dimethyl-1*H*-pyrazole-1-carbonyl)-3-formylbicyclo[2.1.1]hexane-2-carboxylate, 5m

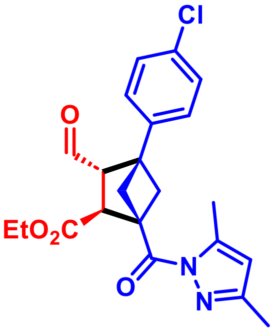

5m

Performed on 0.10 mmol scale following GP2. Full conversion was achieved in 24 h. Purified by FC (silica gel, 10:1 pentane:Et_2O) to obtain **5m** (50% yield, 20.8 mg, 0.050 mmol, >20:1 d.r., >20:1 r.r., 97.5:2.5 e.r.) as a colorless oil.

^{1}H NMR ($CDCl_3$, 400 MHz): δ [ppm] 9.65 (d, *J* = 1.2 Hz, 1H), 7.33 (d, *J* = 8.5 Hz, 2H), 7.17 (d, *J* = 8.5 Hz, 2H), 5.96 - 5.95 (m, 1H), 4.43 (dd, *J* = 4.3, 1.9 Hz, 1H), 4.10 - 3.98 (m, 2H), 3.69-3.66 (m, 1H), 2.59 (d, *J* = 9.4, 7.6 Hz, 1H), 2.54 (d, *J* = 0.8 Hz, 3H), 2.38 - 2.29 (m, 2H), 2.24 (s, 3H), 2.20 (dd, *J* = 7.6, 1.9 Hz, 1H), 1.09 (t, *J* = 7.2 Hz, 3H).

^{13}C NMR ($CDCl_3$, 100 MHz): δ [ppm] 201.3, 172.2, 171.1, 152.5, 144.1, 137.9, 133.3, 129.1 (2C), 127.6 (2C), 110.8, 61.4, 59.4, 53.6, 51.7, 49.5, 45.9, 45.7, 14.4, 14.1, 14.0.

HRMS (ESI+): *m/z* calcd. for $C_{22}H_{24}ClN_2O_4$ $[M+H]^+$: 415.1420; found: 415.1427.

UPC^2: IB-3, CO_2:MeCN gradient [99:1 (0.5 min); 99:1 to 60:40 (over 4 min); 60:40 (1.5 min)], 3.0 mL·min^{-1}, 40 °C, 120 bar] t_{major} = 2.94 min; t_{minor} = 3.16 min, 97.5:2.5.

$[\alpha]_D^{298\,K}$ = -38.6 (*c* 1.0, $CDCl_3$) for 97.5:2.5 e.r.

Ethyl (2*S*,3*S*)-1-(3,5-Dimethyl-1*H*-pyrazole-1-carbonyl)-3-formyl-4-(3-methoxyphenyl)bicyclo[2.1.1]hexane-2-carboxylate, 5n

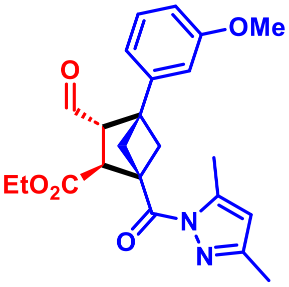

5n

Performed on 0.10 mmol scale following GP2. Full conversion was achieved in 27 h. Purified by FC (silica gel, 7:1 pentane:Et_2O) to obtain **5n** (52% yield, 21.3 mg, 0.052 mmol, >20:1 d.r., >20:1 r.r., 96.5:3.5 e.r.) as a colorless oil.

^{1}H NMR ($CDCl_3$, 400 MHz): δ [ppm] 9.65 (d, J = 1.1 Hz, 1H), 7.27 (t, J = 8.0 Hz, 1H), 6.85 - 6.79 (m, 2H), 6.78 - 6.75 (m, 1H), 5.97 - 5.93 (m, 1H), 4.45 (dd, J = 4.3, 1.9 Hz, 1H), 4.10 - 3.98 (m, 2H), 3.81 (s, 3H), 3.70 - 3.67 (m, 1H), 2.60-2.53 (m, 4H), 2.39-2.30 (m, 2H), 2.24 (s, 3H), 2.22 (d, J = 7.7, 1.9 Hz, 1H), 1.09 (t, J = 7.2 Hz, 3H).

^{13}C NMR ($CDCl_3$, 100 MHz): δ [ppm] 201.6, 172.2, 171.3, 160.0, 152.4, 144.0, 141.0, 130.0, 118.4, 112.7, 111.9, 110.7, 61.3, 59.6, 55.4, 53.5, 52.3, 49.4, 45.9, 45.7, 14.4, 14.1, 14.0.

HRMS (ESI+): *m/z* calcd. for $C_{23}H_{27}N_2O_5$ [M+H]+: 411.1914; found: 411.1919.

UPC^2: IB-3, CO_2:CH_2Cl_2 gradient [99:1 (0.5 min); 99:1 to 90:10 (over 10 min)], 3.0 mL·min^{-1}, 40 °C, 120 bar] t_{major} = 6.72 min; t_{minor} = 7.23 min, 96.5:3.5.

$[\alpha]_D^{298\,K}$ = -40.4 (*c* 1.0, $CDCl_3$) for 96.5:3.5 e.r.

(2*S*,3*S*)-3,4-Dibenzoyl-1-phenylbicyclo[2.1.1]hexane-2-carbaldehyde, 7a

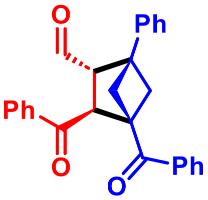

7a

Performed on 0.050 mmol scale following GP3. Full conversion was achieved in 18 h. Purified by FC (silica gel, 9:1 pentane:Et_2O) to obtain **7a** (72% yield, 14.1 mg, 0.036 mmol, >20:1 d.r., >20:1 r.r., 95:5 e.r.) as a yellow solid.

^{1}H NMR ($CDCl_3$, 400 MHz): δ [ppm] 9.64 (d, *J* = 0.8 Hz, 1H), 7.79 (td, *J* = 8.2, 1.3 Hz, 4H), 7.53 – 7.21 (m, 11H), 5.07 (dd, *J* = 4.4, 1.9 Hz, 1H), 3.74 – 3.67 (m, 1H), 2.78 (dd, *J* = 9.5, 7.5 Hz, 1H), 2.55 (dd, *J* = 9.6, 7.4 Hz, 1H), 2.41 (dd, *J* = 7.5, 1.9 Hz, 1H), 2.33 (dd, *J* = 7.3, 1.8 Hz, 1H).

^{13}C NMR ($CDCl_3$, 100 MHz): δ [ppm] 202.3, 200.1, 199.6, 139.1, 137.2, 136.2, 133.6, 132.8, 129.0 (2C), 128.7 (2C), 128.6 (4C), 128.5 (2C), 127.7, 125.9 (2C), 60.4, 57.1, 53.6, 51.6, 46.7, 44.8.

HRMS (ESI+): *m/z* calcd. for $C_{27}H_{23}O_3$ [M+H]$^+$: 395.1642; found: 395.1644.

UPC^2: ID-3, CO_2:MeCN, gradient [99:1 (0.5 min), 99:1 to 60:40 (over 4 min), 60:40 (5.5 min)], 120 bar, 40 °C, 2.0 mL min^{-1}; t_{major} = 4.38 min; t_{minor} = 4.87 min, 95:5 e.r.

$[\alpha]_D^{298\,K}$ = -32.8 (*c* 1.0, CH_2Cl_2) for 95:5 e.r.

(2*S*,3*S*)-4-Benzoyl-3-(4-bromobenzoyl)-1-phenylbicyclo[2.1.1]hexane-2-carbaldehyde, 7b

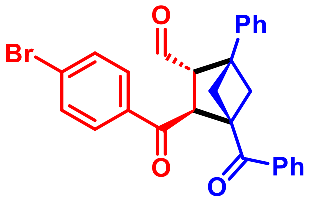

7b

Performed on 0.050 mmol scale following GP3. Full conversion was achieved in 18 h. Purified by FC (silica gel, 9:1 pentane:Et_2O) to obtain **7b** (76% yield, 17.9 mg, 0.038 mmol, >20:1 d.r., >20:1 r.r., 93:7 e.r.) as a yellow solid.

^{1}H NMR ($CDCl_3$, 400 MHz): δ [ppm] 9.62 (d, *J* = 0.7 Hz, 1H), 7.81 – 7.75 (m, 2H), 7.67 – 7.61 (m, 2H), 7.54 – 7.48 (m, 1H), 7.47 – 7.33 (m, 6H), 7.32 – 7.27 (m, 1H), 7.26 – 7.23 (m, 2H), 5.00 (dd, *J* = 4.4, 1.8 Hz, 1H), 3.68 (dd, *J* = 4.3, 1.6 Hz, 1H), 2.80 (dd, *J* = 9.6, 7.5 Hz, 1H), 2.52 (dd, *J* = 9.6, 7.4 Hz, 1H), 2.40 (dd, *J* = 7.5, 1.8 Hz, 1H), 2.34 (dd, *J* = 7.4, 1.9 Hz, 1H).

^{13}C NMR ($CDCl_3$, 100 MHz): δ [ppm] 202.2, 200.0, 198.7, 138.9, 137.0, 135.0, 133.0, 132.0 (2C), 130.1 (2C), 129.0 (2C), 128.9, 128.7 (2C), 128.4 (2C), 127.7, 125.9 (2C), 60.2, 57.3, 53.6, 51.3, 46.6, 45.0.

HRMS (ESI+): *m/z* calcd. for $C_{27}H_{22}{}^{79}BrO_3$ $[M+H]^+$: 473.0747; found: 473.0745. *m/z* calcd. for $C_{27}H_{22}{}^{81}BrO_3$ $[M+H]^+$: 475.0727; found: 475.0729.

UPC^2: IB-3, CO_2:CH_2Cl_2, gradient [99:1 (0.5 min), 99:1 to 60:40 (over 4 min), 60:40 (5.5 min)], 120 bar, 40 °C, 2.0 mL min^{-1}; t_{major} = 5.46 min; t_{minor} = 5.72 min, 93:7 e.r.

$[\alpha]_D^{298\,K}$ = -16.9 (*c* 1.0, CH_2Cl_2) for 94:6 e.r.

(2*S*,3*S*)-4-Benzoyl-3-(4-fluorobenzoyl)-1-phenylbicyclo[2.1.1]hexane-2-carbaldehyde, 7c

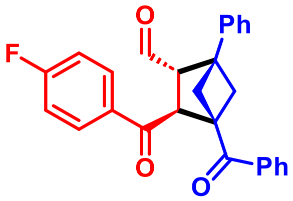

7c

Performed on 0.050 mmol scale following GP3. Full conversion was achieved in 18 h. Purified by FC (silica gel, 9:1 pentane:Et_2O) to obtain **7c** (73% yield, 15.0 mg, 0.036 mmol, >20:1 d.r., >20:1 r.r., 93.5:6.5 e.r.) as a yellow solid.

^{1}H NMR ($CDCl_3$, 400 MHz): δ [ppm] 9.66 – 9.60 (m, 1H), 7.87 – 7.75 (m, 4H), 7.53 – 7.47 (m, 1H), 7.43 – 7.33 (m, 4H), 7.32 – 7.21 (m, 3H), 6.96 (dd, *J* = 9.4, 7.8 Hz, 2H), 5.02 (dd, *J* = 4.4, 1.8 Hz, 1H), 3.70 (dd, *J* = 4.8, 1.7 Hz, 1H), 2.82 (dd, *J* = 9.5, 7.5 Hz, 1H), 2.52 (dd, *J* = 9.5, 7.4 Hz, 1H), 2.41 (dd, *J* = 7.5, 1.8 Hz, 1H), 2.34 (dd, *J* = 7.4, 1.8 Hz, 1H).

^{13}C NMR ($CDCl_3$, 100 MHz): δ [ppm] 202.2, 200.0, 198.0, 166.0 (d, *J* = 256 Hz), 138.9, 137.1, 132.9, 132.7 (d, *J* = 3 Hz), 131.3 (d, *J* = 9 Hz, 2C), 129.0 (2C), 128.6 (2C), 128.5 (2C), 127.7, 125.9 (2C), 115.8 (d, *J* = 22 Hz, 2C), 60.3, 57.3, 53.6, 51.2, 46.6, 45.0.

^{19}F NMR (376 MHz, $CDCl_3$) δ [ppm] -97.5 – -109.2 (m).

HRMS (ESI+): *m/z* calcd. for $C_{27}H_{22}FO_3$ $[M+H]^+$: 413.1547; found: 413.1549.

UPC^2: ID-3, CO_2:MeCN, gradient [99:1 (0.5 min), 99:1 to 60:40 (over 4 min), 60:40 (5.5 min)], 120 bar, 40 °C, 2.0 mL min^{-1}; t_{major} = 4.01 min; t_{minor} = 4.53 min, 93.5:6.5 e.r.

$[\alpha]_D^{298\,K}$ = -30.1 (*c* 0.65, CH_2Cl_2) for 93.5:6.5 e.r.

(2*S*,3*S*)-4-Benzoyl-3-(4-methoxybenzoyl)-1-phenylbicyclo[2.1.1]hexane-2-carbaldehyde, 7d

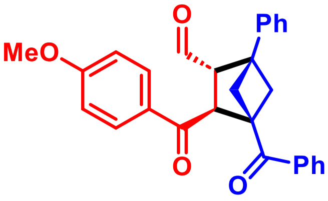

7d

Performed on 0.10 mmol scale following GP3. Full conversion was achieved in 18 h. Purified by FC (iatrobeads, 10:1:1 to 10:2:1 pentane:Et_2O:CH_2Cl_2) to obtain **7d** (91% yield, 38.5 mg, 0.0907 mmol, >20:1 d.r., >20:1 r.r., 92:8 e.r.) as a yellow solid.

^{1}H NMR ($CDCl_3$, 400 MHz): δ [ppm] 9.64 (d, *J* = 1.0 Hz, 1H), 7.82 – 7.71 (m, 4H), 7.57 – 7.47 (m, 1H), 7.45 – 7.33 (m, 4H), 7.32 – 7.22 (m, 3H), 6.83 – 6.75 (m, 2H), 4.94 (dd, *J* = 4.4, 1.9 Hz, 1H), 3.80 (s, 3H), 3.68 (dt, *J* = 4.5, 1.5 Hz, 1H), 2.76 (dd, *J* = 9.5, 7.4 Hz, 1H), 2.53 (dd, *J* = 9.5, 7.4 Hz, 1H), 2.34 (td, *J* = 7.4, 1.9 Hz, 2H).

^{13}C NMR ($CDCl_3$, 100 MHz): δ [ppm] 202.6, 200.3, 197.9, 164.2, 139.6, 137.6, 132.9, 131.1 (2C), 129.5, 129.1 (2C), 128.8 (2C) 128.6 (2C), 127.7, 126.2 (2C), 114.0 (2C), 60.8, 57.3, 55.9, 53.7, 51.7, 46.9, 45.1.

HRMS (ESI+): *m*/*z* calcd. for $C_{28}H_{24}O_4Na$ $[M+Na]^+$: 447.1567; found: 447.1574.

UPC^2: IB-3, CO_2/CH_2Cl_2, gradient [99:1 (0.5 min), 99:1 to 60:40 (over 4 min), 60:40 (5.5 min)], 120 bar, 40 °C, 2.0 mL min^{-1}; t_{major} = 4.73 min; t_{minor} = 4.53 min, 92:8 e.r.

$[\alpha]_D^{298\,K}$ = –20.2 (*c* 1.0, CH_2Cl_2) for 92:8 e.r.

(2*S*,3*S*)-4-Benzoyl-3-(3-methoxybenzoyl)-1-phenylbicyclo[2.1.1]hexane-2-carbaldehyde, 7e

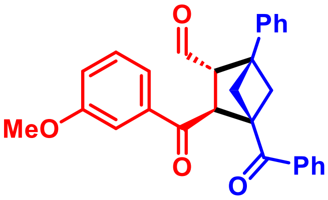

7e

Performed on 0.10 mmol scale following GP3. Full conversion was achieved in 18 h. Purified by FC (iatrobeads, 10:1:1 to 10:2:1 pentane:Et_2O:CH_2Cl_2) to obtain **7e** (92% yield, 39.1 mg, 0.0921 mmol, >20:1 d.r., >20:1 r.r., 94.5:5.5 e.r.) as a yellow solid.

^{1}H NMR (CD_2Cl_2, 400 MHz): δ [ppm] 9.63 (d, *J* = 0.9 Hz, 1H), 7.84 – 7.76 (m, 2H), 7.57 – 7.48 (m, 1H), 7.46 – 7.32 (m, 5H), 7.32 – 7.17 (m, 5H), 7.02 (ddd, *J* = 8.2, 2.7, 1.0 Hz, 1H), 4.98 (dd, *J* = 4.5, 1.9 Hz, 1H), 3.75 (s, 3H), 3.66 (d, *J* = 3.5 Hz, 1H), 2.70 (dd, *J* = 9.6, 7.4 Hz, 1H), 2.52 (dd, *J* = 9.5, 7.4 Hz, 1H), 2.36 (ddd, *J* = 13.5, 7.4, 1.9 Hz, 2H).

^{13}C NMR ($CDCl_3$, 100 MHz): δ [ppm] 202.4, 200.1, 199.5, 160.1, 139.5, 137.8, 137.5, 132.9, 129.9, 129.1 (2C), 128.8 (2C), 128.6 (2C), 127.8, 126.2 (2C), 121.2, 120.4, 112.8, 60.7, 57.2, 55.7, 53.8, 52.2, 47.0, 45.0.

HRMS (ESI+): *m*/*z* calcd. for $C_{28}H_{25}O_4$ $[M+H]^+$: 425.1747; found: 425.1762.

UPC^2: IC-3, CO_2/*i*PrOH, gradient [99:1 (0.5 min), 99:1 to 60:40 (over 4 min), 60:40 (5.5 min)], 120 bar, 40 °C, 2.0 mL min^{-1}; t_{minor} = 4.88 min; t_{major} = 5.25 min, 94.5:5.5 e.r.

$[\alpha]_D^{298\,K}$ = –34.2 (*c* 1.0, CH_2Cl_2) for 94.5:5.5 e.r.

(2*S*,3*S*)-4-Benzoyl-3-(2-methoxybenzoyl)-1-phenylbicyclo[2.1.1]hexane-2-carbaldehyde, 7f

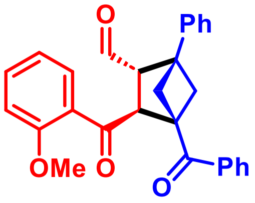

7f

Performed on 0.10 mmol scale following GP3. Full conversion was achieved in 18 h. Purified by FC (silica gel, 8:1:1 pentane:Et_2O:CH_2Cl_2) to obtain **7f** (63% yield, 26.7 mg, 0.063 mmol, 80% purity, >20:1 d.r., >20:1 r.r., 93:7 e.r.) as a yellow solid.

^{1}H NMR ($CDCl_3$, 400 MHz): δ [ppm] 9.71 (d, *J* = 2.1 Hz, 1H), 7.84 – 7.76 (m, 2H), 7.52 – 7.43 (m, 2H), 7.42 – 7.36 (m, 2H), 7.36 – 7.30 (m, 3H), 7.29 – 7.23 (m, 1H), 7.23 – 7.18 (m, 2H), 6.90 (td, *J* = 7.5, 1.0 Hz, 1H), 6.67 (dd, *J* = 8.4, 1.0 Hz, 1H), 5.06 (dd, *J* = 4.3, 2.0 Hz, 1H), 3.71 (dt, *J* = 4.1, 2.0 Hz, 1H), 3.64 (s, 3H), 2.74 (dd, *J* = 9.5, 7.3 Hz, 1H), 2.54 (dd, *J* = 9.5, 7.5 Hz, 1H), 2.32 (td, *J* = 7.1, 1.9 Hz, 2H).

^{13}C NMR ($CDCl_3$, 100 MHz): δ [ppm] 202.5, 201.5, 199.8, 157.8, 139.4, 136.9, 133.9, 132.5, 130.7, 128.8 (2C), 128.4 (4C), 127.5, 127.4, 126.0 (2C), 120.9, 111.1, 59.2, 57.7, 57.4, 55.2, 53.8, 46.2, 45.1.

HRMS (ESI+): *m/z* calcd. for $C_{28}H_{25}O_4$ [M+H]$^+$: 425.1747; found: 425.1747.

UPC^2: IB-3, CO_2:*i*PrOH, gradient [99:1 (0.5 min), 99:1 to 60:40 (over 4 min), 60:40 (5.5 min)], 120 bar, 40 °C, 2.0 mL min^{-1}; t_{minor} = 4.25 min; t_{major} = 4.37 min, 93:7 e.r.

$[\alpha]_D^{298\,K}$ = -19.3 (*c* 0.47, CH_2Cl_2) for 93:7 e.r.

(2*S*,3*S*)-4-Benzoyl-3-hexanoyl-1-phenylbicyclo[2.1.1]hexane-2-carbaldehyde, 7g

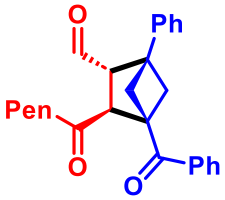

7g

Performed on 0.050 mmol scale following GP3. Full conversion was achieved in 18 h. Purified by FC (silica gel, 9:1 pentane:Et_2O) to obtain **7g** (71% yield, 11.3 mg, 0.036 mmol, >20:1 d.r., >20:1 r.r., 97:3 e.r.) as a yellow solid.

^{1}H NMR ($CDCl_3$, 400 MHz): δ [ppm] 9.64 (d, *J* = 1.0 Hz, 1H), 7.93 – 7.87 (m, 2H), 7.61 – 7.54 (m, 1H), 7.52 – 7.45 (m, 2H), 7.40 – 7.33 (m, 2H), 7.32 – 7.20 (m, 3H), 4.20 (dd, *J* = 4.5, 1.9 Hz, 1H), 3.63 (dt, *J* = 4.6, 1.6 Hz, 1H), 2.64 (dd, *J* = 9.5, 7.5 Hz, 1H), 2.45 (dd, *J* = 9.5, 7.3 Hz, 1H), 2.37 – 2.16 (m, 3H), 1.49 – 1.35 (m, 2H), 1.27 – 1.12 (m, 2H), 1.11 – 1.02 (m, 2H), 0.79 (t, *J* = 7.2 Hz, 3H).

^{13}C NMR ($CDCl_3$, 100 MHz): δ [ppm] 209.5, 202.2, 200.1, 139.0, 136.7, 133.3, 129.0 (2C), 128.9 (2C), 128.6 (2C), 127.6, 125.9 (2C), 59.4, 57.0, 56.0, 52.8, 46.0, 45.7, 42.8, 31.2, 23.2, 22.4, 14.0.

HRMS (ESI+): *m/z* calcd. for $C_{26}H_{29}O_3$ [M+H]$^+$: 389.2111; found: 389.2113.

UPC^2: ID-3, CO_2:MeCN, gradient [99:1 (0.5 min), 99:1 to 60:40 (over 4 min), 60:40 (5.5 min)], 120 bar, 40 °C, 2.0 mL min^{-1}; t_{major} = 3.57 min; t_{minor} = 3.95 min, 97:3 e.r.

$[\alpha]_D^{298\,K}$ = -14.8 (*c* 0.65, CH_2Cl_2) for 97:3 e.r.

(2*S*,3*S*)-4-Benzoyl-1-phenyl-3-pivaloylbicyclo[2.1.1]hexane-2-carbaldehyde, 7h

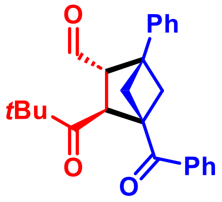

7h

Performed on 0.050 mmol scale following GP3. Full conversion was achieved in 18 h. Purified by FC (silica gel, 9:0.5:0.5 pentane:Et_2O:CH_2Cl_2) to obtain **7h** (59% yield, 11.0 mg, 0.030 mmol, >20:1 d.r., >20:1 r.r., 95.5:4.5 e.r.) as a yellow oil.

^{1}H NMR ($CDCl_3$, 400 MHz): δ [ppm] 9.59 (d, *J* = 1.4 Hz, 1H), 7.87 – 7.81 (m, 2H), 7.56 – 7.50 (m, 1H), 7.48 – 7.41 (m, 2H), 7.38 – 7.31 (m, 2H), 7.31 – 7.24 (m, 1H), 7.23 – 7.17 (m, 2H), 4.51 (dd, *J* = 4.6, 1.8 Hz, 1H), 3.39 (dt, *J* = 4.7, 1.6 Hz, 1H), 3.22 (dd, *J* = 9.6, 7.3 Hz, 1H), 2.44 (dd, *J* = 9.6, 7.4 Hz, 1H), 2.33 (dd, *J* = 7.3, 1.8 Hz, 1H), 2.23 (dd, *J* = 7.4, 1.8 Hz, 1H), 0.95 (s, 9H).

^{13}C NMR ($CDCl_3$, 100 MHz): δ [ppm] 216.3, 202.0, 200.6, 139.0, 136.9, 133.0, 128.9 (2C), 128.7 (4C), 127.6, 125.9 (2C), 62.0, 58.2, 53.5, 48.9, 46.8, 45.0, 26.1 (3C).

HRMS (ESI+): *m/z* calcd. for $C_{25}H_{27}O_3$ $[M+H]^+$: 375.1955; found: 375.1954.

UPC²: IB-3, CO_2:MeCN, gradient [99:1 (0.5 min), 99:1 to 60:40 (over 4 min), 60:40 (5.5 min)], 120 bar, 40 °C, 2.0 mL min^{-1}; t_{major} = 3.19 min; t_{minor} = 3.66 min, 95.5:4.5 e.r.

$[\alpha]_D^{298\,K}$ = -61.1 (*c* 0.29, CH_2Cl_2) for 95.5:4.5 e.r.

(2*S*,3*S*)-4-(3,5-Dimethyl-1*H*-pyrazole-1-carbonyl)-3-hexanoyl-1-phenylbicyclo[2.1.1]hexane-2-carbaldehyde, 7i

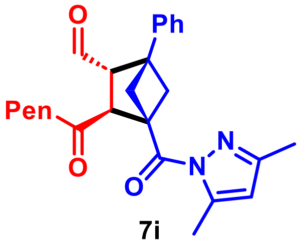

7i

Performed on 0.050 mmol scale following GP3. Full conversion was achieved in 18 h. Purified by FC (silica gel, 9:1 pentane:Et_2O) to obtain **7i** (58% yield, 9.7 mg, 0.029 mmol, >20:1 d.r., >20:1 r.r., 96:4 e.r.) as a yellow solid.

^{1}H NMR ($CDCl_3$, 400 MHz): δ [ppm] 9.66 (d, *J* = 1.5 Hz, 1H), 7.36 (tt, *J* = 6.9, 1.0 Hz, 2H), 7.31 – 7.21 (m, 3H), 5.99 – 5.92 (m, 1H), 4.56 (dd, *J* = 4.8, 1.8 Hz, 1H), 3.48 (dt, *J* = 4.8, 1.8 Hz, 1H), 2.62 – 2.51 (m, 4H), 2.42 – 2.35 (m, 2H), 2.32 – 2.21 (m, 6H), 1.54 – 1.44 (m, 2H), 1.28 – 1.08 (m, 4H), 0.83 (t, *J* = 7.2 Hz, 3H).

^{13}C NMR ($CDCl_3$, 100 MHz): δ [ppm] 209.5, 202.1, 171.5, 152.6, 144.4, 139.5, 128.9 (2C), 127.5, 126.1 (2C), 110.9, 59.2, 56.6, 53.5, 52.1, 45.8, 45.4, 41.9, 31.4, 23.4, 22.5, 14.5, 14.1, 14.0.

HRMS (ESI+): *m/z* calcd. for $C_{25}H_{31}N_2O_3$ $[M+H]^+$: 407.2329; found: 407.2336.

UPC²: IB-3, CO_2:MeCN, gradient [99:1 (0.5 min), 99:1 to 60:40 (over 4 min), 60:40 (5.5 min)], 120 bar, 40 °C, 2.0 mL min^{-1}; t_{major} = 2.92 min; t_{minor} = 3.06 min, 96:4 e.r.

$[\alpha]_D^{298\,K}$ = -32.6 (*c* 0.92, CH_2Cl_2) for 96:4 e.r.

(2*S*,3*S*)-3-(4-Fluorophenyl)-1-phenyl-4-(1*H*-pyrazole-1-carbonyl)bicyclo[2.1.1]hexane-2-carbaldehyde, 7j

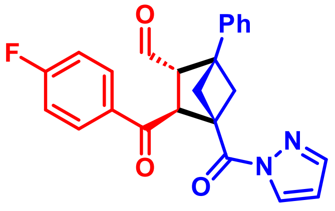

7j

Performed on 0.10 mmol scale following GP3. Full conversion was achieved in 24 h. Purified by FC (silica gel, 10:1 pentane:Et_2O) to obtain **7j** (45% yield, 18.0 mg, 0.45 mmol, >20:1 d.r., >20:1 r.r., 92.5:7.5 e.r.) as an off-white solid.

^{1}H NMR ($CDCl_3$, 400 MHz): δ [ppm] 9.63 (d, J = 0.7 Hz, 1H), 8.07 (d, *J* = 2.9 Hz, 1H), 7.84 (dd, *J* = 8.9, 5.4 Hz, 2H), 7.73 (d, *J* = 1.4 Hz, 1H), 7.41-7.34 (m, 2H), 7.32-7.27 (m, 1H), 7.27-7.22 (m, 2H), 6.98 (t, *J* = 8.8 Hz, 2H), 6.37 (dd, *J* = 2.8, 1.5 Hz, 1H), 5.40 (dd, *J* = 4.5, 1.7 Hz, 1H), 3.68 (dd, *J* = 4.5, 1.5 Hz, 1H), 2.69-2.61 (m, 2H), 2.51-2.43 (m, 2H).

^{13}C NMR ($CDCl_3$, 100 MHz): δ [ppm] 202.1, 197.8, 169.6, 165.9 (d, *J* = 245.9 Hz,), 144.5, 139.0, 132.7 (d, J = 3.0 Hz), 131.1 (d, *J* = 9.5 Hz, 2C), 129.0 (2C), 129.0, 127.7, 126.0 (2C), 115.8 (d, *J* = 21.8 Hz, 2C), 109.5, 59.9, 53.3, 52.9, 51.7, 45.9, 45.1.

^{19}F NMR (376 MHz, $CDCl_3$): δ [ppm] -104.4.

HRMS (ESI+): *m/z* calcd. for $C_{24}H_{19}FN_2O_3Na$ [M+Na]+: 425.1272; found: 425.1271.

UPC^2: IB-3, CO_2:CH_2Cl_2 gradient [99:1 (0.5 min); 99:1 to 90:10 (over 10 min)], 3.0 mL·min^{-1}, 40 °C, 120 bar] t_{major} = 3.69 min; t_{minor} = 4.11 min, 92.5:7.5 e.r.

$[\alpha]_D^{298\,K}$ = -23.5 (*c* 0.7, $CDCl_3$) for 92.5:7.5 e.r.

Ethyl (*E*)-3-((2*S*,3*S*)-3-(4-fluorobenzoyl)-4-(methoxy(methyl)carbamoyl)-1-phenylbicyclo[2.1.1]hexan-2-yl)acrylate, 7k

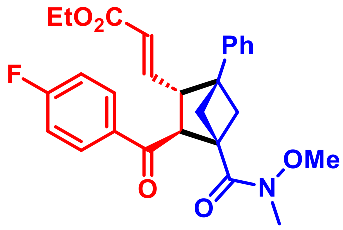

7k

Performed on 0.050 mmol scale following GP3. Full conversion was achieved in 18 h after which the mixture was reduced approximately by half and Wittig reagent (5.0 equiv.) was added and the mixture was stirred for a further 6 h. Purified by FC (silica gel, 3:2 Et_2O/pentane) to obtain **7k** (57% yield, 13.3 mg, 0.029 mmol, >20:1 d.r., >20:1 r.r., 94:6 e.r.) as a colorless oil.

^{1}H NMR ($CDCl_3$, 400 MHz): δ [ppm] 7.99 – 7.95 (m, 2H), 7.33 – 7.29 (dd, *J* = 8.1, 6.9 Hz, 2H), 7.24 – 7.20 (m, 1H), 7.18 – 7.09 (m, 4H), 6.98 – 6.92 (dd, *J* = 15.6, 9.2 Hz, 1H), 5.70 – 5.66 (dd, *J* = 15.6, 0.9 Hz, 1H), 4.20 (dd, *J* = 4.3, 1.5 Hz, 1H), 4.16 – 4.05 (qd, *J* = 7.1, 2.5 Hz, 2H), 3.72 (m, 1H), 3.61 (s, 3H), 2.77 (s, 3H), 2.63 – 2.49 (ddd, *J* = 38.8, 9.5, 7.3 Hz, 1H), 2.33 (m, 1H), 2.11 (dd, *J* = 7.3, 1.6 Hz, 1H), 1.24 – 1.20 (t, *J* = 7.1 Hz, 3H).

^{13}C NMR ($CDCl_3$, 100 MHz): δ [ppm] 196.9, 166.1, 165.8 (d, *J* = 244 Hz), 147.7, 139.8, 133.4 (d, *J* = 2.9 Hz), 131.4 (d, *J* = 9.5 Hz), 128.4 (2C), 126.9, 126.2 (2C), 124.0, 115.6 (d, *J* = 21 Hz), 61.4, 60.4, 55.7, 53.8, 53.1, 50.1, 44.8, 44.6, 32.2, 14.2.

^{19}F NMR ($CDCl_3$, 376 MHz): δ [ppm] – 104.81 (s).

HRMS (ESI+): *m/z* calcd. for $C_{27}H_{29}FNO_5$ [M+H]$^+$: 466.2024; found: 466.2019.

UPC^2: IB-3, CO_2/MeCN, gradient [99:1 (0.5 min), 99:1 to 60:40 (over 4 min), 60:40 (1.5 min)], 120 bar, 40 °C, 2.0 mL min^{-1}; t_{major} = 3.41 min; t_{minor} = 3.56 min, 94:6 e.r.

$[\alpha]_D^{298\,K}$ = -6.2 (*c* 0.55, CH_2Cl_2) for 94:6 e.r.

Methyl (2*S*,3*S*)-3-((*E*)-3-ethoxy-3-oxoprop-1-en-1-yl)-2-(4-fluorobenzoyl)-4-phenylbicyclo[2.1.1]hexane-1-carboxylate, 7l

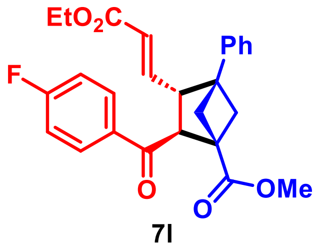

7l

Performed on 0.050 mmol scale following GP3. Full conversion was achieved in 24 h after which Wittig reagent (5.0 equiv.) was added and the mixture was stirred for a further 24 h. Purified by FC (silica gel, 3:1 pentane:Et_2O) to obtain **7l** (69% yield, 15.0 mg, 0,034 mmol, >20:1 d.r., >20:1 r.r., 93.5:6.5 e.r.) as a colorless oil.

^{1}H NMR ($CDCl_3$, 400 MHz): δ [ppm] 7.99-7.91 (m, 2H), 7.31-7.26 (m, 2H), 7.23-7.18 (m, 1H), 7.16-7.04 (m, 4H), 6.99 (dd, *J* = 15.5, 9.7 Hz, 1H), 5.58 (dd, *J* = 15.5, 0.7 Hz, 1H), 4.14 (dq, *J* = 4.4, 1.7 Hz, 2H), 4.08 (dd, *J* = 4.4, 1.7 Hz, 1H), 3.58 (s, 3H), 3.16 (ddd, *J* = 9.6, 4.4, 1.2 Hz, 1H), 2.51 (dd, *J* = 9.5, 7.4 Hz, 1H), 2.41 (dd, *J* = 9.4, 7.2 Hz, 1H), 2.29 (ddd, *J* = 14.6, 7.2, 1.7 Hz, 2H), 1.24 (t, *J* = 7.1 Hz, 3H).

^{13}C NMR ($CDCl_3$, 100 MHz): δ [ppm] 197.8, 171.6, 166.1 (d, *J* = 256.7 Hz), 165.9, 147.1, 139.6, 133.0 (d, *J* = 3.0 Hz), 131.6 (d, *J* = 9.4 Hz, 2C), 128.5 (2C), 127.1, 126.1 (2C), 125.0, 115.8 (d, *J* = 21.9 Hz, 2C), 60.7, 55.4, 54.6, 52.6, 52.0 (2C), 44.5, 43.6, 14.3.

^{19}F NMR (376 MHz, $CDCl_3$): δ [ppm] -104.3 – -104.4 (m).

HRMS (ESI+): *m/z* calcd. for $C_{26}H_{26}FO_5$ [M+H]+: 437.1759; found: 437.1760.

UPC^2: IB-3, $CO_2CH_2Cl_2$ gradient [99:1 (0.5 min); 99:1 to 90:10 (over 10 min)], 3.0 mL·min^{-1}, 40 °C, 120 bar] t_{major} = 3.36 min; t_{minor} = 3.54 min, 93.5:76.5 e.r.

$[\alpha]_D^{298\,K}$ = -6.7 (*c* 1.4, CH_2Cl_2) for 93.5:6.5 e.r.

4.4 Unsuccessful substrates

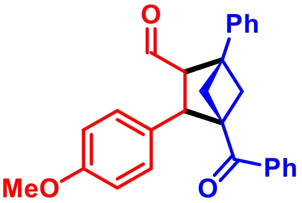

10% conv. after 48 h

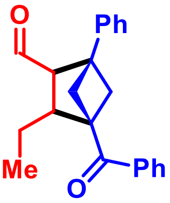

traces

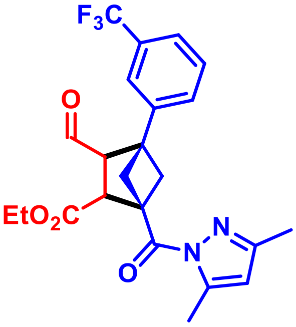

no reactivity

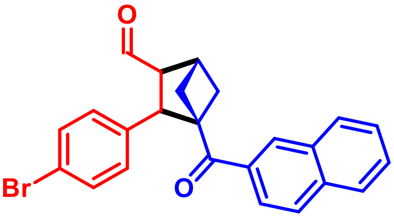

no reactivity

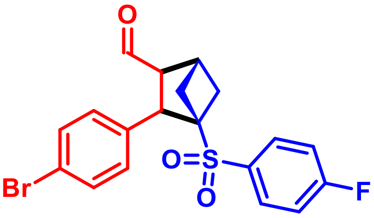

no reactivity

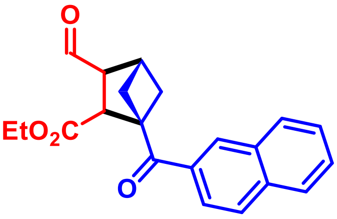

no reactivity

4.5 Synthetic Transformations

((2*R*,3*S*)-2-(4-Bromophenyl)-4-phenylbicyclo[2.1.1]hexane-1,3-diyl)dimethanol, 8a

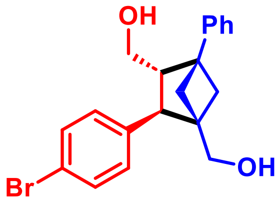

8a

The bicyclohexane **3b** (46.3 mg, 0.10 mmol, 93:7 e.r., 1.0 equiv.) was dissolved in CH_2Cl_2 (0.3 mL) and MeOH (0.1 mL). $NaBH_4$ (9.5 mg, 0.25 mmol, 2.5 equiv.) was added at rt, and the mixture was allowed to stir at rt for 2 h. The mixture was diluted with CH_2Cl_2 (2 mL) and quenched with sat. aq. $NaHCO_3$ (2 mL). The phases were separated, and the aqueous layer was extracted with CH_2Cl_2 (2 x 2 mL). The combined organic layers were dried over $MgSO_4$, filtered and concentrated *in vacuo*. The crude product was purified by FC (silica gel, CH_2Cl_2:MeOH 50:1) to give the diol **8a** as a colorless oil (25.4 mg, 0.068 mmol, 68% yield).

^{1}H NMR (400 MHz, $CDCl_3$): δ [ppm] 7.47 (d, *J* = 8.5 Hz, 1H), 7.39-7.29 (m, 4H), 7.28-7.21 (m, 3H), 3.75 (dd, *J* = 11.2, 6.9 Hz, 1H), 3.61 (dd, *J* = 11.2, 6.2 Hz, 1H), 3.52 (dd, *J* = 13.7, 11.7 Hz, 2H), 3.05 (dd, *J* = 5.0, 1.4 Hz, 1H), 2.77-2.69 (m, 1H), 2.26 (dd, *J* = 9.7, 7.1 Hz, 1H), 2.08 (dd, *J* = 9.6, 6.9 Hz, 1H), 1.90 (dd, *J* = 6.8, 1.8 Hz, 1H), 1.68 (dd, *J* = 7.1, 1.8 Hz, 1H), 1.23 (bs, 1H), 0.89 (bs, 1H).

^{13}C NMR (100 MHz, $CDCl_3$): δ [ppm] 142.2, 140.6, 131.6 (2C), 130.4 (2C), 128.8 (2C), 126.8, 125.8 (2C), 120.7, 64.6, 62.9, 55.2, 53.1, 52.1, 50.8, 42.7, 41.3.

HRMS (ESI+) m/z calcd. for $C_{20}H_{21}{}^{79}BrNaO_2$ $[M+Na]^+$: 395.0616; found: 395.0617, calcd. for $C_{20}H_{21}{}^{81}BrNaO_2$ $[M+Na]^+$: 397.0597; found: 397.0599.

$[\alpha]_D^{298\,K}$ = -48.0 (*c* 1.0, $CDCl_3$).

(2*R*,3*S*)-2-(4-Bromophenyl)-3-formyl-4-phenylbicyclo[2.1.1]hexane-1-carboxylic acid, 8b

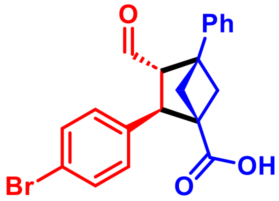

8b

The bicyclohexane **3b** (23.2 mg, 0.050 mmol, 93:7 e.r., 1.0 equiv.) was dissolved in THF/H_2O (v/v = 1:1, 0.7 mL). Lithium hydroxide monohydrate (8.4 mg, 0.20 mmol, 4.0 equiv.) was added at rt, and the mixture was allowed to stir at rt for 2 h. Then EtOAc (3 mL) was added, and the phases were separated. The aqueous phase was acidified with 1 M HCl to pH = 1, and extracted with EtOAc (3 x 10 mL). The combined organic phase was dried with Na_2SO_4, filtered and reduced *in vacuo*. To obtain analytically pure sample, the crude product was purified by FC (silica gel, CH_2Cl_2:MeOH 20:1) to give **8b** as a white solid (12.6 mg, 0.033 mmol, 65% yield).

^{1}H NMR (400 MHz, $CDCl_3$): δ [ppm] 9.65 (d, *J* = 1.7 Hz, 1H), 7.34 (t, *J* = 7.3 Hz, 4H), 7.29 – 7.24 (m, 1H), 7.15 (d, *J* = 6.8 Hz, 2H), 7.10 (d, *J* = 8.4 Hz, 2H), 4.17 (d, *J* = 3.4 Hz, 1H), 3.15 (d, *J* = 4.2 Hz, 1H), 2.44 – 2.33 (m, 1H), 2.27 (t, *J* = 7.9 Hz, 1H), 2.19 (t, *J* = 7.7 Hz, 2H).

^{13}C NMR (100 MHz, $CDCl_3$): δ [ppm] 202.4, 183.6, 139.8, 139.1, 131.9 (2C), 129.3 (2C), 129.0 (2C), 127.6, 125.9 (2C), 120.8, 64.5, 53.3, 51.5, 47.4, 45.0, 44.3.

HRMS (ESI+) m/z calcd. for $C_{20}H_{17}{}^{79}BrO_3Na$ $[M+Na]^+$: 407.0254; found: 407.0253, calcd. for $C_{20}H_{17}{}^{81}BrO_3Na$ $[M+Na]^+$: 409.0233; found: 409.0228.

$[\alpha]_D^{298\,K}$ = -52.8 (*c* 0.25, CH_2Cl_2).

(2*S*,3*R*)-4-(3,5-Dimethyl-1*H*-pyrazole-1-carbonyl)-3-(4'-methoxy-[1,1'-biphenyl]-4-yl)-1-phenylbicyclo[2.1.1]hexane-2-carbaldehyde, 8c

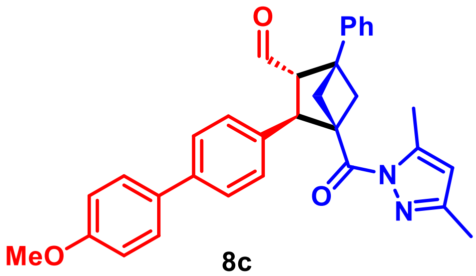

8c

To a Schlenk tube was added bicyclohexane **3b** (46.3 mg, 0.10 mmol, 93:7 e.r., 1.0 equiv.), 4-methoxyphenylboronic acid (30.4 mg, 0.2 mmol, 2 equiv.), K_2CO_3 (27.6 mg, 0.2 mmol, 2 equiv.) and P(*o*-tol)$_3$ (0.6 mg, 2 mol, 2 mol %). The solids were dissolved in 1,4-dioxane:H_2O 5:1 (1.2 mL) and the mixture were purged with Ar for 10 min. $Pd_2(dba)_3 \cdot CHCl_3$(1.0 mg, 1 µmol, 1 mol %) was added and the solution was purged with Ar for 5 further min before being capped and stirred at 50 °C for 2 h. After full conversion was determined, the crude mixture was directly transferred to FC (silica gel, 5:1 pentane:Et_2O) to yield **8c** as a pale-yellow oil (43.5 mg, 0.089 mmol, 89%).

^{1}H NMR (400 MHz, $CDCl_3$): δ [ppm] 9.83 (d, *J* = 2.4 Hz, 1H), 7.51 – 7.44 (m, 4H), 7.40 – 7.34 (m, 2H), 7.31 – 7.24 (m, 5H), 7.00 – 6.94 (m, 2H), 5.94 (d, *J* = 1.2 Hz, 1H), 4.88 (dd, *J* = 4.8, 1.9 Hz, 1H), 3.85 (s, 3H), 3.42 (dt, *J* = 4.8, 2.3 Hz, 1H), 2.93 (dd, *J* = 9.4, 7.5 Hz, 1H), 2.71 (dd, *J* = 9.5, 7.6 Hz, 1H), 2.52 (dd, *J* = 7.4, 2.1 Hz, 1H), 2.47 (d, *J* = 1.1 Hz, 3H), 2.30 (dd, *J* = 7.7, 1.8 Hz, 1H), 2.28 (s, 3H).

^{13}C NMR (100 MHz, $CDCl_3$): δ [ppm] 202.9, 172.5, 159.2, 152.6, 144.2, 139.9, 139.4, 139.0, 133.3, 128.8 (2C), 128.1 (2C), 127.9 (2C), 127.3, 127.0 (2C), 126.2 (2C), 114.3 (2C), 110.9, 63.9, 55.6, 55.4, 52.5, 50.2, 47.1, 44.8, 14.4, 14.2.

HRMS (ESI+) m/z calcd. for $C_{32}H_{31}N_2O_3$ $[M+H]^+$: 491.2329; found: 491.2331.

$[\alpha]_D^{298\,K}$ = +3.5 (*c* 2.67, CH_2Cl_2).

Ethyl (*E*)-3-((2*S*,3*R*)-3-(4-bromophenyl)-4-(3,5-dimethyl-1*H*-pyrazole-1-carbonyl)-1-phenylbicyclo[2.1.1]hexan-2-yl)acrylate, 8d

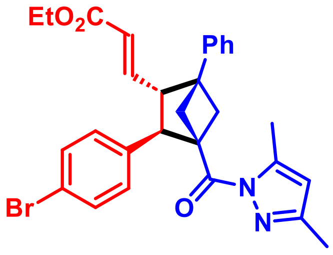

8d

The bicyclohexane **3b** (46.3 mg, 0.10 mmol, 93:7 e.r., 1.0 equiv.) was dissolved in CH_2Cl_2 (0.5 mL). Ethyl (triphenylphosphoranyliden)acetate (52.2 mg, 0.15 mmol, 1.5 equiv.) was added at rt, and the mixture was allowed to stir at rt for 90 min. After full conversion was determined, the crude mixture was directly transferred to FC (silica gel, 9:1 to 4:1 pentane:Et_2O) to yield **8d** as a white solid (48.4 mg, 0.091 mmol, 91%).

^{1}H NMR (400 MHz, $CDCl_3$): δ [ppm] 7.40 – 7.35 (m, 2H), 7.34 – 7.28 (m, 2H), 7.25 – 7.19 (m, 1H), 7.17 – 7.06 (m, 5H), 5.92 (d, *J* = 1.1 Hz, 1H), 5.72 (dd, *J* = 15.5, 0.9 Hz, 1H), 4.27 (dd, *J* = 4.8, 2.0 Hz, 1H), 4.15 (qt, *J* = 7.3, 3.7 Hz, 2H), 3.13 (dddd, *J* = 9.4, 4.8, 2.1, 0.9 Hz, 1H), 2.90 (dd, *J* = 9.4, 7.5 Hz, 1H), 2.58 (dd, *J* = 9.4, 7.6 Hz, 1H), 2.47 – 2.42 (m, 4H), 2.29 – 2.24 (m, 4H), 1.25 (t, *J* = 7.2 Hz, 3H).

^{13}C NMR (100 MHz, $CDCl_3$): δ [ppm] 172.5, 166.4, 152.6, 148.7, 144.2, 140.3, 140.0, 131.8 (2C), 129.1 (2C), 128.5 (2C), 126.9, 126.2 (2C), 123.2, 120.8, 110.9, 60.5, 56.9, 56.1, 55.5, 53.4, 47.2, 43.9, 14.4, 14.3, 14.2.

HRMS (ESI+) m/z calcd. for $C_{29}H_{30}{}^{79}BrN_2O_3$ $[M+H]^+$: 533.1435; found: 533.1425, calcd. for $C_{29}H_{30}{}^{81}BrN_2O_3$ $[M+H]^+$: 535.1414; found: 535.1411.

$[\alpha]_D^{298\,K}$ = -60.9 (*c* 3.0, CH_2Cl_2).

((2*R*,3*S*)-3-((*E*)-(2-(4-bromo-2-nitrophenyl)hydrazineylidene)methyl)-2-(4-bromophenyl)-4-phenylbicyclo[2.1.1]hexan-1-yl)(3,5-dimethyl-1*H*-pyrazol-1-yl)methanone, 8e

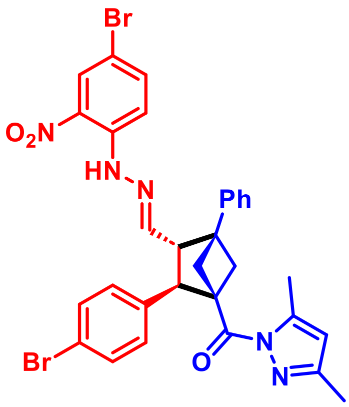

8e

The bicyclohexane **3b** (46.3 mg, 0.10 mmol, 93:7 e.r., 1.0 equiv.) was dissolved in CH_2Cl_2 (0.6 mL) and MeOH (0.3 mL). 4-Bromo-2-nitrophenylhydrazine hydrochloride (32.2 mg, 0.12 mmol, 1.2 equiv.) was added at rt, and the mixture was allowed to stir at rt for 45 min. After full conversion was determined, the crude mixture was directly transferred to FC (silica gel, 9:1 pentane:Et_2O) to yield **8e** as an orange-red solid (55.4 mg, 0.082 mmol, 82%).

^{1}H NMR (400 MHz, $CDCl_3$): δ [ppm] 10.74 (s, 1H), 8.27 (d, *J* = 2.3 Hz, 1H), 7.76 (d, *J* = 9.2 Hz, 1H), 7.52 (dd, *J* = 9.2, 2.3 Hz, 1H), 7.44 – 7.32 (m, 5H), 7.29 – 7.20 (m, 3H), 7.18 – 7.14 (m, 2H), 5.96 (d, *J* = 1.1 Hz, 1H), 4.87 (dd, *J* = 4.8, 2.0 Hz, 1H), 3.38 (td, *J* = 5.1, 2.0 Hz, 1H), 2.90 (dd, *J* = 9.3, 7.3 Hz, 1H), 2.63 (dd, *J* = 9.4, 7.6 Hz, 1H), 2.50 – 2.45 (m, 4H), 2.33 – 2.26 (m, 4H).

^{13}C NMR (100 MHz, $CDCl_3$): δ [ppm] 172.6, 152.5, 147.8, 144.3, 141.2, 140.4, 140.2, 138.9, 131.8 (2C), 130.9, 129.2 (2C), 128.8 (2C), 128.1, 127.1, 126.2 (2C), 120.7, 118.0, 110.9, 109.5, 55.9, 55.5, 53.0, 52.4, 46.8, 44.4, 14.5, 14.2.

HRMS (ESI+) m/z calcd. for $C_{31}H_{28}{}^{79}Br_2N_5O_3$ $[M+H]^+$: 676.0554; found: 676.0552, calcd. for $C_{31}H_{28}{}^{79}Br^{81}BrN_5O_3$ $[M+H]^+$: 678.0533; found: 678.0533, calcd. for $C_{31}H_{28}{}^{81}Br_2N_5O_3$ $[M+H]^+$: 680.0513; found: 680.0520.

$[\alpha]_D^{298\,K}$ = -121.9 (*c* 2.33, CH_2Cl_2).

Methyl (2*R*,3*S*)-3-((benzylamino)methyl)-2-(4-bromophenyl)-4-phenylbicyclo[2.1.1]hexane-1-carboxylate, 8f

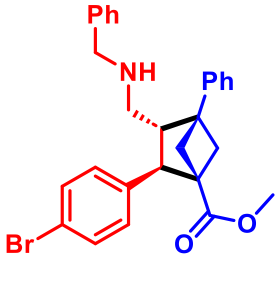

8f

The bicyclohexane **3b** (23.2 mg, 0.050 mmol, 93:7 e.r., 1.0 equiv.) was dissolved in CH_2Cl_2 (0.5 mL). Benzylamine (16.1 mg, 0.15 mmol, 3.0 equiv.) and Et_3N (25.3 mg, 0.25 mmol, 5.0 equiv.) were added at rt, and the mixture was allowed to stir at rt for 2 h. Then $NaBH_4$ (4.8 mg, 0.125 mmol, 2.5 equiv.) and MeOH (0.1 mL) was added at rt, and the mixture was allowed to stir at rt for 1 h. The mixture was diluted with CH_2Cl_2 (2 mL) and quenched with sat. aq. $NaHCO_3$ (2 mL). The phases were separated, and the aqueous layer was extracted with CH_2Cl_2 (2 x 2 mL). The combined organic layers were dried over $MgSO_4$, filtered and concentrated *in vacuo*. The crude product was purified by FC (silica gel, CH_2Cl_2:MeOH 50:1) to give **8f** as a colorless oil (13.2 mg, 0.027 mmol, 54% yield).

^{1}H NMR (400 MHz, $CDCl_3$): δ [ppm] 7.47 – 7.39 (m, 2H), 7.35 – 7.19 (m, 8H), 7.17 – 7.13 (m, 2H), 7.06 – 6.99 (m, 2H), 3.59 (s, 3H), 3.54 (s, 2H), 3.37 (dd, *J* = 4.8, 2.0 Hz, 1H), 2.75 (qd, *J* = 12.0, 6.8 Hz, 2H), 2.59 (tdd, *J* = 6.8, 4.8, 2.1 Hz, 1H), 2.44 (dd, *J* = 9.5, 7.2 Hz, 1H), 2.33 (dd, *J* = 9.5, 6.8 Hz, 1H), 2.17 (dd, *J* = 6.8, 1.9 Hz, 1H), 2.10 (dd, *J* = 7.2, 1.9 Hz, 1H).

^{13}C NMR (100 MHz, $CDCl_3$): δ [ppm] 172.8, 141.4, 140.8, 140.2, 131.6 (2C), 129.8 (2C), 128.7 (2C), 128.4 (2C), 128.0 (2C), 126.9, 126.9, 125.8 (2C), 120.6, 54.0, 53.7, 52.8, 52.7, 52.5, 52.0, 51.7, 44.6, 43.7.

HRMS (ESI+) m/z calcd. for $C_{28}H_{29}{}^{79}BrNO_2$ $[M+H]^+$: 490.1377; found: 490.1385, calcd. for $C_{28}H_{29}{}^{81}BrNO_2$ $[M+H]^+$: 492.1356; found: 492.1368.

$[\alpha]_D^{298\,K}$ = -50.4 (*c* 0.83, CH_2Cl_2).

5. Crystallographic Data

Single crystal of compound **8e** was obtained by using the following procedure: **8e** was dissolved in toluene and crystals were allowed to form by vapor diffusion with pentane. Single crystal X-ray diffraction for **8e** was carried out on a Bruker D8 VENTURE diffractometer with MoKα radiation (λ = 0.71073 Å) at 100 K. As all picked crystals of **8e** turned out racemic, this structure only reflects the relative configuration of **8e**.

Item	Value
Molecular formula	0.667($C_{31}H_{27}Br_2N_5O_3$)
Formula weight	451.60
Crystal system	Monoclinic
Space Group	$P2_1/n$
a (Å)	13.376(1)
b (Å)	12.6964(11)
c (Å)	16.5390(14)
α (°)	90
β (°)	96.340(3)
γ (°)	90
Volume (Å^3)	2791.6(4)
Z	6
T (K)	100
ρ (g cm^{-1})	1.612
λ (Å)	0.71073
μ (mm^{-1})	2.947
# measured refl	5513
# unique refl	3808
R_{int}	0.1385
# parameters	372
R(F^2), all refl	0.0403
R_w(F^2), all refl	0.1167
Goodness of fit	1.062

Crystal data for [**8e**]: 0.667($C_{31}H_{27}Br_2N_5O_3$), M = 451.60, monoclinic, space group $P2_1/n$, a = 13.376(1) Å, b = 12.6964(11) Å, c = 16.5390(14) Å, α = 90°, β = 96.340(3)°, γ = 90°, V = 2791.6(4) Å^3, T = 100 K, Z = 6, d_c = 1.612 g cm^{-3}, μ(Mo Kα, λ = 0.71073 Å) = 2.947 mm^{-1}, 5513 reflections collected, 3808 unique [R_{int} = 0.1385], which were used in all calculations. Refinement on F^2, final R(F) = 0.0403, R_w(F2) = 0.1167. CCDC number 2463774.

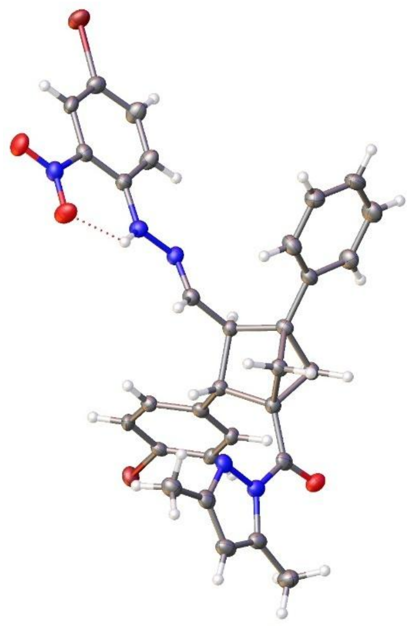

X-Ray crystallographic structure of compound **8e** (50% thermal probability ellipsoids)

6. ECD and UV-vis Calculations for Determination of Absolute Stereochemistry of 3b

To gain insight into the absolute configuration of the bicyclo[2.1.1]hexanes, ECD calculations were carried out. As two enantiomers only differ in sign and not in magnitude, ECD calculations were conducted for one of the enantiomers. The calculations were carried out with the (*S*,*R*)-configured enantiomer as shown in Figure S1.

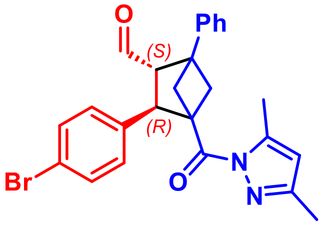

3b

Figure S1. Absolute configuration of **3b** used for ECD and UV-vis calculations.

Experimental ECD spectra were obtained by diluting **3b** in CH_3CN (0.01 mg/mL) in a 1.0 cm quartz glass cuvette at 25 °C. Experimental UV-vis spectrum was obtained by **3b** in CH_3CN (0.01 mg/mL) in a 1.0 cm quartz glass cuvette at 25 °C.

All quantum chemical calculations were performed with Gaussian 16.[2] A total of 10 conformations of **3b** ((*S*,*R*)-configuration) were generated using force-field method[3] (OPLS3e (SPMC), 21 kJ·mol^{-1} range) in Schrödinger-Maestro.[4] The conformers were optimized using DFT with the ωB97xD/pcseg-1/SMD(CH_3CN) level of theory.[5-7] Re-optimization of the 10 structures with ωB97xD/aug-pcseg-1/SMD(CH_3CN) using keyword int(grid=ultrafinegrid,acc2e=15) yielded 9 unique conformations. The free energies reported herein are quasi-harmonic corrected in GoodVibes with a frequency cut-off at 100 cm^{-1}.[8] All structures were confirmed to be minima on the potential energy surface via the inclusion of a frequency calculation revealing no imaginary frequencies.

ECD and UV-vis spectra for each unique ωB97xD/aug-pcseg-1 optimized conformation were predicted via single-point calculation using TD-DFT[9] ωB97xD/aug-pcseg-1/SMD(CH_3CN) and keywords td=(nstates=50, root=1) and int(grid=superfinegrid,acc2e=15). ECD and UV-vis spectra were extracted from GaussView 6[10]] and displayed with band broadening of σ = 0.33 eV (Figure S2). ECD and UV-vis spectrum for **3b** were generated by Boltzmann-weighing each conformation contribution using the Gibbs free energy (ECD: Figure S3). The calculated Boltzmann-averaged ECD spectrum was wavelength corrected by comparing λ_{max} between the calculated UV-vis spectrum and the experimental obtained UV-vis spectrum. This provides a shift for the calculated UV-vis spectrum of +15 nm (Figure S4). The experimental ECD spectrum in CH_3CN was compared to the calculated (wavelength-corrected) ECD spectrum (Figure S5). It strongly suggests the absolute configuration of **3b** to be the (*S*,*R*)-configured product.

Table S1. Energies used to obtain the Boltzmann fraction for each conformation of **3b**. Gibbs free energies were obtained at the ωB97xD/aug-pcseg-1/SMD(CH_3CN) level of theory.

Conformation	Gibbs free energy (Hartree)	Relative energy (kcal/mol)	Boltzmann distribution	Boltzmann fraction
**3b**_1	-3799.4522	0.35	0.55	17.7
**3b**_2	-3799.4503	1.00	0.08	2.6
**3b**_3	-3799.4527	0.00	1.00	32.1
**3b**_4	-3799.4497	1.90	0.04	1.3
**3b**_5	-3799.4523	0.29	0.61	19.6
**3b**_6	-3799.4506	1.33	0.11	3.4
**3b**_7	-3799.4524	0.23	0.68	21.8
**3b**_8	-3799.4476	3.25	0.00	0.1
**3b**_9	-3799.4498	1.83	0.05	1.5

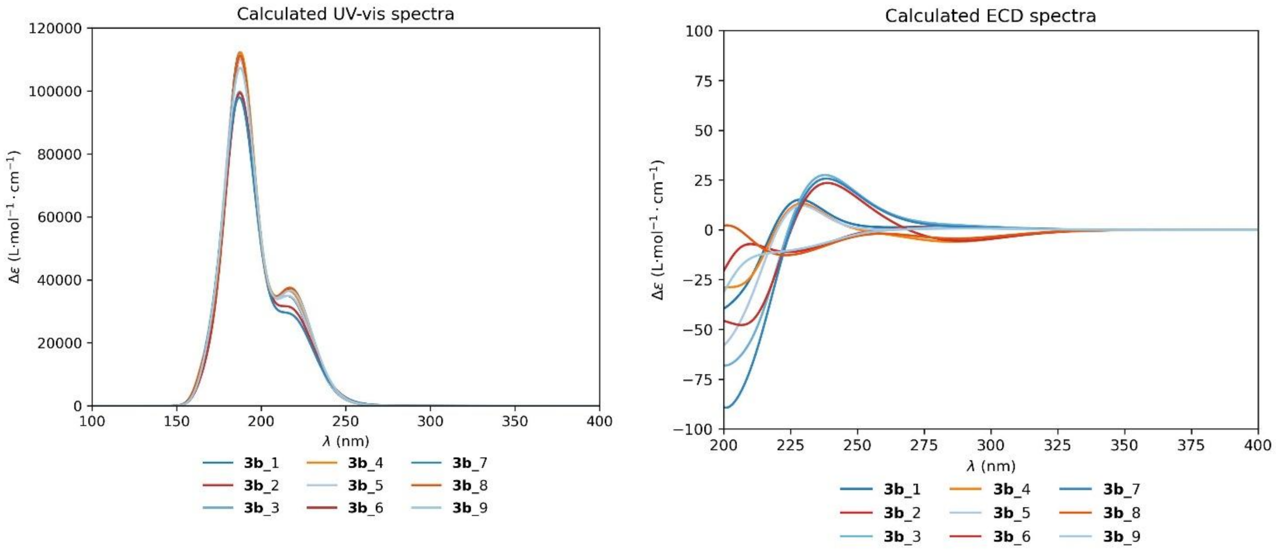

Figure S2. a) Calculated UV-vis spectrum and ECD spectrum for each unique conformation of **3b**.

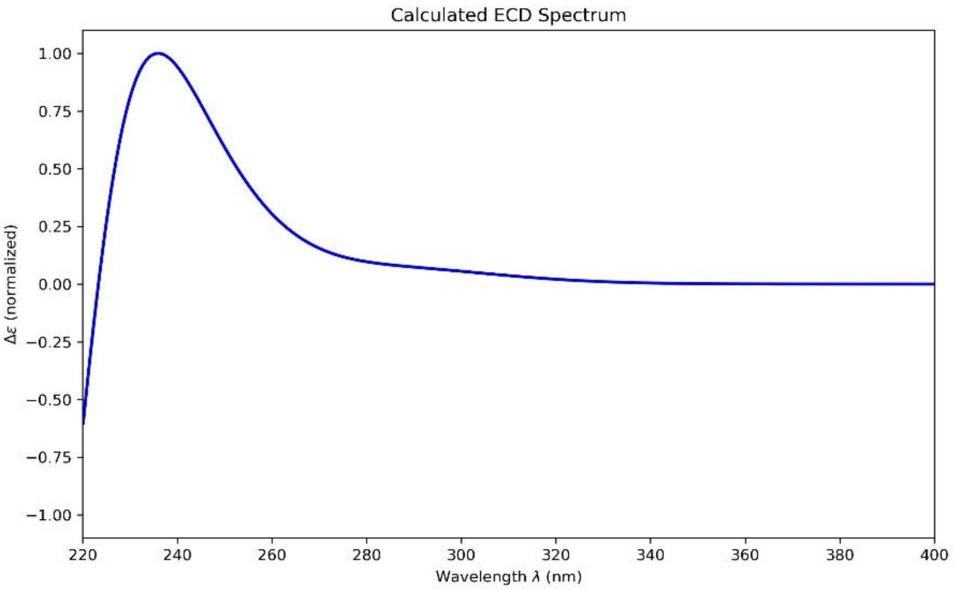

Figure S3. Calculated and normalized ECD spectrum of **3b** by Boltzmann-averaging all the conformations contributing to the spectrum using ωB97xD/aug-pcseg-1 level of theory in CH_3CN.

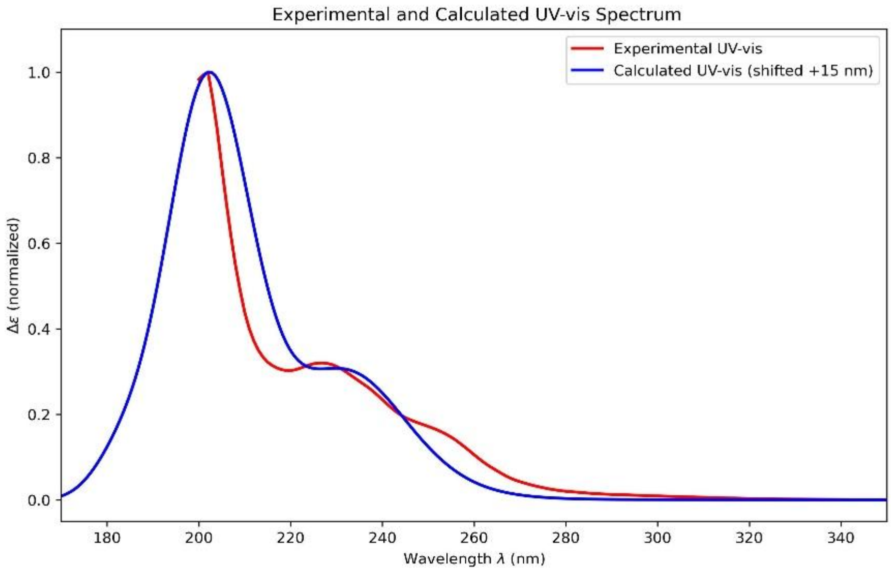

Figure S4. Comparison between calculated Boltzmann-averaged UV-vis spectrum (blue, wavelength corrected by +15 nm) and experimentally obtained UV-vis spectrum (red) of **3b** in CH_3CN. The spectrum has been normalized.

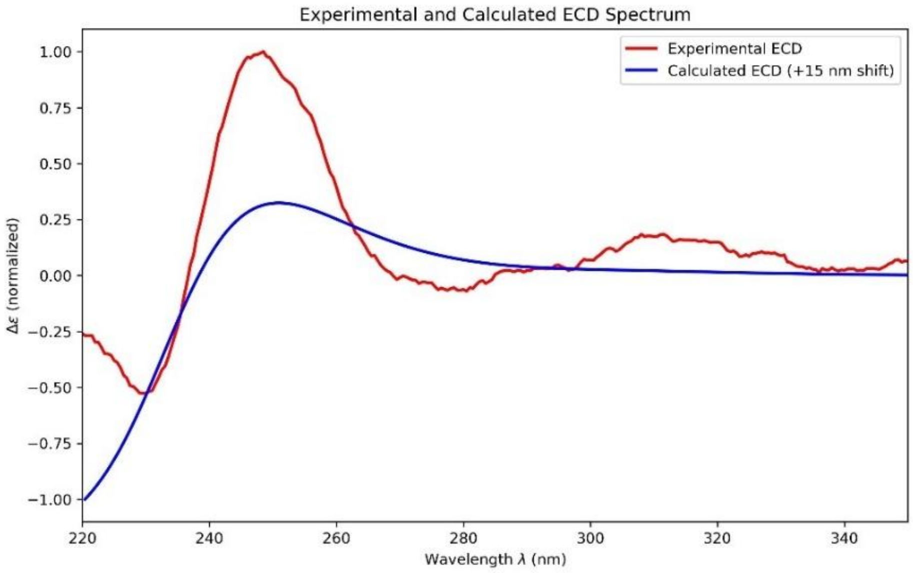

Figure S5. Comparison between calculated Boltzmann-averaged ECD (blue, wavelength corrected by +15 nm) and experimentally obtained ECD (red) of **3b**. The spectrum has been normalized.

7. NMR

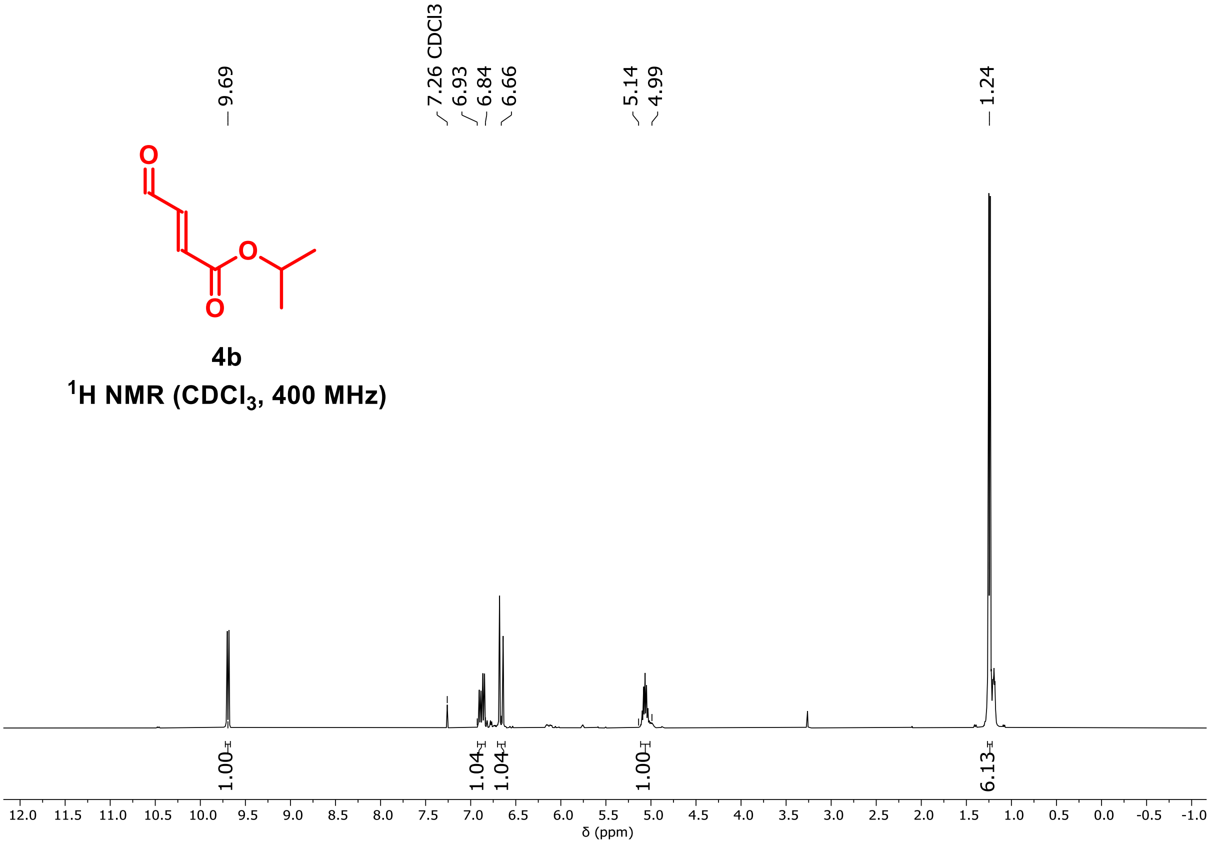
4b
1H NMR (CDCl3, 400 MHz)
9.69
7.26 CDCl3
6.93
6.84
6.66
5.14
4.99
1.24
1.00
1.04
1.04
1.00
6.13
12.0 11.5 11.0 10.5 10.0 9.5 9.0 8.5 8.0 7.5 7.0 6.5 6.0 5.5 5.0 4.5 4.0 3.5 3.0 2.5 2.0 1.5 1.0 0.5 0.0 -0.5 -1.0
δ (ppm)

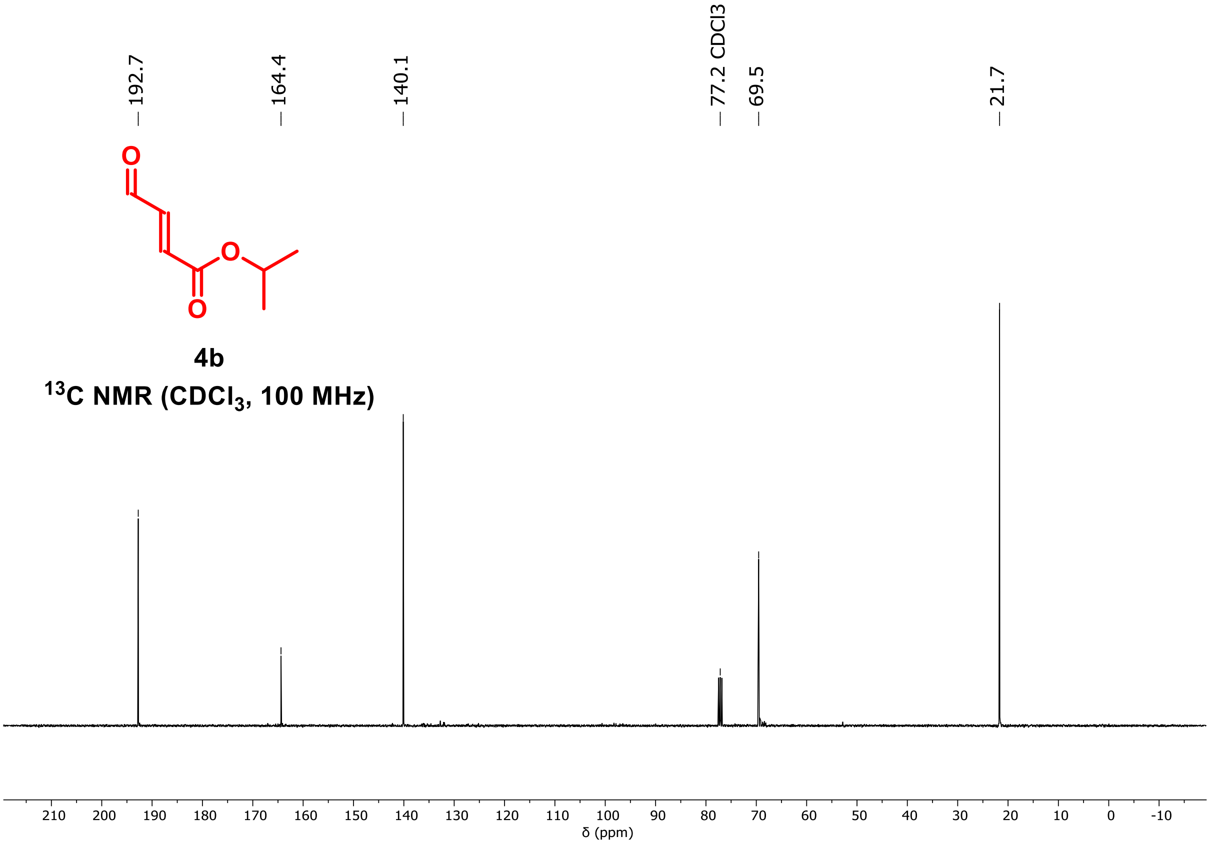
4b
13C NMR (CDCl3, 100 MHz)
192.7
164.4
140.1
77.2 CDCl3
69.5
21.7
210 200 190 180 170 160 150 140 130 120 110 100 90 80 70 60 50 40 30 20 10 0 -10
δ (ppm)

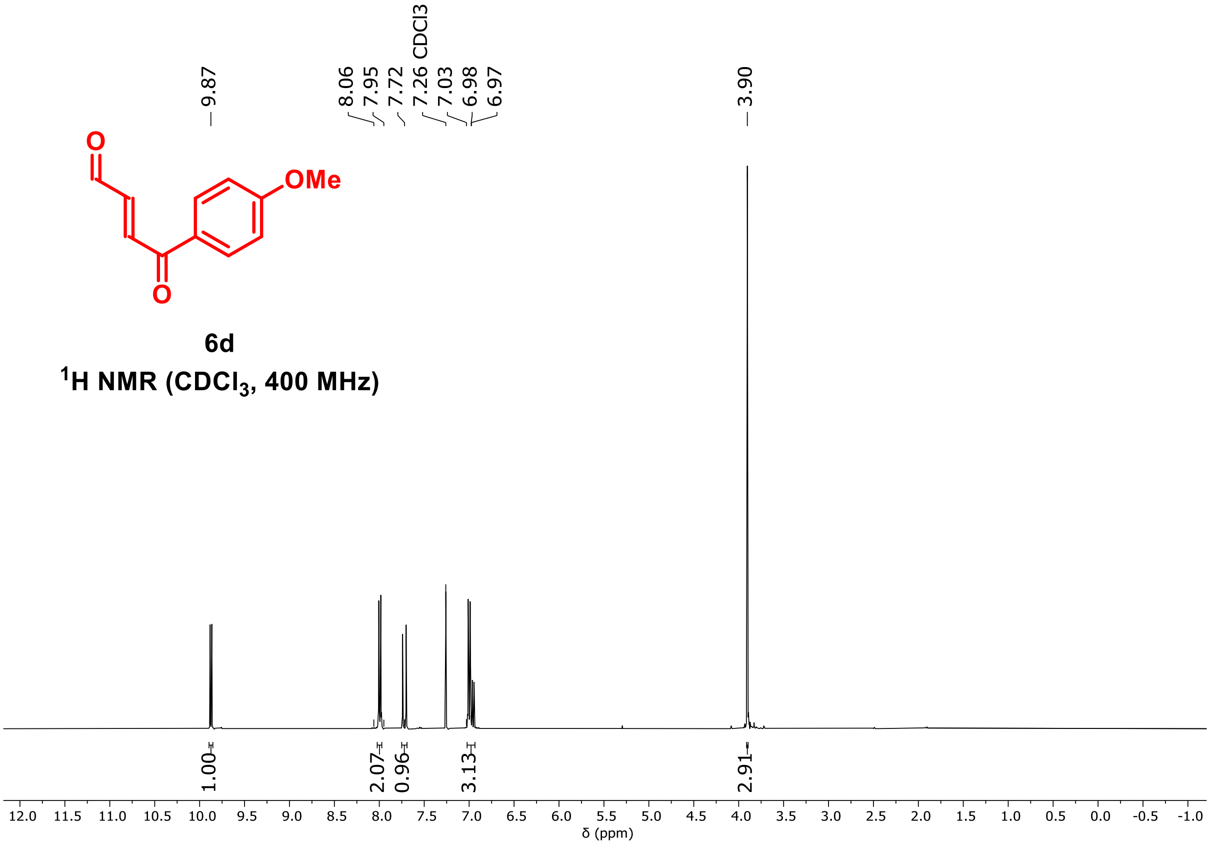

6d

^{1}H NMR ($CDCl_3$, 400 MHz)

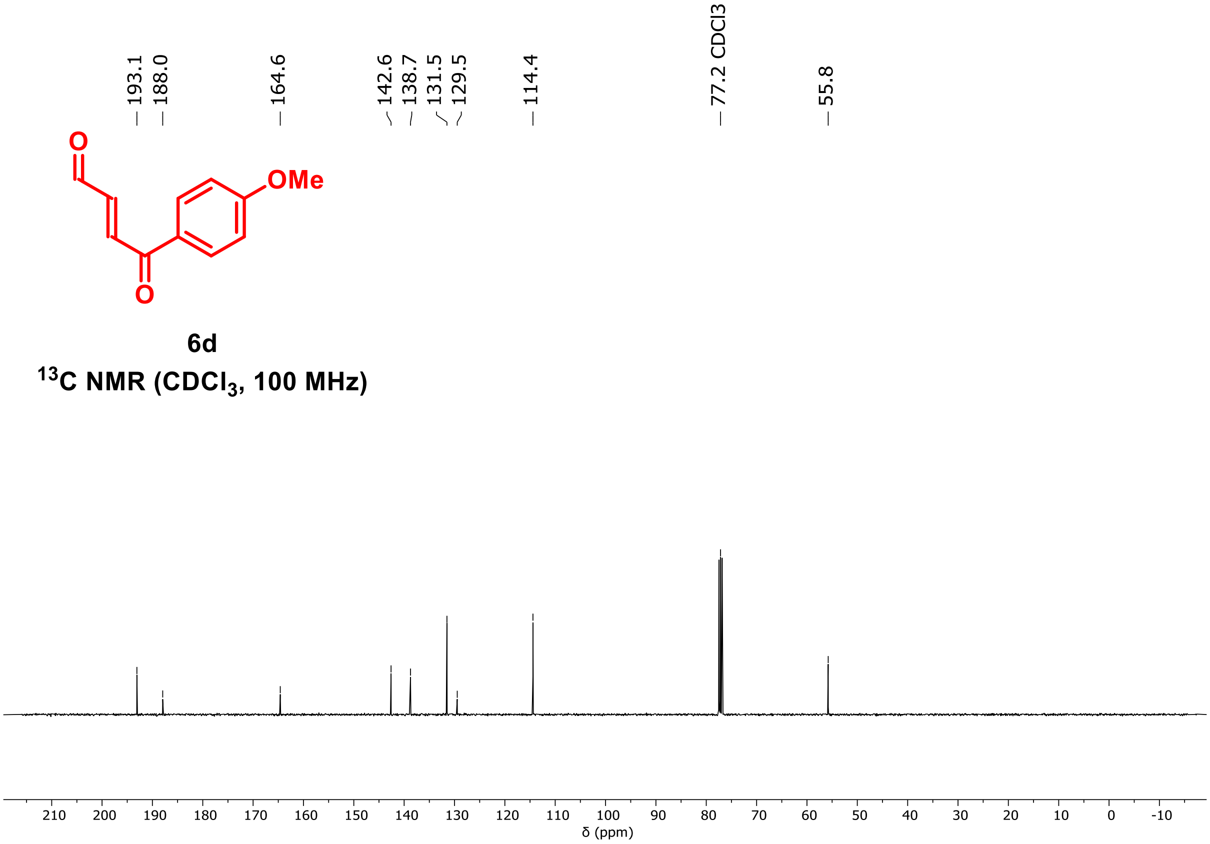

6d

^{13}C NMR ($CDCl_3$, 100 MHz)

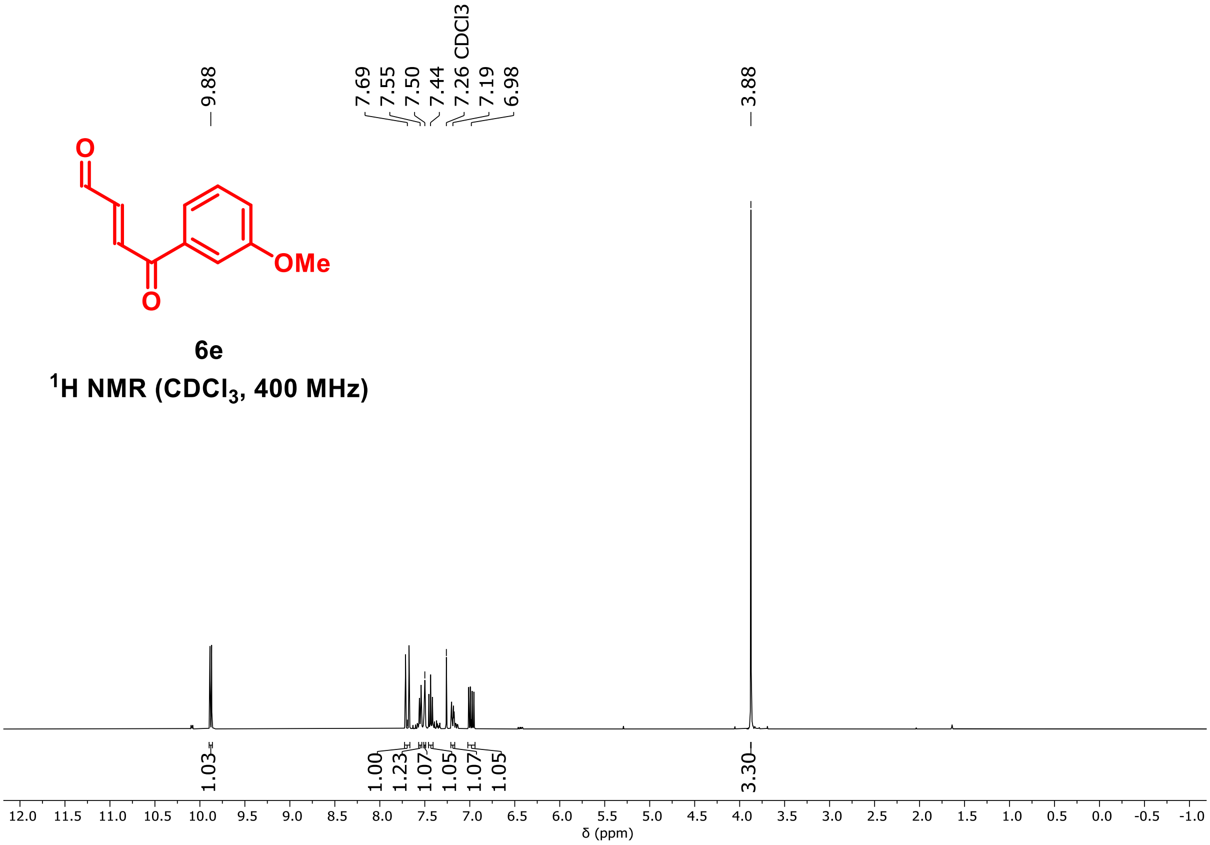

6e

^{1}H NMR ($CDCl_3$, 400 MHz)

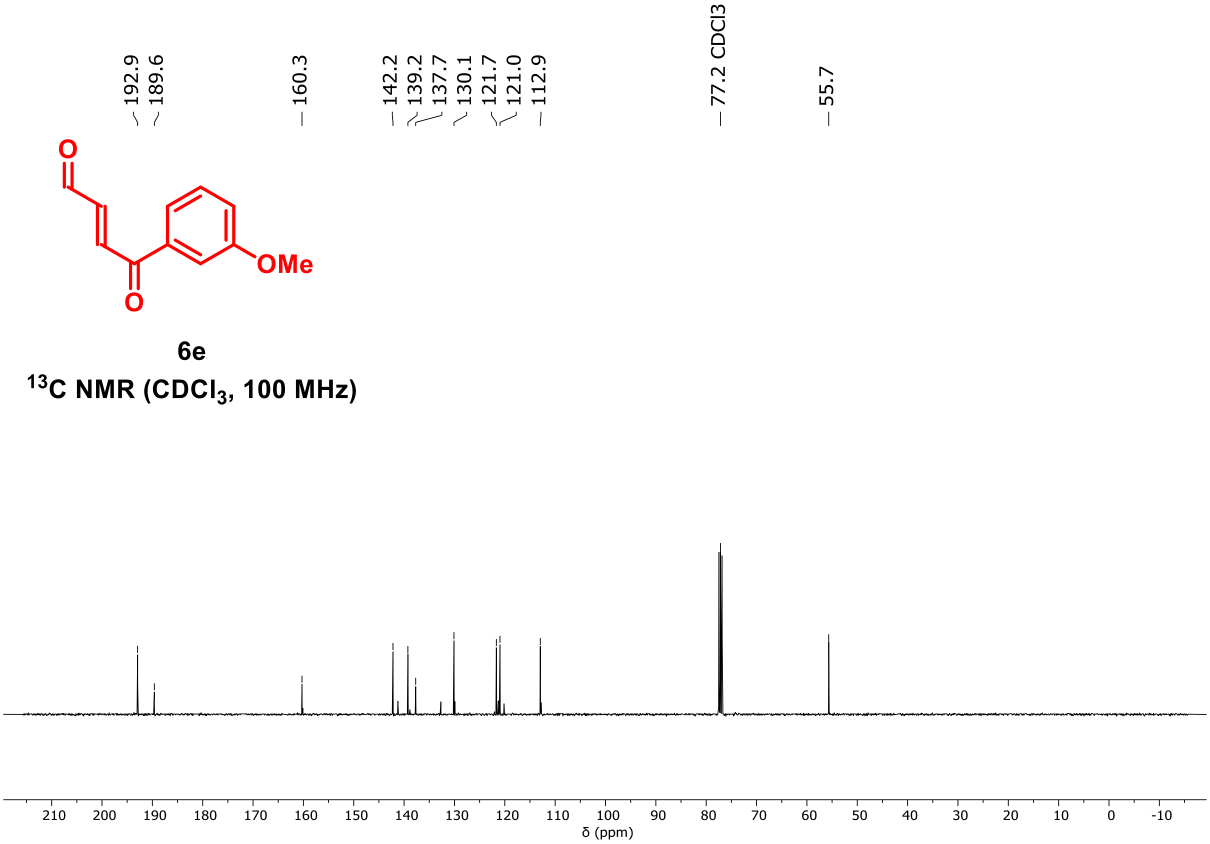

6e

^{13}C NMR ($CDCl_3$, 100 MHz)

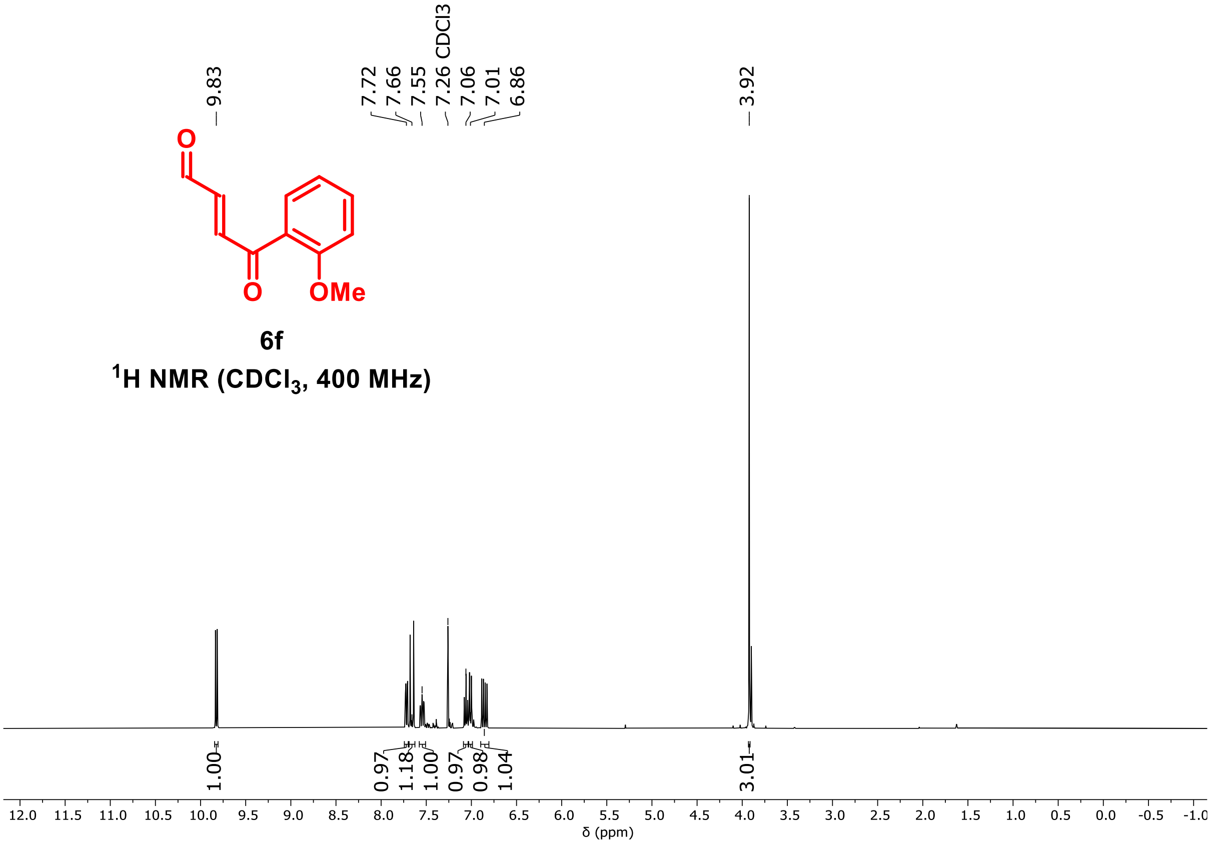
9.83
7.72
7.66
7.55
7.26 CDCl3
7.06
7.01
6.86
3.92
O
OMe
6f
^{1}H NMR ($CDCl_3$, 400 MHz)
1.00
0.97
1.18
1.00
0.97
0.98
1.04
3.01
12.0 11.5 11.0 10.5 10.0 9.5 9.0 8.5 8.0 7.5 7.0 6.5 6.0 5.5 5.0 4.5 4.0 3.5 3.0 2.5 2.0 1.5 1.0 0.5 0.0 -0.5 -1.0
δ (ppm)

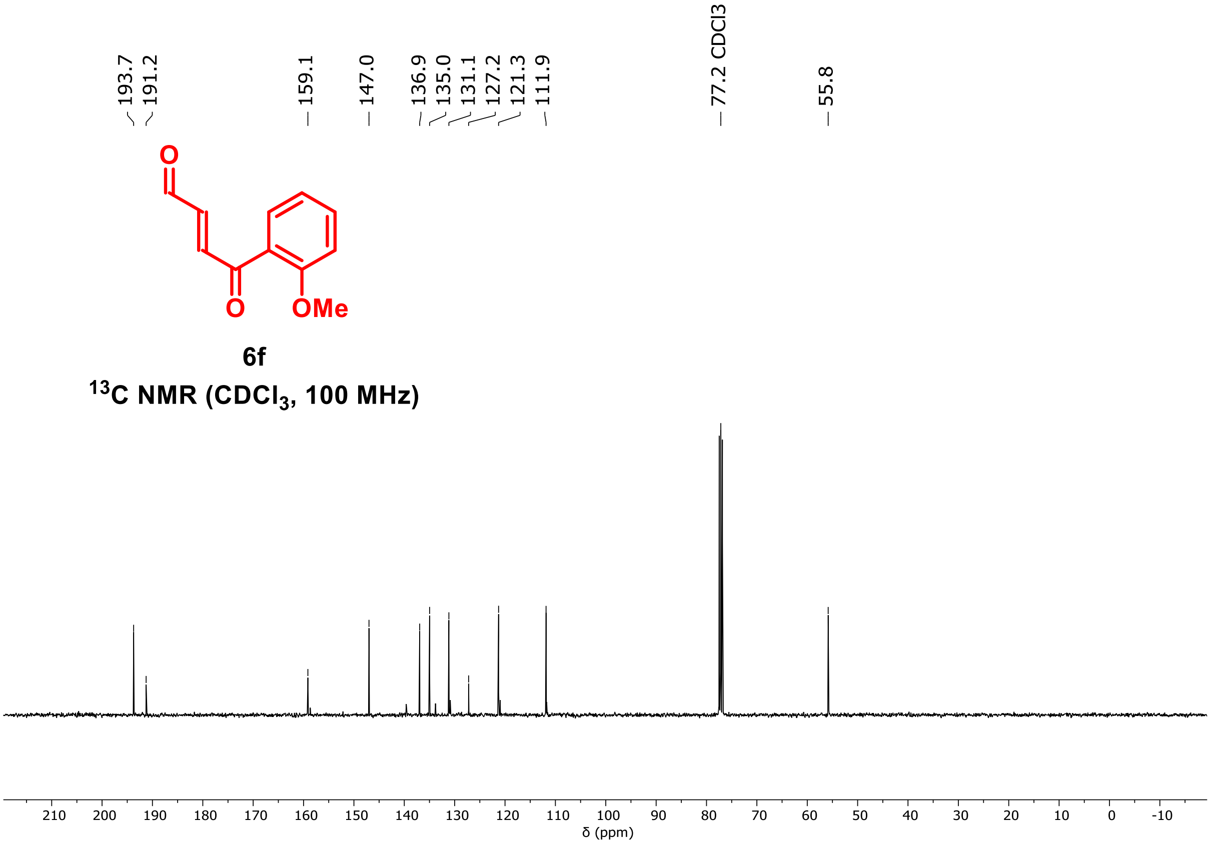
193.7
191.2
159.1
147.0
136.9
135.0
131.1
127.2
121.3
111.9
77.2 CDCl3
55.8
O
OMe
6f
^{13}C NMR ($CDCl_3$, 100 MHz)
210 200 190 180 170 160 150 140 130 120 110 100 90 80 70 60 50 40 30 20 10 0 -10
δ (ppm)

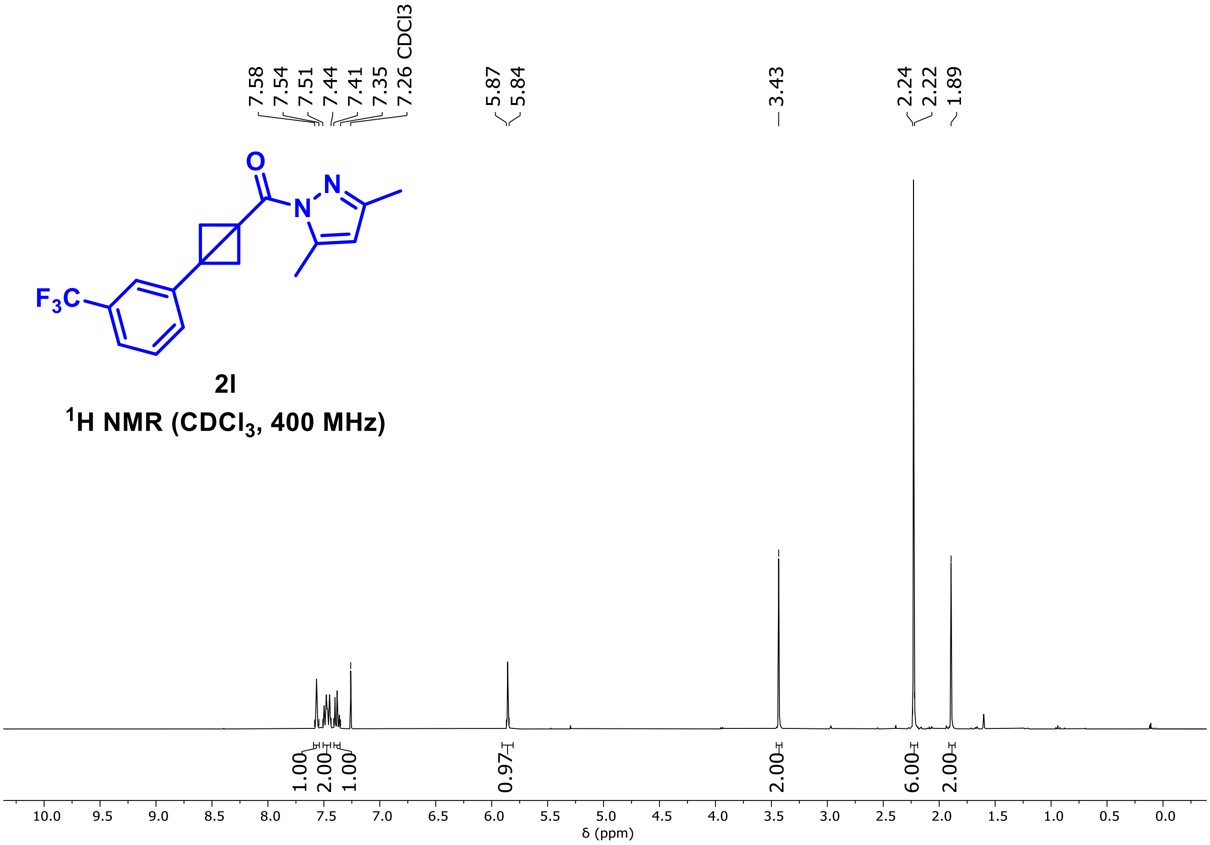
7.58
7.54
7.51
7.44
7.41
7.35
7.26 CDCl3
5.87
5.84
3.43
2.24
2.22
1.89
O
N
N
F3C
2l
1H NMR (CDCl3, 400 MHz)
1.00
2.00
1.00
0.97
2.00
6.00
2.00
10.0 9.5 9.0 8.5 8.0 7.5 7.0 6.5 6.0 5.5 5.0 4.5 4.0 3.5 3.0 2.5 2.0 1.5 1.0 0.5 0.0
δ (ppm)

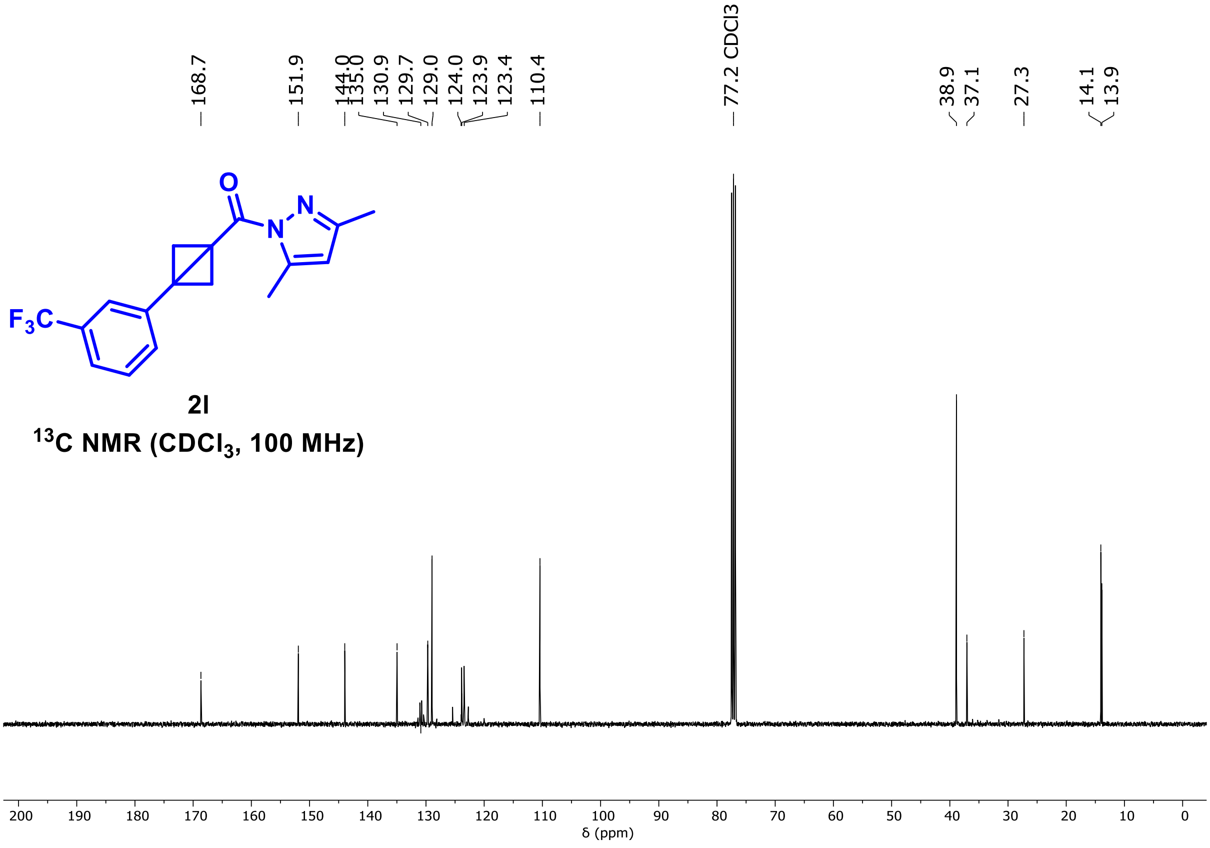
168.7
151.9
144.0
135.0
130.9
129.7
129.0
124.0
123.9
123.4
110.4
77.2 CDCl3
38.9
37.1
27.3
14.1
13.9
O
N
N
F3C
2l
13C NMR (CDCl3, 100 MHz)
200 190 180 170 160 150 140 130 120 110 100 90 80 70 60 50 40 30 20 10 0
δ (ppm)

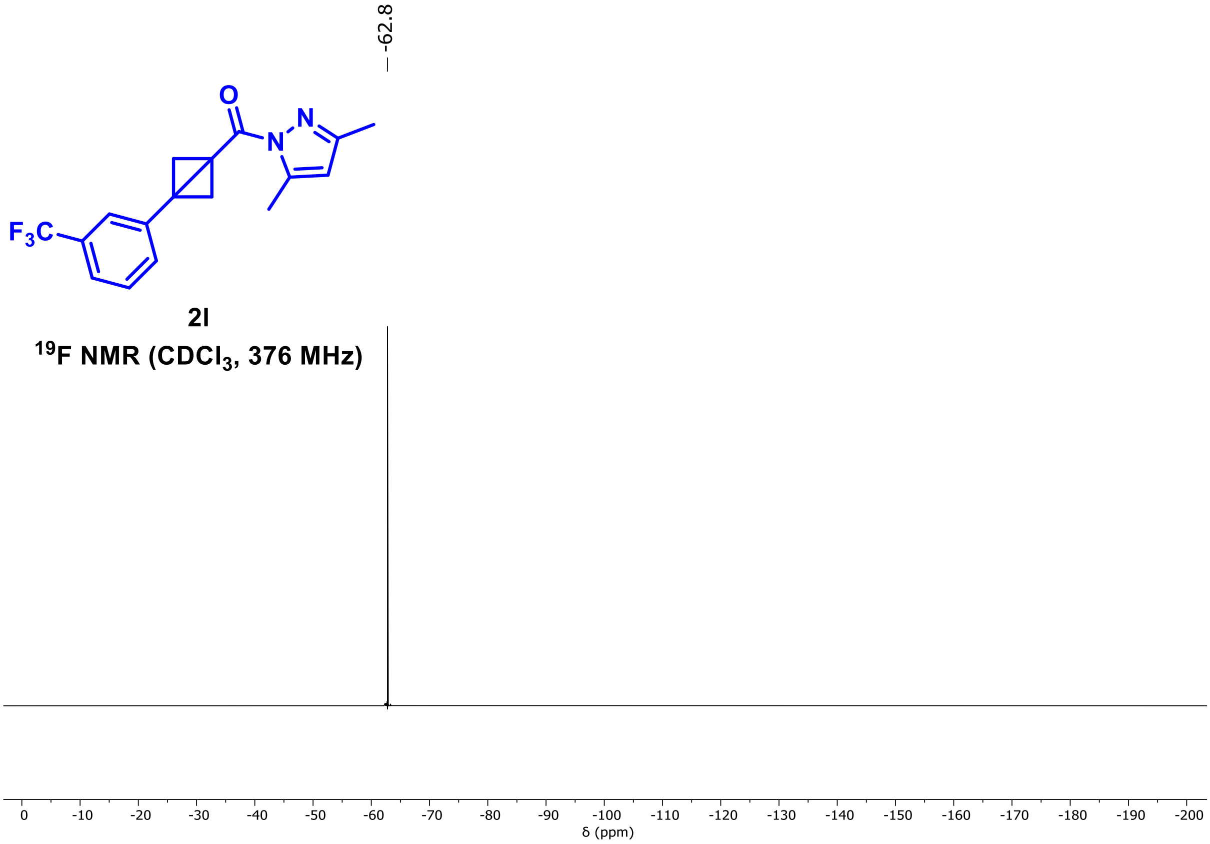

2l

^{19}F NMR ($CDCl_3$, 376 MHz)

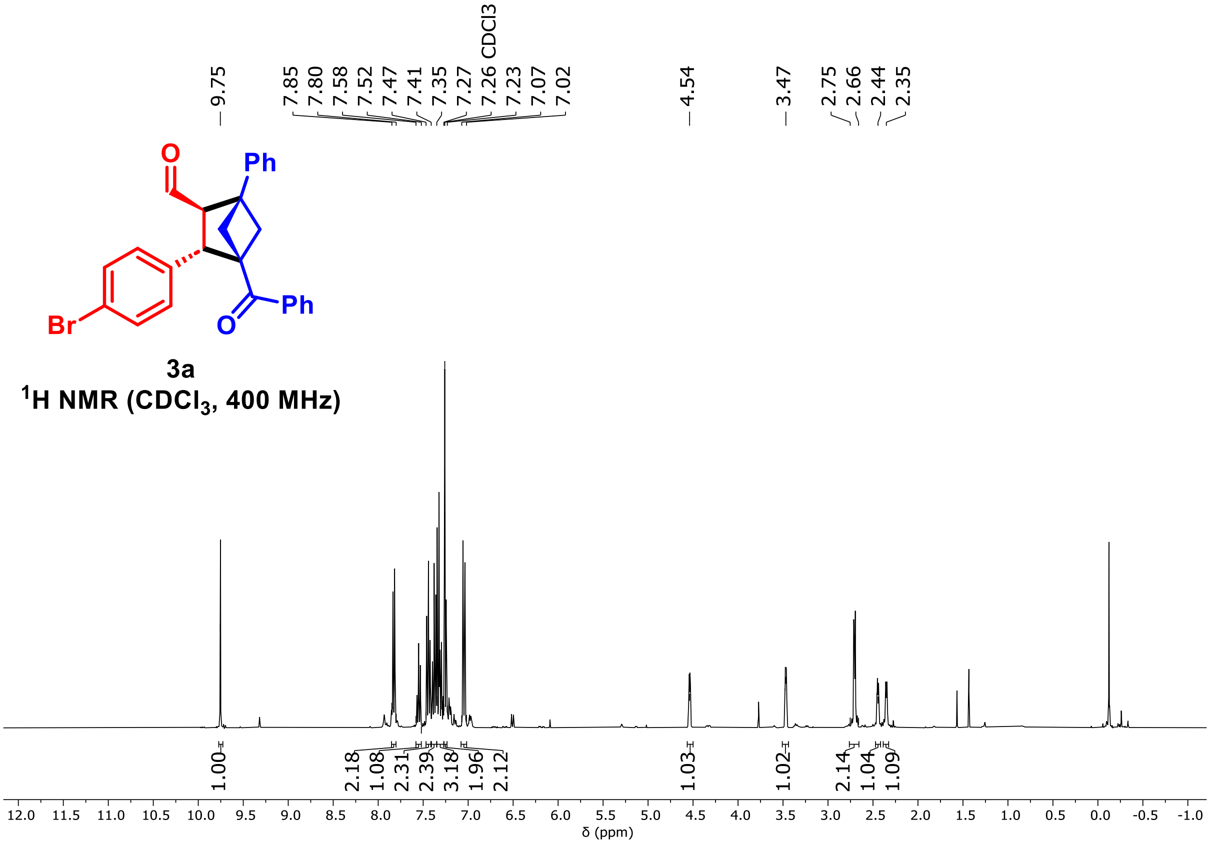

3a
^{1}H NMR ($CDCl_3$, 400 MHz)

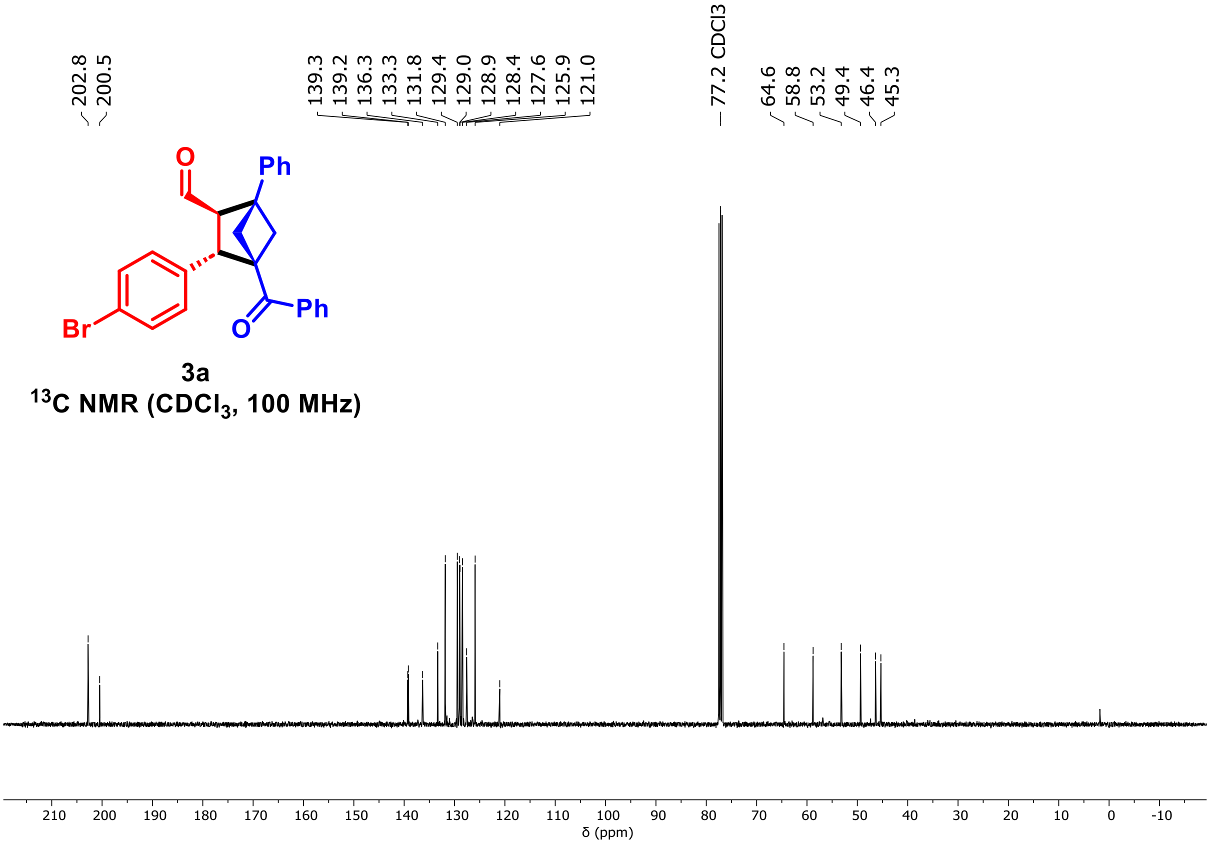

3a
^{13}C NMR ($CDCl_3$, 100 MHz)

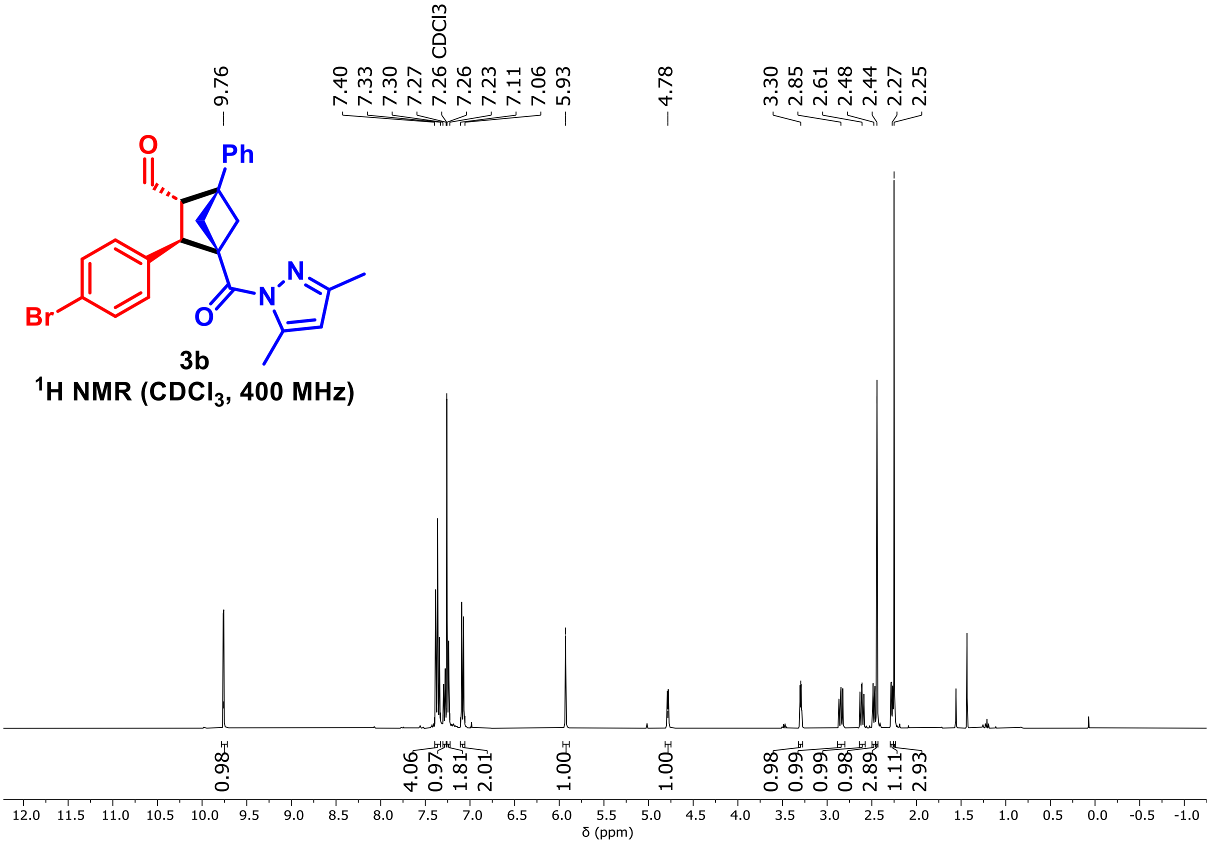

3b

^{1}H NMR ($CDCl_3$, 400 MHz)

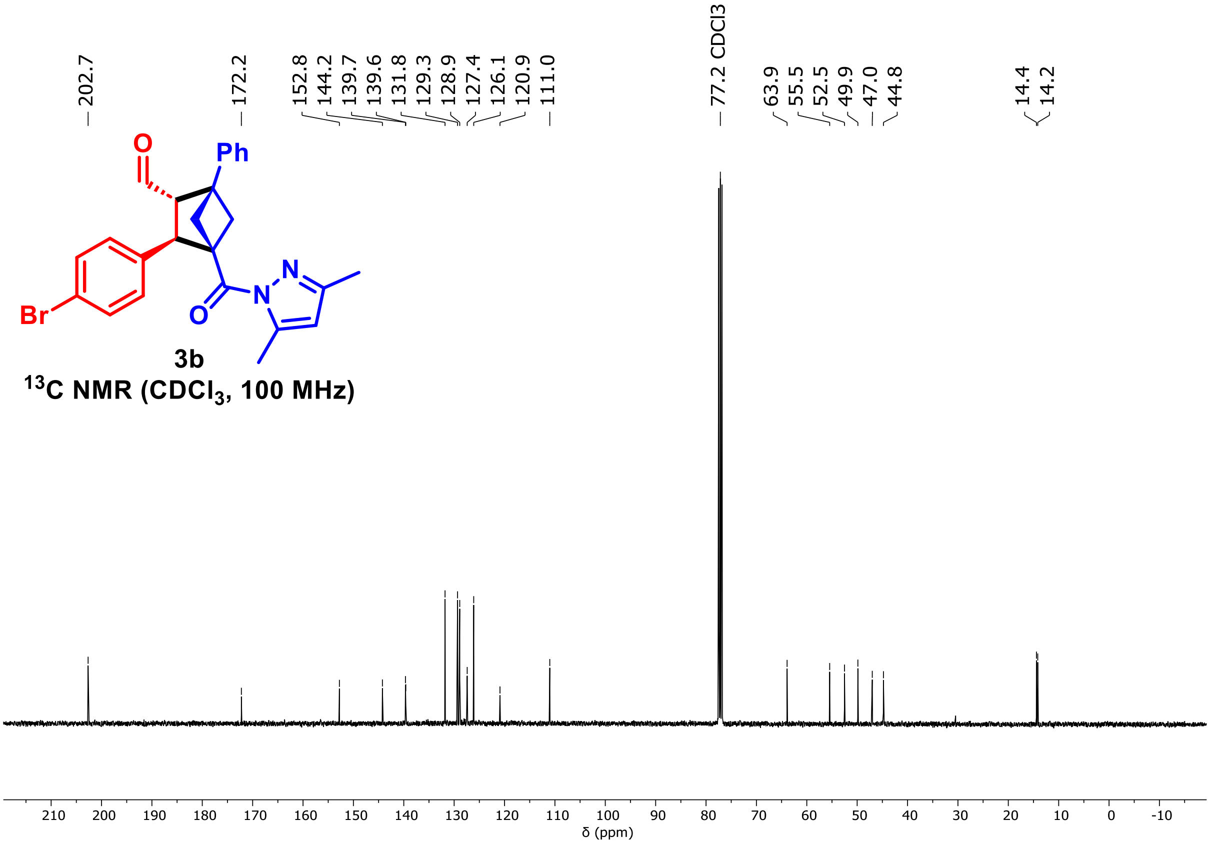

3b

^{13}C NMR ($CDCl_3$, 100 MHz)

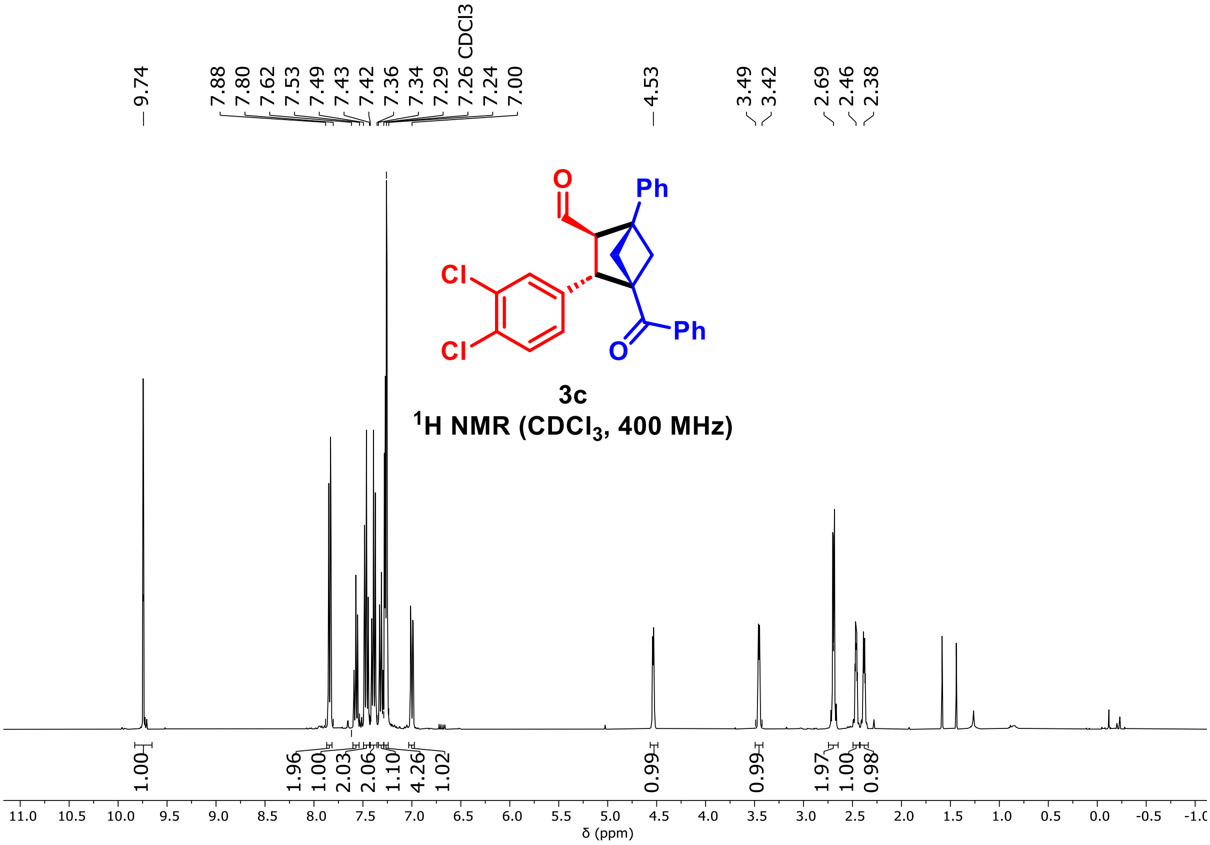

9.74
7.88
7.80
7.62
7.53
7.49
7.43
7.42
7.36
7.34
7.29
7.26 CDCl3
7.24
7.00
4.53
3.49
3.42
2.69
2.46
2.38
O
Ph
Cl
Cl
O
Ph
3c
1H NMR (CDCl3, 400 MHz)
1.00
1.96
1.00
2.03
2.06
1.10
4.26
1.02
0.99
0.99
1.97
1.00
0.98
δ (ppm)

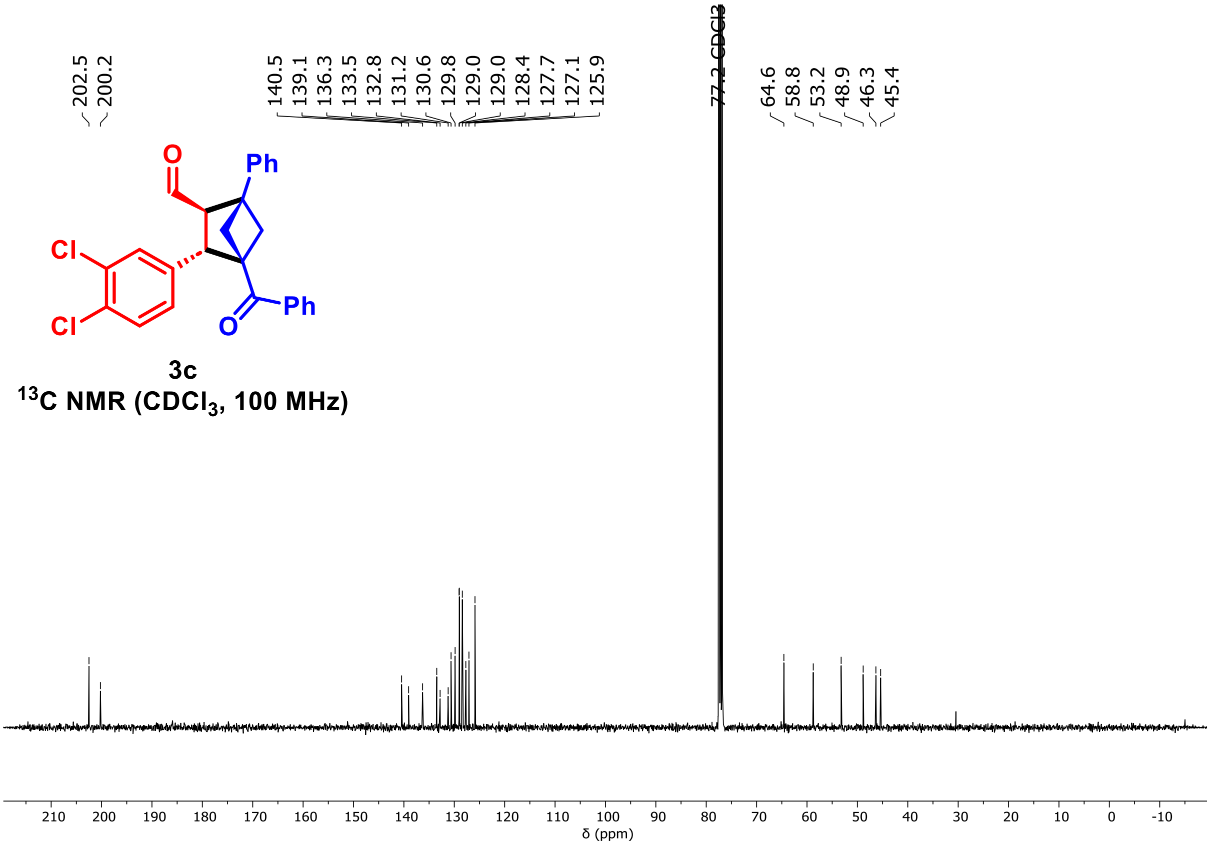

202.5
200.2
140.5
139.1
136.3
133.5
132.8
131.2
130.6
129.8
129.0
129.0
128.4
127.7
127.1
125.9
77.2 CDCl3
64.6
58.8
53.2
48.9
46.3
45.4
O
Ph
Cl
Cl
O
Ph
3c
13C NMR (CDCl3, 100 MHz)
δ (ppm)

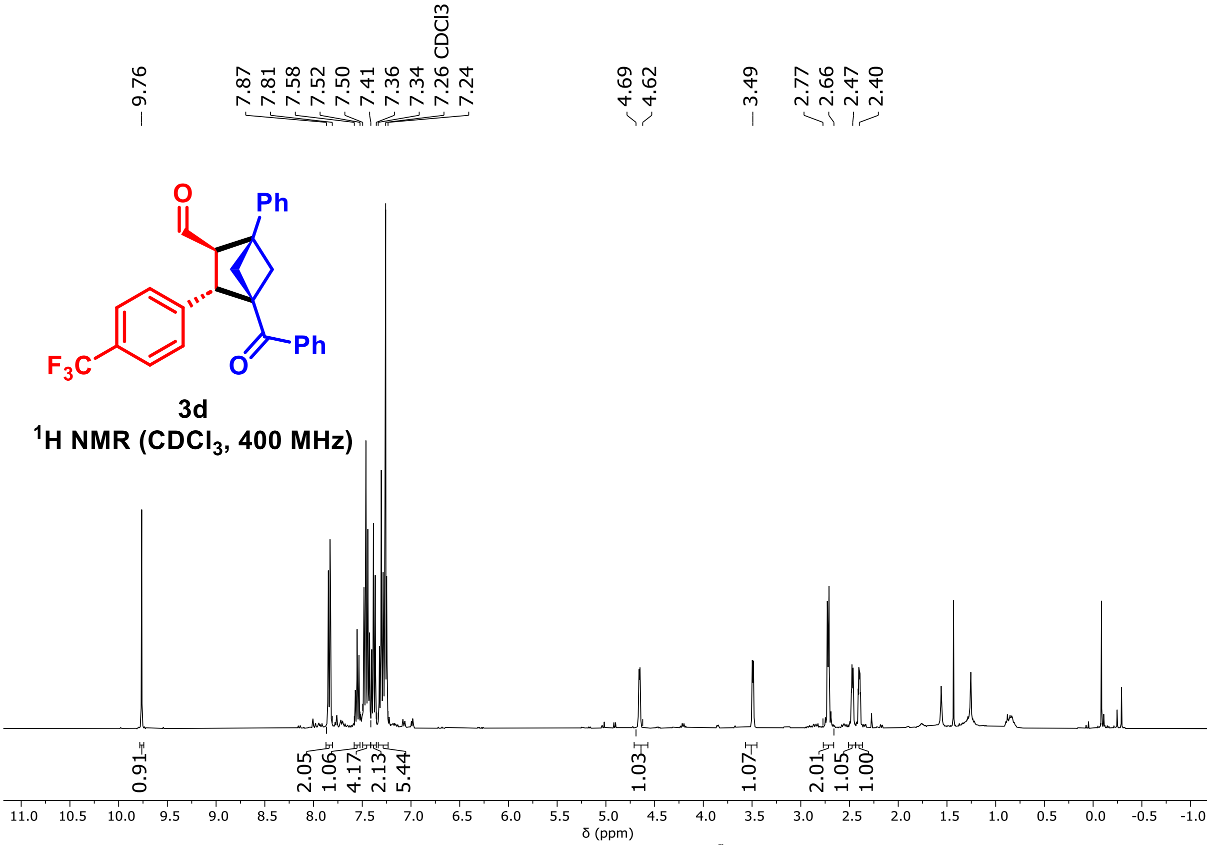
9.76
7.87
7.81
7.58
7.52
7.50
7.41
7.36
7.34
7.26 CDCl3
7.24
4.69
4.62
3.49
2.77
2.66
2.47
2.40
O
Ph
F_3C
O
Ph
3d
^1H NMR ($CDCl_3$, 400 MHz)
0.91
2.05
1.06
4.17
2.13
5.44
1.03
1.07
2.01
1.05
1.00
11.0 10.5 10.0 9.5 9.0 8.5 8.0 7.5 7.0 6.5 6.0 5.5 5.0 4.5 4.0 3.5 3.0 2.5 2.0 1.5 1.0 0.5 0.0 -0.5 -1.0
δ (ppm)

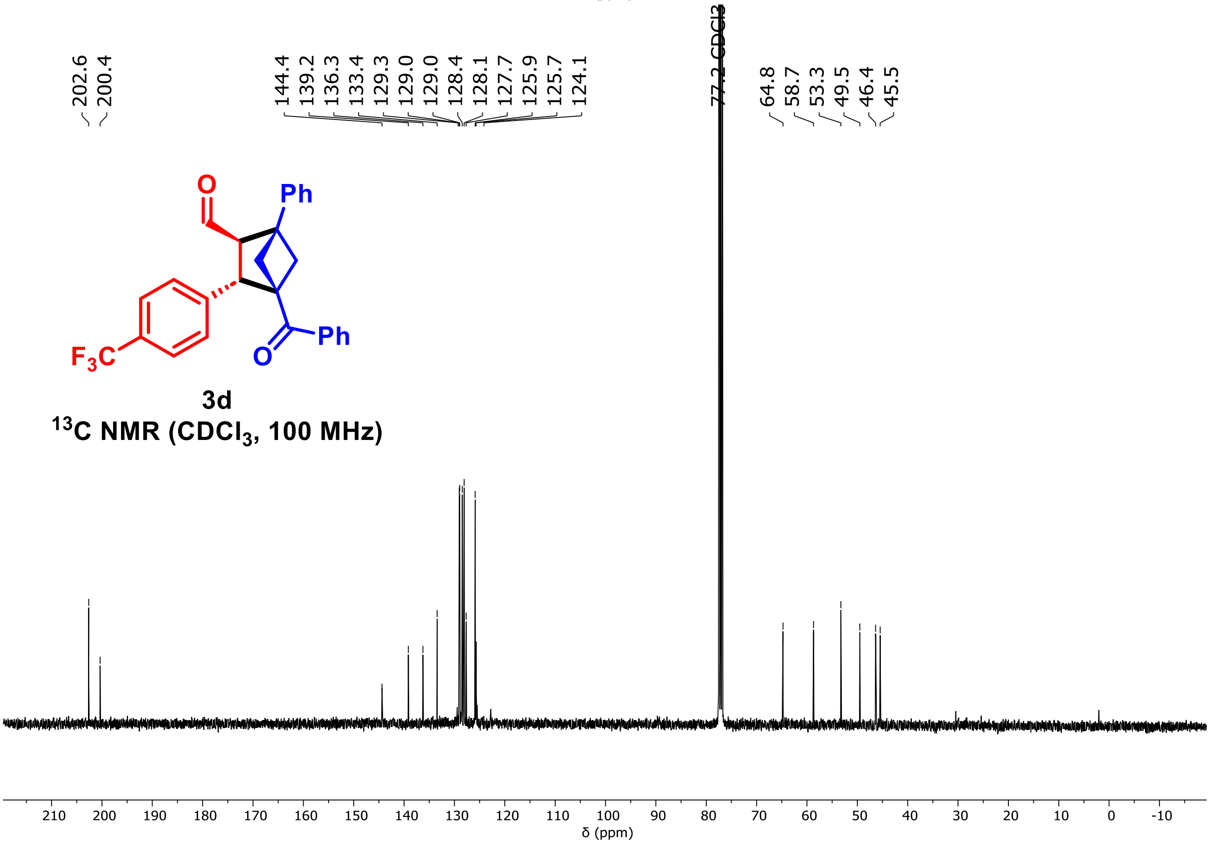
202.6
200.4
144.4
139.2
136.3
133.4
129.3
129.0
129.0
128.4
128.1
127.7
125.9
125.7
124.1
77.2 CDCl3
64.8
58.7
53.3
49.5
46.4
45.5
O
Ph
F_3C
O
Ph
3d
^{13}C NMR ($CDCl_3$, 100 MHz)
210 200 190 180 170 160 150 140 130 120 110 100 90 80 70 60 50 40 30 20 10 0 -10
δ (ppm)

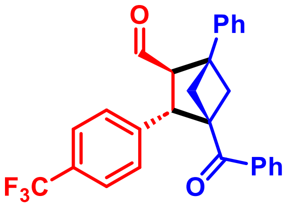

3d

[1]F NMR ($CDCl_3$, 376 MHz)

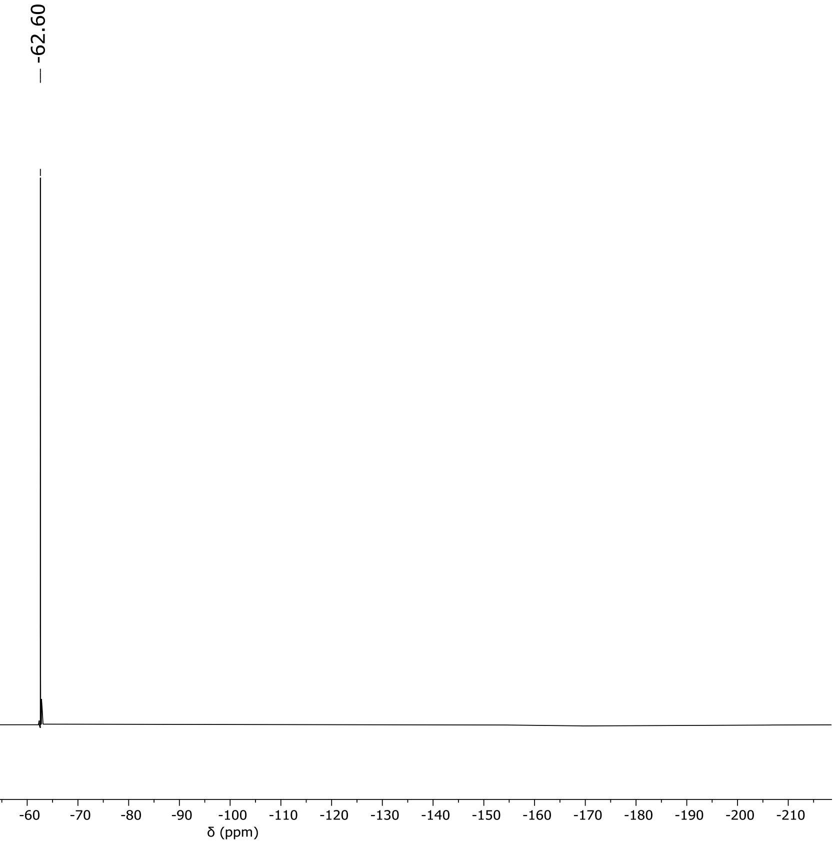

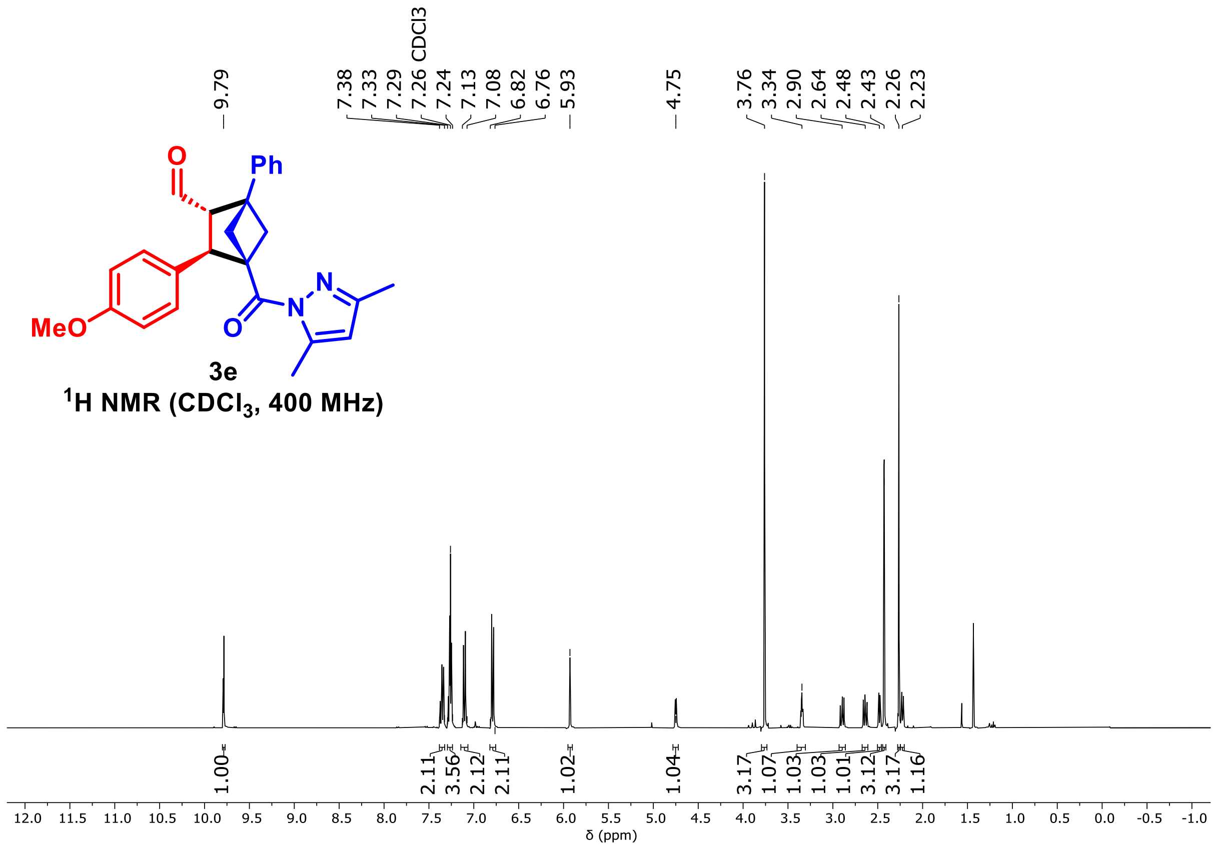
9.79
7.38
7.33
7.29
7.26 CDCl3
7.24
7.13
7.08
6.82
6.76
5.93
4.75
3.76
3.34
2.90
2.64
2.48
2.43
2.26
2.23
O
Ph
MeO
N
N
O
3e
¹H NMR (CDCl₃, 400 MHz)
1.00
2.11
3.56
2.12
2.11
1.02
1.04
3.17
1.07
1.03
1.03
1.01
3.12
3.17
1.16
δ (ppm)

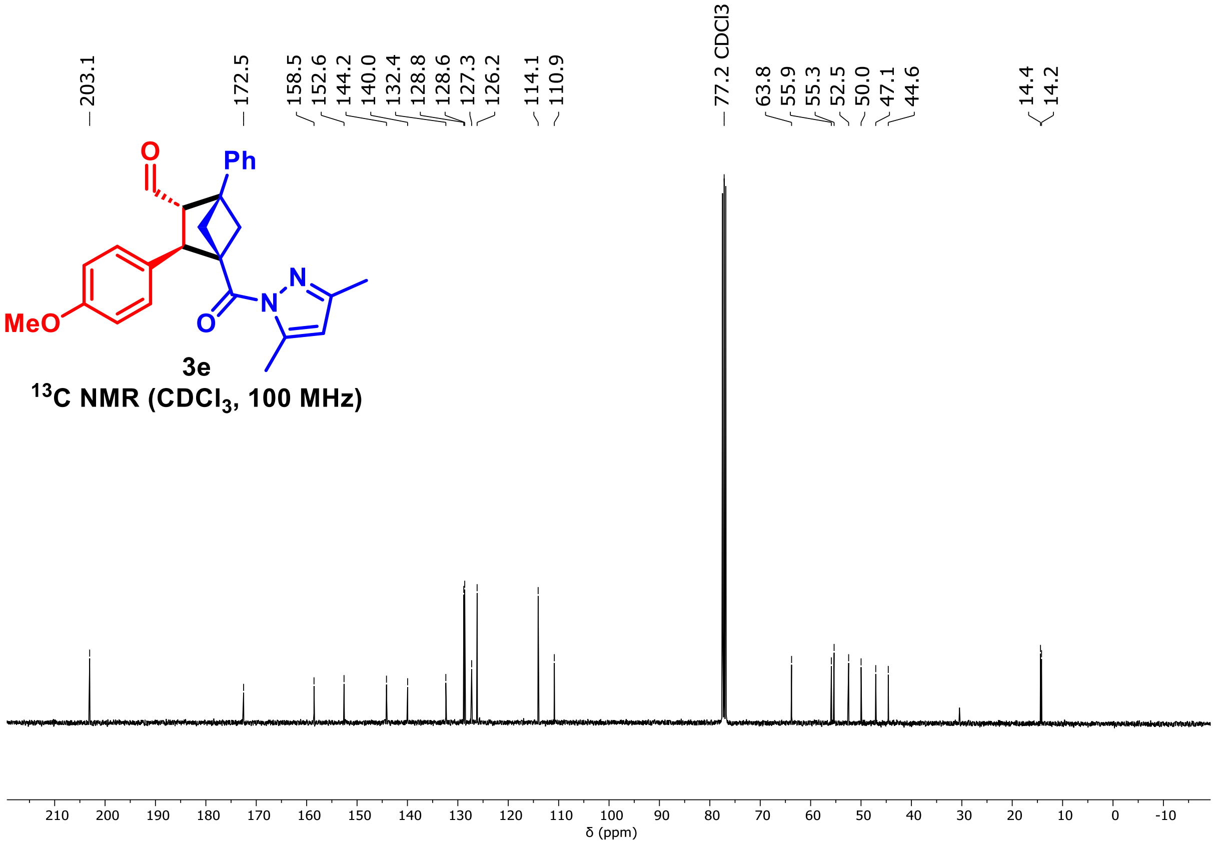
203.1
172.5
158.5
152.6
144.2
140.0
132.4
128.8
128.6
127.3
126.2
114.1
110.9
77.2 CDCl3
63.8
55.9
55.3
52.5
50.0
47.1
44.6
14.4
14.2
O
Ph
MeO
N
N
O
3e
¹³C NMR (CDCl₃, 100 MHz)
δ (ppm)

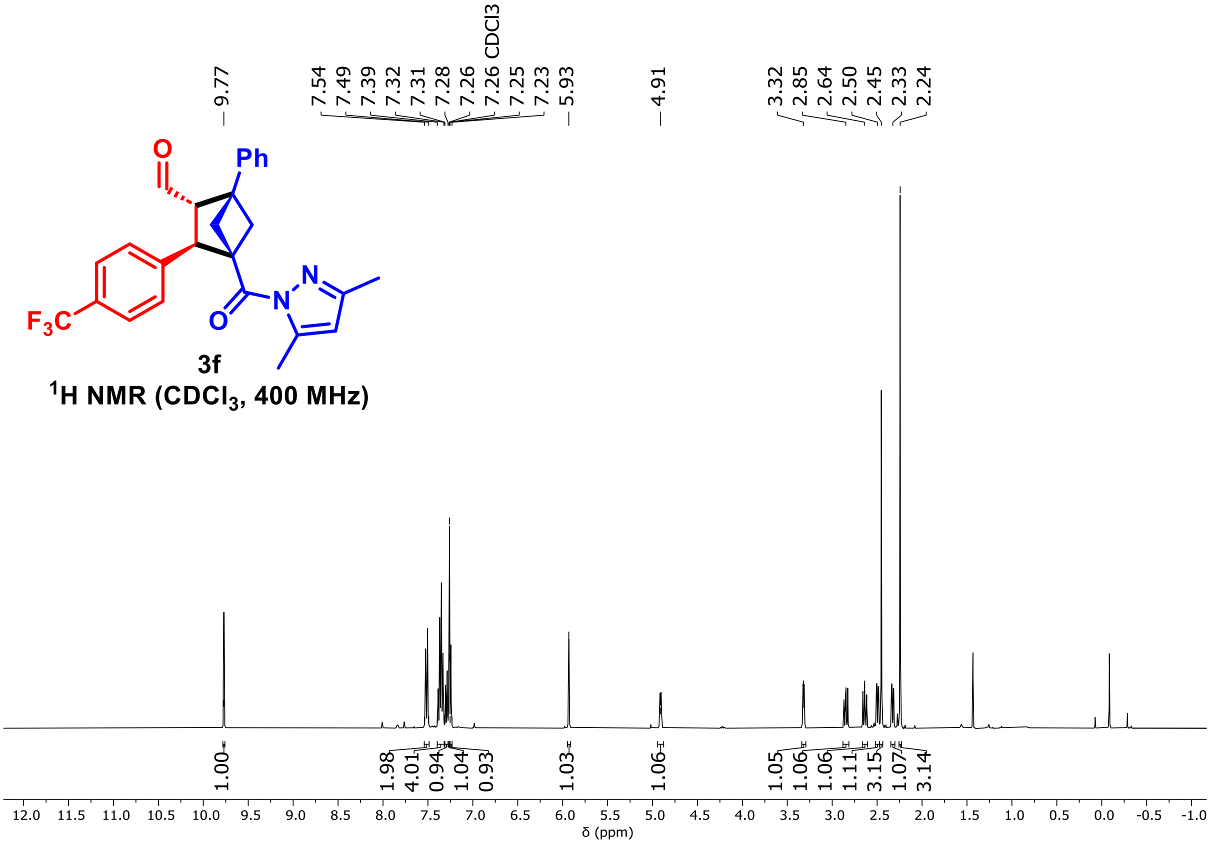

9.77
7.54
7.49
7.39
7.32
7.31
7.28
7.26
7.26 CDCl3
7.25
7.23
5.93
4.91
3.32
2.85
2.64
2.50
2.45
2.33
2.24
O
Ph
F3C
N
N
O
3f
1H NMR (CDCl3, 400 MHz)
1.00
1.98
4.01
0.94
1.04
0.93
1.03
1.06
1.05
1.06
1.06
1.11
3.15
1.07
3.14
12.0 11.5 11.0 10.5 10.0 9.5 9.0 8.5 8.0 7.5 7.0 6.5 6.0 5.5 5.0 4.5 4.0 3.5 3.0 2.5 2.0 1.5 1.0 0.5 0.0 -0.5 -1.0
δ (ppm)

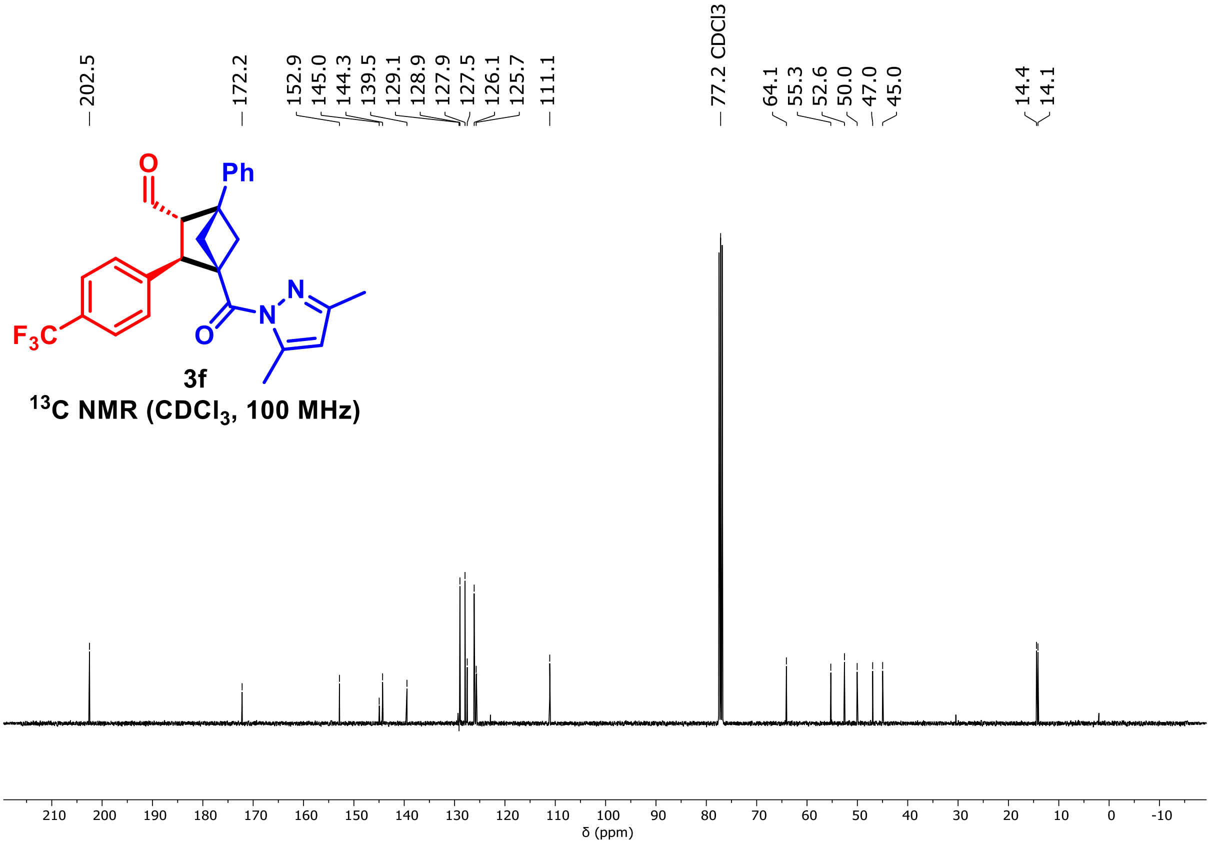

202.5
172.2
152.9
145.0
144.3
139.5
129.1
128.9
127.9
127.5
126.1
125.7
111.1
77.2 CDCl3
64.1
55.3
52.6
50.0
47.0
45.0
14.4
14.1
O
Ph
F3C
N
N
O
3f
13C NMR (CDCl3, 100 MHz)
210 200 190 180 170 160 150 140 130 120 110 100 90 80 70 60 50 40 30 20 10 0 -10
δ (ppm)

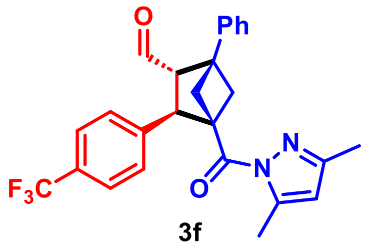

3f

^{19}F NMR ($CDCl_3$, 376 MHz)

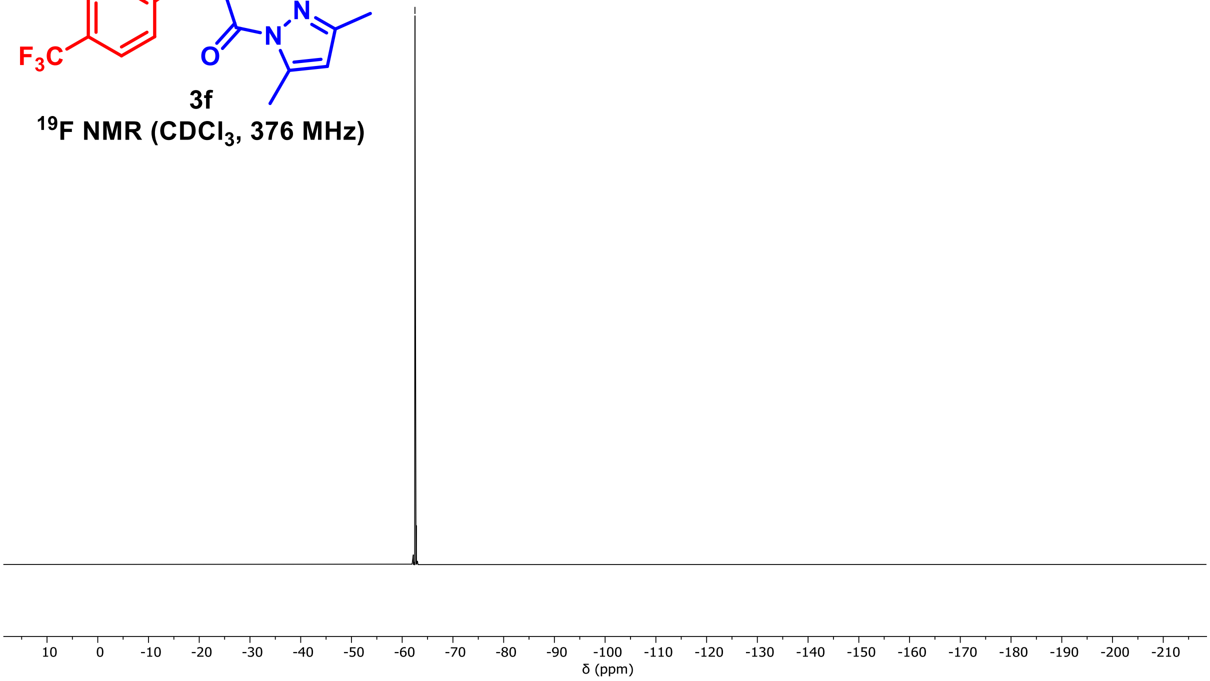

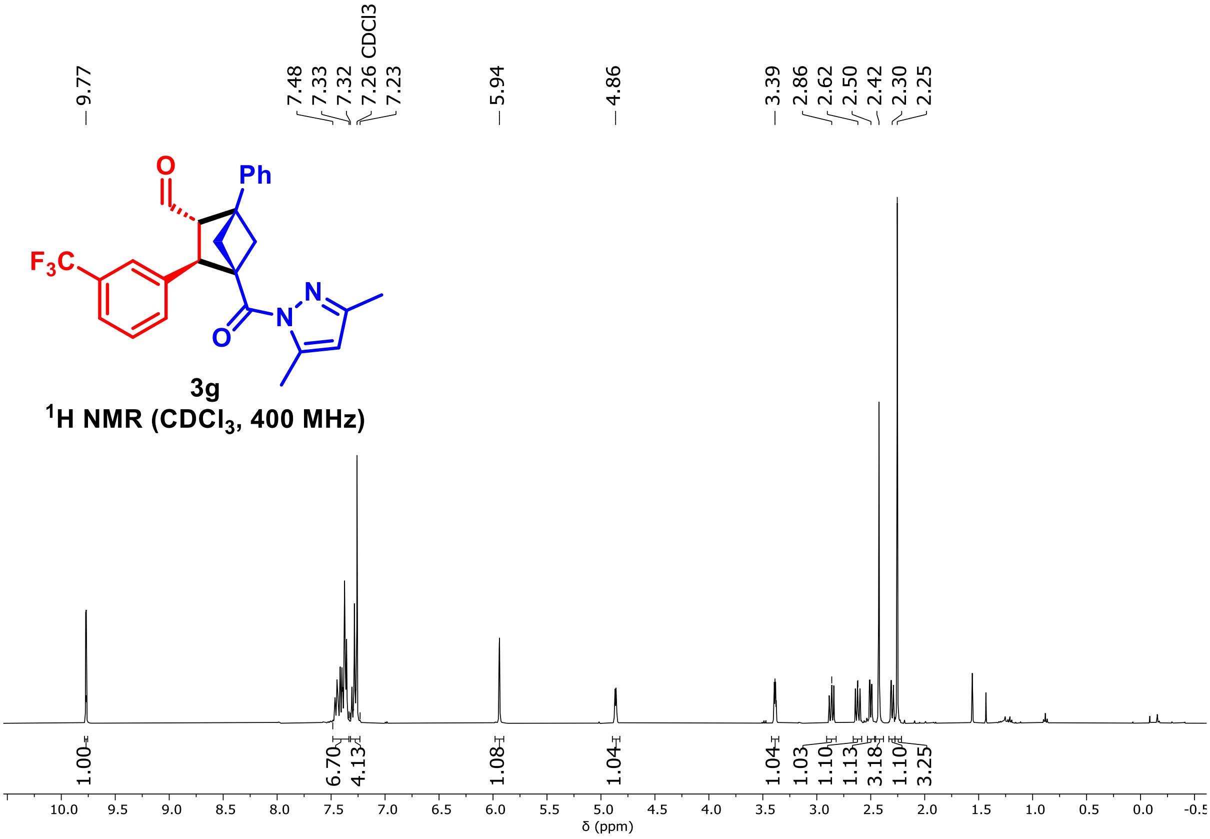
9.77
7.48
7.33
7.32
7.26 CDCl3
7.23
5.94
4.86
3.39
2.86
2.62
2.50
2.42
2.30
2.25
Ph
O
F3C
N
N
O
3g
1H NMR (CDCl3, 400 MHz)
1.00
6.70
4.13
1.08
1.04
1.04
1.03
1.10
1.13
3.18
1.10
3.25
δ (ppm)

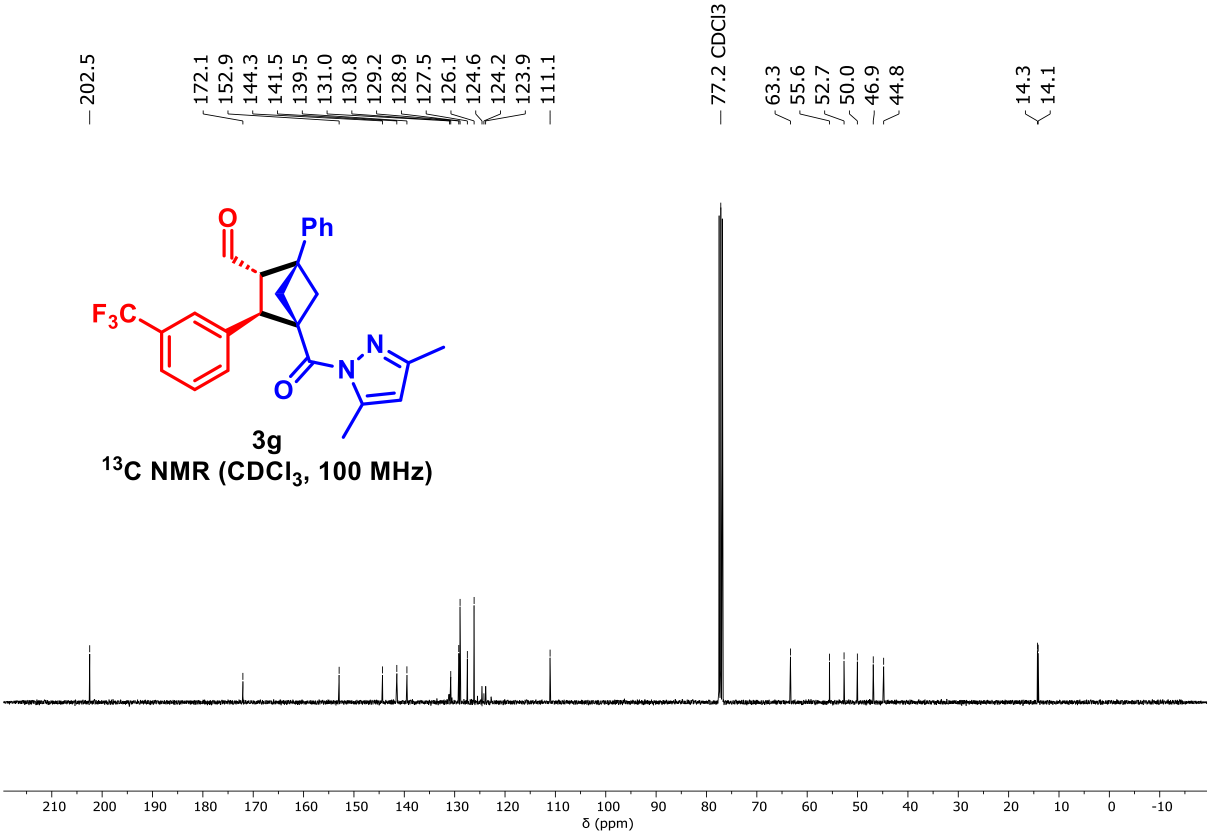
202.5
172.1
152.9
144.3
141.5
139.5
131.0
130.8
129.2
128.9
127.5
126.1
124.6
124.2
123.9
111.1
77.2 CDCl3
63.3
55.6
52.7
50.0
46.9
44.8
14.3
14.1
Ph
O
F3C
N
N
O
3g
13C NMR (CDCl3, 100 MHz)
δ (ppm)

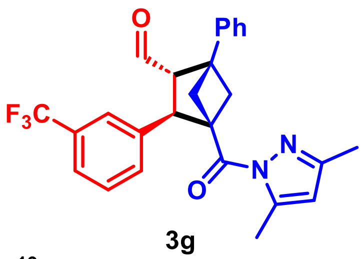

3g

^{19}F NMR ($CDCl_3$, 376 MHz)

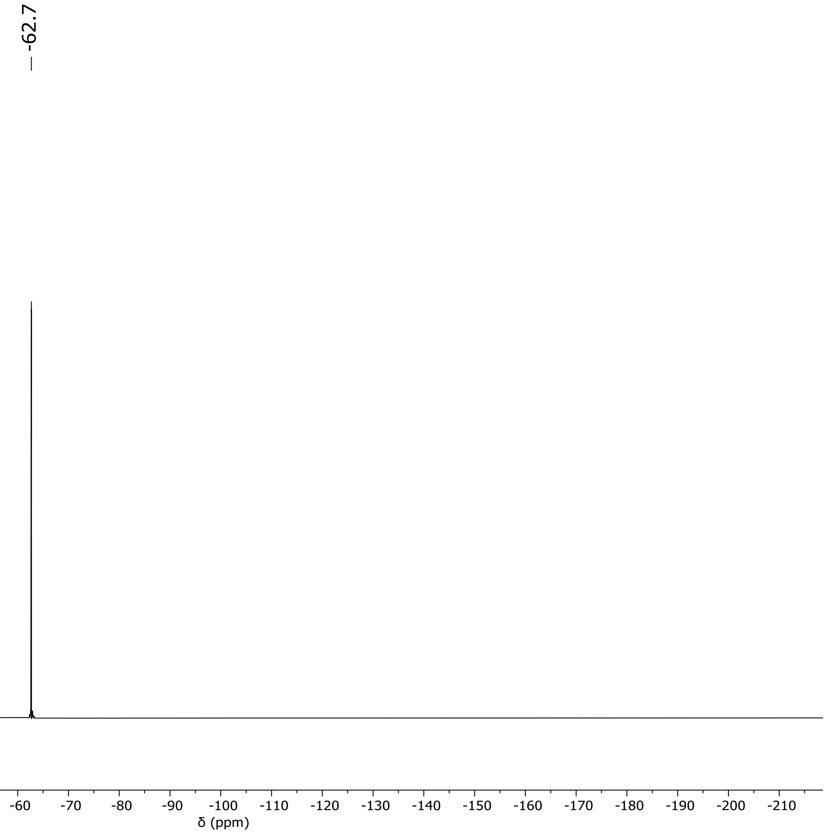

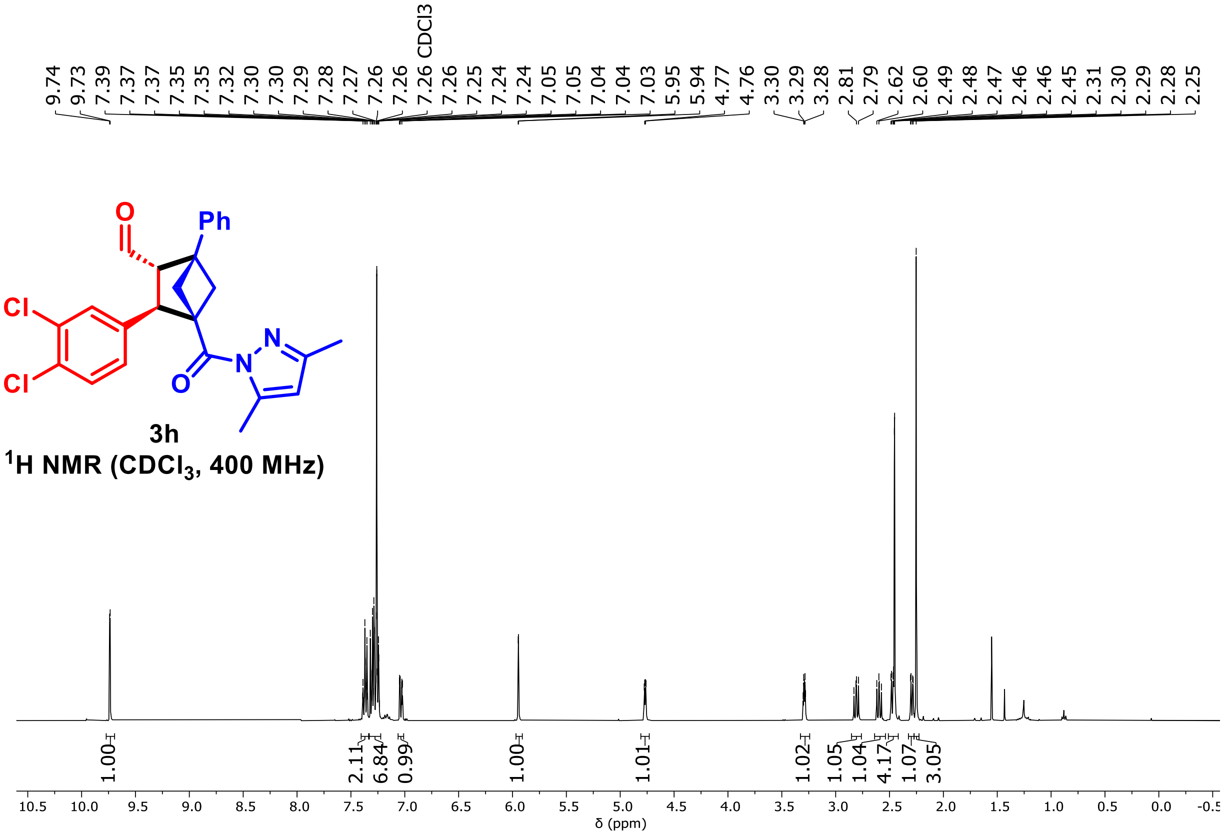
9.74 9.73 7.39 7.37 7.37 7.35 7.35 7.32 7.30 7.30 7.29 7.28 7.27 7.26 7.26 7.26 CDCl3 7.26 7.25 7.24 7.24 7.05 7.05 7.04 7.04 7.03 5.95 5.94 4.77 4.76 3.30 3.29 3.28 2.81 2.79 2.62 2.60 2.49 2.48 2.47 2.46 2.46 2.45 2.31 2.30 2.29 2.28 2.25
O
Ph
Cl
Cl
N
N
O
3h
1H NMR (CDCl3, 400 MHz)
1.00 2.11 6.84 0.99 1.00 1.01 1.02 1.05 1.04 4.17 1.07 3.05
δ (ppm)

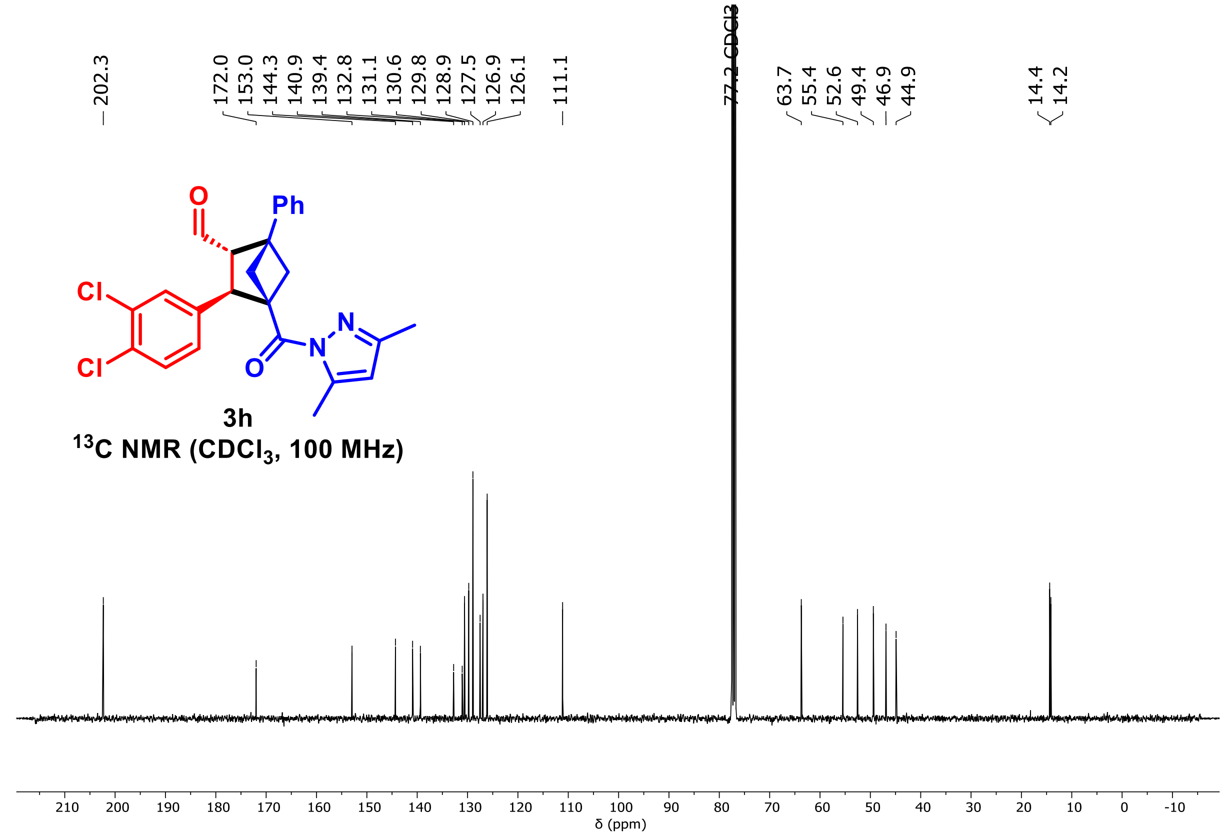
202.3
172.0 153.0 144.3 140.9 139.4 132.8 131.1 130.6 129.8 128.9 127.5 126.9 126.1
111.1
77.2 CDCl3
63.7 55.4 52.6 49.4 46.9 44.9
14.4 14.2
O
Ph
Cl
Cl
N
N
O
3h
13C NMR (CDCl3, 100 MHz)
δ (ppm)

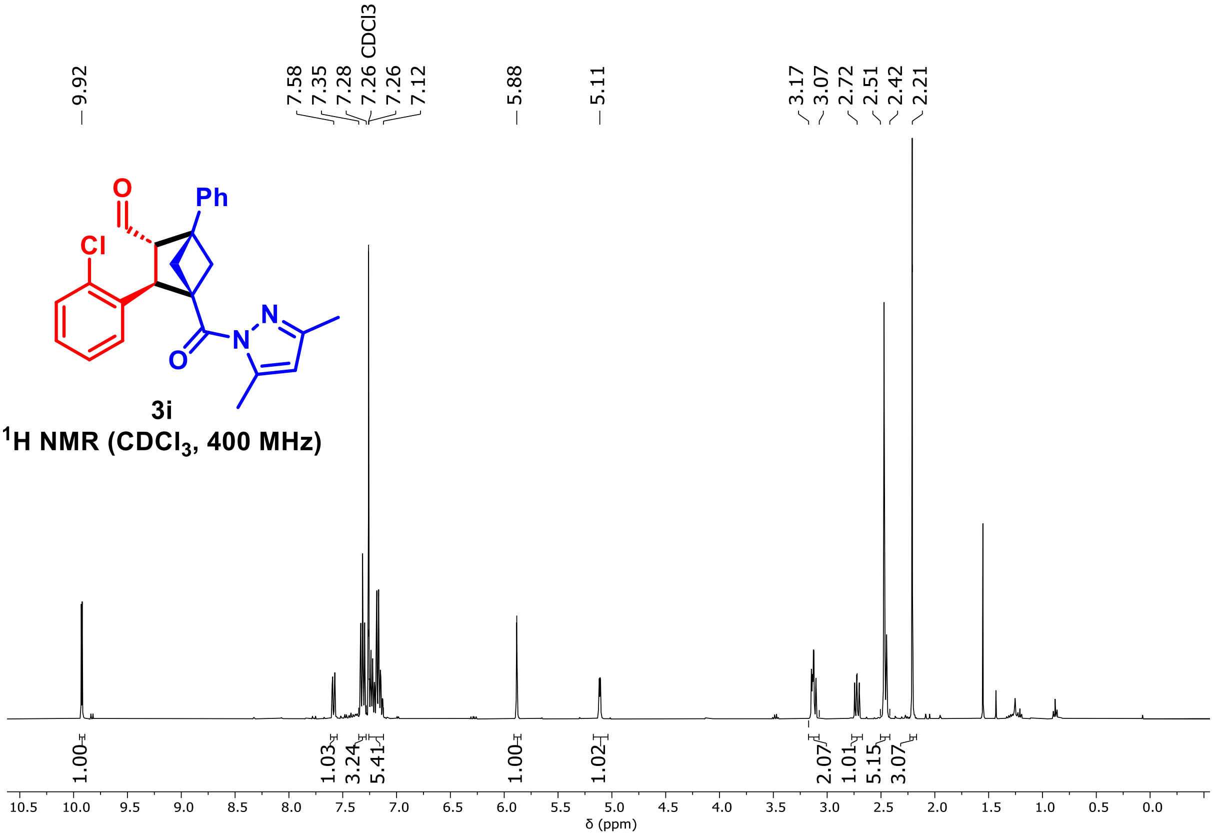
3i
^{1}H NMR (CDCl$_3$, 400 MHz)
9.92
7.58
7.35
7.28
7.26 CDCl3
7.26
7.12
5.88
5.11
3.17
3.07
2.72
2.51
2.42
2.21
1.00
1.03
3.24
5.41
1.00
1.02
2.07
1.01
5.15
3.07
δ (ppm)

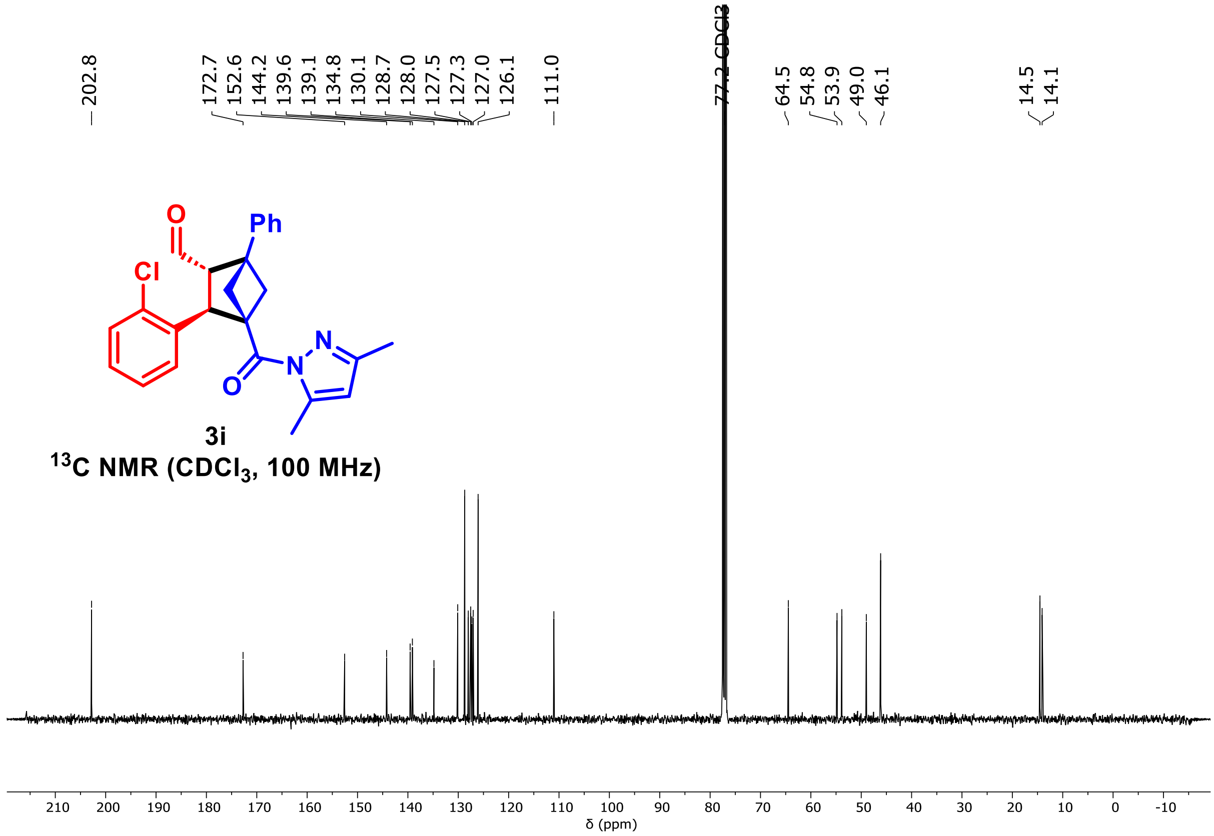
3i
^{13}C NMR (CDCl$_3$, 100 MHz)
202.8
172.7
152.6
144.2
139.6
139.1
134.8
130.1
128.7
128.0
127.5
127.3
127.0
126.1
111.0
77.2 CDCl3
64.5
54.8
53.9
49.0
46.1
14.5
14.1
δ (ppm)

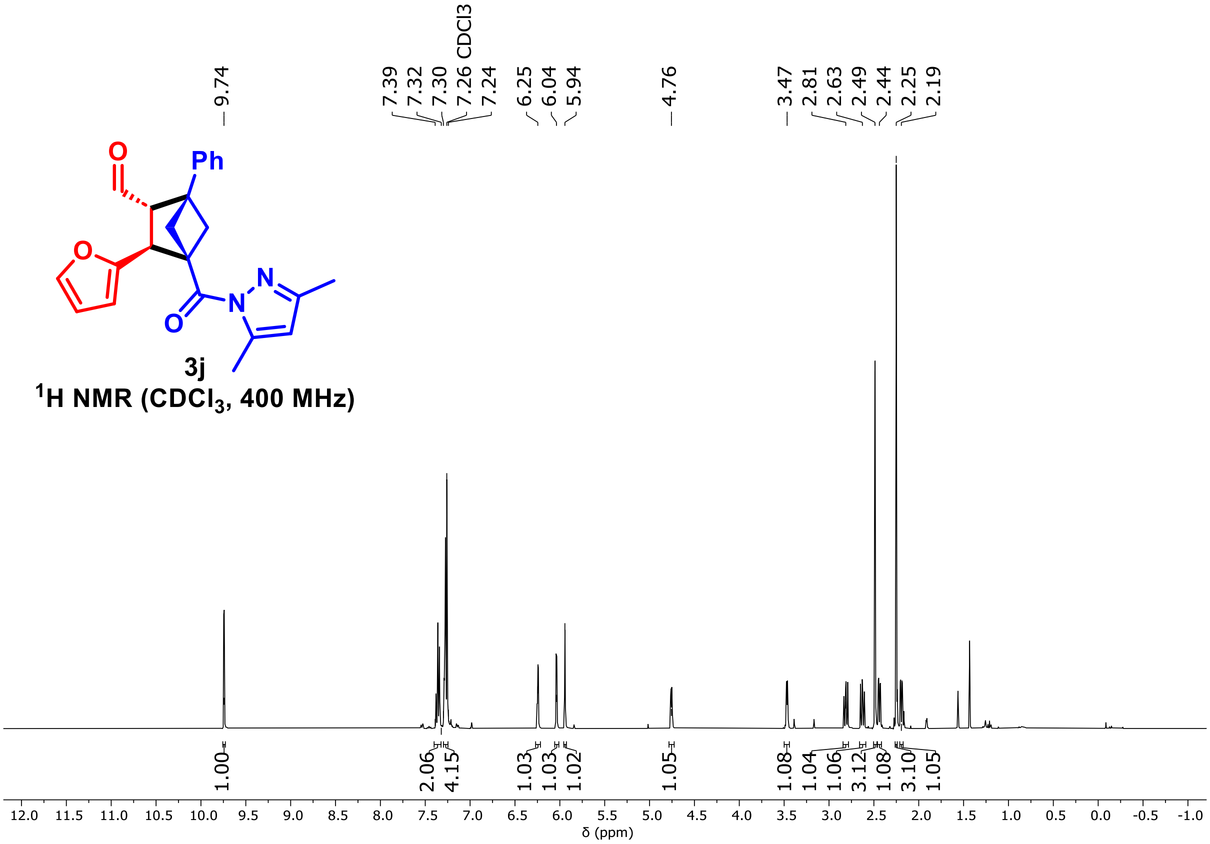

9.74
7.39
7.32
7.30
7.26 CDCl3
7.24
6.25
6.04
5.94
4.76
3.47
2.81
2.63
2.49
2.44
2.25
2.19
O
Ph
O
N
N
O
3j
^{1}H NMR (CDCl$_3$, 400 MHz)
1.00
2.06
4.15
1.03
1.03
1.02
1.05
1.08
1.04
1.06
3.12
1.08
3.10
1.05
12.0 11.5 11.0 10.5 10.0 9.5 9.0 8.5 8.0 7.5 7.0 6.5 6.0 5.5 5.0 4.5 4.0 3.5 3.0 2.5 2.0 1.5 1.0 0.5 0.0 -0.5 -1.0
δ (ppm)

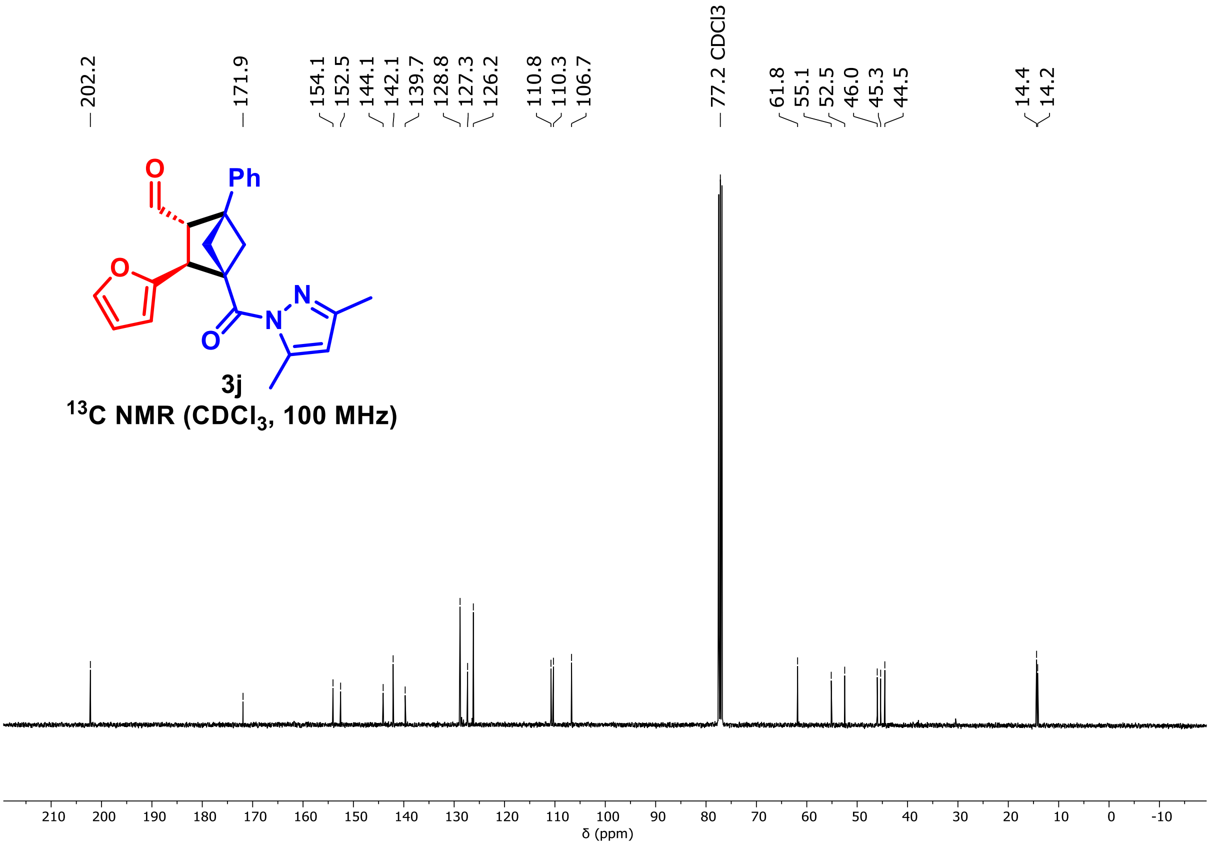

202.2
171.9
154.1
152.5
144.1
142.1
139.7
128.8
127.3
126.2
110.8
110.3
106.7
77.2 CDCl3
61.8
55.1
52.5
46.0
45.3
44.5
14.4
14.2
O
Ph
O
N
N
O
3j
^{13}C NMR (CDCl$_3$, 100 MHz)
210 200 190 180 170 160 150 140 130 120 110 100 90 80 70 60 50 40 30 20 10 0 -10
δ (ppm)

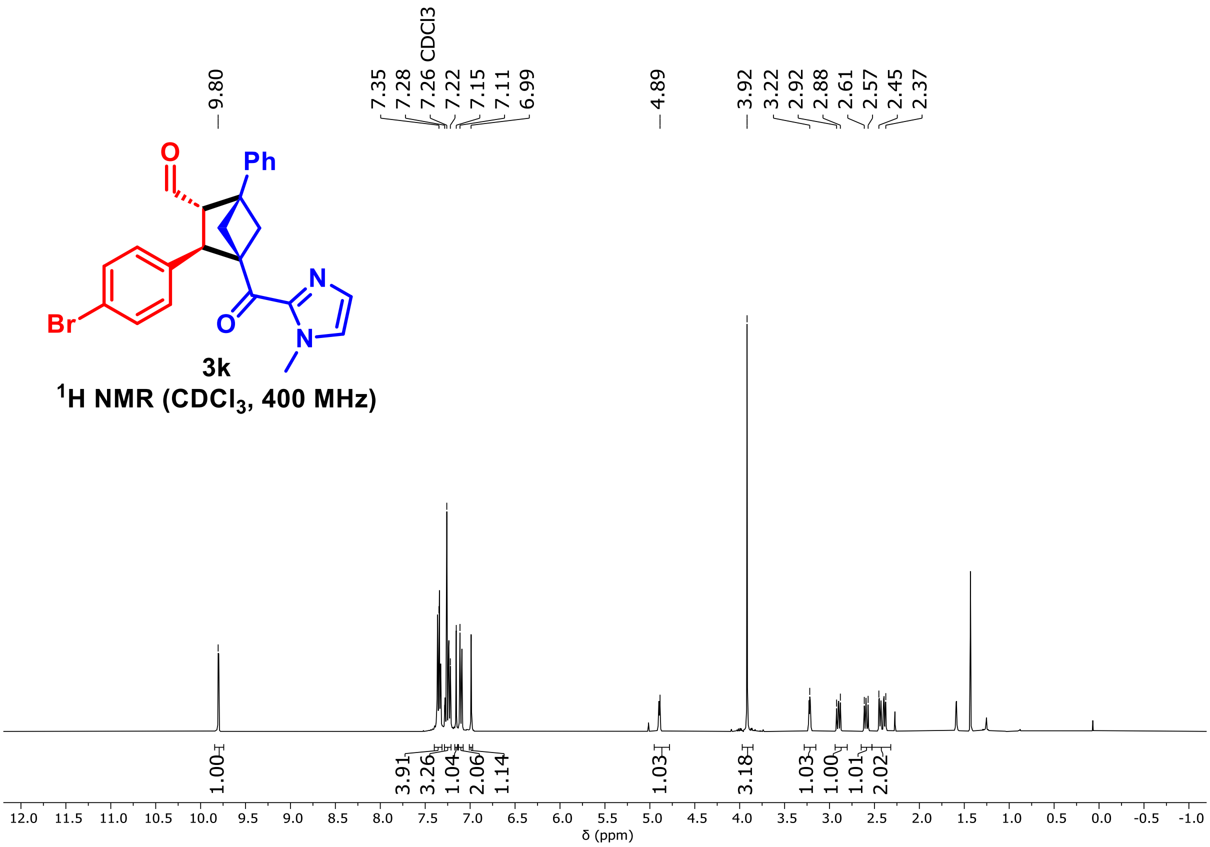

9.80
7.35
7.28
7.26 CDCl3
7.22
7.15
7.11
6.99
4.89
3.92
3.22
2.92
2.88
2.61
2.57
2.45
2.37
Ph
O
Br
N
O
N
3k
1H NMR (CDCl3, 400 MHz)
1.00
3.91
3.26
1.04
2.06
1.14
1.03
3.18
1.03
1.00
1.01
2.02
δ (ppm)

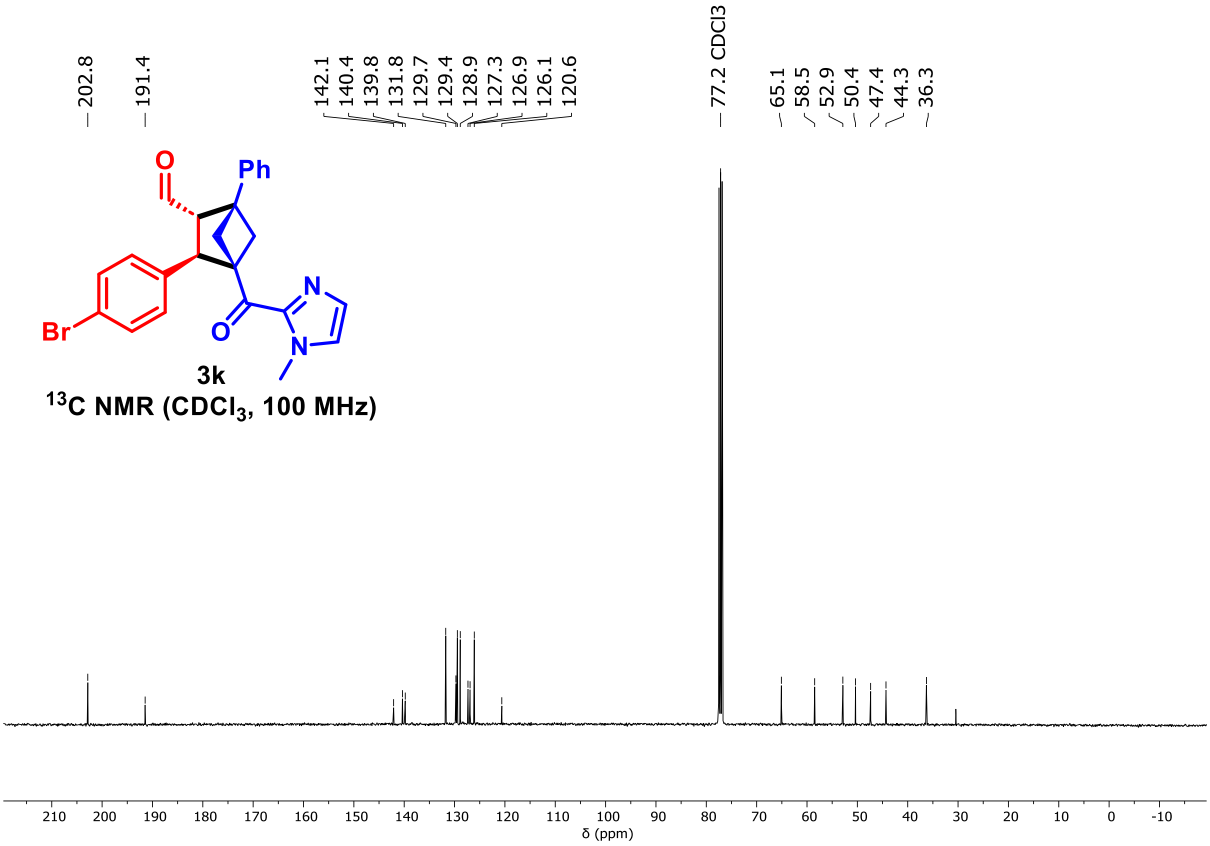

202.8
191.4
142.1
140.4
139.8
131.8
129.7
129.4
128.9
127.3
126.9
126.1
120.6
77.2 CDCl3
65.1
58.5
52.9
50.4
47.4
44.3
36.3
Ph
O
Br
N
O
N
3k
13C NMR (CDCl3, 100 MHz)
δ (ppm)

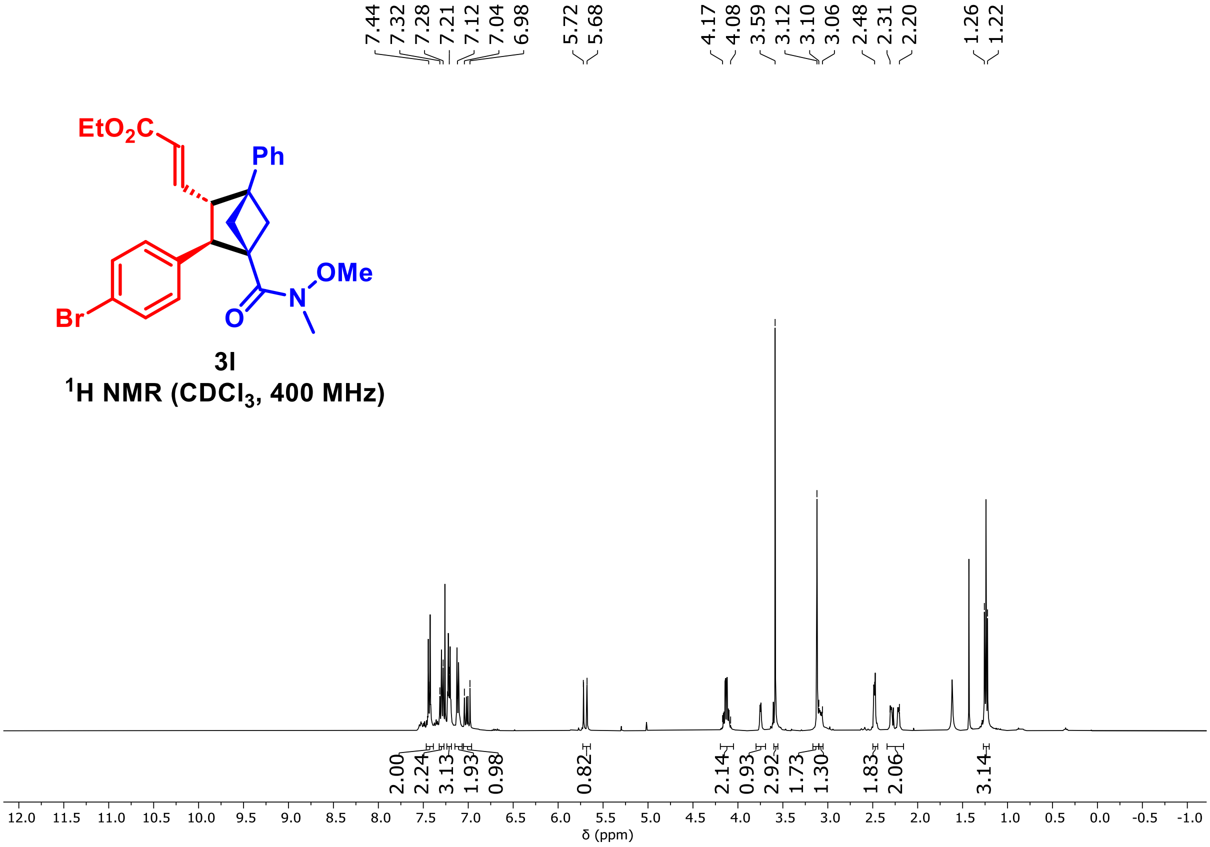
EtO2C
Ph
OMe
N
O
Br
3l
1H NMR (CDCl3, 400 MHz)
7.44
7.32
7.28
7.21
7.12
7.04
6.98
5.72
5.68
4.17
4.08
3.59
3.12
3.10
3.06
2.48
2.31
2.20
1.26
1.22
2.00
2.24
3.13
1.93
0.98
0.82
2.14
0.93
2.92
1.73
1.30
1.83
2.06
3.14
δ (ppm)

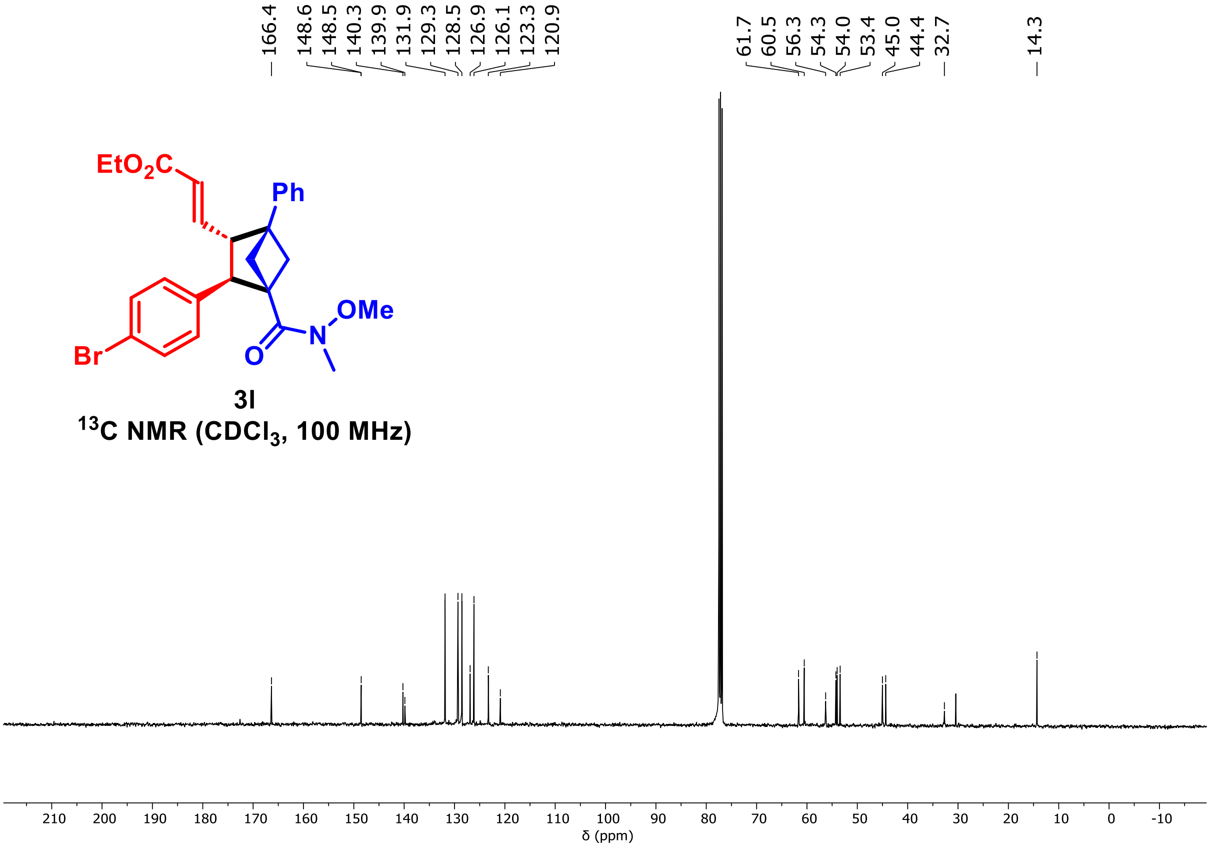
EtO2C
Ph
OMe
N
O
Br
3l
13C NMR (CDCl3, 100 MHz)
166.4
148.6
148.5
140.3
139.9
131.9
129.3
128.5
126.9
126.1
123.3
120.9
61.7
60.5
56.3
54.3
54.0
53.4
45.0
44.4
32.7
14.3
δ (ppm)

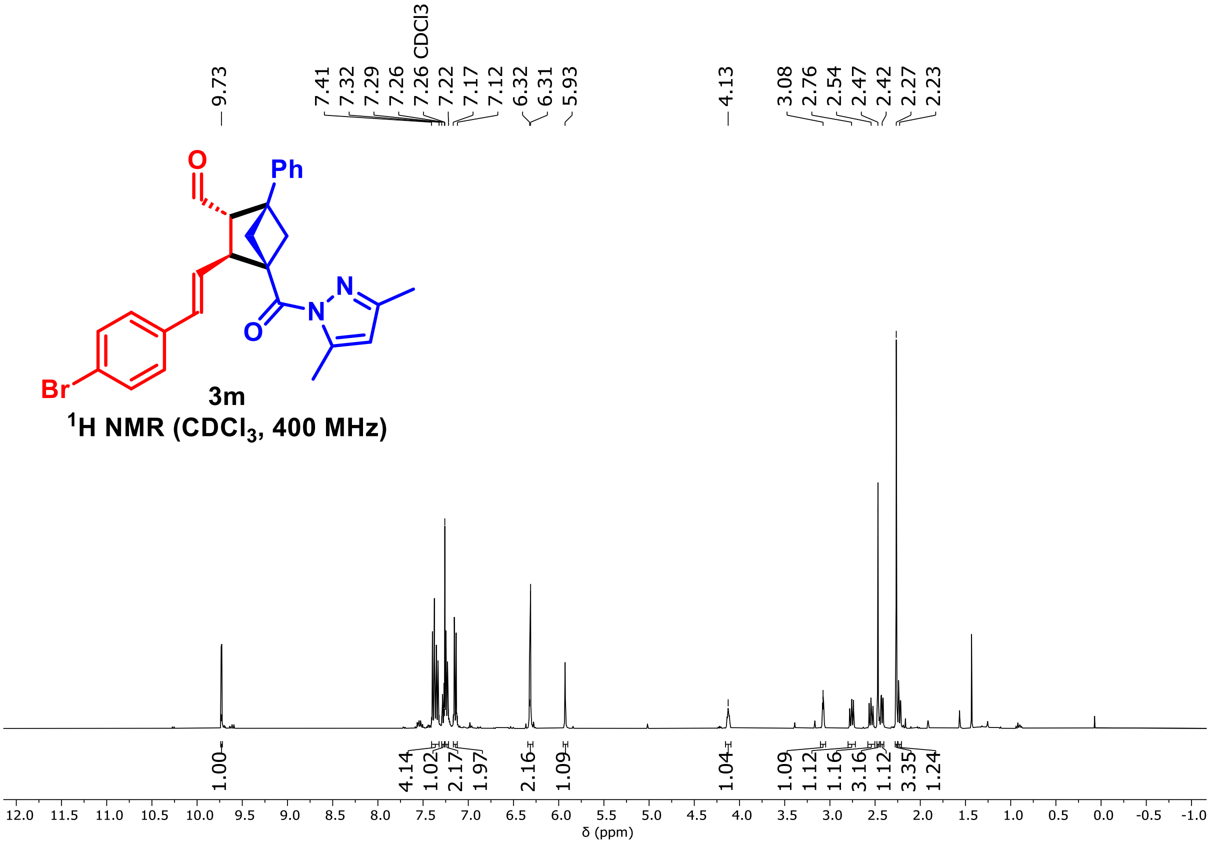
9.73
7.41 7.32 7.29 7.26 7.26 CDCl3 7.22 7.17 7.12
6.32 6.31 5.93
4.13
3.08 2.76 2.54 2.47 2.42 2.27 2.23
O
Ph
N
N
O
Br
3m
1H NMR (CDCl3, 400 MHz)
1.00
4.14 1.02 2.17 1.97
2.16 1.09
1.04
1.09 1.12 1.16 3.16 1.12 3.35 1.24
δ (ppm)

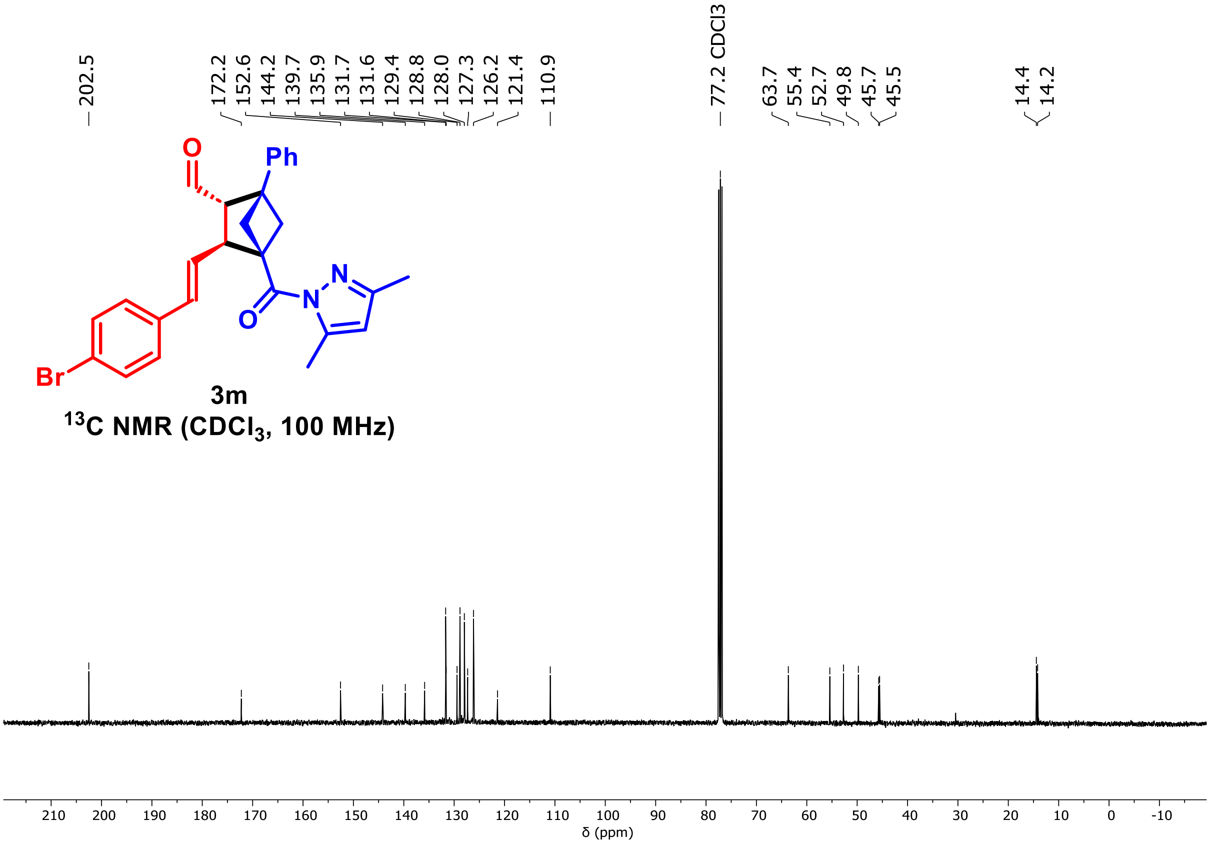
202.5
172.2 152.6 144.2 139.7 135.9 131.7 131.6 129.4 128.8 128.0 127.3 126.2 121.4
110.9
77.2 CDCl3
63.7 55.4 52.7 49.8 45.7 45.5
14.4 14.2
O
Ph
N
N
O
Br
3m
13C NMR (CDCl3, 100 MHz)
δ (ppm)

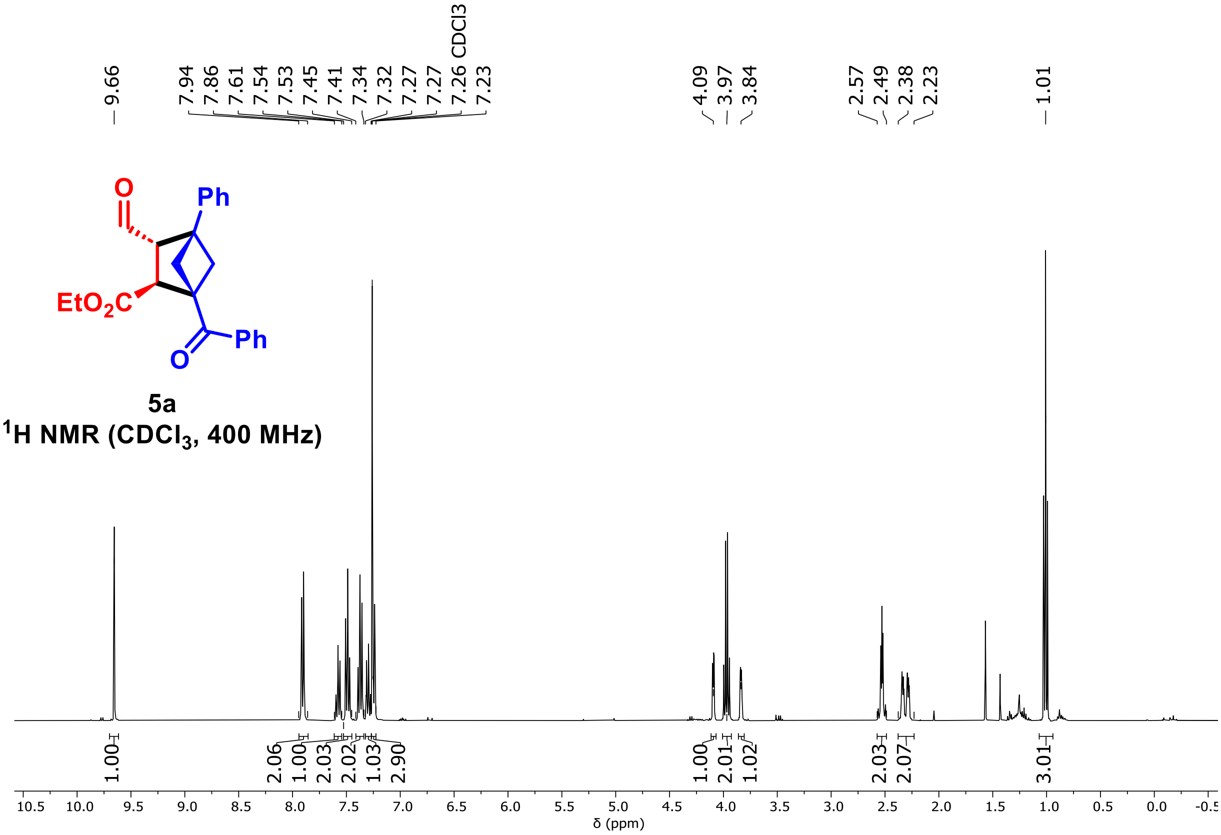
9.66
7.94 7.86 7.61 7.54 7.53 7.45 7.41 7.34 7.32 7.27 7.27 7.26 CDCl3 7.23
4.09 3.97 3.84
2.57 2.49 2.38 2.23
1.01
O
Ph
EtO2C
Ph
O
5a
1H NMR (CDCl3, 400 MHz)
1.00
2.06 1.00 2.03 2.02 1.03 2.90
1.00 2.01 1.02
2.03 2.07
3.01
10.5 10.0 9.5 9.0 8.5 8.0 7.5 7.0 6.5 6.0 5.5 5.0 4.5 4.0 3.5 3.0 2.5 2.0 1.5 1.0 0.5 0.0 -0.5
δ (ppm)

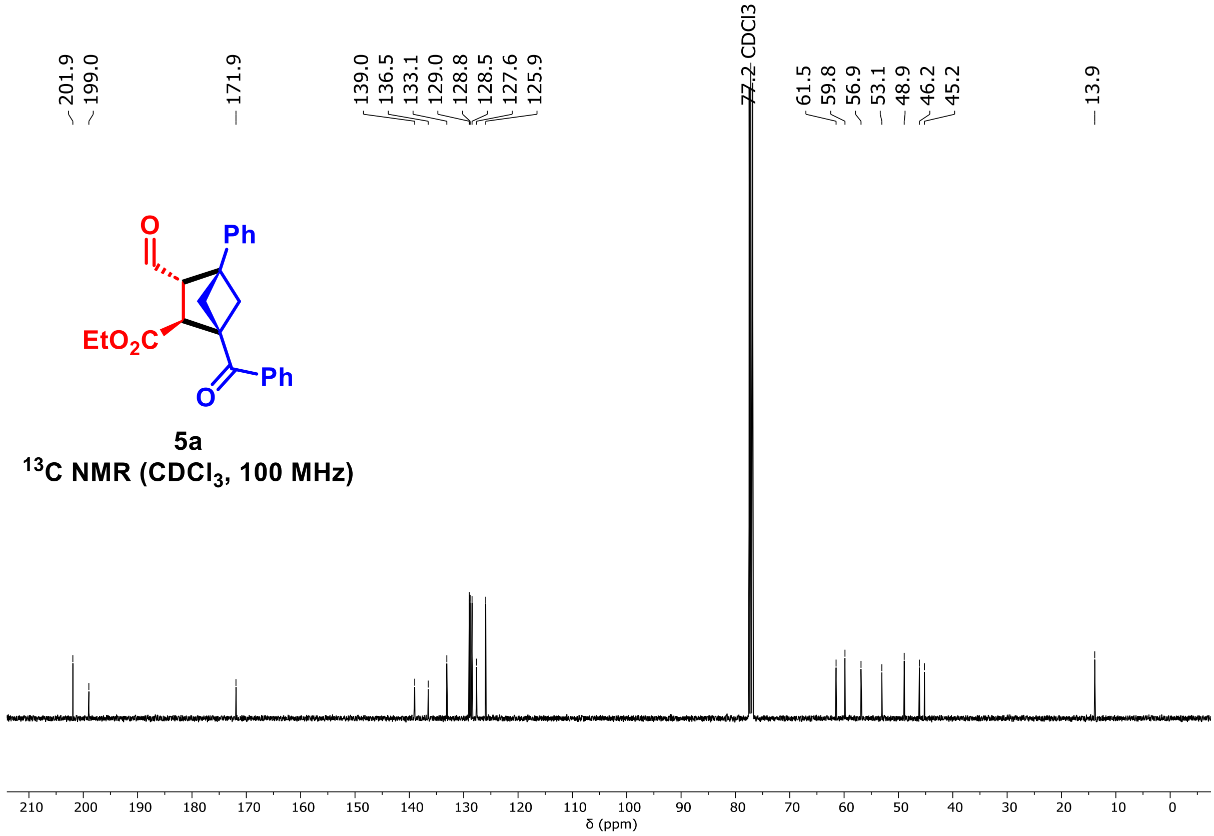
201.9 199.0
171.9
139.0 136.5 133.1 129.0 128.8 128.5 127.6 125.9
77.2 CDCl3
61.5 59.8 56.9 53.1 48.9 46.2 45.2
13.9
O
Ph
EtO2C
Ph
O
5a
13C NMR (CDCl3, 100 MHz)
210 200 190 180 170 160 150 140 130 120 110 100 90 80 70 60 50 40 30 20 10 0
δ (ppm)

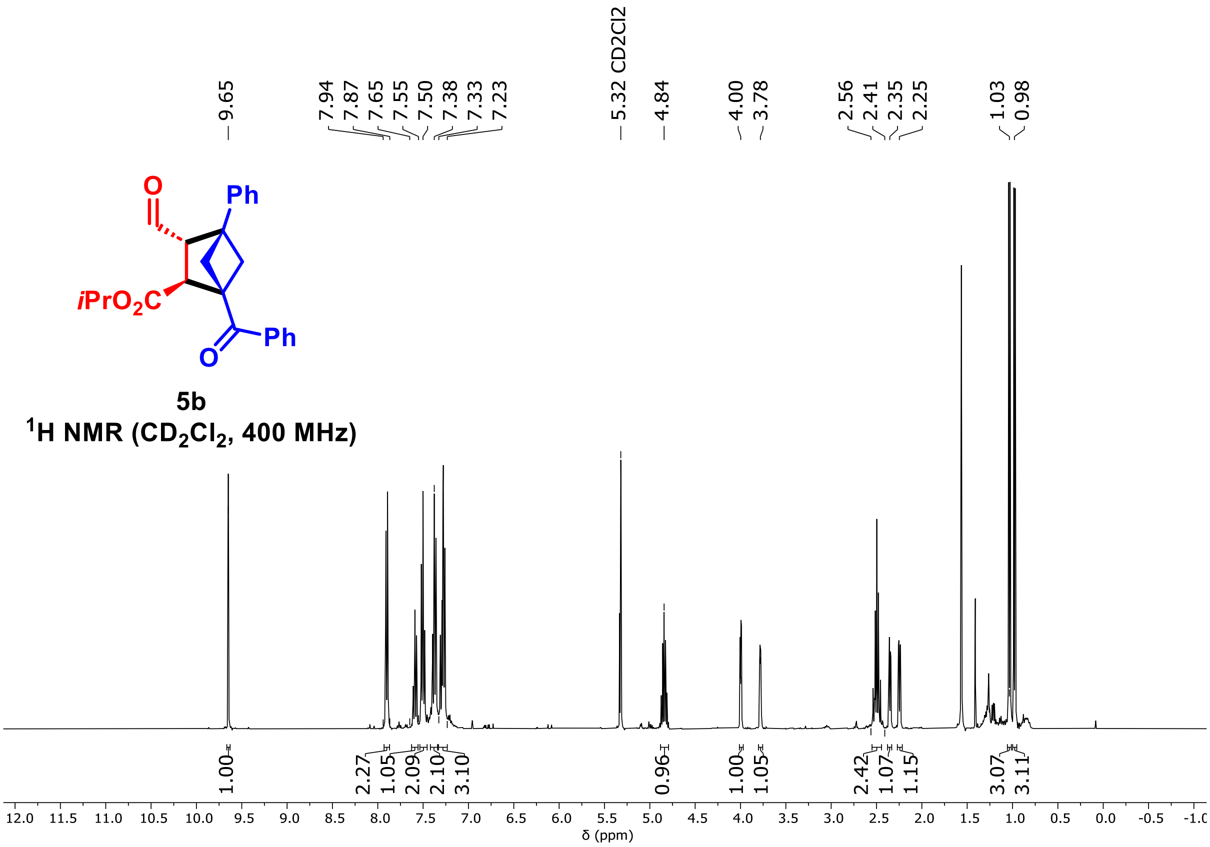

5b

^{1}H NMR (CD_2Cl_2, 400 MHz)

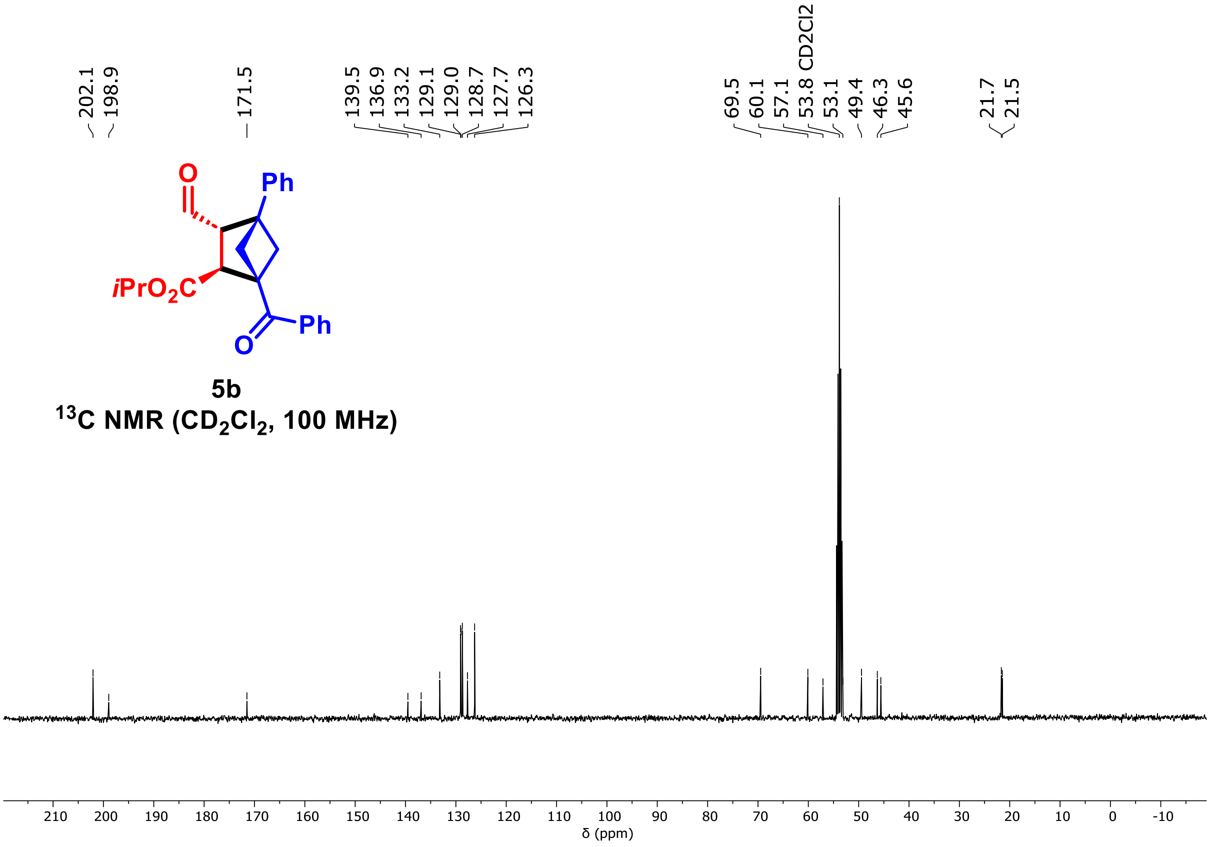

5b

^{13}C NMR (CD_2Cl_2, 100 MHz)

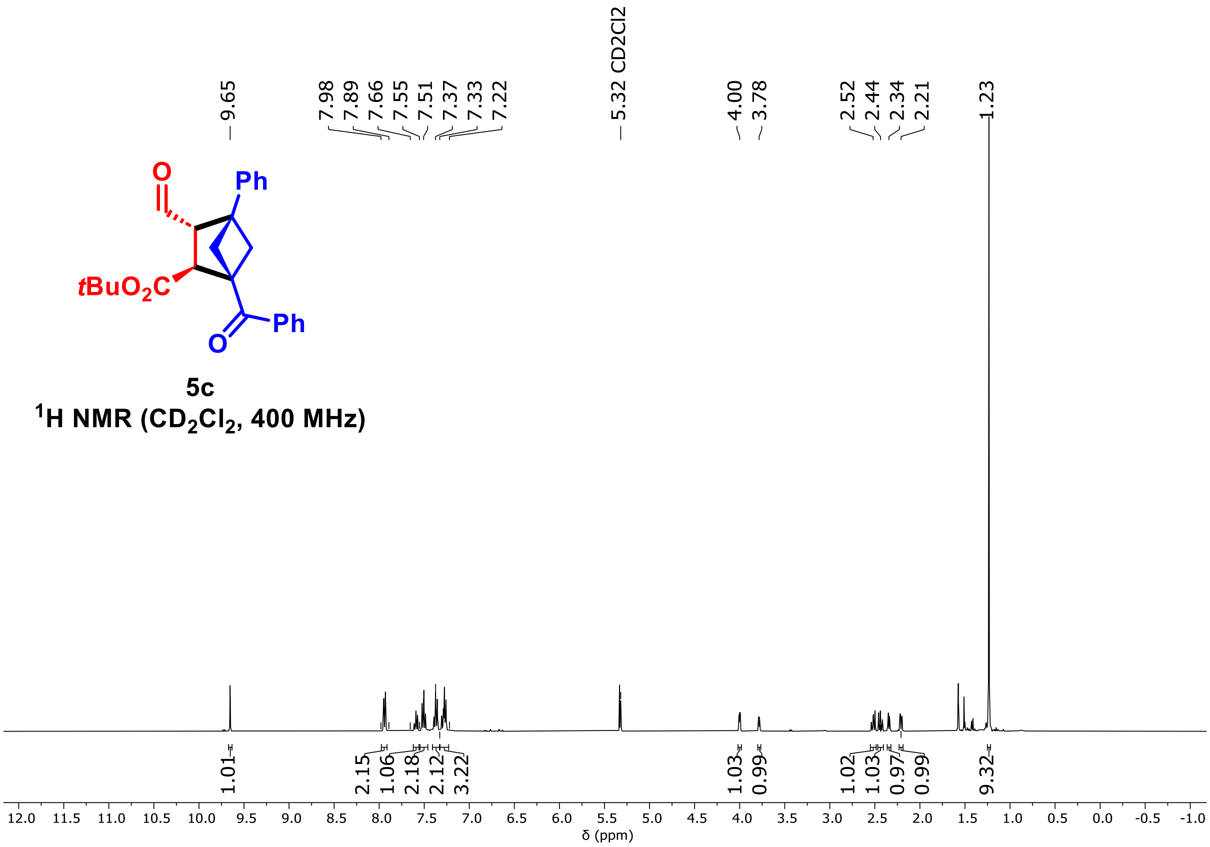

5c

^{1}H NMR (CD_2Cl_2, 400 MHz)

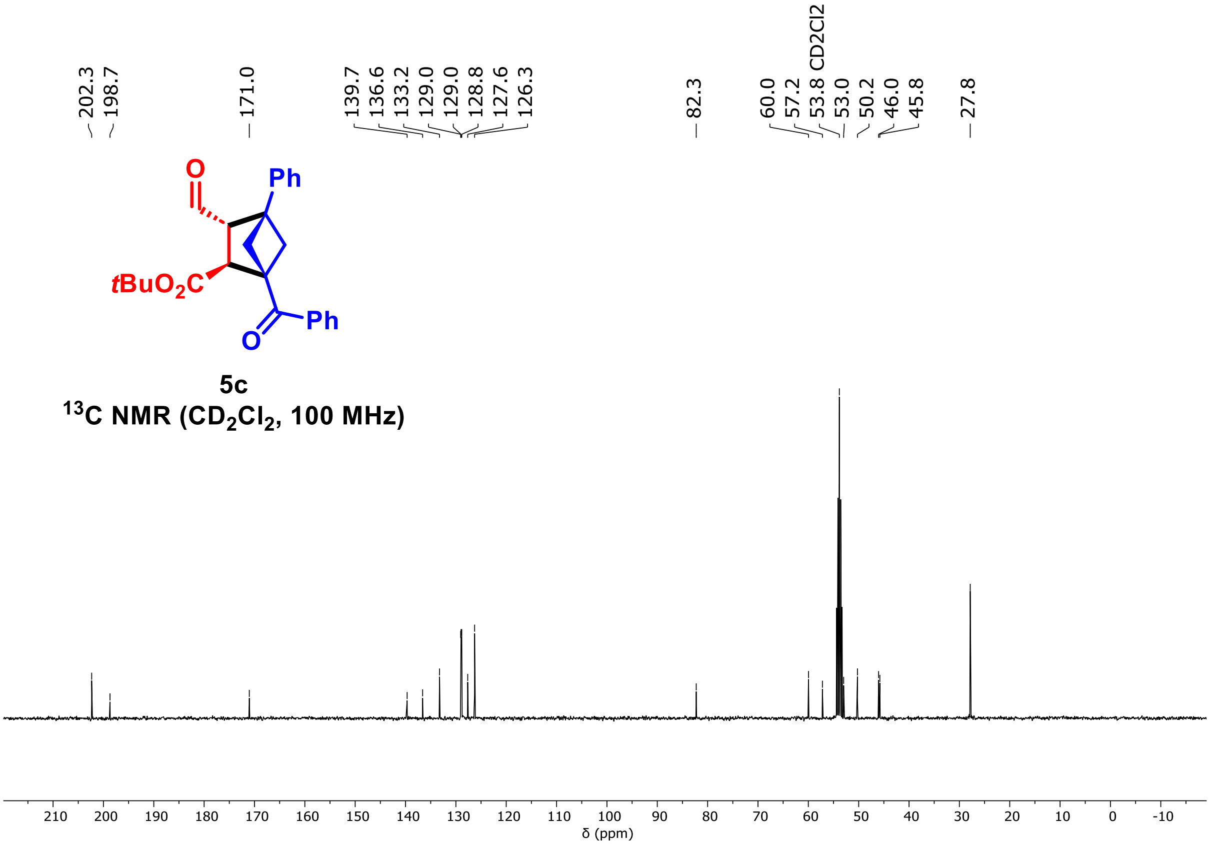

5c

^{13}C NMR (CD_2Cl_2, 100 MHz)

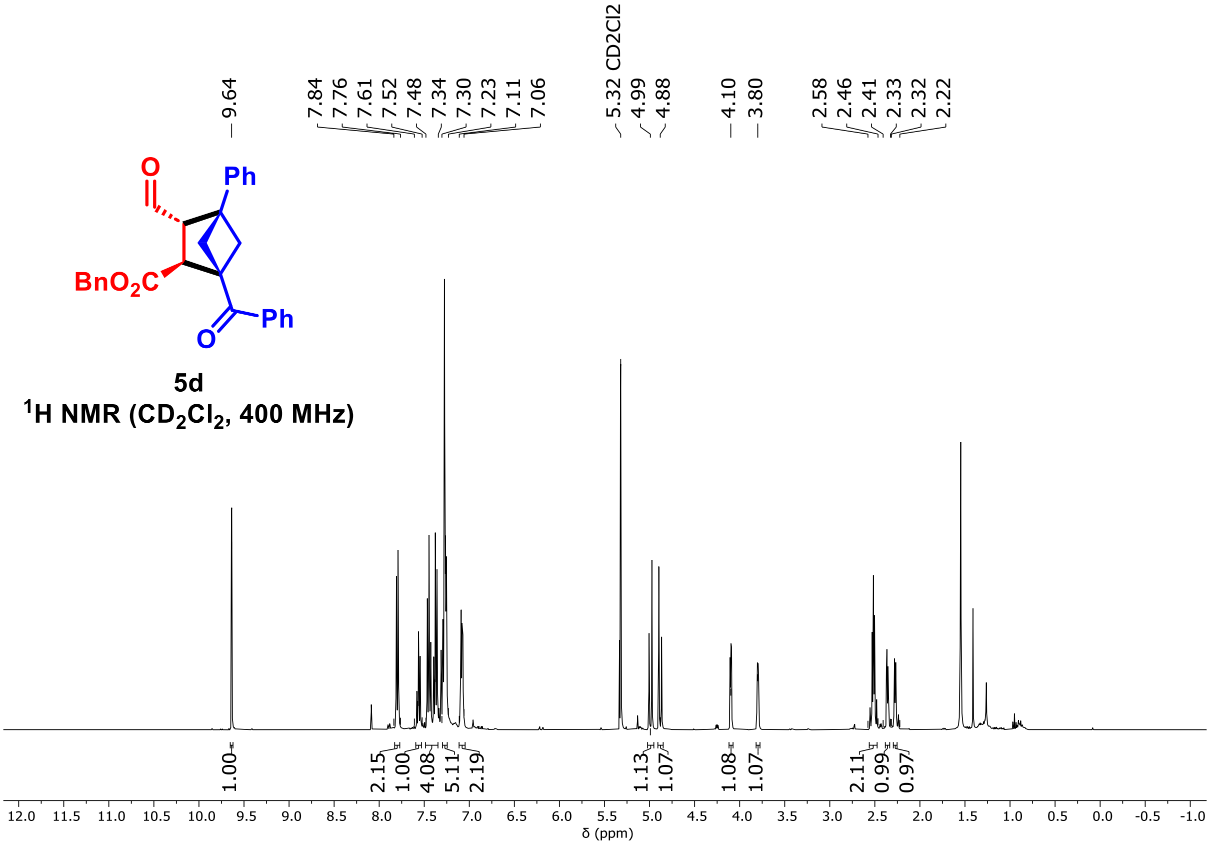
9.64
7.84
7.76
7.61
7.52
7.48
7.34
7.30
7.23
7.11
7.06
5.32 CD2Cl2
4.99
4.88
4.10
3.80
2.58
2.46
2.41
2.33
2.32
2.22
O
Ph
BnO2C
O
Ph
5d
1H NMR (CD2Cl2, 400 MHz)
1.00
2.15
1.00
4.08
5.11
2.19
1.13
1.07
1.08
1.07
2.11
0.99
0.97
12.0 11.5 11.0 10.5 10.0 9.5 9.0 8.5 8.0 7.5 7.0 6.5 6.0 5.5 5.0 4.5 4.0 3.5 3.0 2.5 2.0 1.5 1.0 0.5 0.0 -0.5 -1.0
δ (ppm)

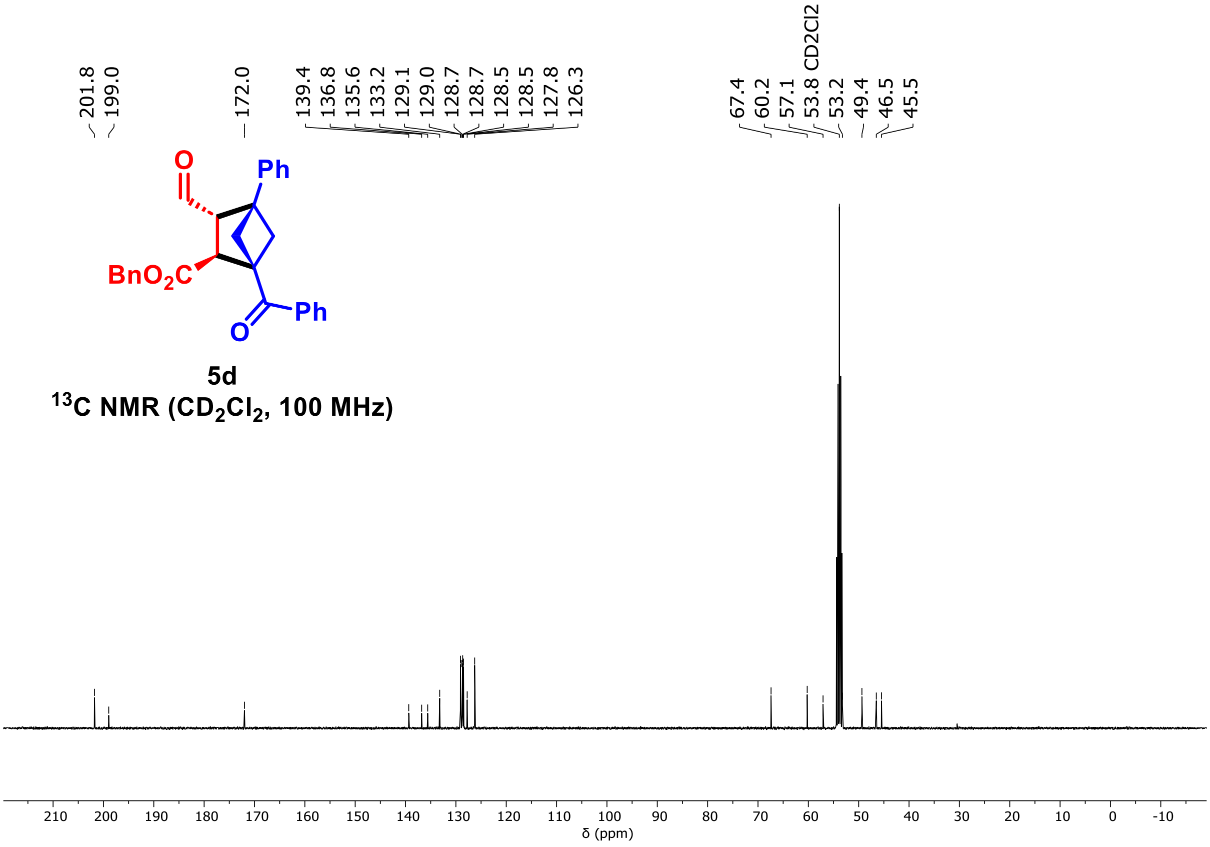
201.8
199.0
172.0
139.4
136.8
135.6
133.2
129.1
129.0
128.7
128.7
128.5
128.5
127.8
126.3
67.4
60.2
57.1
53.8 CD2Cl2
53.2
49.4
46.5
45.5
O
Ph
BnO2C
O
Ph
5d
13C NMR (CD2Cl2, 100 MHz)
210 200 190 180 170 160 150 140 130 120 110 100 90 80 70 60 50 40 30 20 10 0 -10
δ (ppm)

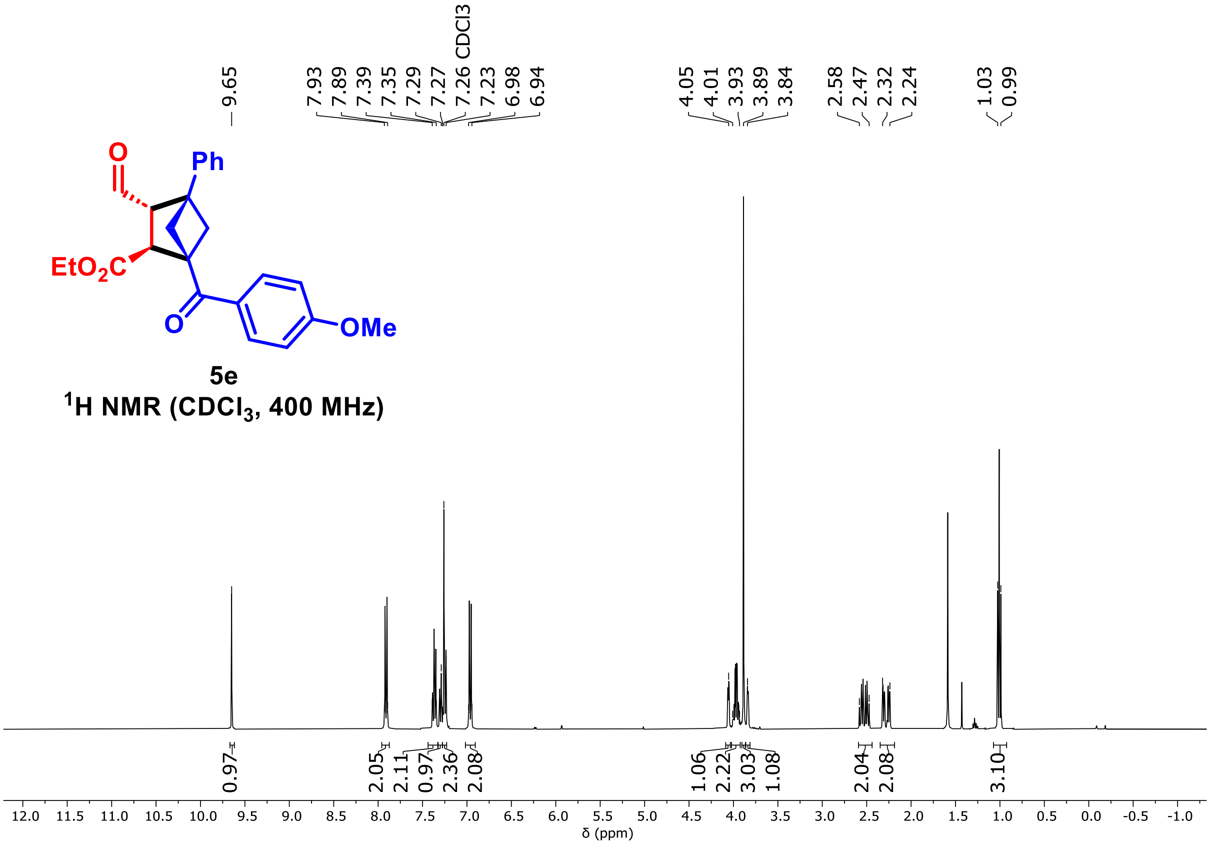
9.65
7.93
7.89
7.39
7.35
7.29
7.27
7.26 CDCl3
7.23
6.98
6.94
4.05
4.01
3.93
3.89
3.84
2.58
2.47
2.32
2.24
1.03
0.99
Ph
EtO2C
O
OMe
5e
1H NMR (CDCl3, 400 MHz)
0.97
2.05
2.11
0.97
2.36
2.08
1.06
2.22
3.03
1.08
2.04
2.08
3.10
12.0 11.5 11.0 10.5 10.0 9.5 9.0 8.5 8.0 7.5 7.0 6.5 6.0 5.5 5.0 4.5 4.0 3.5 3.0 2.5 2.0 1.5 1.0 0.5 0.0 -0.5 -1.0
δ (ppm)

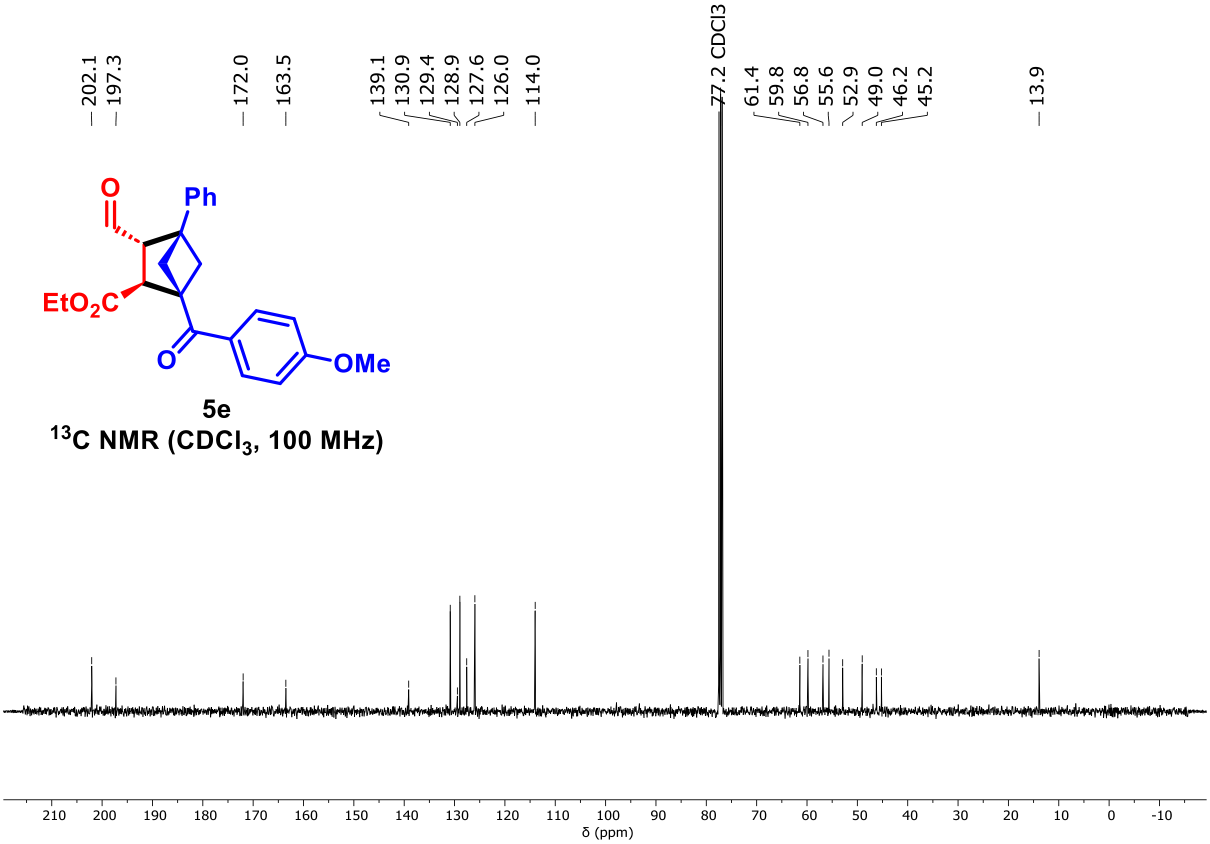
202.1
197.3
172.0
163.5
139.1
130.9
129.4
128.9
127.6
126.0
114.0
77.2 CDCl3
61.4
59.8
56.8
55.6
52.9
49.0
46.2
45.2
13.9
Ph
EtO2C
O
OMe
5e
13C NMR (CDCl3, 100 MHz)
210 200 190 180 170 160 150 140 130 120 110 100 90 80 70 60 50 40 30 20 10 0 -10
δ (ppm)

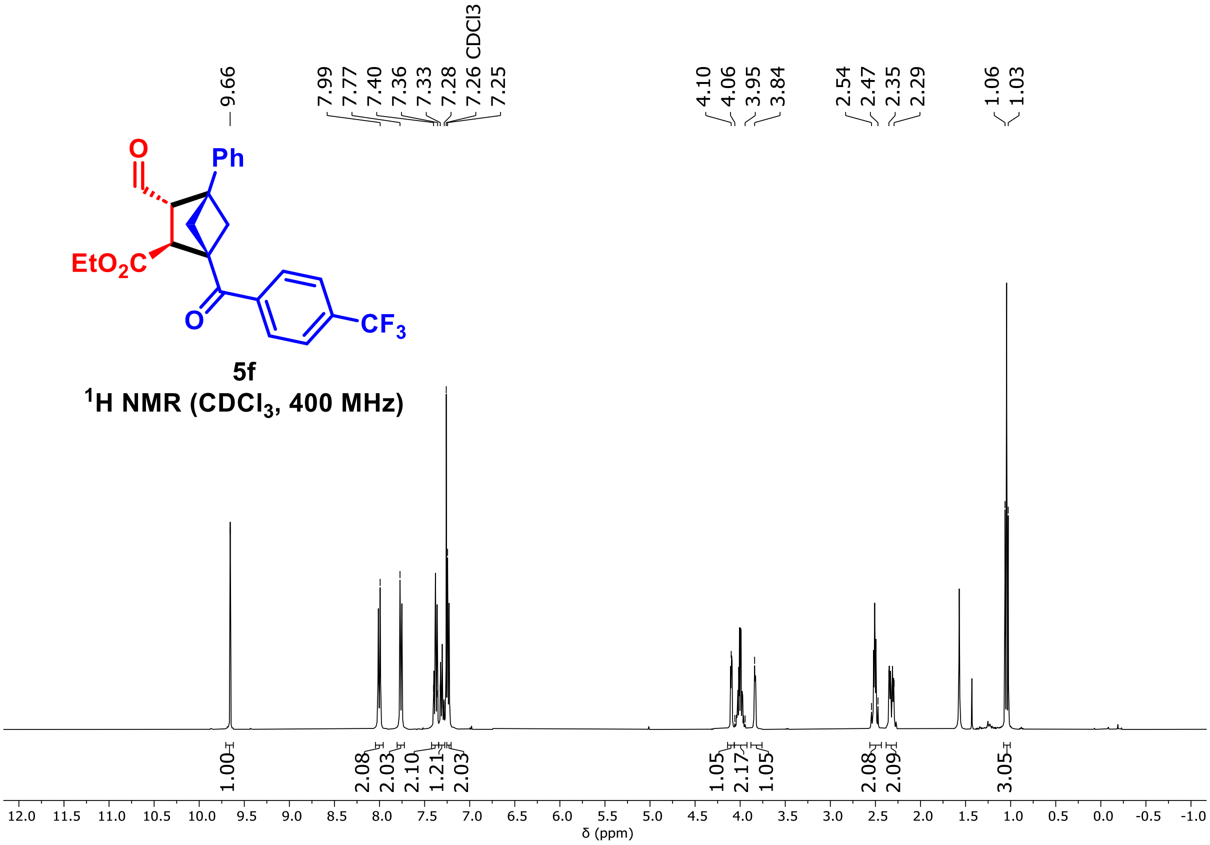
9.66
7.99
7.77
7.40
7.36
7.33
7.28
7.26 CDCl3
7.25
4.10
4.06
3.95
3.84
2.54
2.47
2.35
2.29
1.06
1.03
O
Ph
EtO2C
O
CF3
5f
1H NMR (CDCl3, 400 MHz)
1.00
2.08
2.03
2.10
1.21
2.03
1.05
2.17
1.05
2.08
2.09
3.05
δ (ppm)

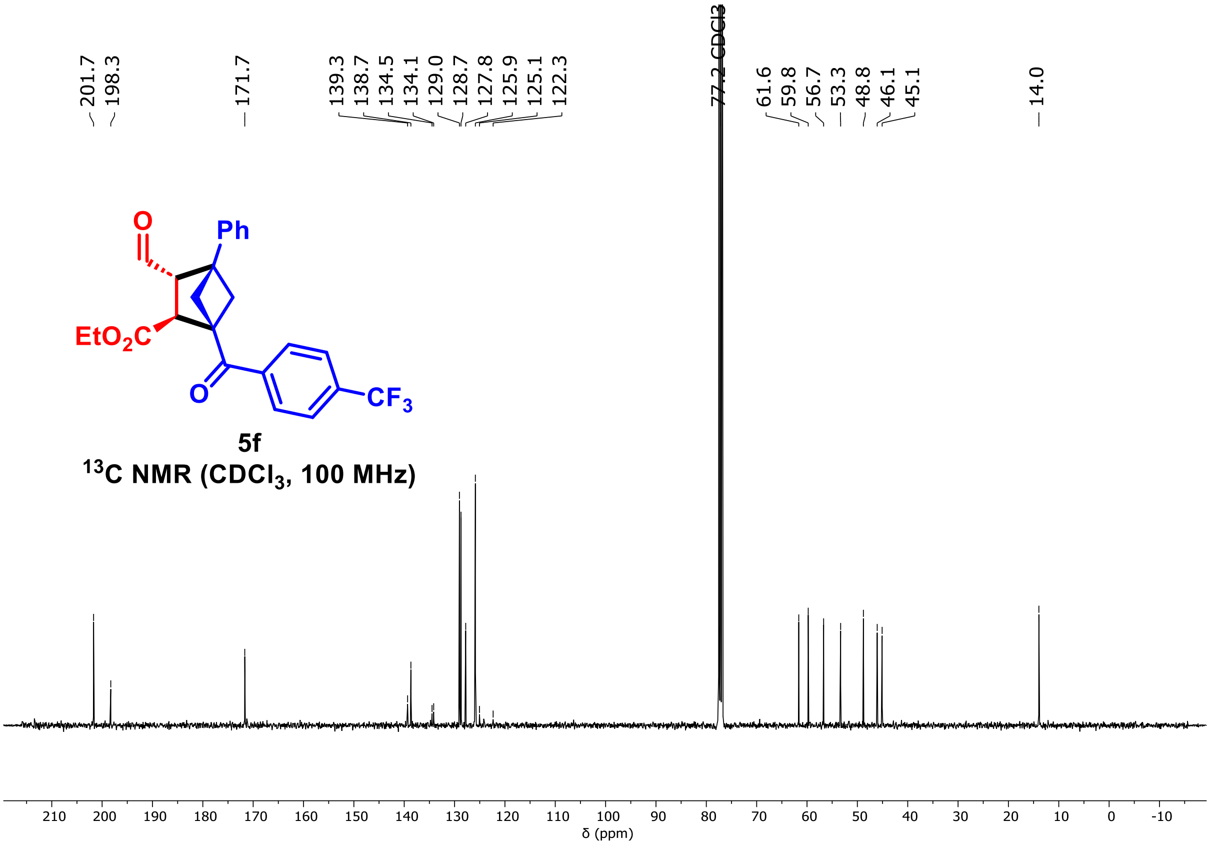
201.7
198.3
171.7
139.3
138.7
134.5
134.1
129.0
128.7
127.8
125.9
125.1
122.3
77.2 CDCl3
61.6
59.8
56.7
53.3
48.8
46.1
45.1
14.0
O
Ph
EtO2C
O
CF3
5f
13C NMR (CDCl3, 100 MHz)
δ (ppm)

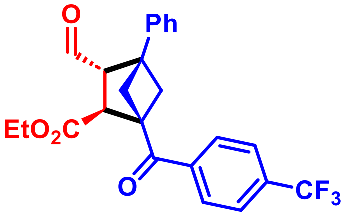

5f
^{19}F NMR ($CDCl_3$, 376 MHz)

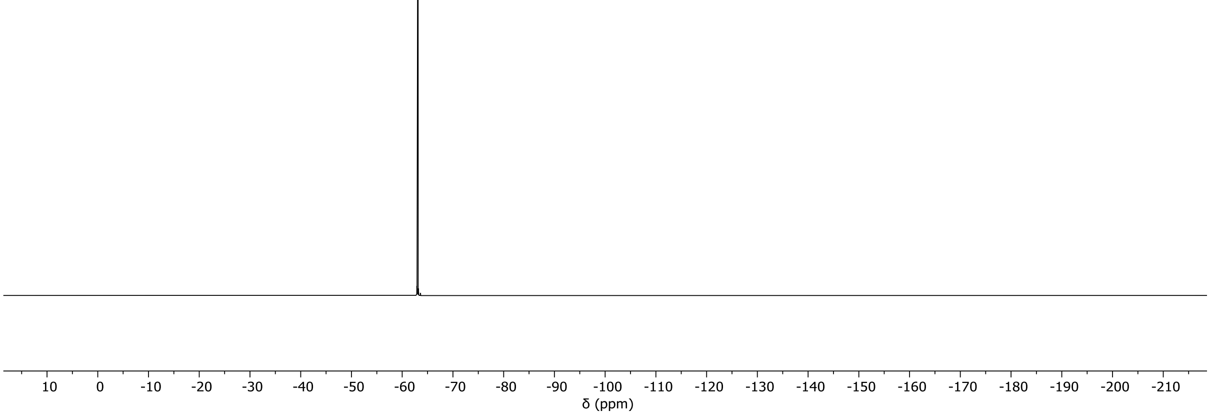

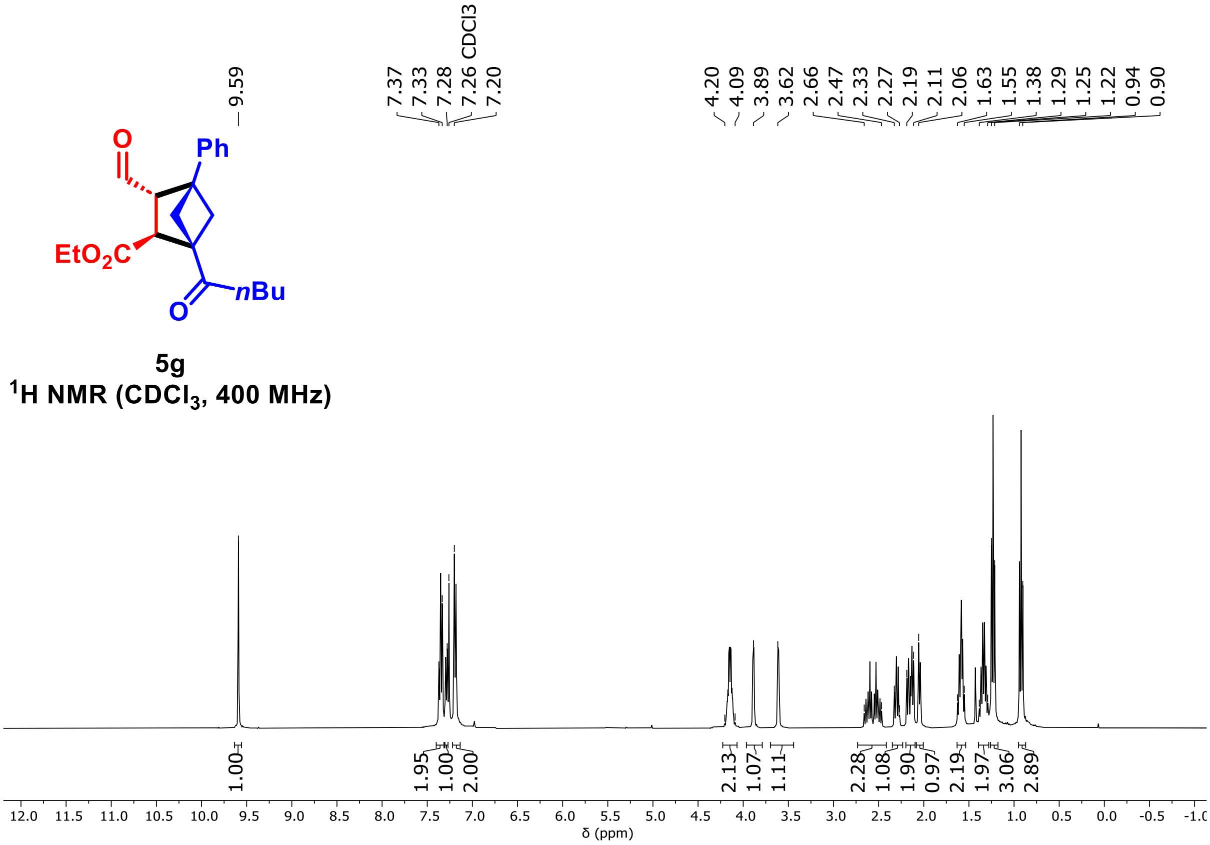

5g

^{1}H NMR ($CDCl_3$, 400 MHz)

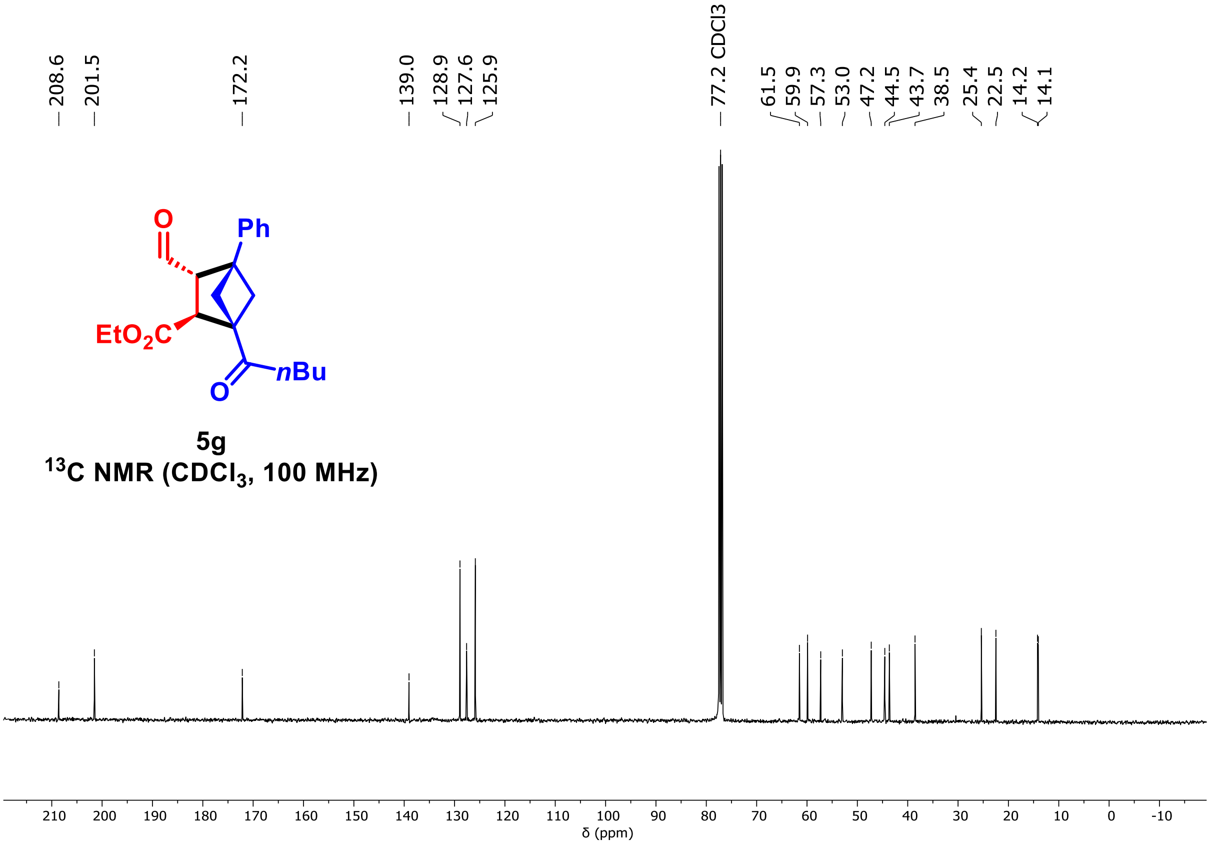

5g

^{13}C NMR ($CDCl_3$, 100 MHz)

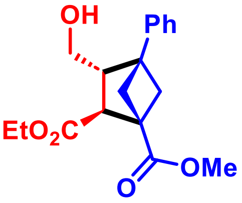

5h

^{1}H NMR ($CDCl_3$, 400 MHz)

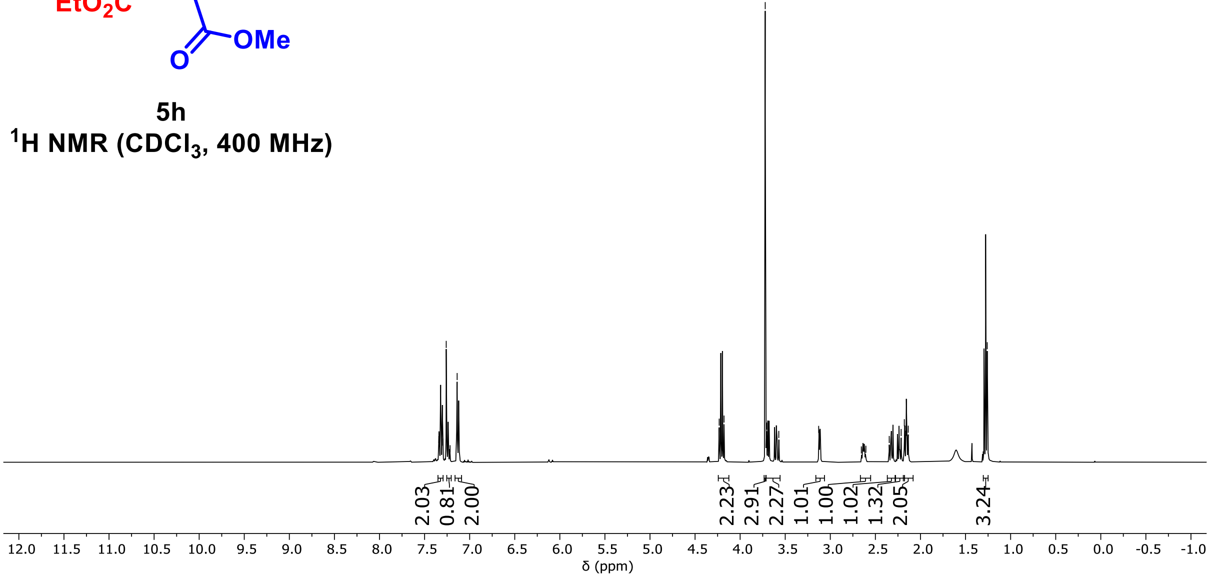

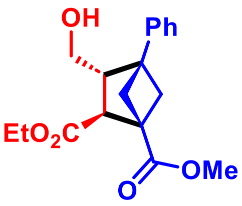

5h

^{13}C NMR ($CDCl_3$, 100 MHz)

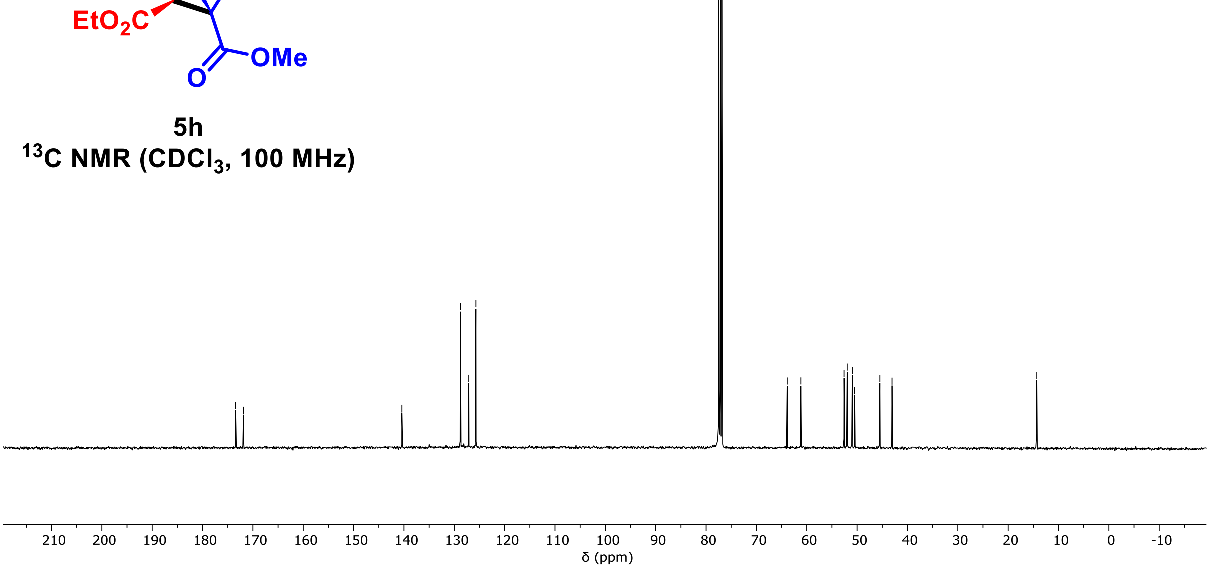

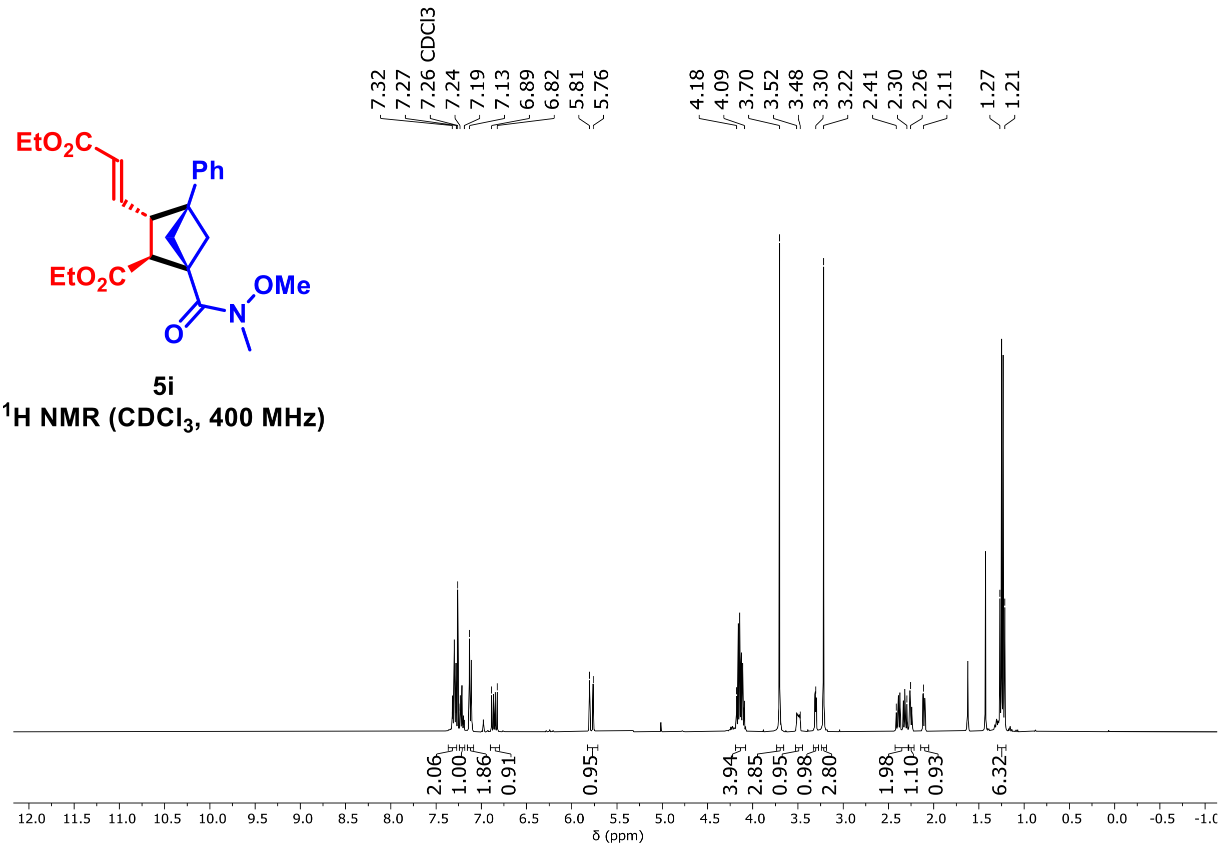

5i

^{1}H NMR ($CDCl_3$, 400 MHz)

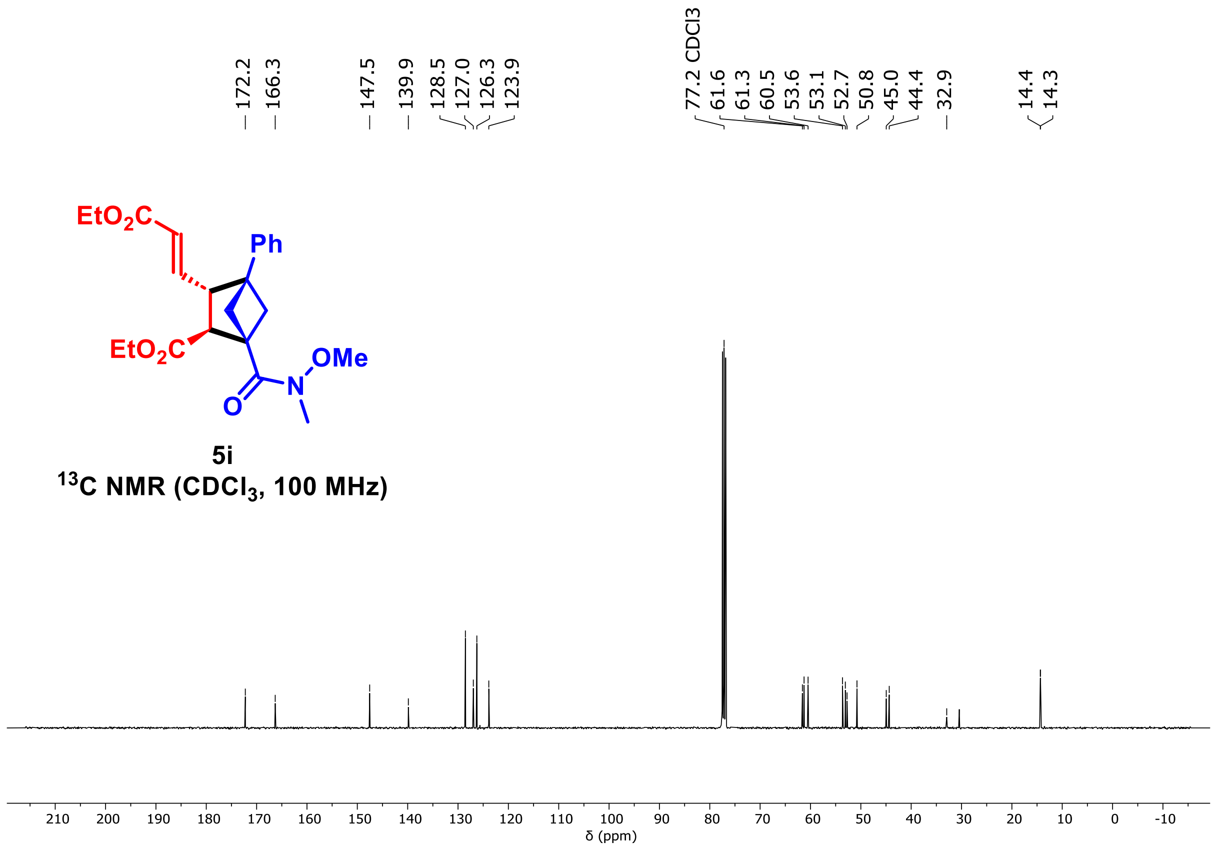

5i

^{13}C NMR ($CDCl_3$, 100 MHz)

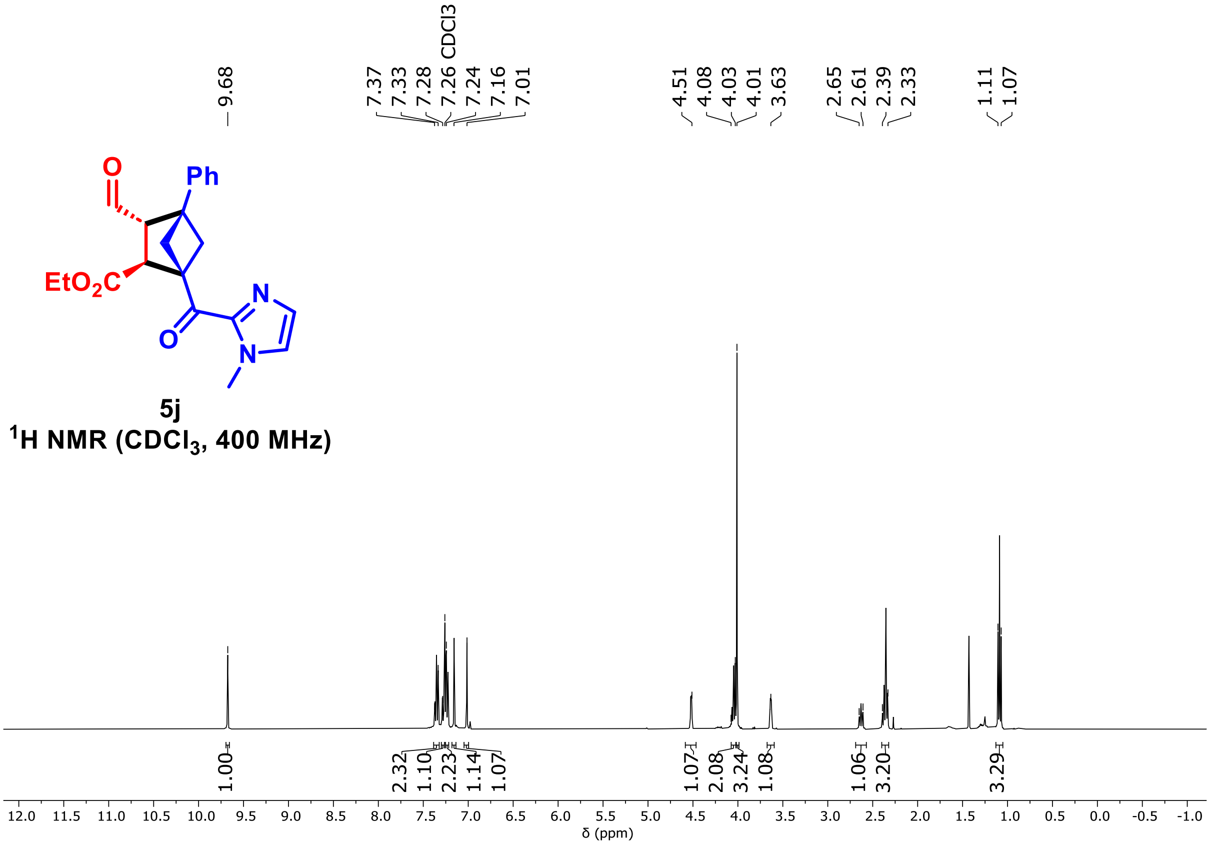
9.68
7.37 7.33 7.28 7.26 CDCl3 7.24 7.16 7.01
4.51 4.08 4.03 4.01 3.63
2.65 2.61 2.39 2.33
1.11 1.07
Ph
EtO2C
O
N
5j
1H NMR (CDCl3, 400 MHz)
1.00
2.32 1.10 2.23 1.14 1.07
1.07 2.08 3.24 1.08
1.06 3.20
3.29
12.0 11.5 11.0 10.5 10.0 9.5 9.0 8.5 8.0 7.5 7.0 6.5 6.0 5.5 5.0 4.5 4.0 3.5 3.0 2.5 2.0 1.5 1.0 0.5 0.0 -0.5 -1.0
δ (ppm)

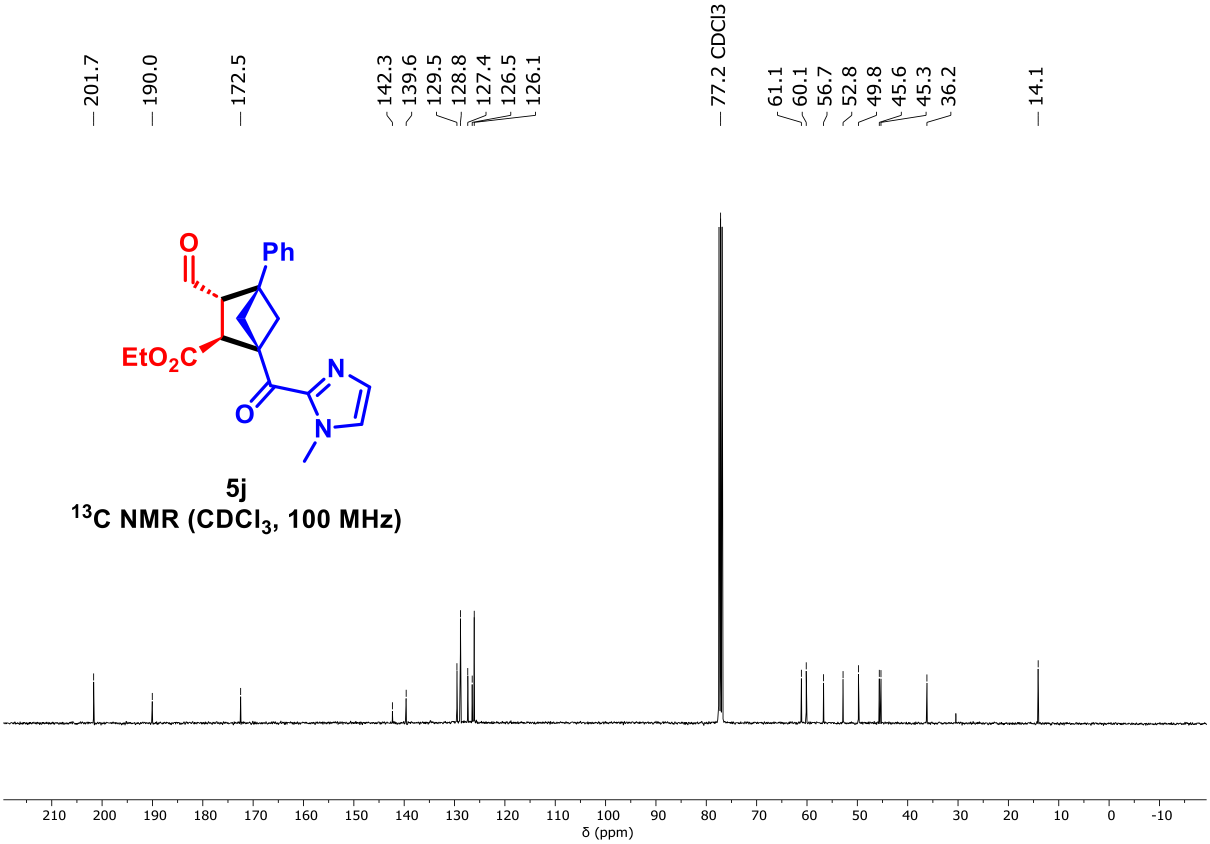
201.7
190.0
172.5
142.3 139.6 129.5 128.8 127.4 126.5 126.1
77.2 CDCl3
61.1 60.1 56.7 52.8 49.8 45.6 45.3 36.2
14.1
Ph
EtO2C
O
N
5j
13C NMR (CDCl3, 100 MHz)
210 200 190 180 170 160 150 140 130 120 110 100 90 80 70 60 50 40 30 20 10 0 -10
δ (ppm)

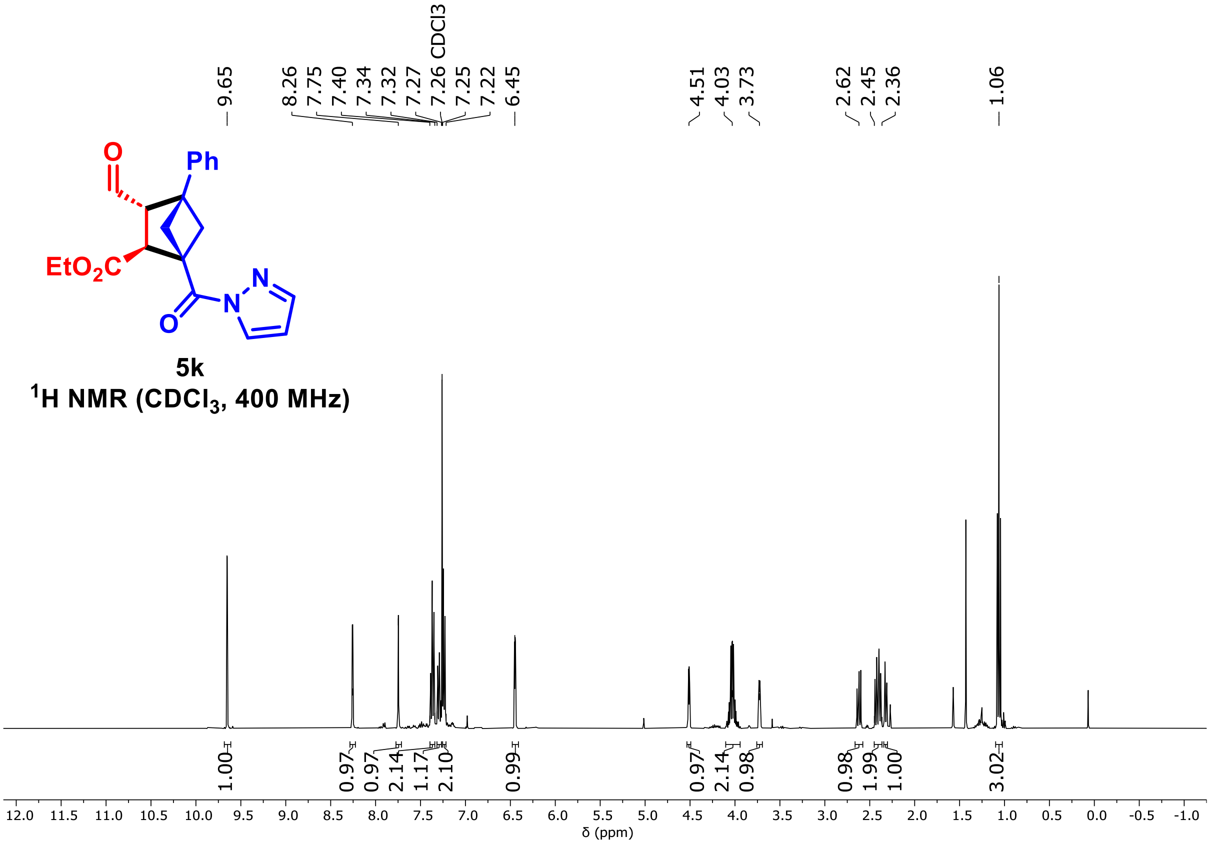
9.65
8.26
7.75
7.40
7.34
7.32
7.27
7.26 CDCl3
7.25
7.22
6.45
4.51
4.03
3.73
2.62
2.45
2.36
1.06
O
Ph
EtO2C
N
N
O
5k
1H NMR (CDCl3, 400 MHz)
1.00
0.97
0.97
2.14
1.17
2.10
0.99
0.97
2.14
0.98
0.98
1.99
1.00
3.02
δ (ppm)

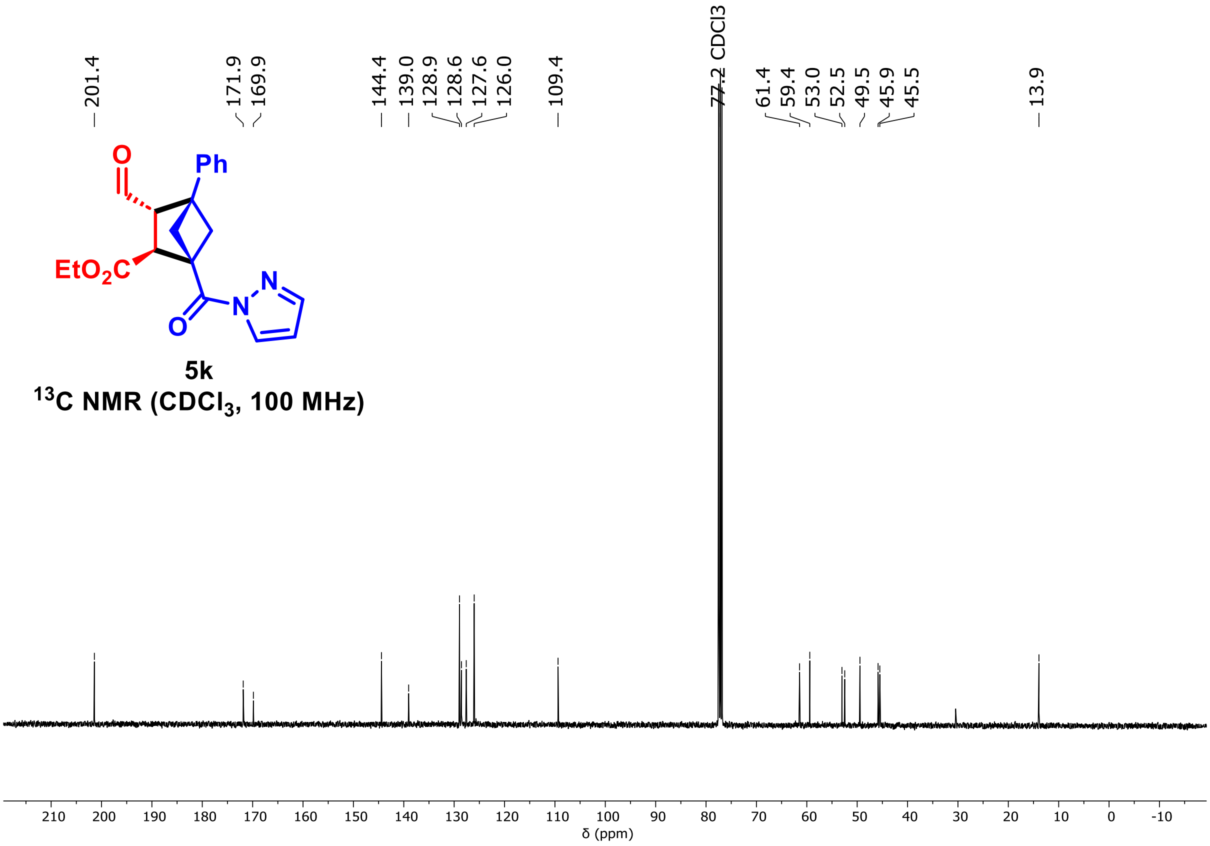
201.4
171.9
169.9
144.4
139.0
128.9
128.6
127.6
126.0
109.4
77.2 CDCl3
61.4
59.4
53.0
52.5
49.5
45.9
45.5
13.9
O
Ph
EtO2C
N
N
O
5k
13C NMR (CDCl3, 100 MHz)
δ (ppm)

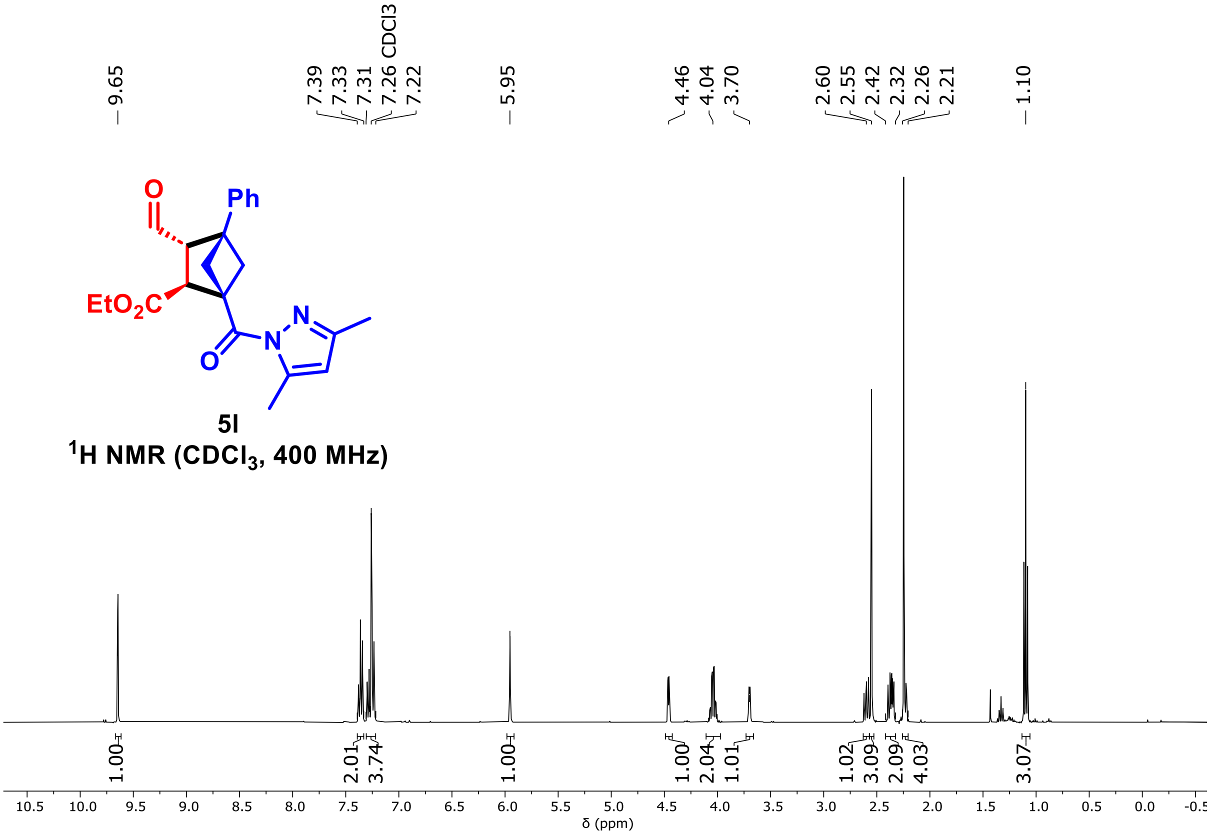
9.65
7.39
7.33
7.31
7.26 CDCl3
7.22
5.95
4.46
4.04
3.70
2.60
2.55
2.42
2.32
2.26
2.21
1.10
O
Ph
EtO2C
N
N
O
5l
1H NMR (CDCl3, 400 MHz)
1.00
2.01
3.74
1.00
1.00
2.04
1.01
1.02
3.09
2.09
4.03
3.07
δ (ppm)

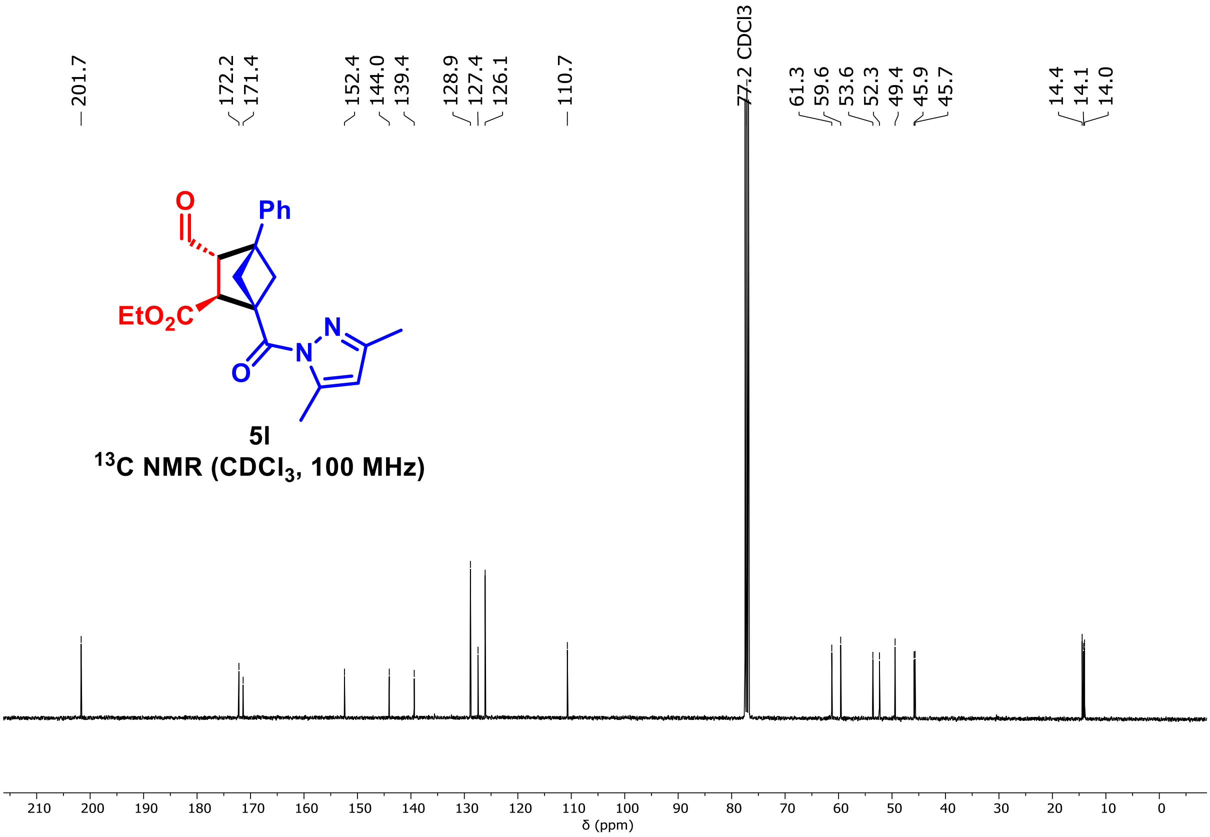
201.7
172.2
171.4
152.4
144.0
139.4
128.9
127.4
126.1
110.7
77.2 CDCl3
61.3
59.6
53.6
52.3
49.4
45.9
45.7
14.4
14.1
14.0
O
Ph
EtO2C
N
N
O
5l
13C NMR (CDCl3, 100 MHz)
δ (ppm)

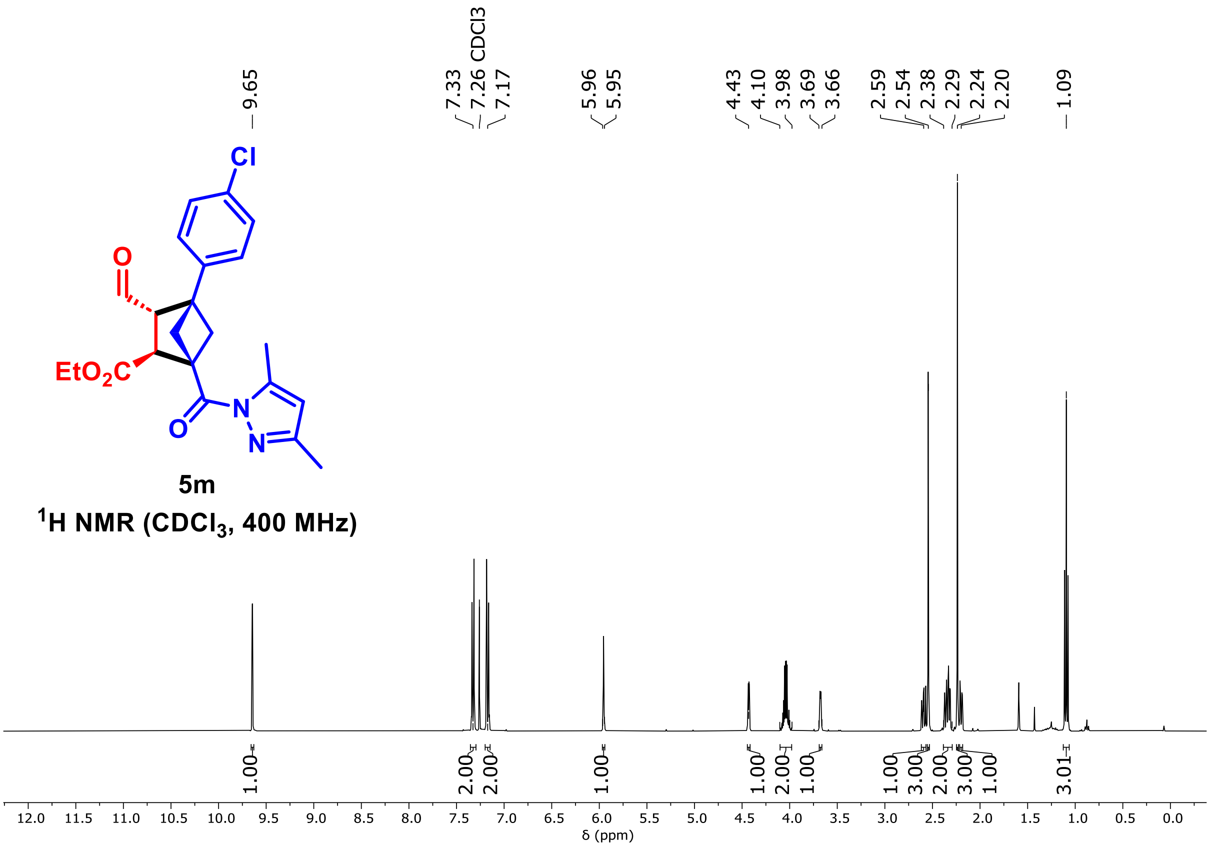

5m

^{1}H NMR ($CDCl_3$, 400 MHz)

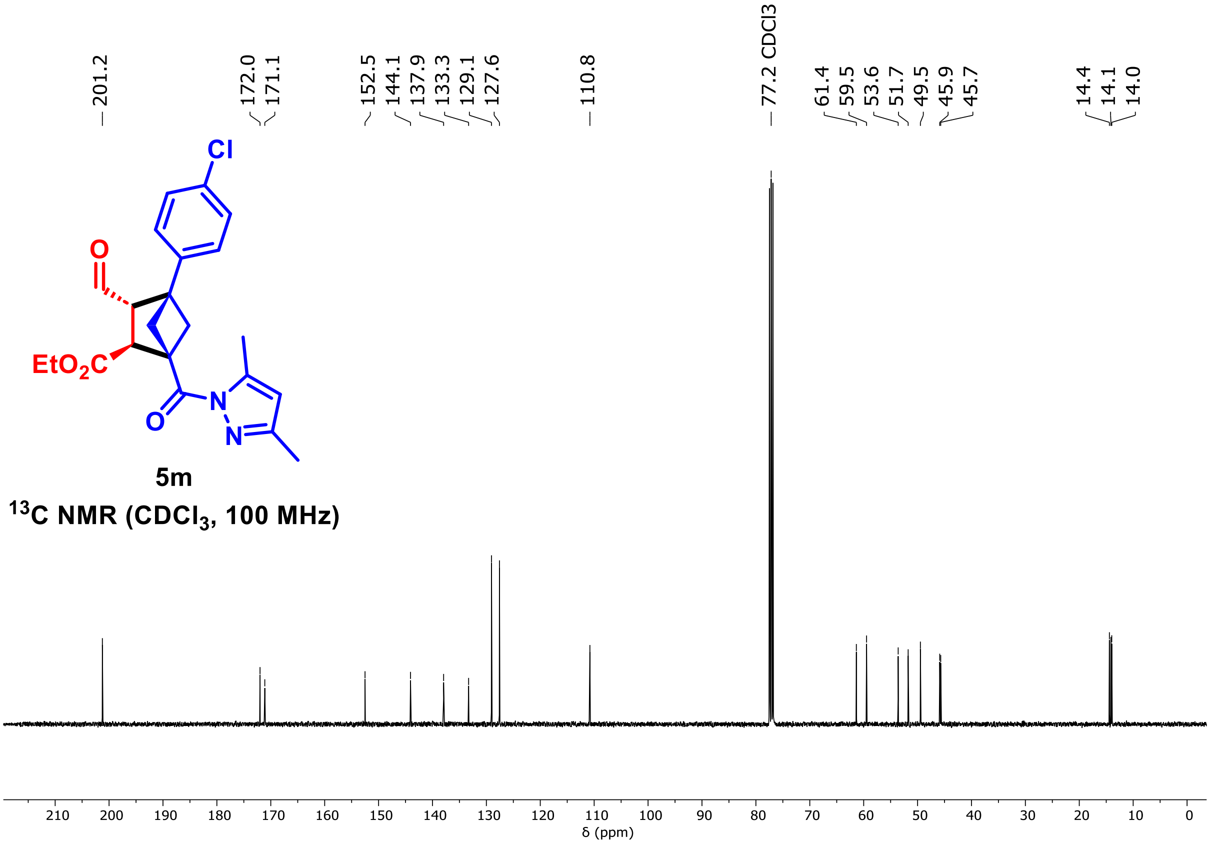

5m

^{13}C NMR ($CDCl_3$, 100 MHz)

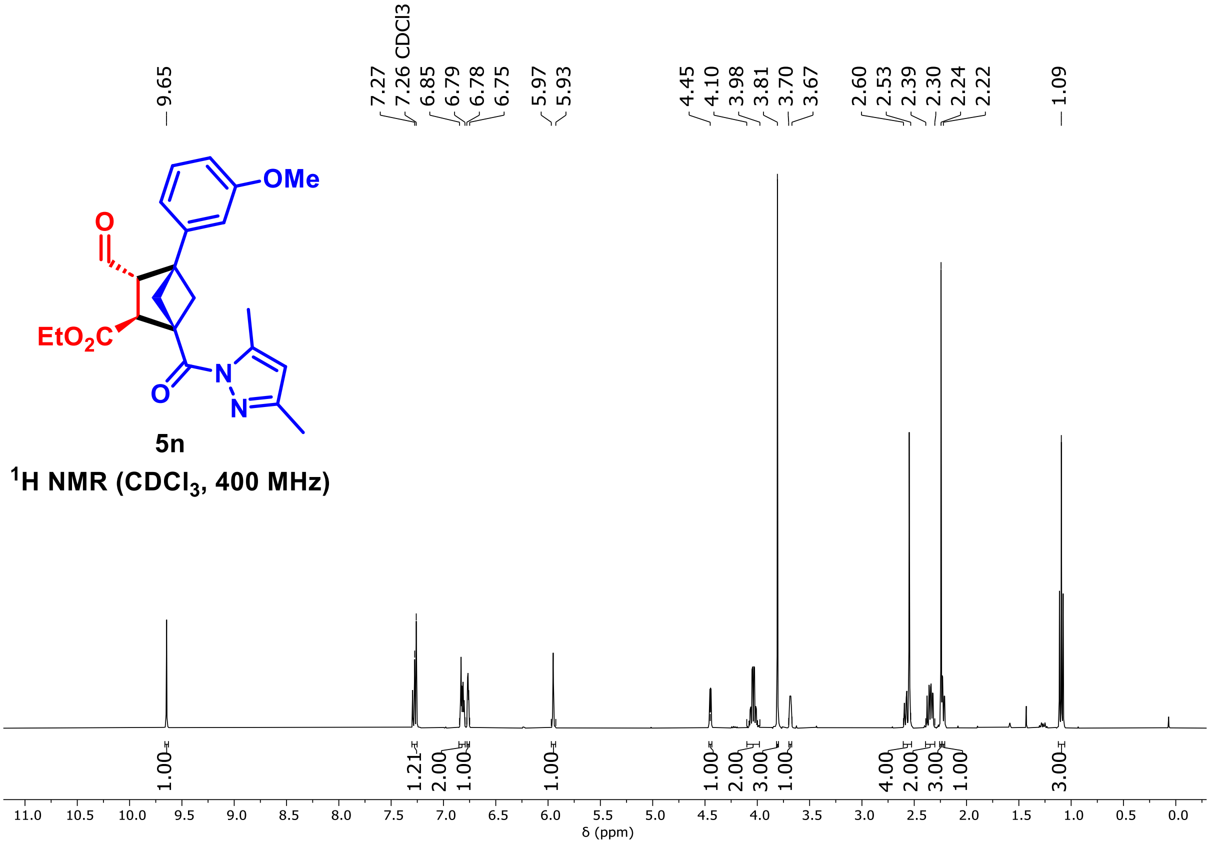
9.65
7.27
7.26 CDCl3
6.85
6.79
6.78
6.75
5.97
5.93
4.45
4.10
3.98
3.81
3.70
3.67
2.60
2.53
2.39
2.30
2.24
2.22
1.09
OMe
O
EtO2C
N
N
O
5n
1H NMR (CDCl3, 400 MHz)
1.00
1.21
2.00
1.00
1.00
1.00
2.00
3.00
1.00
4.00
2.00
3.00
1.00
3.00
11.0
10.5
10.0
9.5
9.0
8.5
8.0
7.5
7.0
6.5
6.0
5.5
5.0
4.5
4.0
3.5
3.0
2.5
2.0
1.5
1.0
0.5
0.0
δ (ppm)

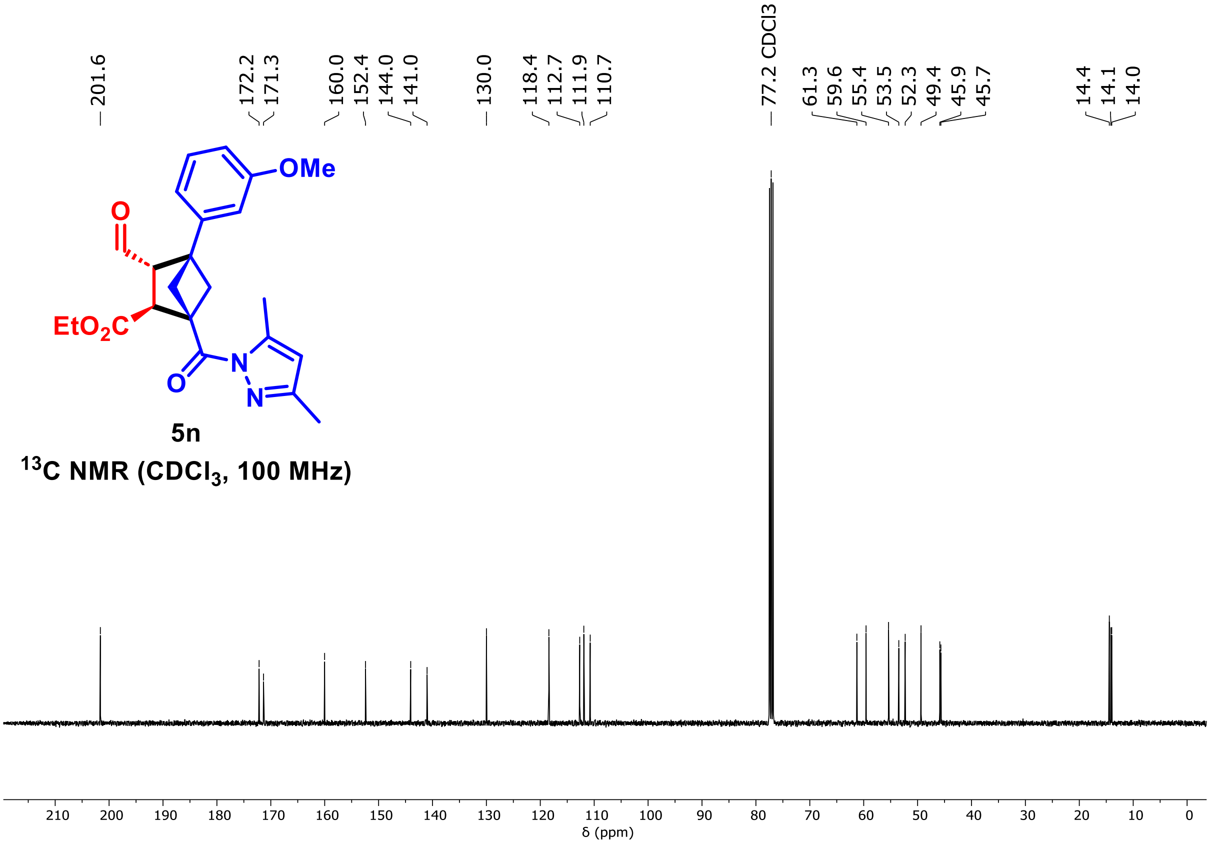
201.6
172.2
171.3
160.0
152.4
144.0
141.0
130.0
118.4
112.7
111.9
110.7
77.2 CDCl3
61.3
59.6
55.4
53.5
52.3
49.4
45.9
45.7
14.4
14.1
14.0
OMe
O
EtO2C
N
N
O
5n
13C NMR (CDCl3, 100 MHz)
210
200
190
180
170
160
150
140
130
120
110
100
90
80
70
60
50
40
30
20
10
0
δ (ppm)

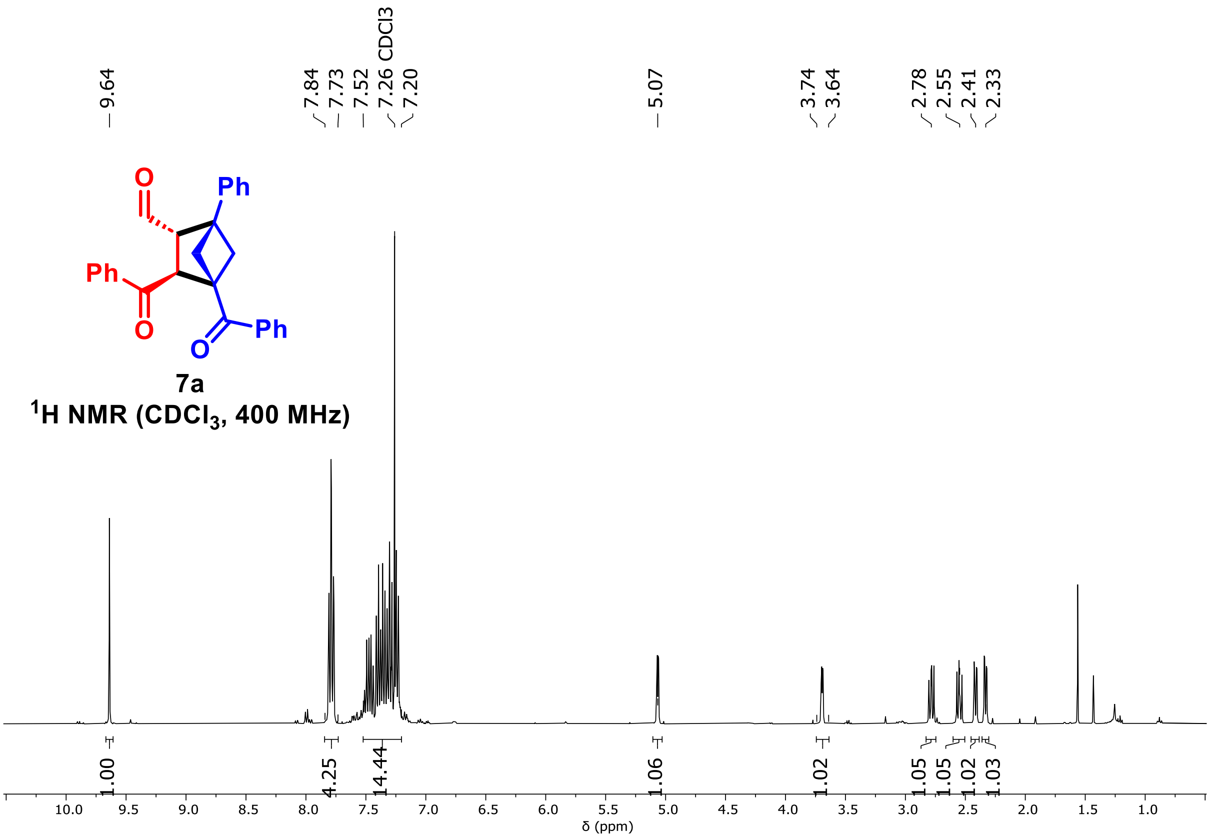
9.64
7.84
7.73
7.52
7.26 CDCl3
7.20
5.07
3.74
3.64
2.78
2.55
2.41
2.33
O
Ph
Ph
O
O
Ph
7a
1H NMR (CDCl3, 400 MHz)
1.00
4.25
14.44
1.06
1.02
1.05
1.05
1.02
1.03
10.0 9.5 9.0 8.5 8.0 7.5 7.0 6.5 6.0 5.5 5.0 4.5 4.0 3.5 3.0 2.5 2.0 1.5 1.0
δ (ppm)

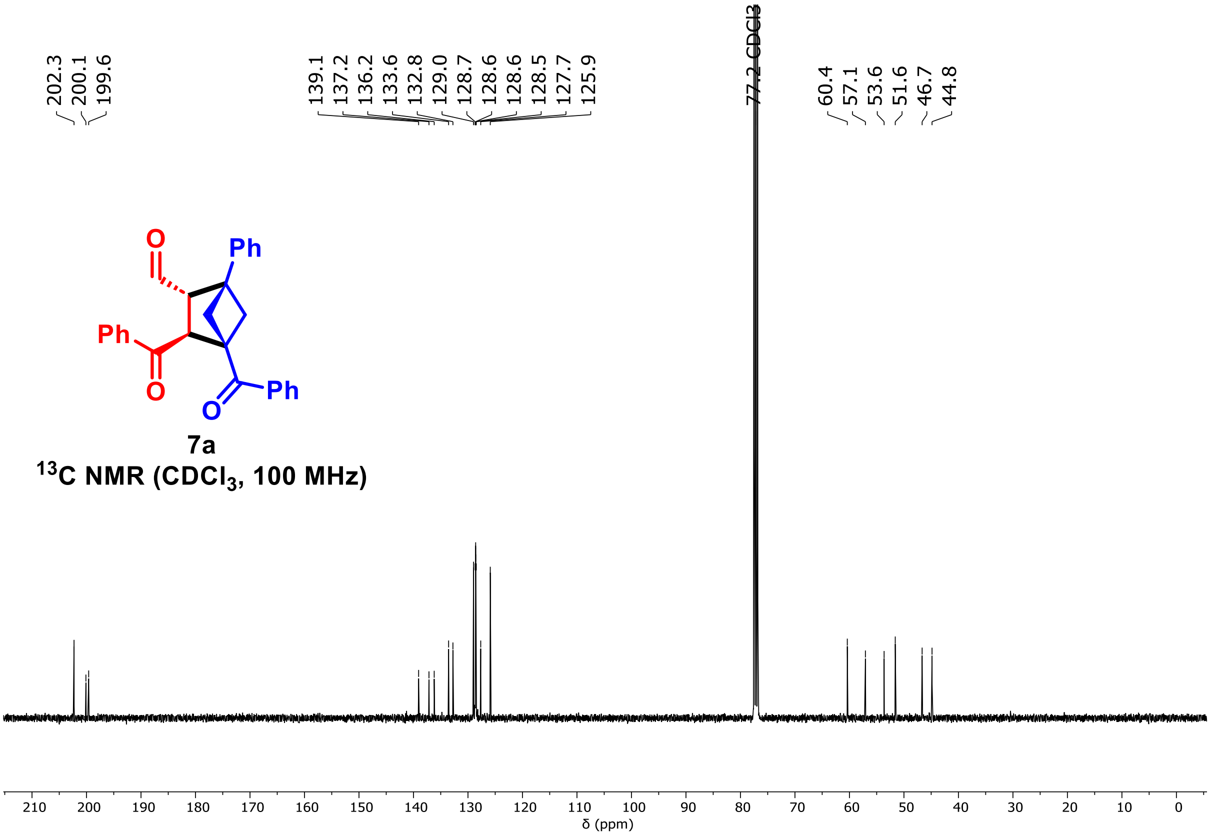
202.3
200.1
199.6
139.1
137.2
136.2
133.6
132.8
129.0
128.7
128.6
128.6
128.5
127.7
125.9
77.2 CDCl3
60.4
57.1
53.6
51.6
46.7
44.8
O
Ph
Ph
O
O
Ph
7a
13C NMR (CDCl3, 100 MHz)
210 200 190 180 170 160 150 140 130 120 110 100 90 80 70 60 50 40 30 20 10 0
δ (ppm)

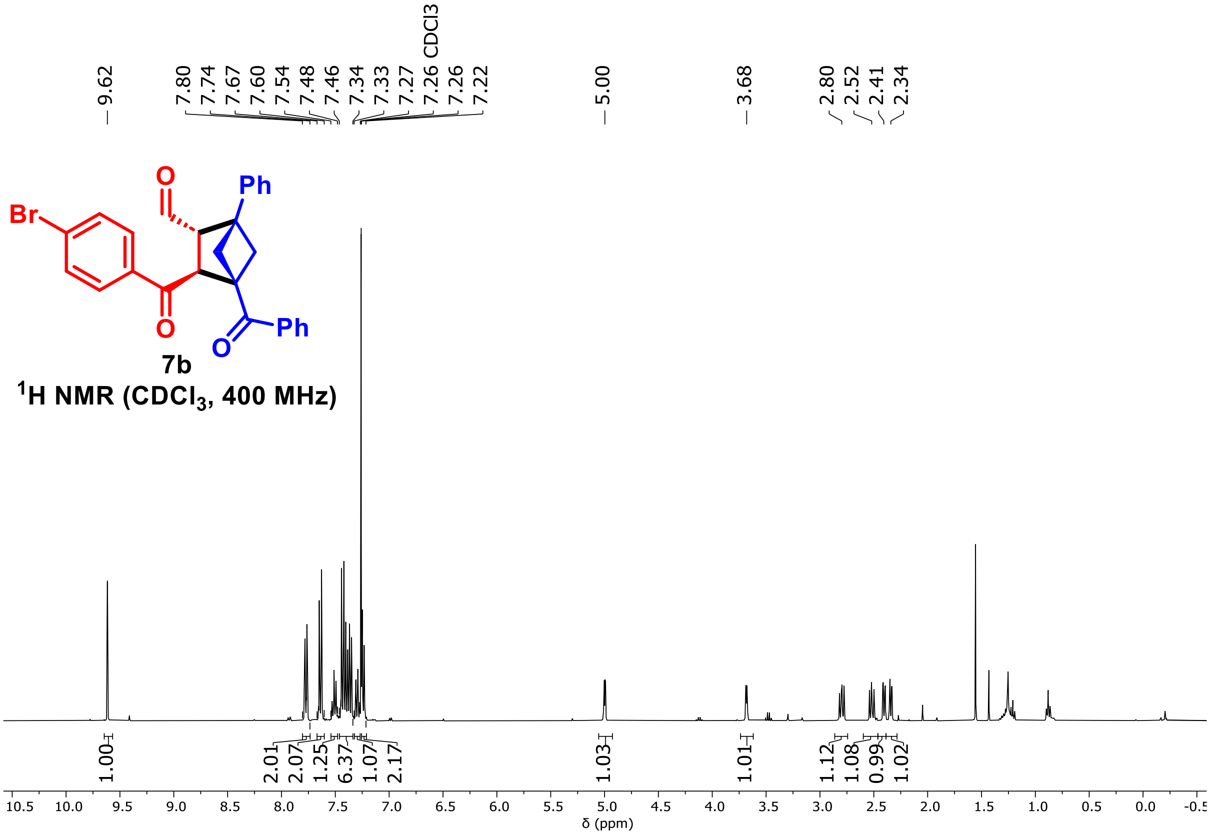
9.62
7.80
7.74
7.67
7.60
7.54
7.48
7.46
7.34
7.33
7.27
7.26 CDCl3
7.26
7.22
5.00
3.68
2.80
2.52
2.41
2.34
Br
O
Ph
O
O
Ph
7b
^{1}H NMR ($CDCl_3$, 400 MHz)
1.00
2.01
2.07
1.25
6.37
1.07
2.17
1.03
1.01
1.12
1.08
0.99
1.02
δ (ppm)

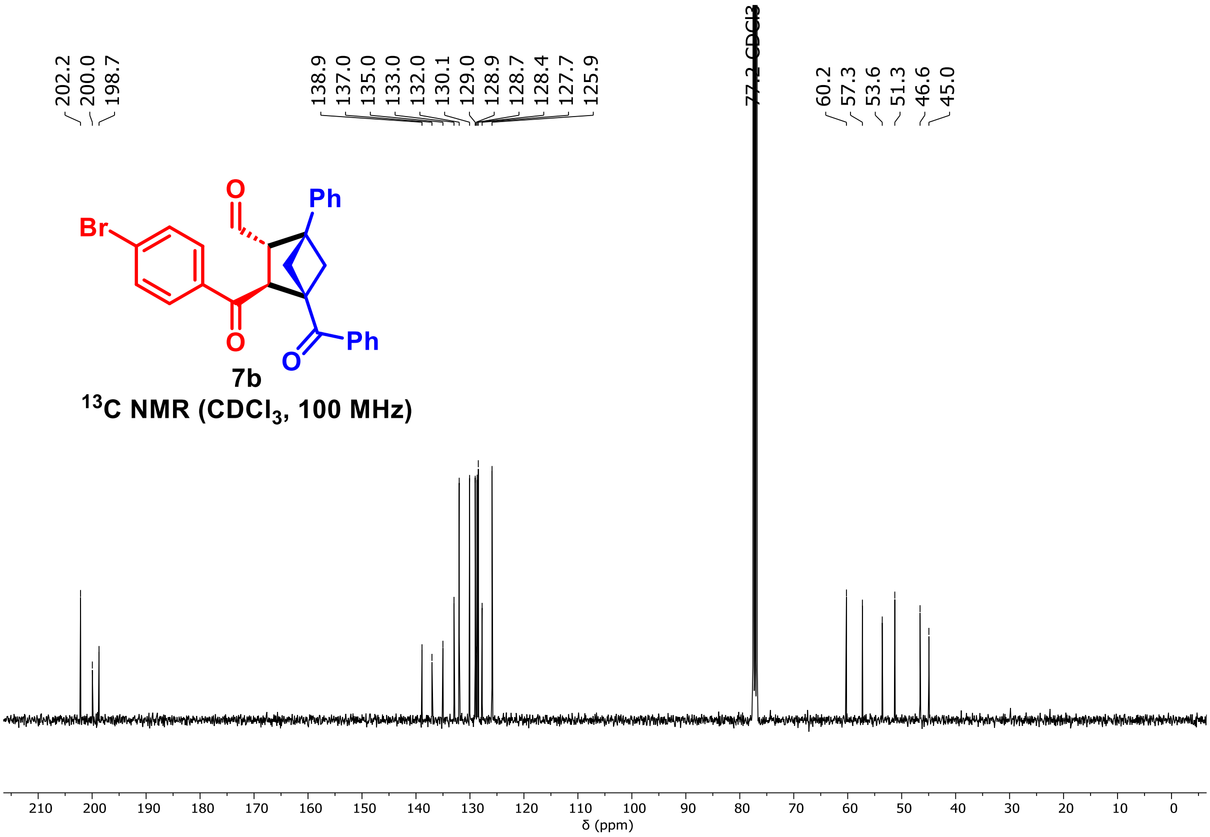
202.2
200.0
198.7
138.9
137.0
135.0
133.0
132.0
130.1
129.0
128.9
128.7
128.4
127.7
125.9
77.2 CDCl3
60.2
57.3
53.6
51.3
46.6
45.0
Br
O
Ph
O
O
Ph
7b
^{13}C NMR ($CDCl_3$, 100 MHz)
δ (ppm)

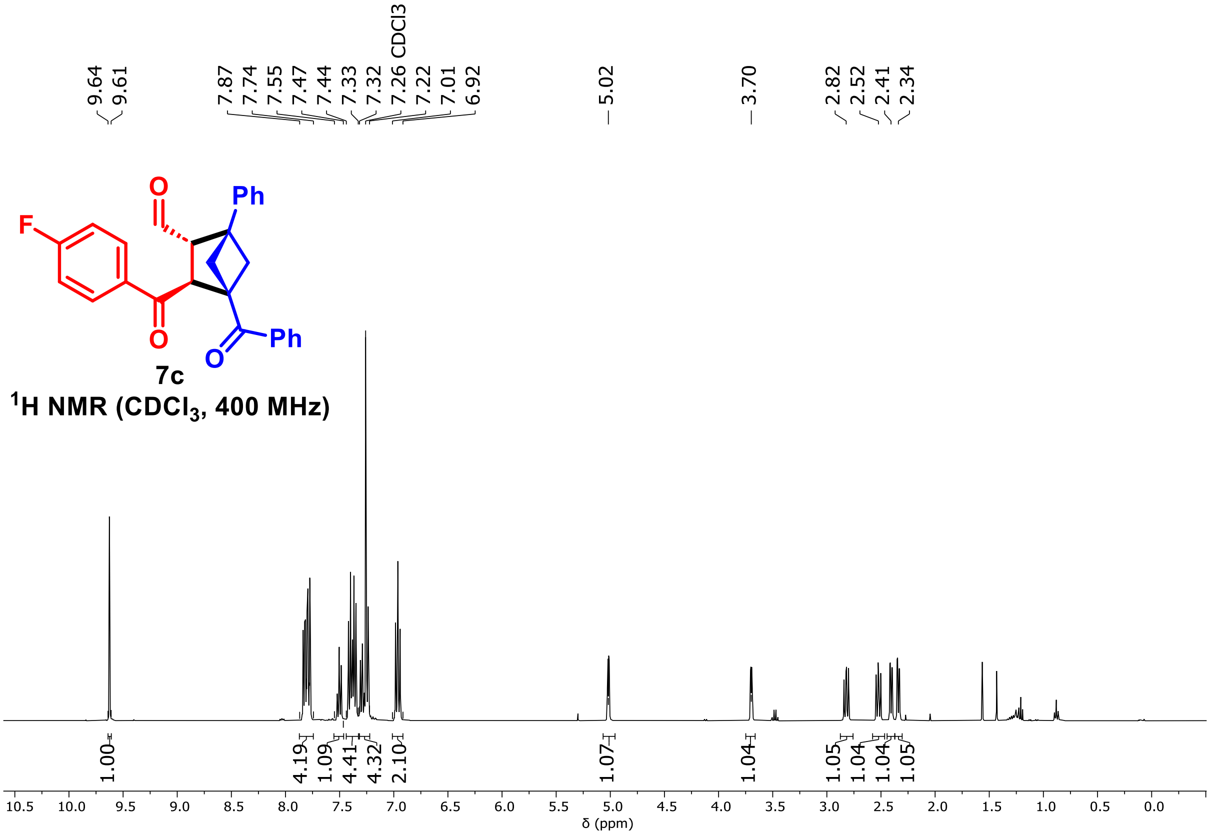
9.64
9.61
7.87
7.74
7.55
7.47
7.44
7.33
7.32
7.26 CDCl3
7.22
7.01
6.92
5.02
3.70
2.82
2.52
2.41
2.34
F
O
Ph
O
O
Ph
7c
^{1}H NMR ($CDCl_3$, 400 MHz)
1.00
4.19
1.09
4.41
4.32
2.10
1.07
1.04
1.05
1.04
1.04
1.05
δ (ppm)

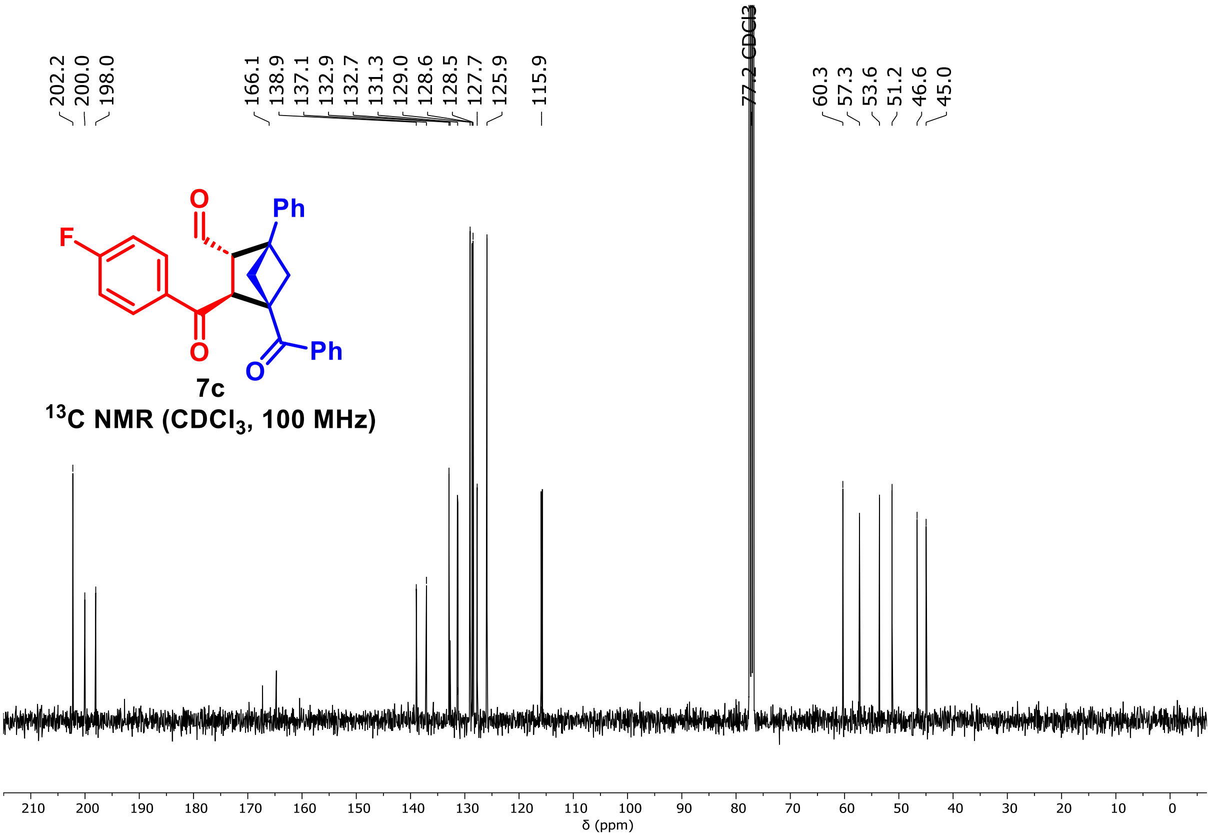
202.2
200.0
198.0
166.1
138.9
137.1
132.9
132.7
131.3
129.0
128.6
128.5
127.7
125.9
115.9
77.2 CDCl3
60.3
57.3
53.6
51.2
46.6
45.0
F
O
Ph
O
O
Ph
7c
^{13}C NMR ($CDCl_3$, 100 MHz)
δ (ppm)

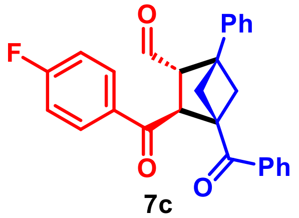

7c

^{19}F NMR ($CDCl_3$, 376 MHz)

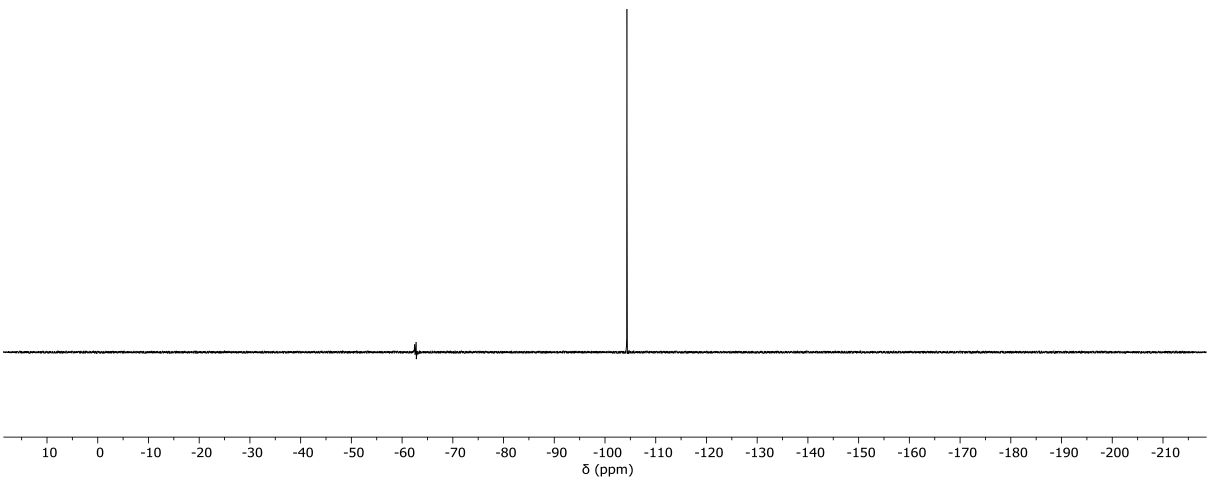

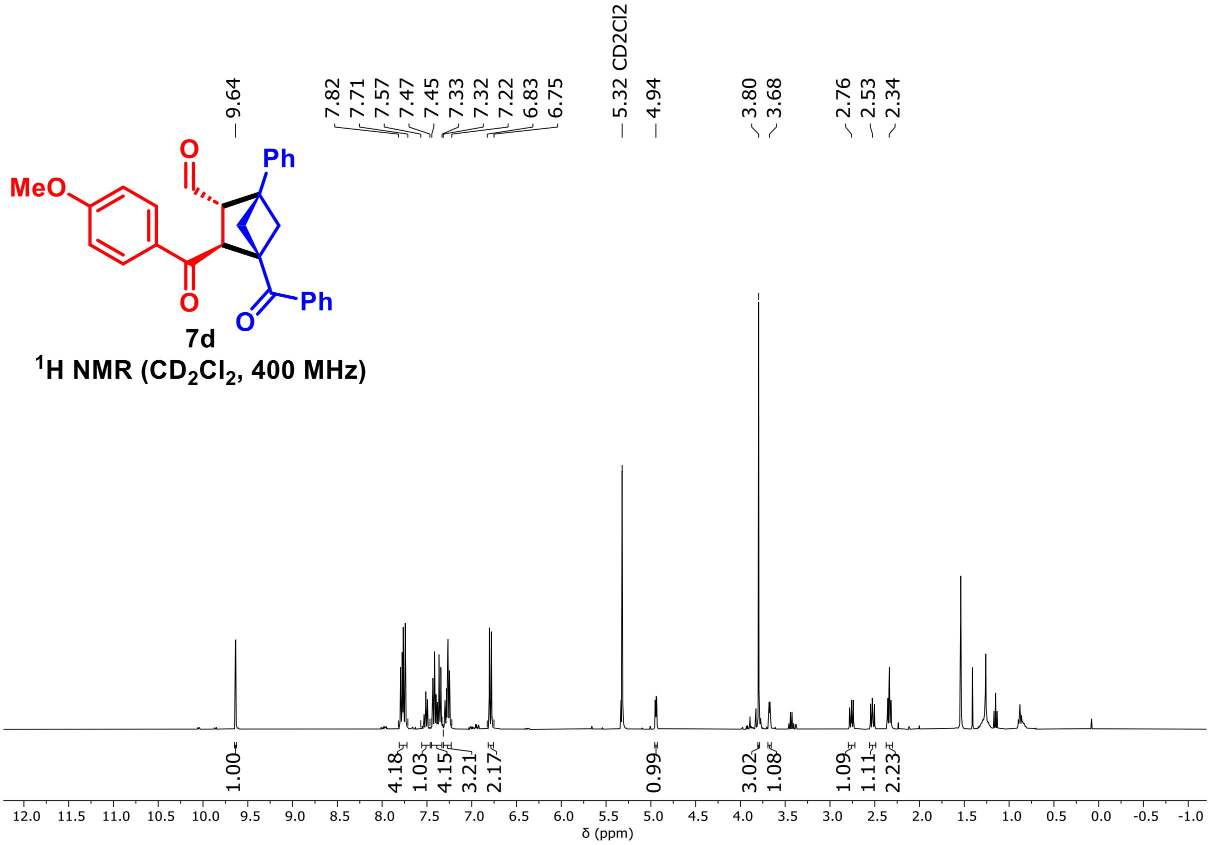
9.64
7.82 7.71 7.57 7.47 7.45 7.33 7.32 7.22 6.83 6.75
5.32 CD2Cl2
4.94
3.80 3.68
2.76 2.53 2.34
MeO
O
Ph
O
O
Ph
7d
^{1}H NMR (CD_2Cl_2, 400 MHz)
1.00 4.18 1.03 4.15 3.21 2.17 0.99 3.02 1.08 1.09 1.11 2.23
δ (ppm)

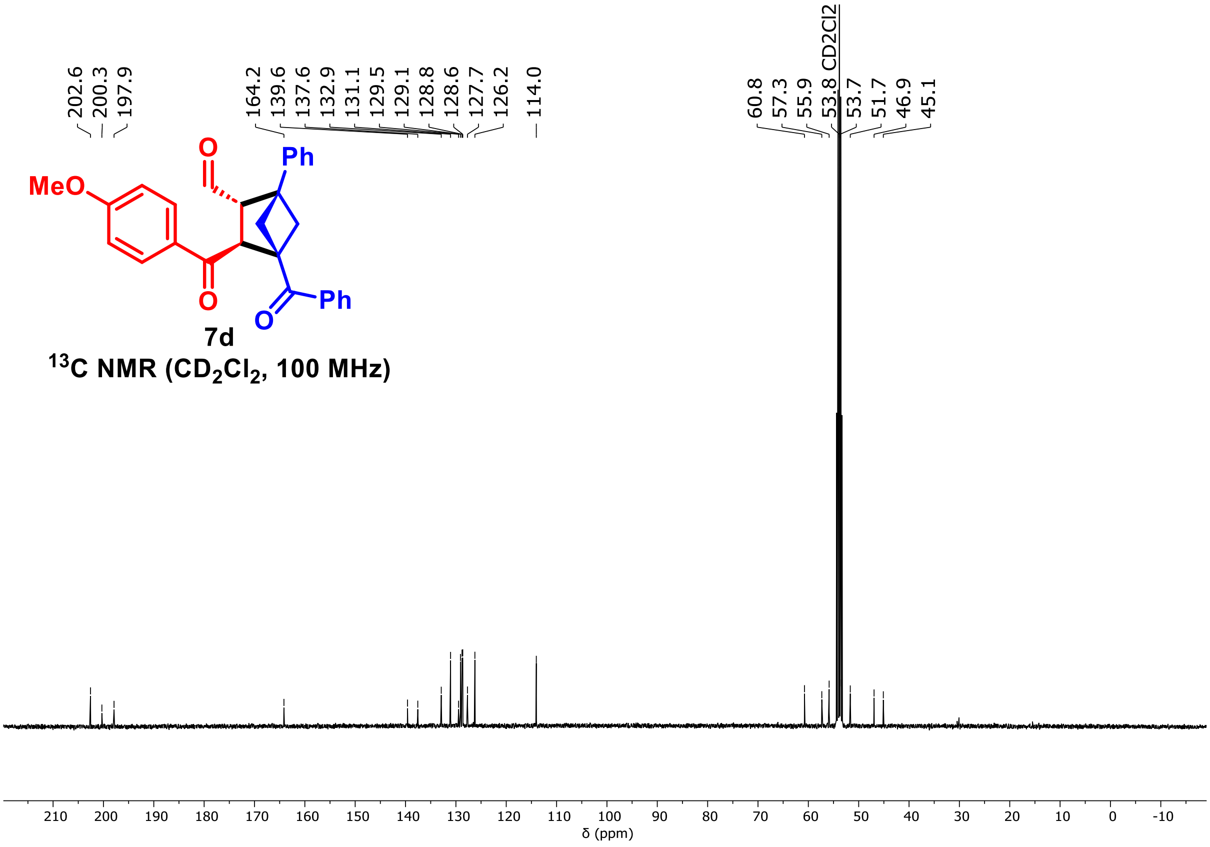
202.6 200.3 197.9
164.2 139.6 137.6 132.9 131.1 129.5 129.1 128.8 128.6 127.7 126.2
114.0
60.8 57.3 55.9 53.8 CD2Cl2 53.7 51.7 46.9 45.1
MeO
O
Ph
O
O
Ph
7d
^{13}C NMR (CD_2Cl_2, 100 MHz)
δ (ppm)

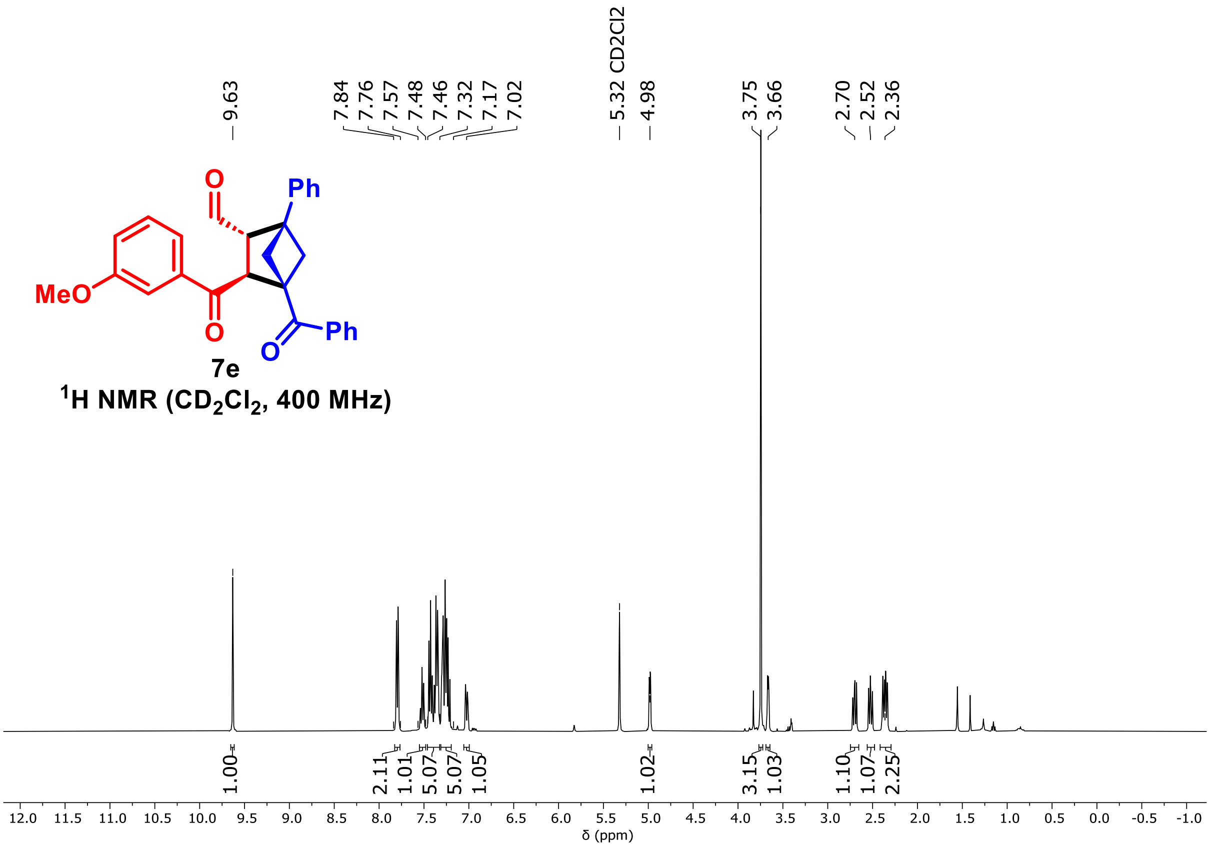
9.63
7.84
7.76
7.57
7.48
7.46
7.32
7.17
7.02
5.32 CD2Cl2
4.98
3.75
3.66
2.70
2.52
2.36
Ph
MeO
O
Ph
7e
1H NMR (CD2Cl2, 400 MHz)
1.00
2.11
1.01
5.07
5.07
1.05
1.02
3.15
1.03
1.10
1.07
2.25
12.0 11.5 11.0 10.5 10.0 9.5 9.0 8.5 8.0 7.5 7.0 6.5 6.0 5.5 5.0 4.5 4.0 3.5 3.0 2.5 2.0 1.5 1.0 0.5 0.0 -0.5 -1.0
δ (ppm)

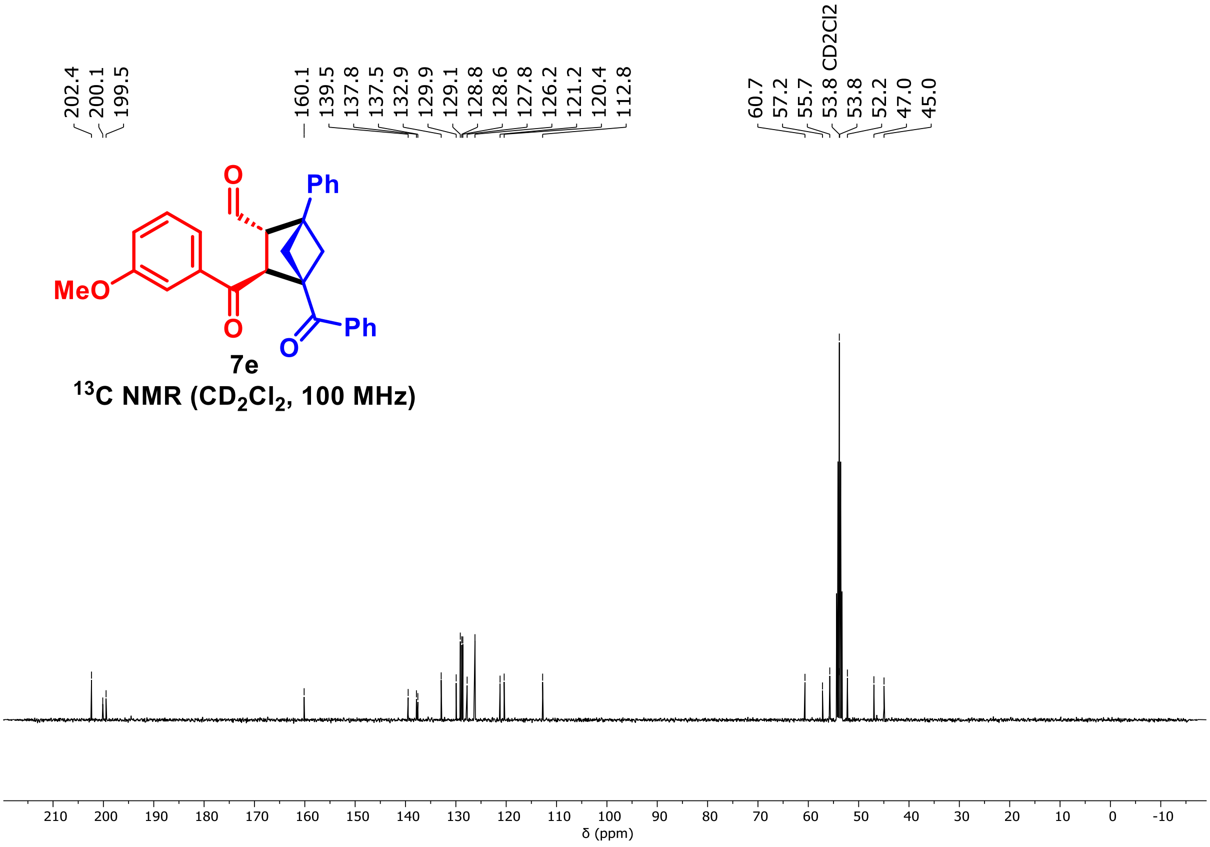
202.4
200.1
199.5
160.1
139.5
137.8
137.5
132.9
129.9
129.1
128.8
128.6
127.8
126.2
121.2
120.4
112.8
60.7
57.2
55.7
53.8 CD2Cl2
53.8
52.2
47.0
45.0
Ph
MeO
O
Ph
7e
13C NMR (CD2Cl2, 100 MHz)
210 200 190 180 170 160 150 140 130 120 110 100 90 80 70 60 50 40 30 20 10 0 -10
δ (ppm)

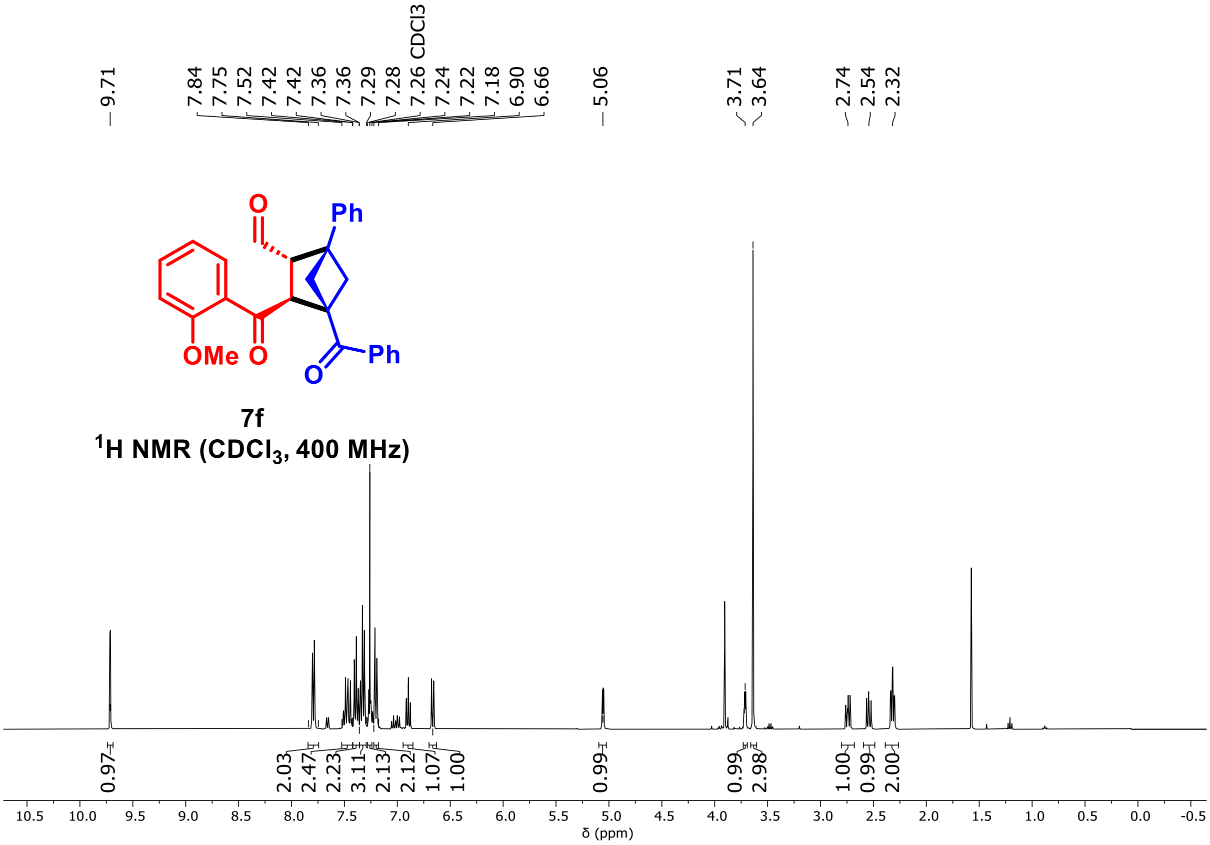

7f
^{1}H NMR ($CDCl_3$, 400 MHz)

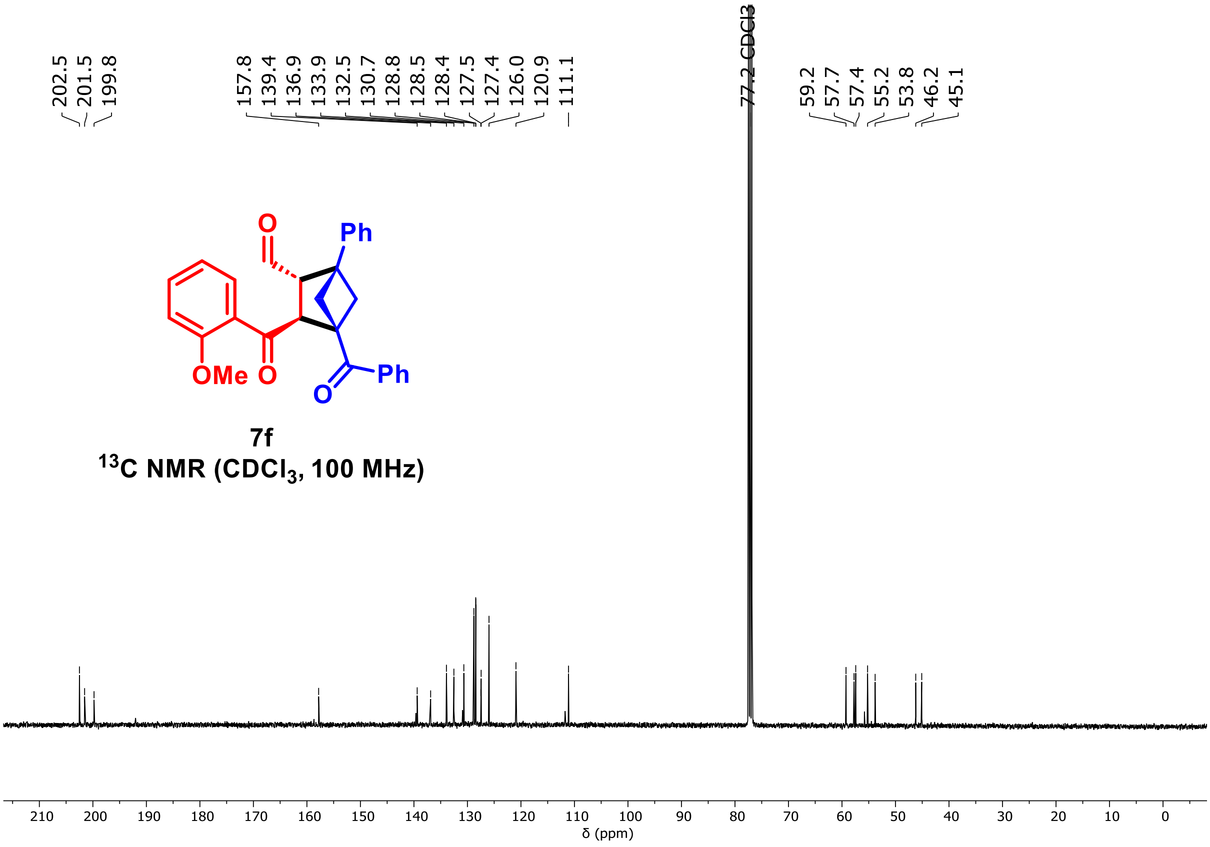

7f
^{13}C NMR ($CDCl_3$, 100 MHz)

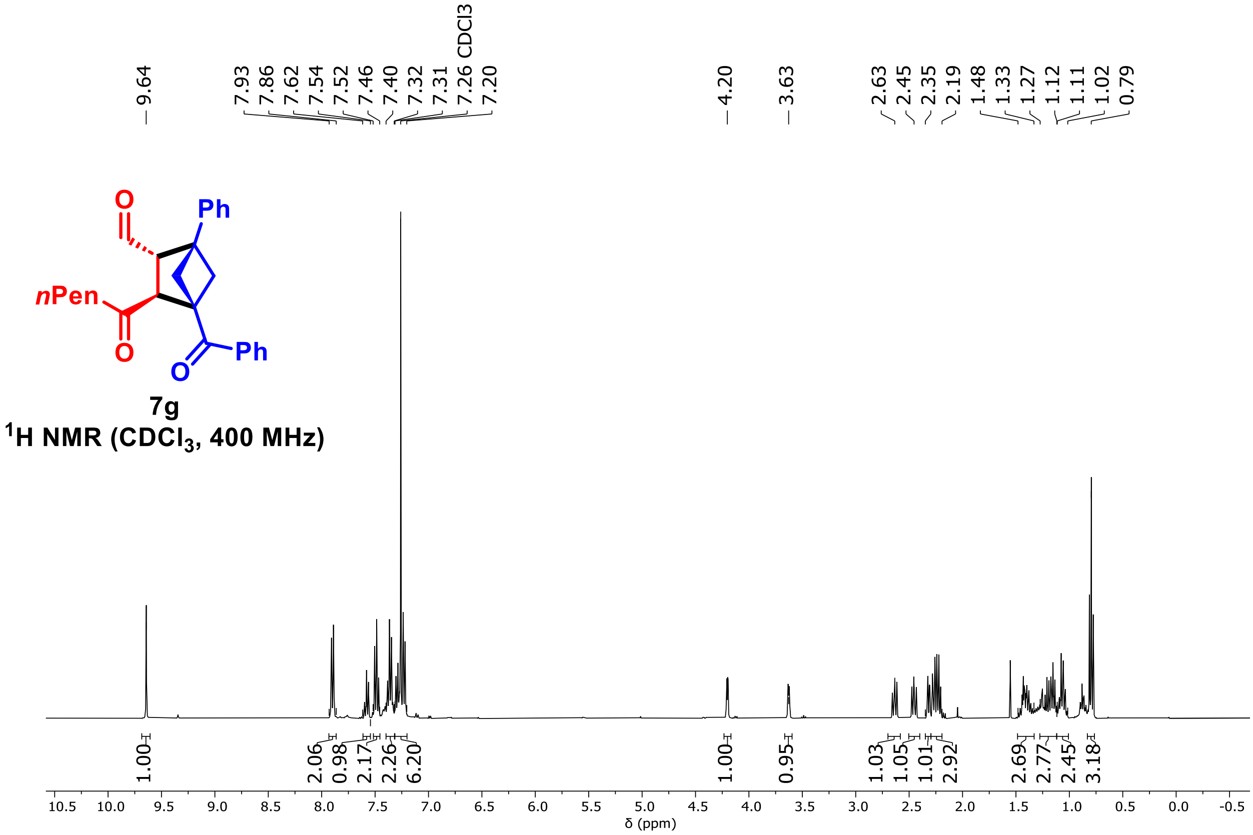
9.64
7.93 7.86 7.62 7.54 7.52 7.46 7.40 7.32 7.31 7.26 CDCl3 7.20
4.20
3.63
2.63 2.45 2.35 2.19 1.48 1.33 1.27 1.12 1.11 1.02 0.79
O
Ph
nPen
O
O
Ph
7g
1H NMR (CDCl3, 400 MHz)
1.00 2.06 0.98 2.17 2.26 6.20 1.00 0.95 1.03 1.05 1.01 2.92 2.69 2.77 2.45 3.18
10.5 10.0 9.5 9.0 8.5 8.0 7.5 7.0 6.5 6.0 5.5 5.0 4.5 4.0 3.5 3.0 2.5 2.0 1.5 1.0 0.5 0.0 -0.5
δ (ppm)

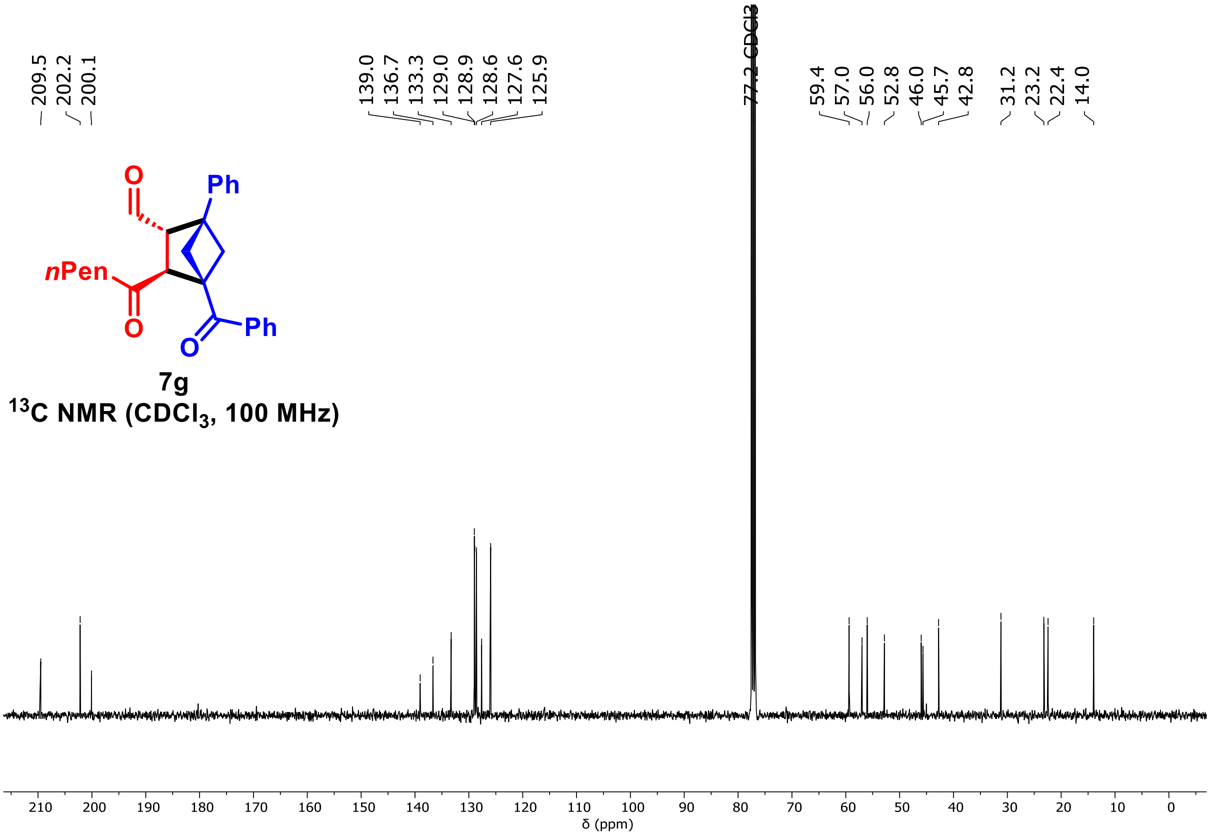
209.5 202.2 200.1
139.0 136.7 133.3 129.0 128.9 128.6 127.6 125.9
77.2 CDCl3
59.4 57.0 56.0 52.8 46.0 45.7 42.8 31.2 23.2 22.4 14.0
O
Ph
nPen
O
O
Ph
7g
13C NMR (CDCl3, 100 MHz)
210 200 190 180 170 160 150 140 130 120 110 100 90 80 70 60 50 40 30 20 10 0
δ (ppm)

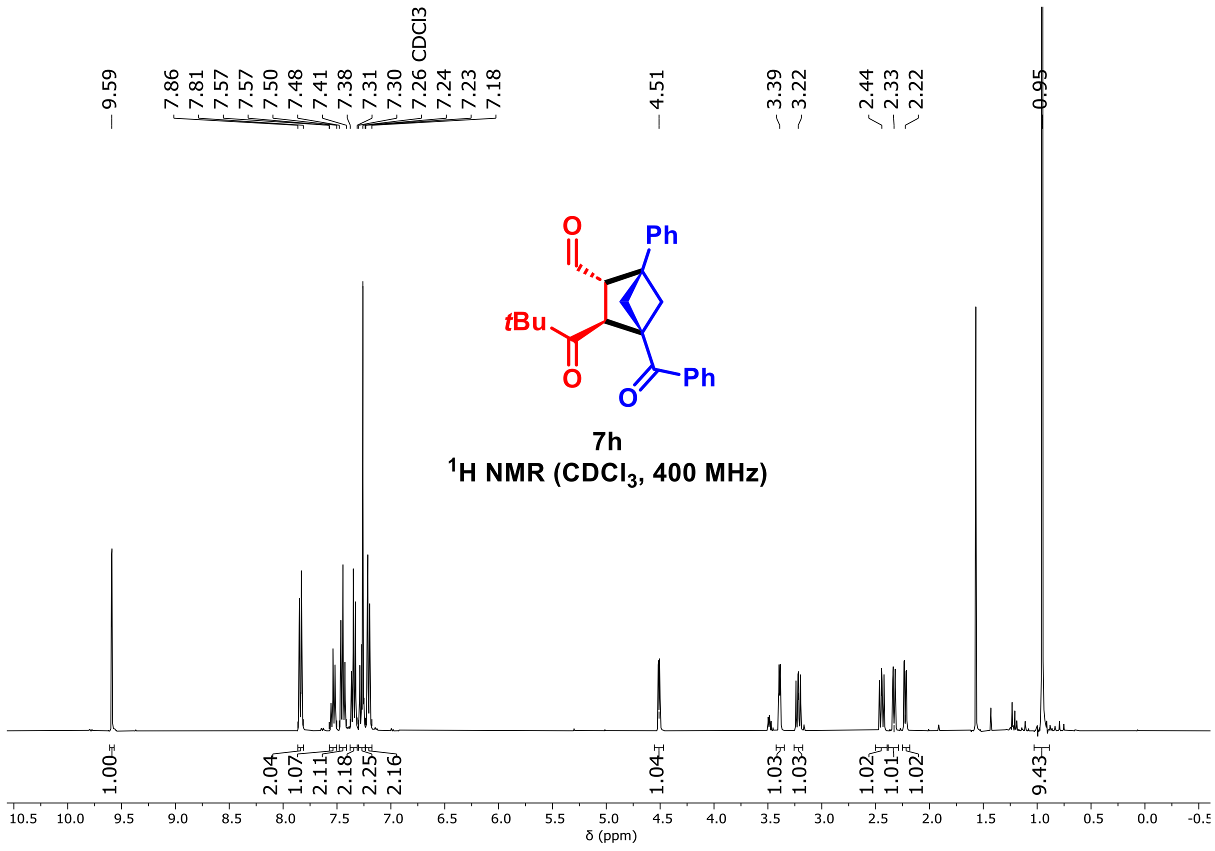

9.59
7.86
7.81
7.57
7.57
7.50
7.48
7.41
7.38
7.31
7.30
7.26 CDCl3
7.24
7.23
7.18
4.51
3.39
3.22
2.44
2.33
2.22
0.95
Ph
tBu
O
Ph
7h
1H NMR (CDCl3, 400 MHz)
1.00
2.04
1.07
2.11
2.18
2.25
2.16
1.04
1.03
1.03
1.02
1.01
1.02
9.43
δ (ppm)

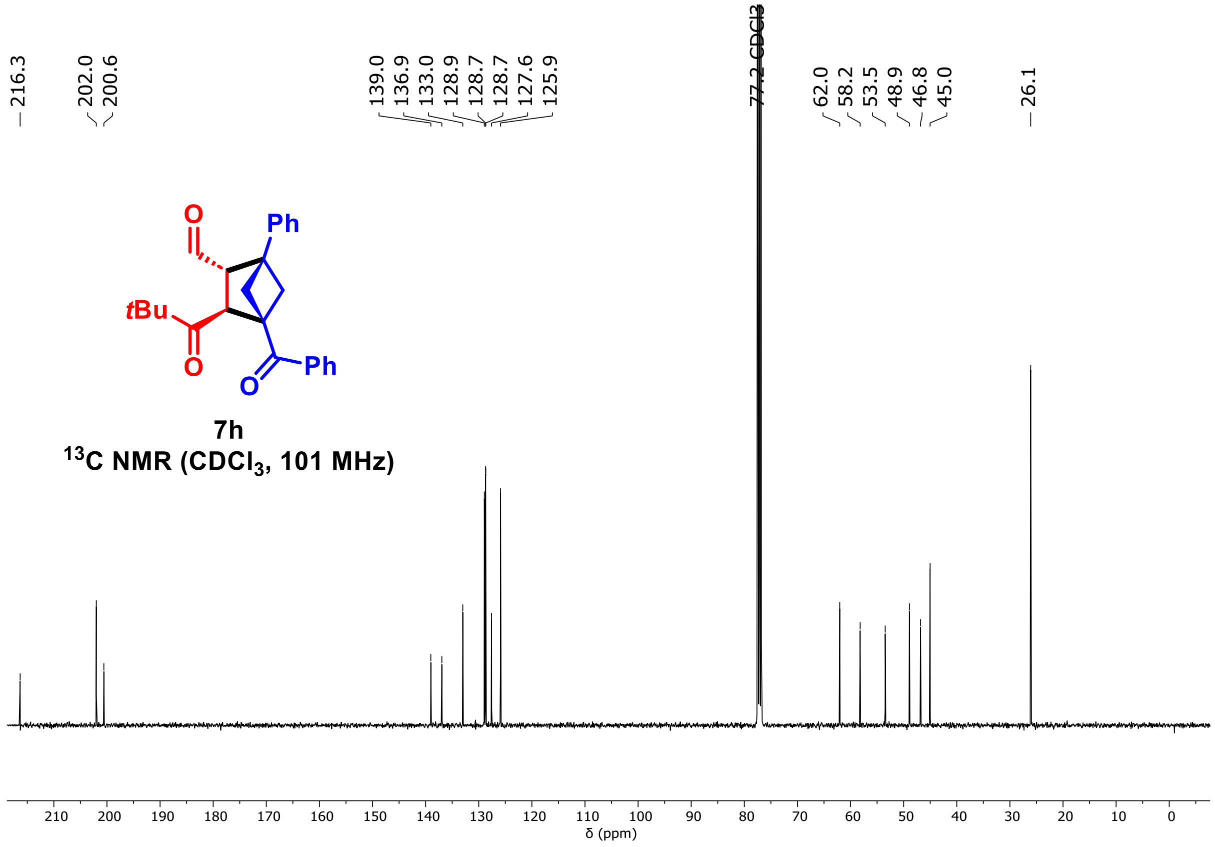

216.3
202.0
200.6
139.0
136.9
133.0
128.9
128.7
128.7
127.6
125.9
77.2 CDCl3
62.0
58.2
53.5
48.9
46.8
45.0
26.1
Ph
tBu
O
Ph
7h
13C NMR (CDCl3, 101 MHz)
δ (ppm)

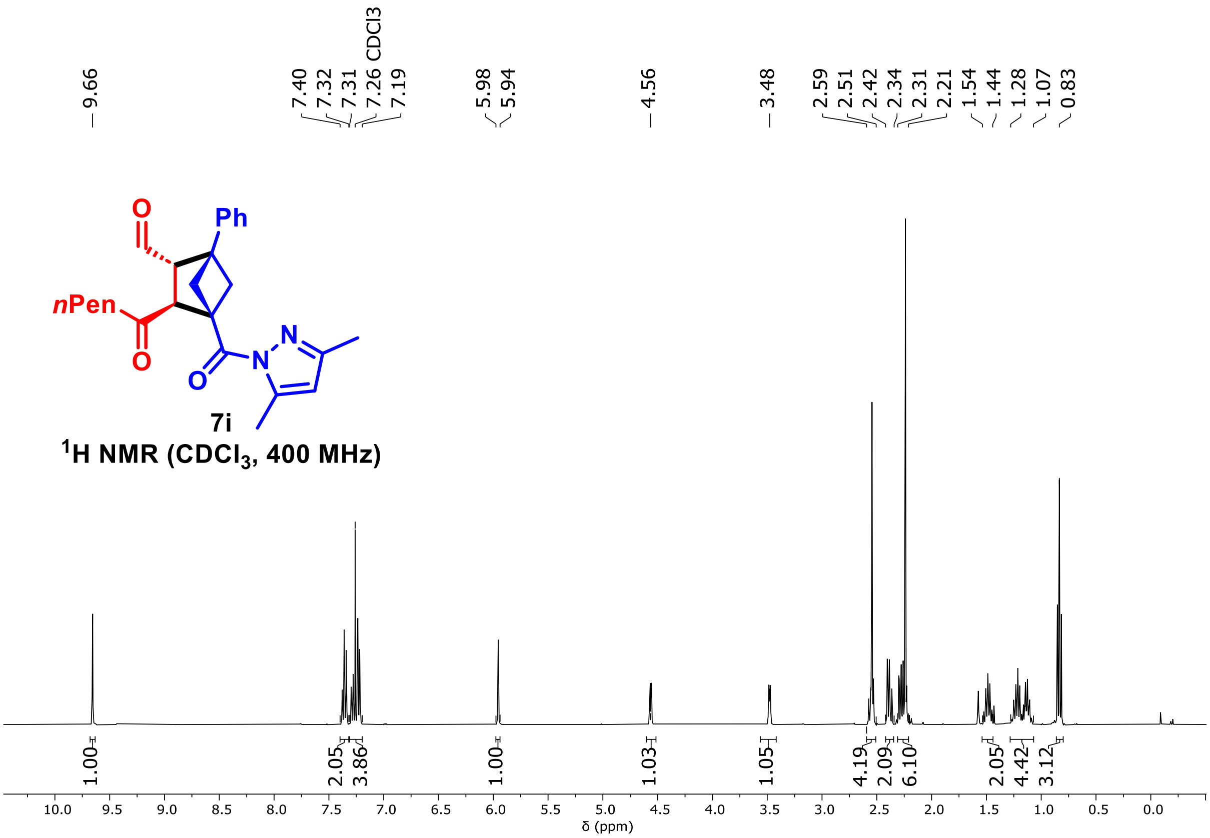
9.66
7.40
7.32
7.31
7.26 CDCl3
7.19
5.98
5.94
4.56
3.48
2.59
2.51
2.42
2.34
2.31
2.21
1.54
1.44
1.28
1.07
0.83
Ph
nPen
O
N
7i
1H NMR (CDCl3, 400 MHz)
1.00
2.05
3.86
1.00
1.03
1.05
4.19
2.09
6.10
2.05
4.42
3.12
δ (ppm)

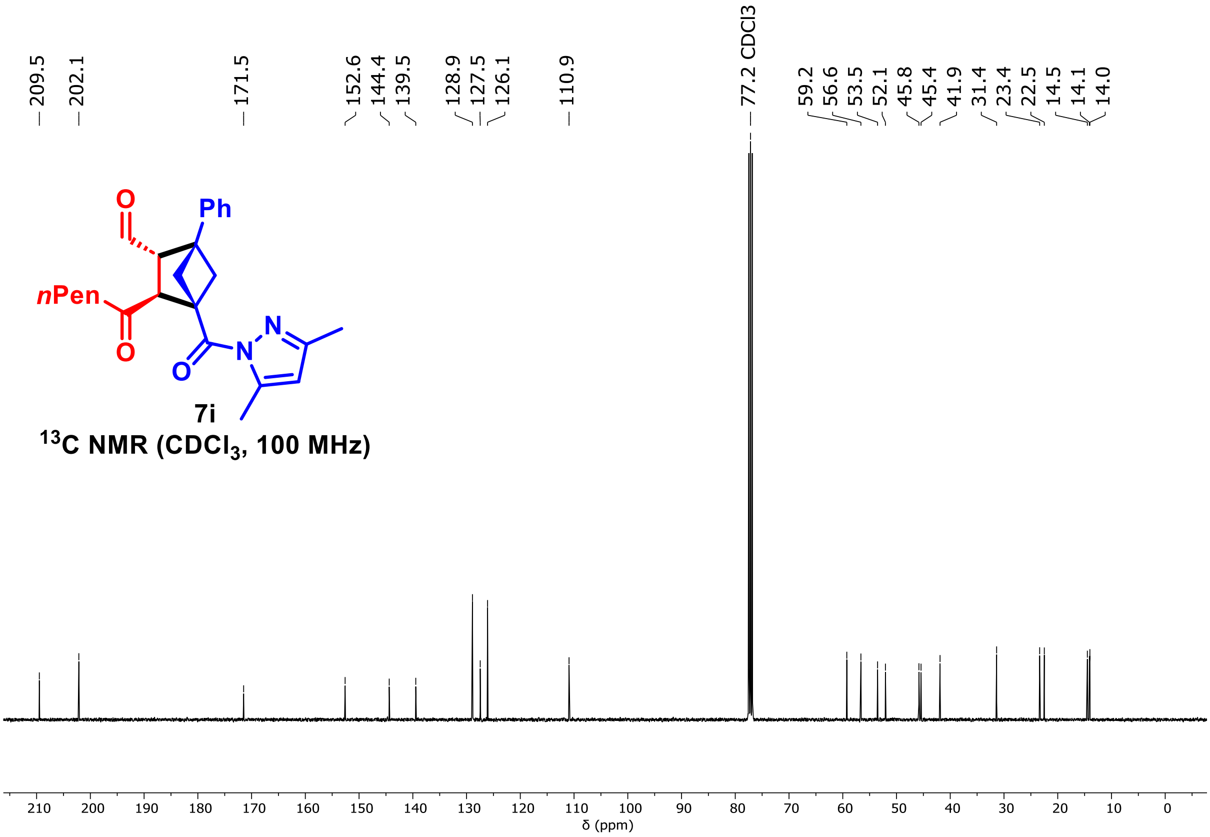
209.5
202.1
171.5
152.6
144.4
139.5
128.9
127.5
126.1
110.9
77.2 CDCl3
59.2
56.6
53.5
52.1
45.8
45.4
41.9
31.4
23.4
22.5
14.5
14.1
14.0
Ph
nPen
O
N
7i
13C NMR (CDCl3, 100 MHz)
δ (ppm)

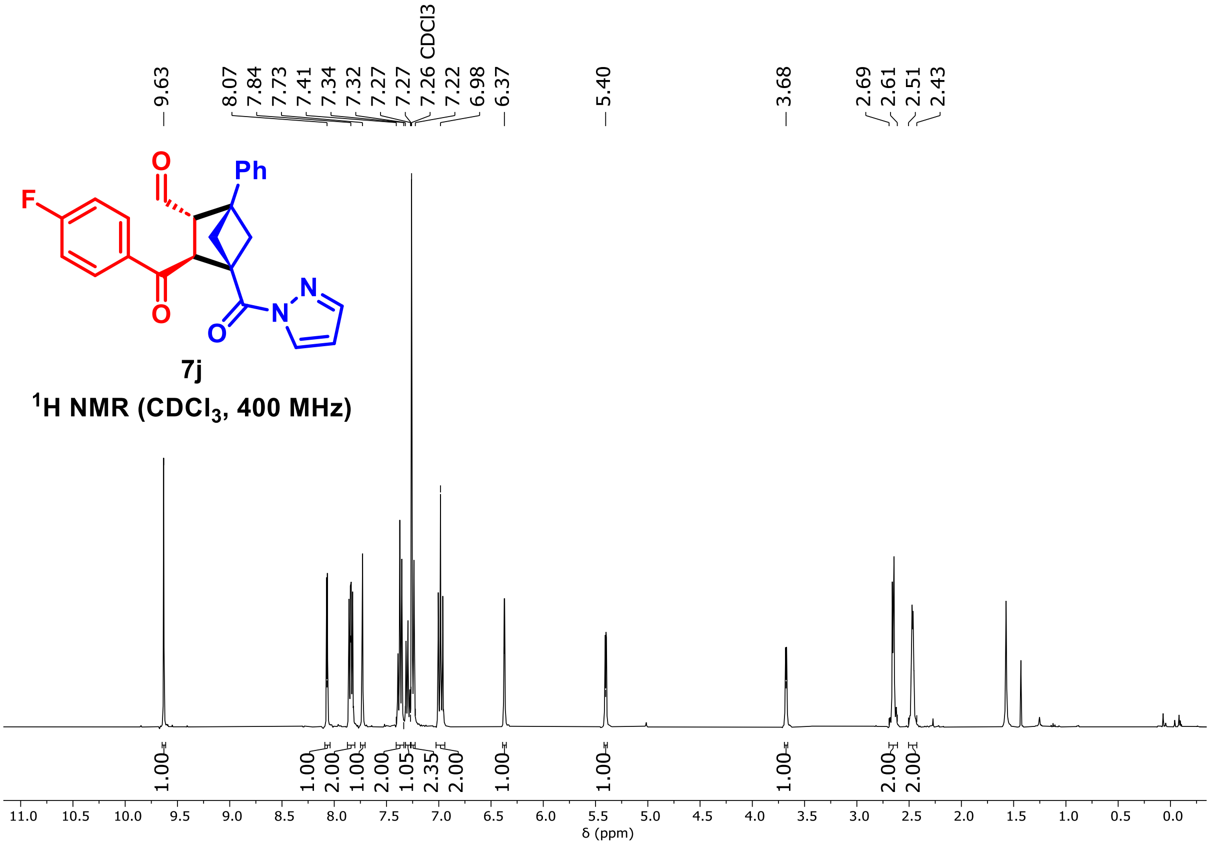
9.63
8.07
7.84
7.73
7.41
7.34
7.32
7.27
7.27
7.26 CDCl3
7.22
6.98
6.37
5.40
3.68
2.69
2.61
2.51
2.43
F
O
O
Ph
N
N
O
7j
1H NMR (CDCl3, 400 MHz)
1.00
1.00
2.00
1.00
2.00
1.05
2.35
2.00
1.00
1.00
1.00
2.00
2.00
δ (ppm)

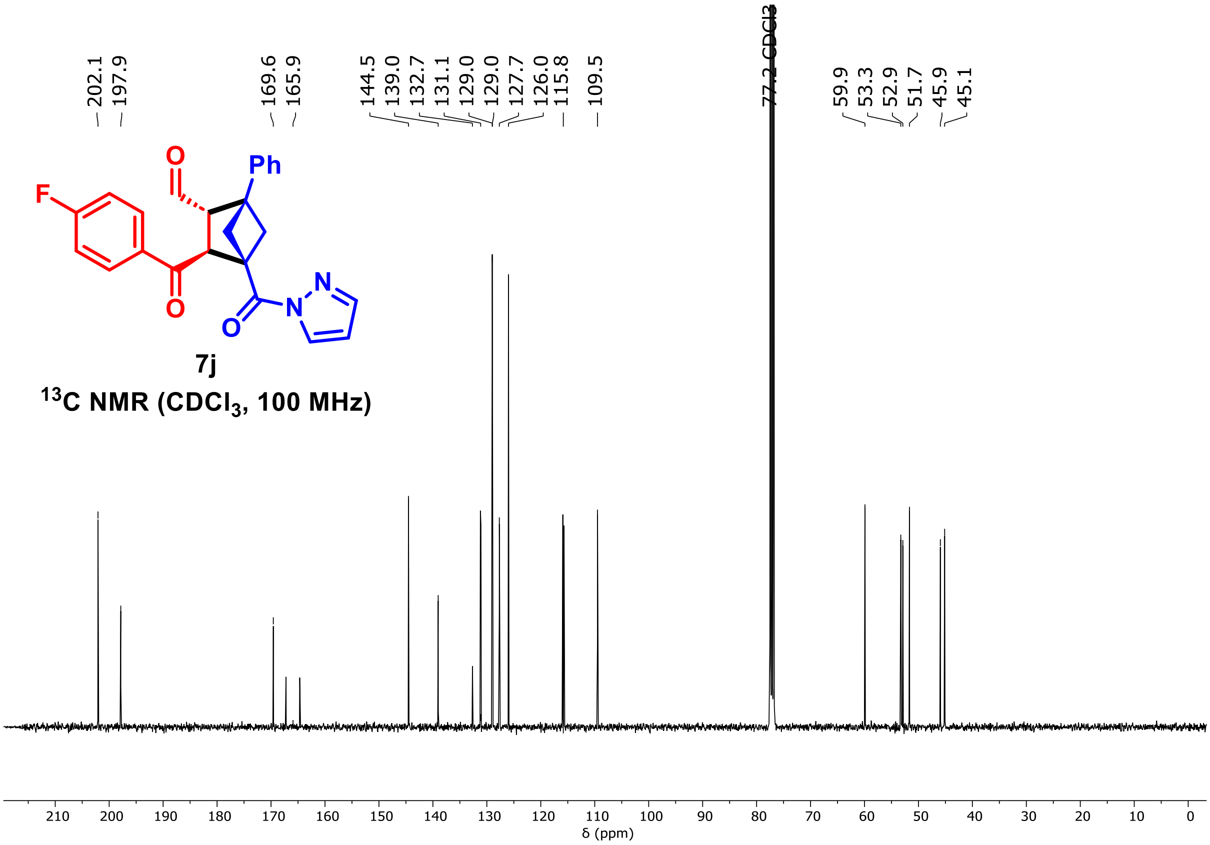
202.1
197.9
169.6
165.9
144.5
139.0
132.7
131.1
129.0
129.0
127.7
126.0
115.8
109.5
77.2 CDCl3
59.9
53.3
52.9
51.7
45.9
45.1
F
O
O
Ph
N
N
O
7j
13C NMR (CDCl3, 100 MHz)
δ (ppm)

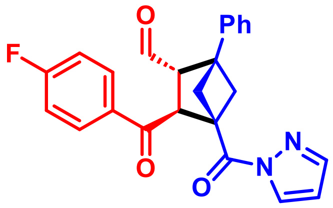

7j

^{19}F NMR (CDCl$_3$, 376 MHz)

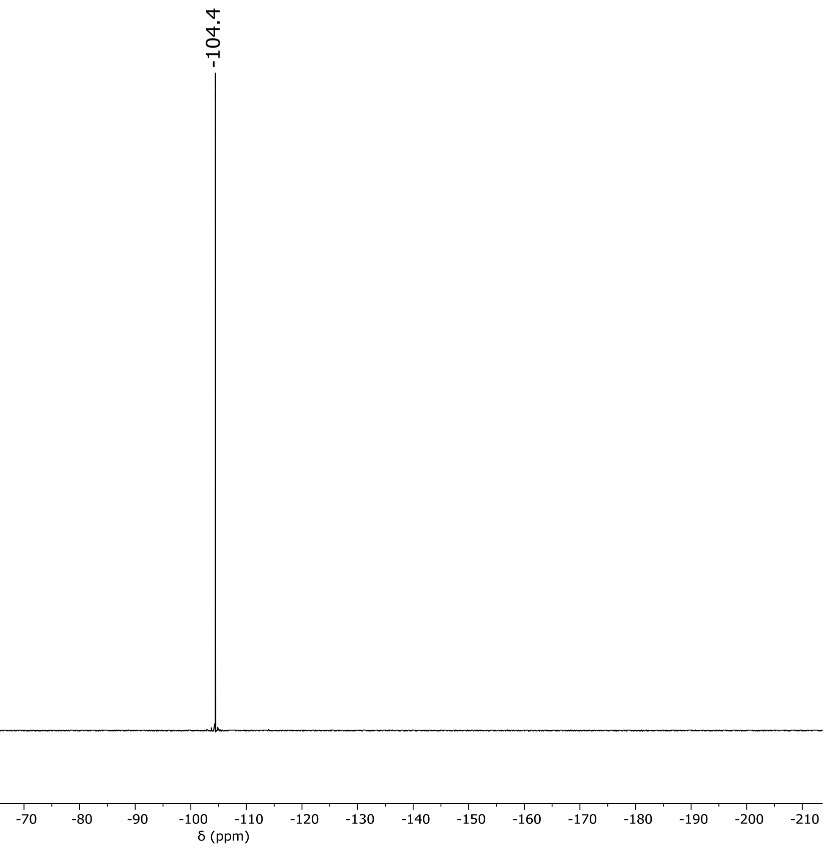

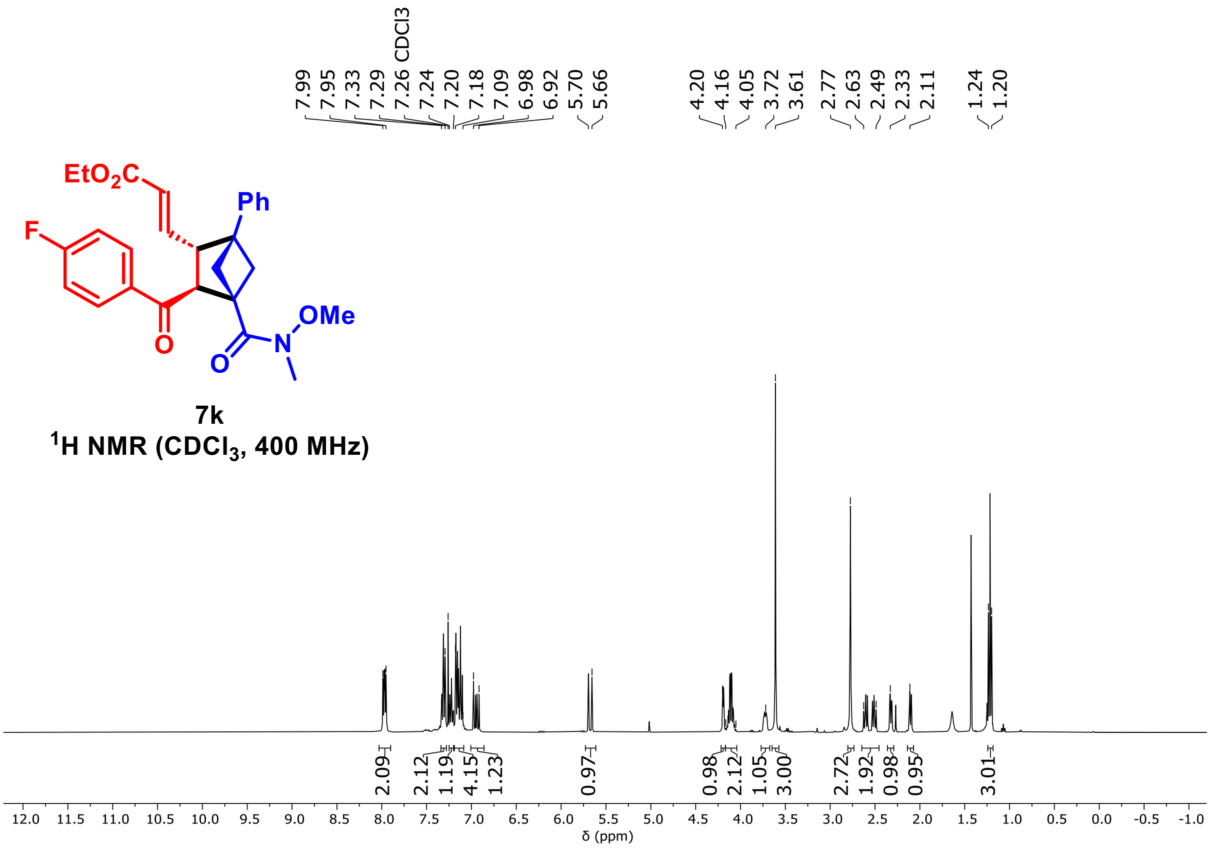

7k
^{1}H NMR ($CDCl_3$, 400 MHz)

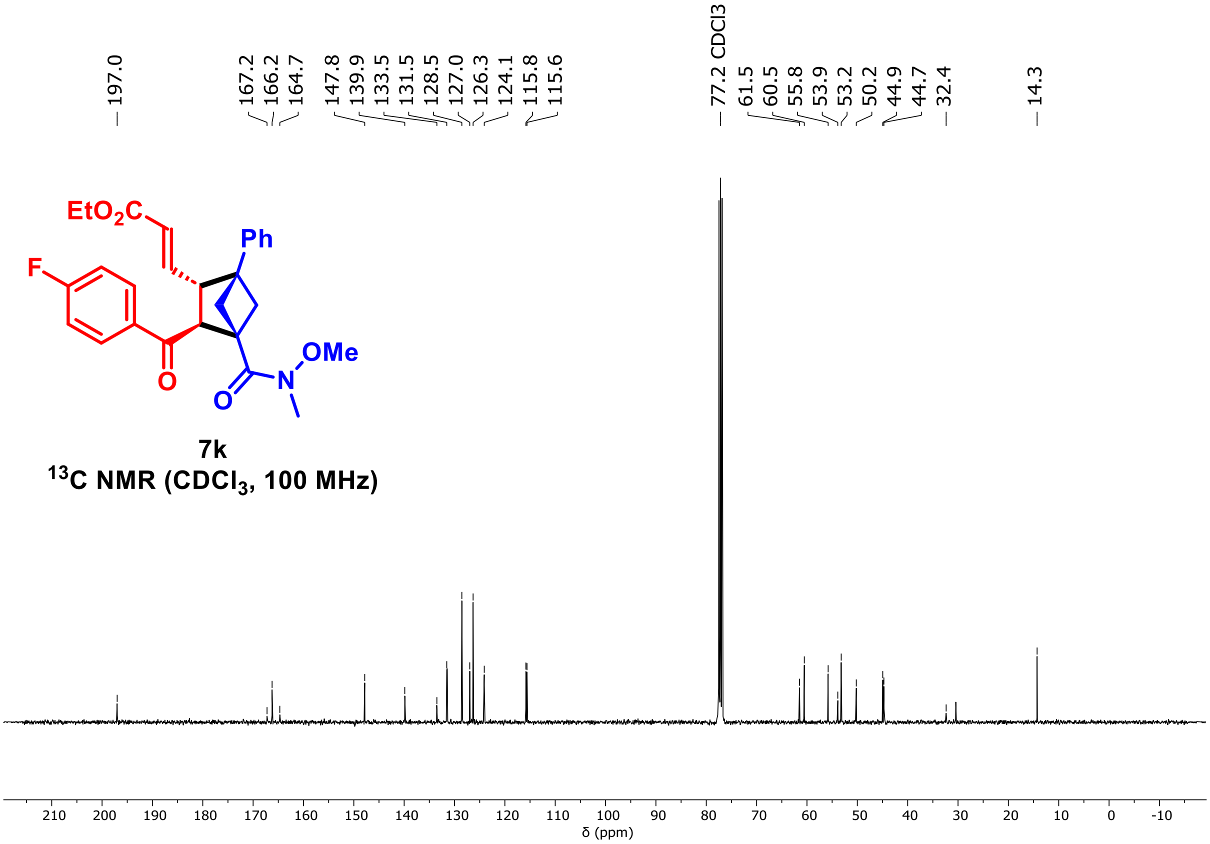

7k
^{13}C NMR ($CDCl_3$, 100 MHz)

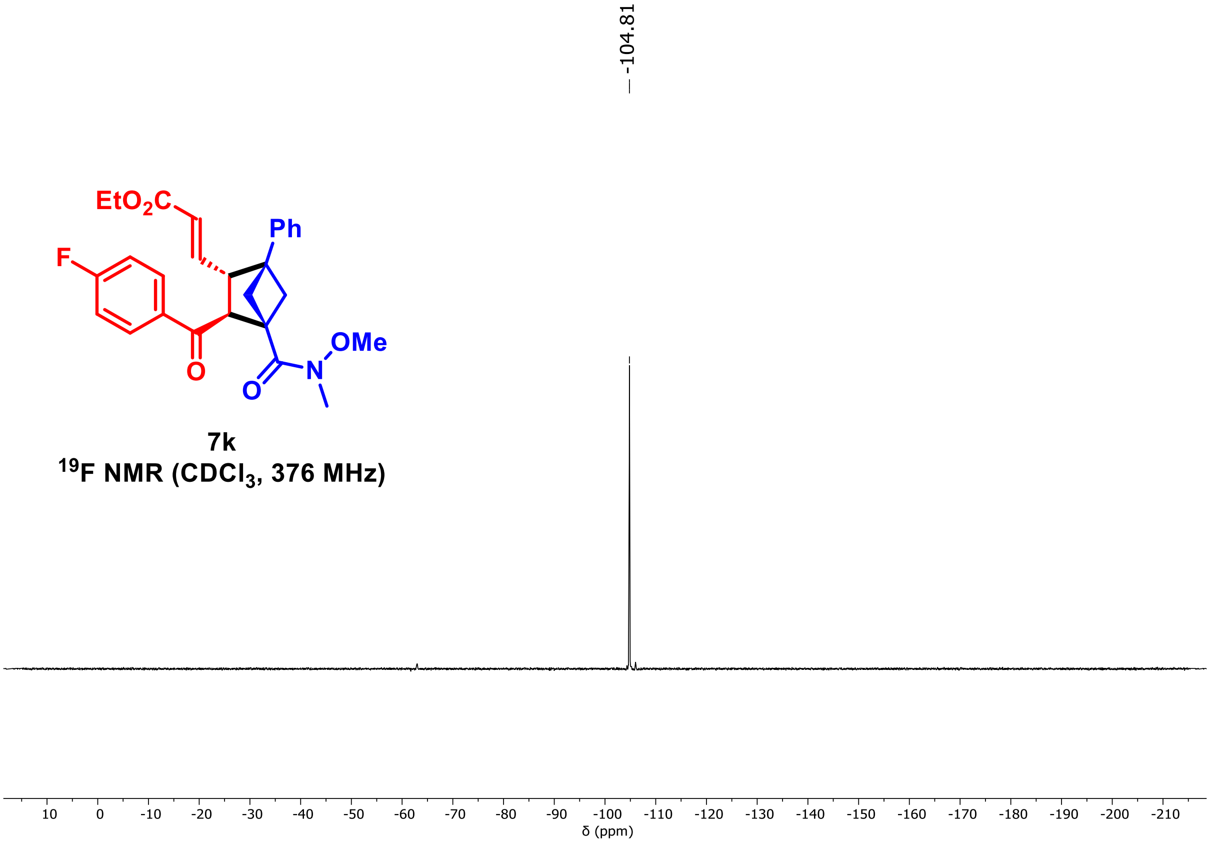

7k
^{19}F NMR ($CDCl_3$, 376 MHz)

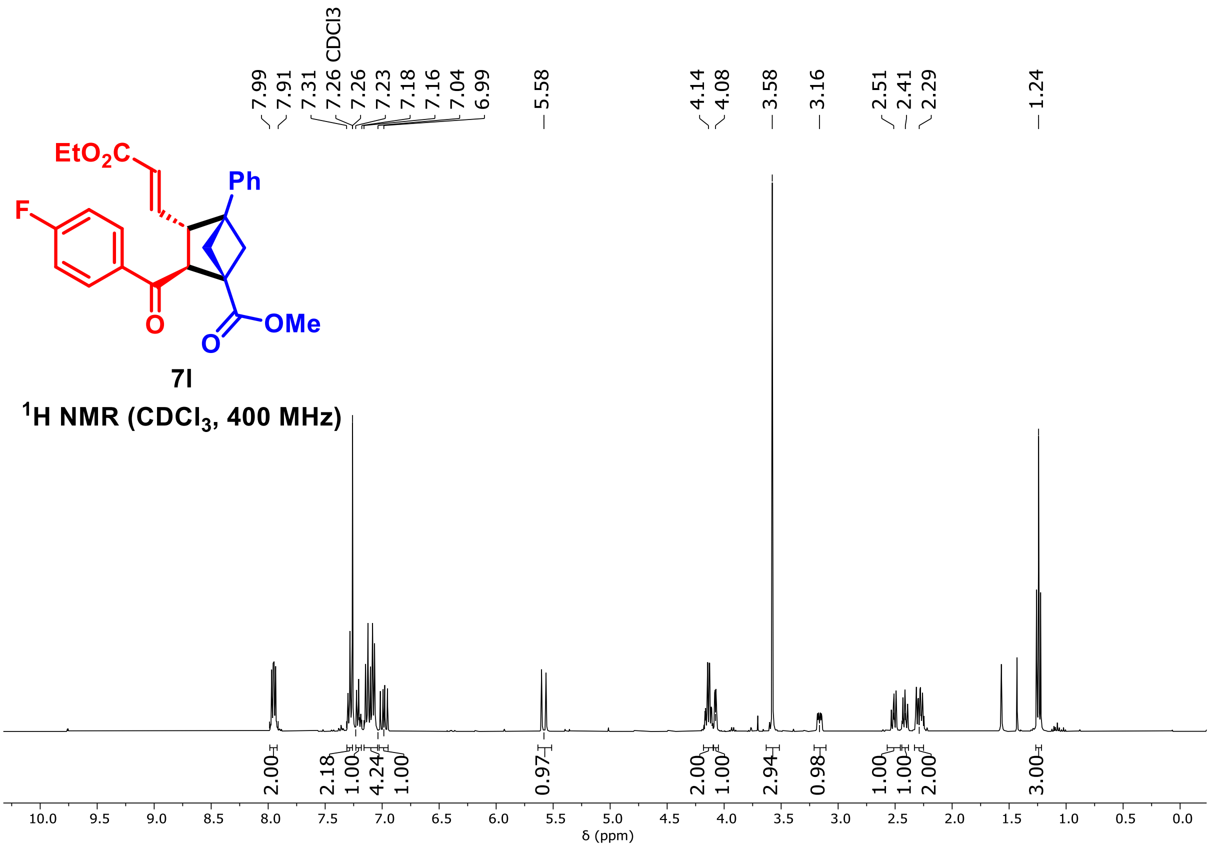
7.99
7.91
7.31
7.26 CDCl3
7.26
7.23
7.18
7.16
7.04
6.99
5.58
4.14
4.08
3.58
3.16
2.51
2.41
2.29
1.24
EtO2C
Ph
F
O
OMe
O
7l
1H NMR (CDCl3, 400 MHz)
2.00
2.18
1.00
4.24
1.00
0.97
2.00
1.00
2.94
0.98
1.00
1.00
2.00
3.00
δ (ppm)

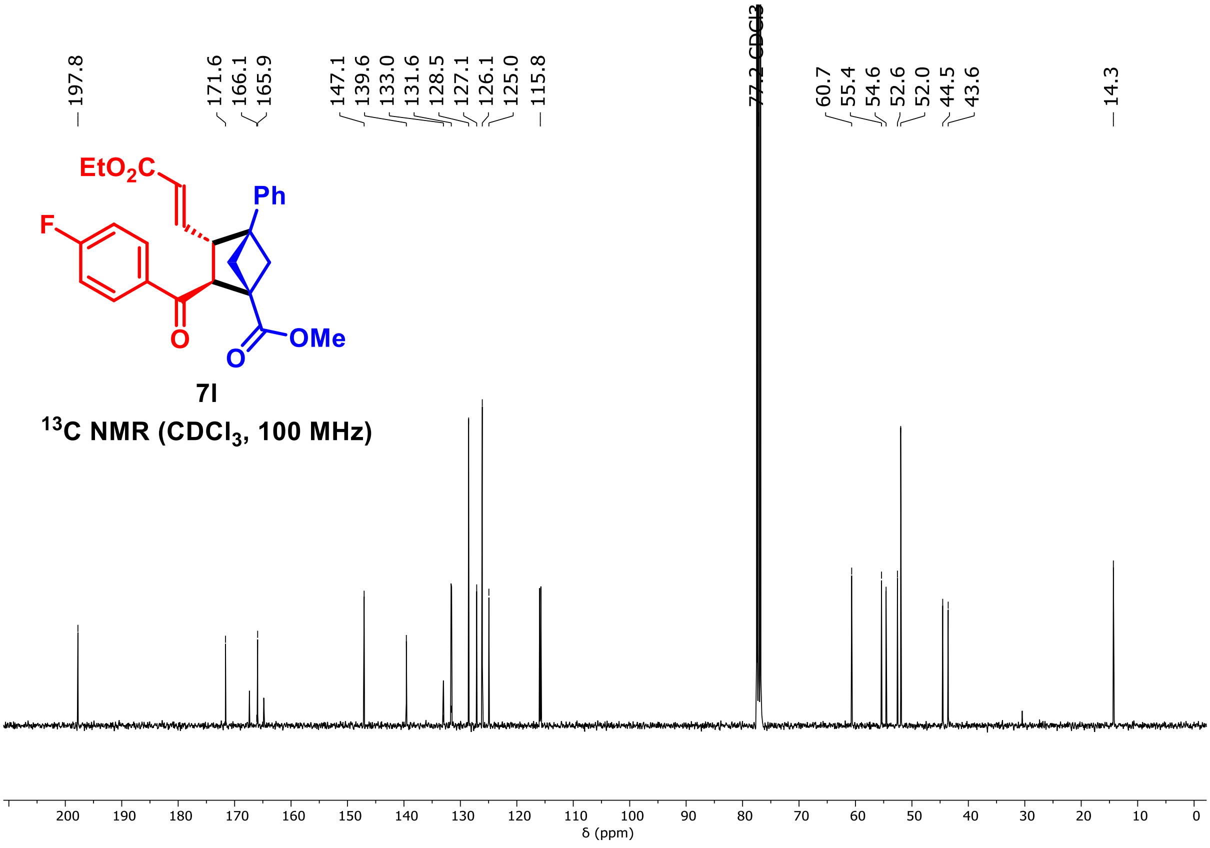
197.8
171.6
166.1
165.9
147.1
139.6
133.0
131.6
128.5
127.1
126.1
125.0
115.8
77.2 CDCl3
60.7
55.4
54.6
52.6
52.0
44.5
43.6
14.3
EtO2C
Ph
F
O
OMe
O
7l
13C NMR (CDCl3, 100 MHz)
δ (ppm)

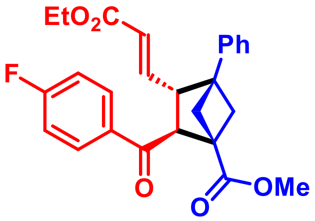

7l

^{19}F NMR ($CDCl_3$, 376 MHz)

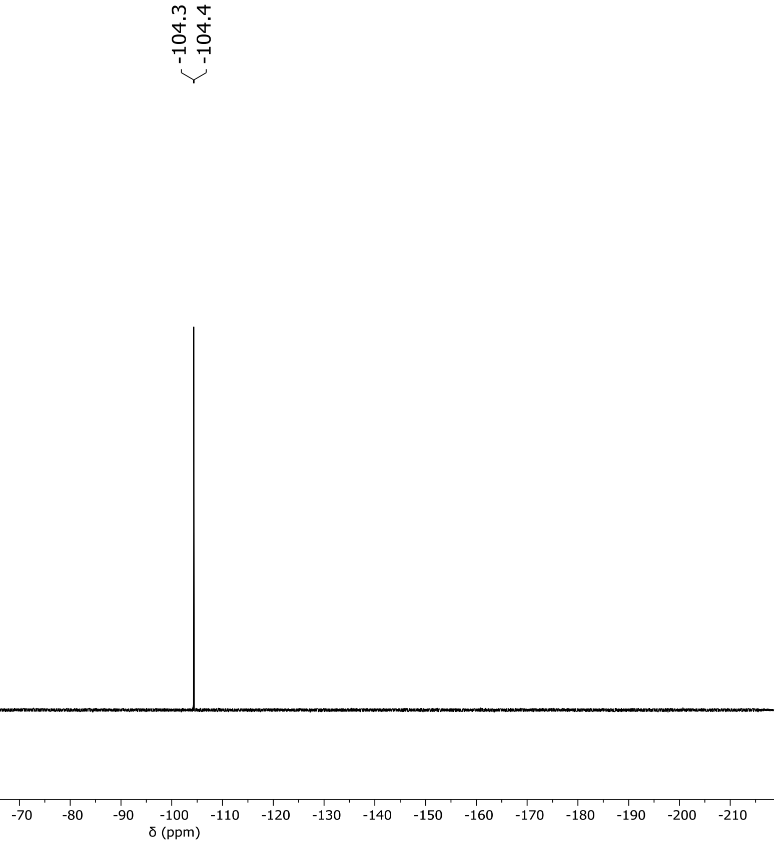

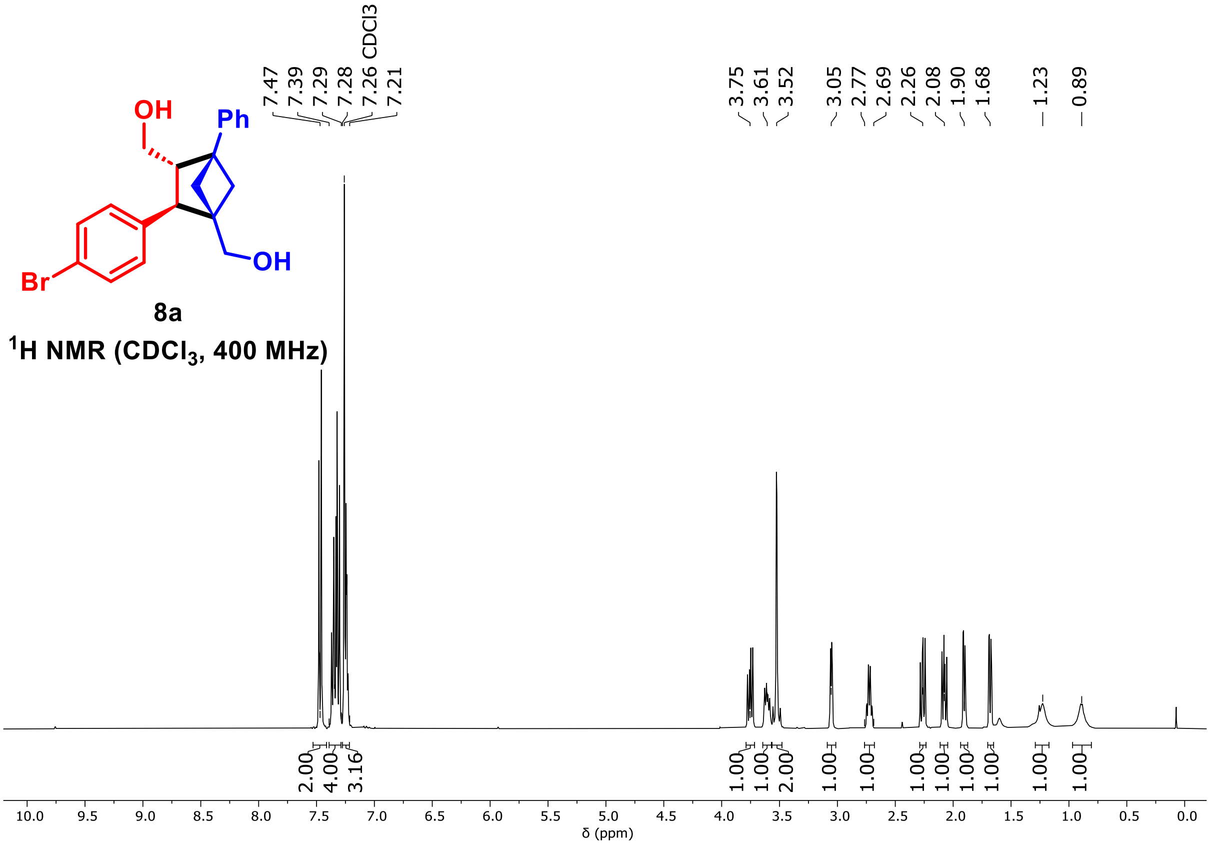

8a

^{1}H NMR ($CDCl_3$, 400 MHz)

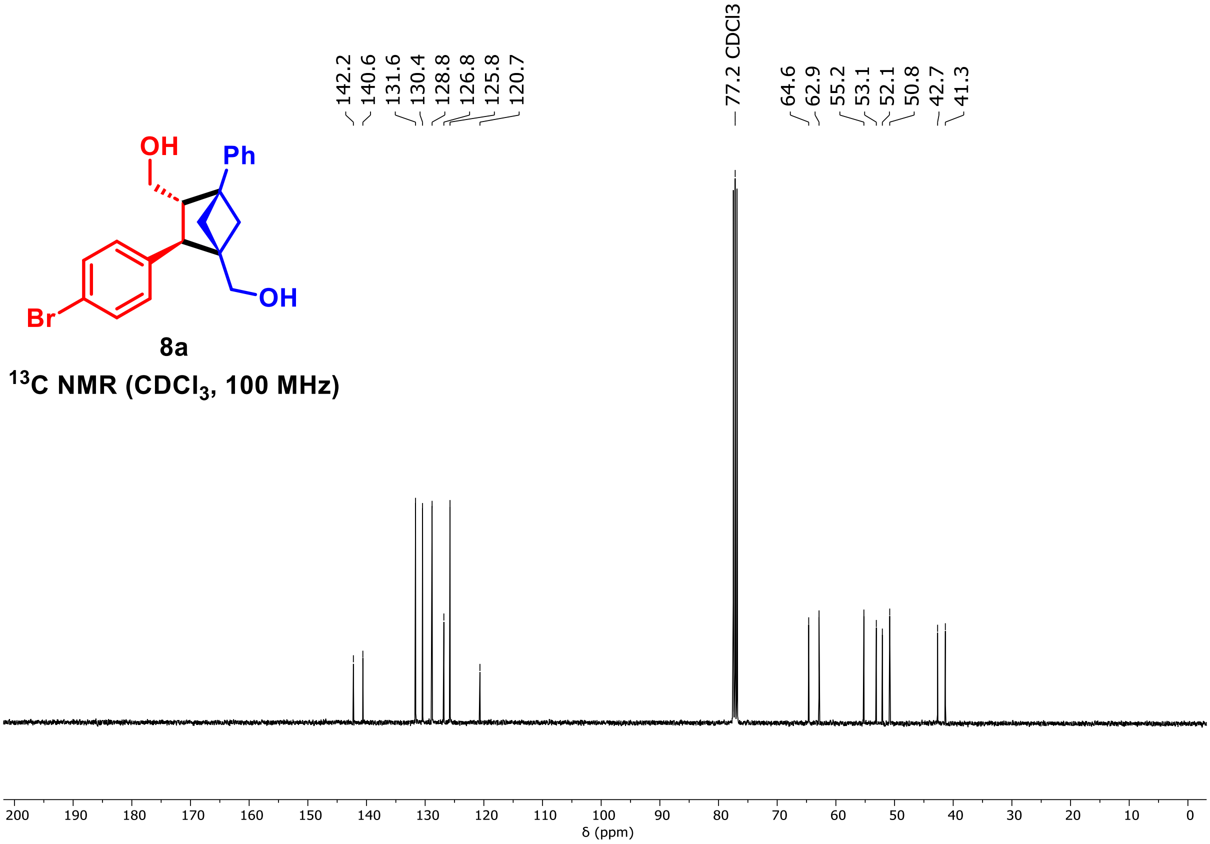

8a

^{13}C NMR ($CDCl_3$, 100 MHz)

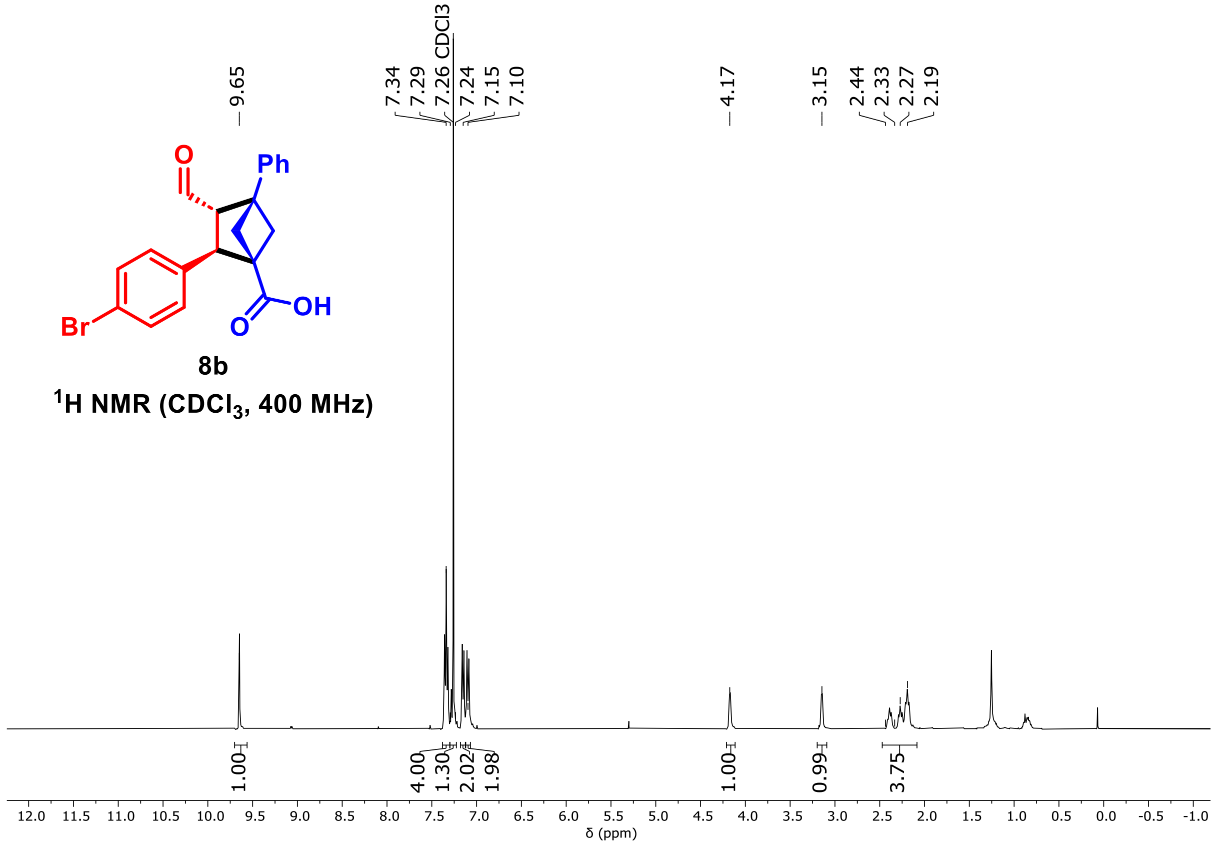
9.65
7.34
7.29
7.26 CDCl3
7.24
7.15
7.10
4.17
3.15
2.44
2.33
2.27
2.19
O
Ph
Br
O
OH
8b
^{1}H NMR (CDCl$_3$, 400 MHz)
1.00
4.00
1.30
2.02
1.98
1.00
0.99
3.75
δ (ppm)

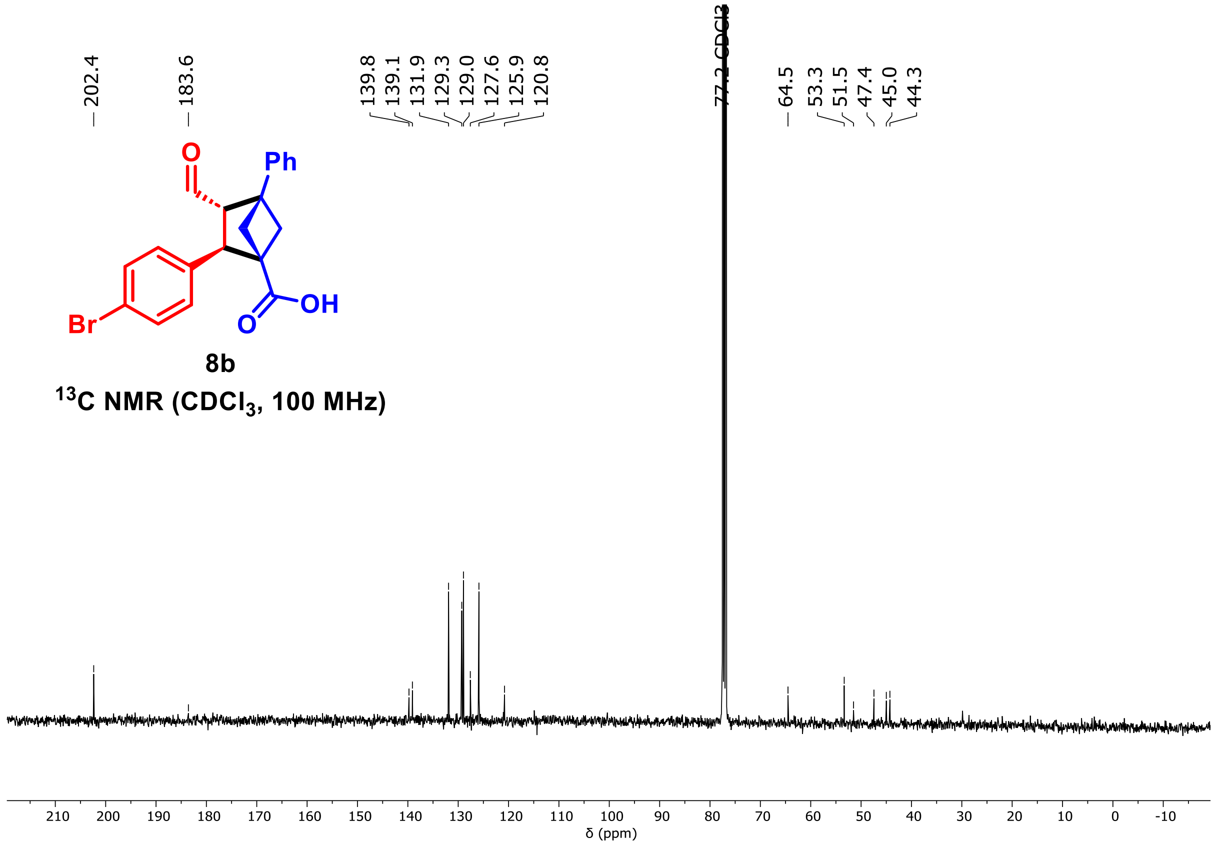
202.4
183.6
139.8
139.1
131.9
129.3
129.0
127.6
125.9
120.8
77.2 CDCl3
64.5
53.3
51.5
47.4
45.0
44.3
O
Ph
Br
O
OH
8b
^{13}C NMR (CDCl$_3$, 100 MHz)
δ (ppm)

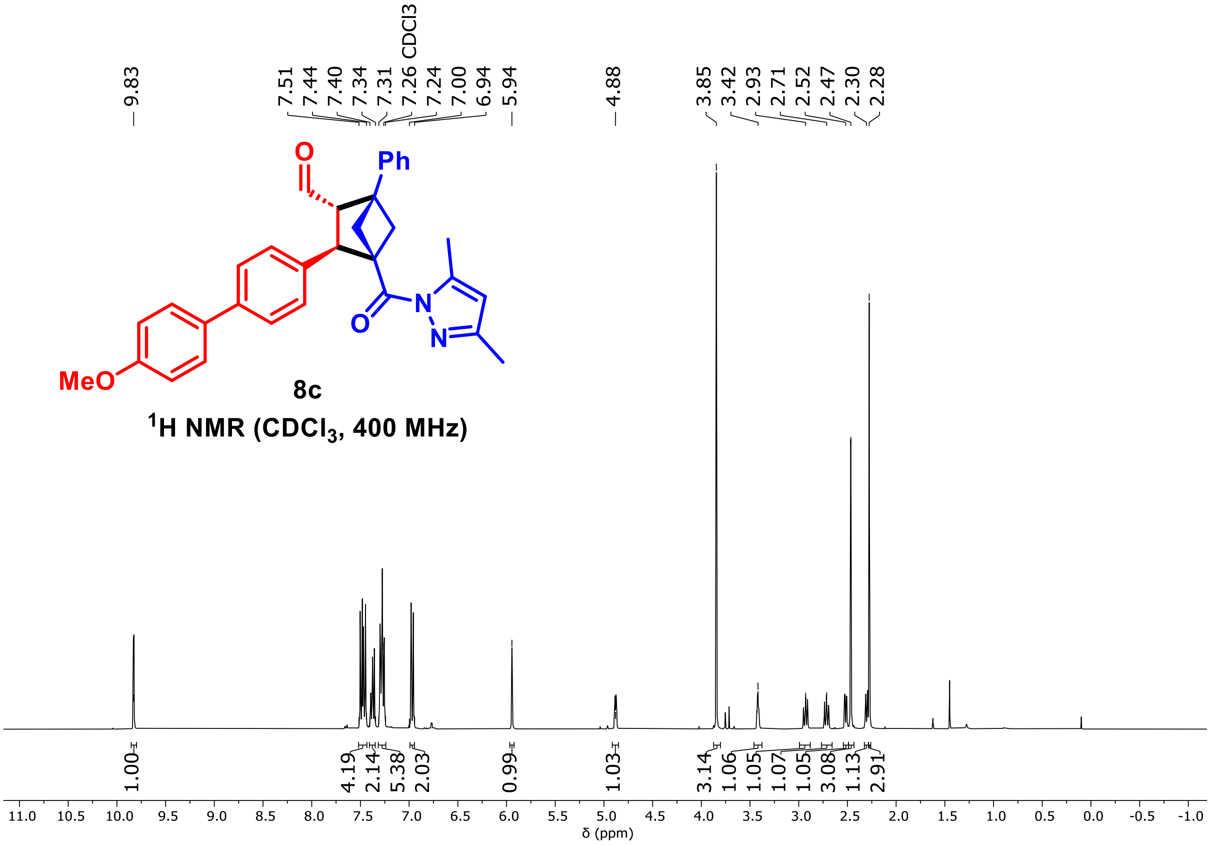

8c

^{1}H NMR ($CDCl_3$, 400 MHz)

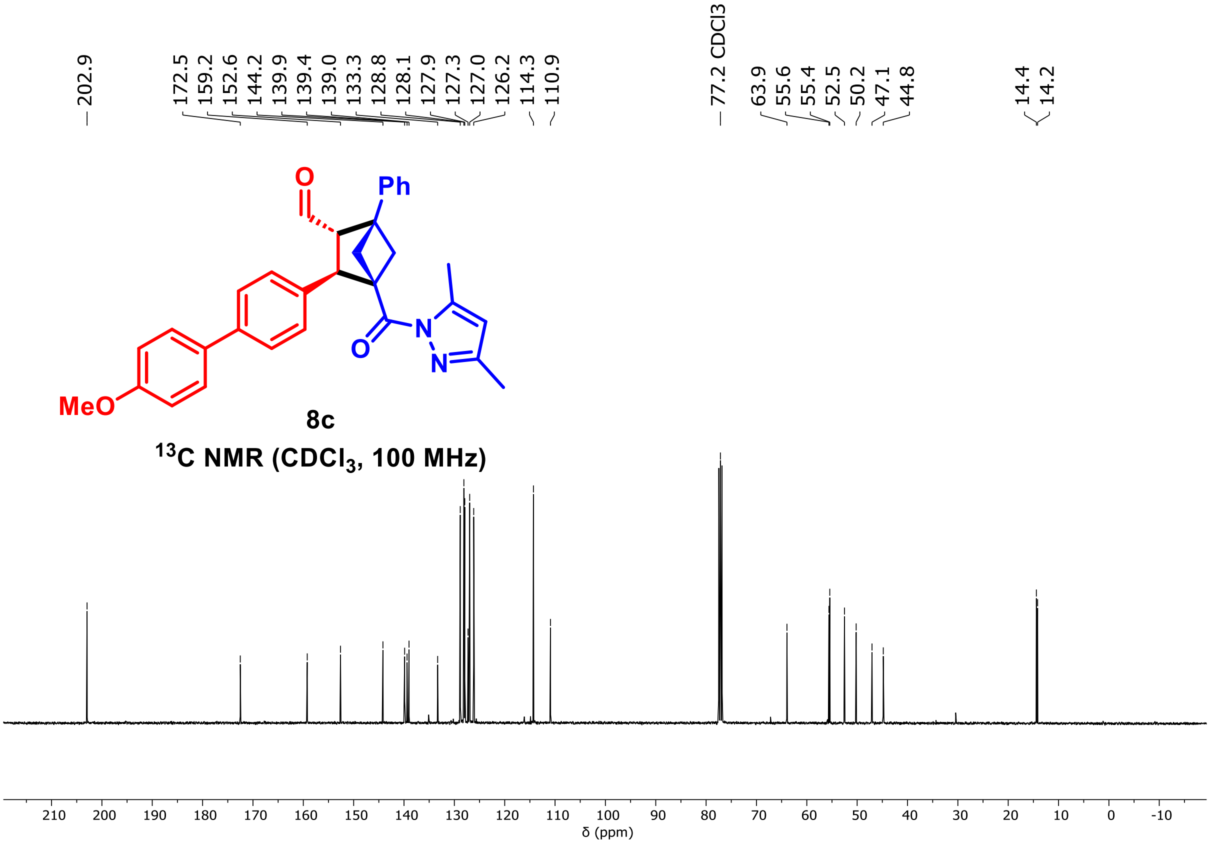

8c

^{13}C NMR ($CDCl_3$, 100 MHz)

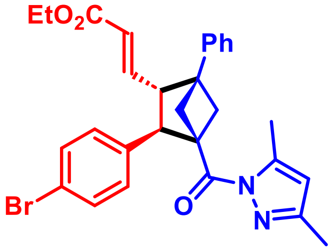

8d

^{1}H NMR ($CDCl_3$, 400 MHz)

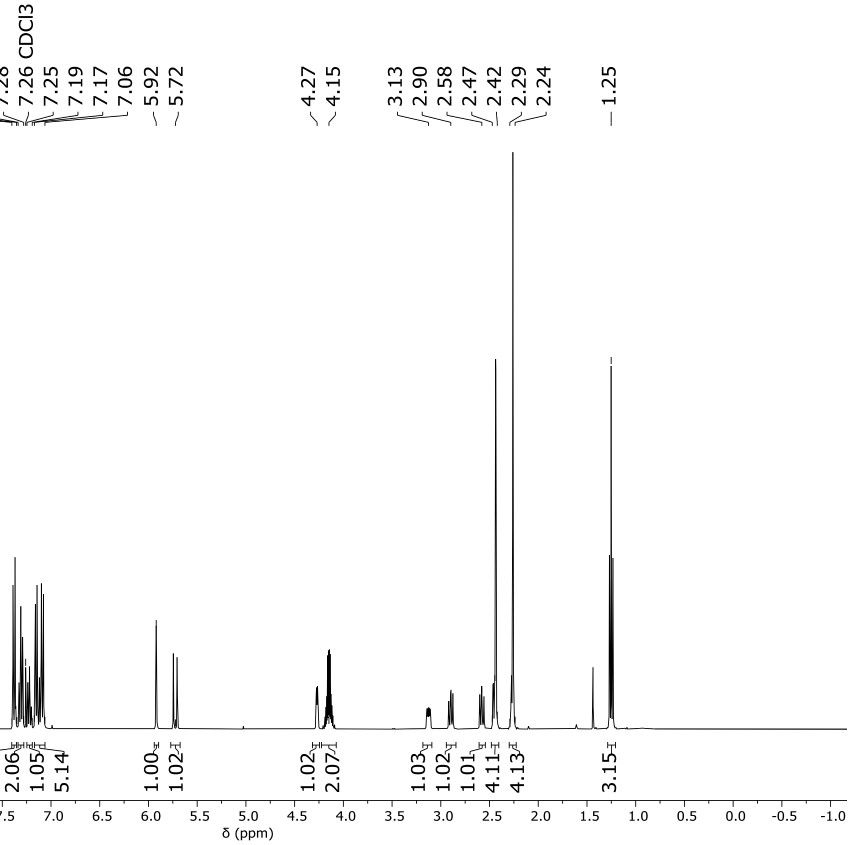

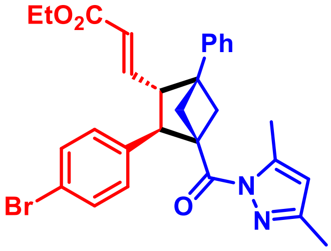

8d

^{13}C NMR ($CDCl_3$, 100 MHz)

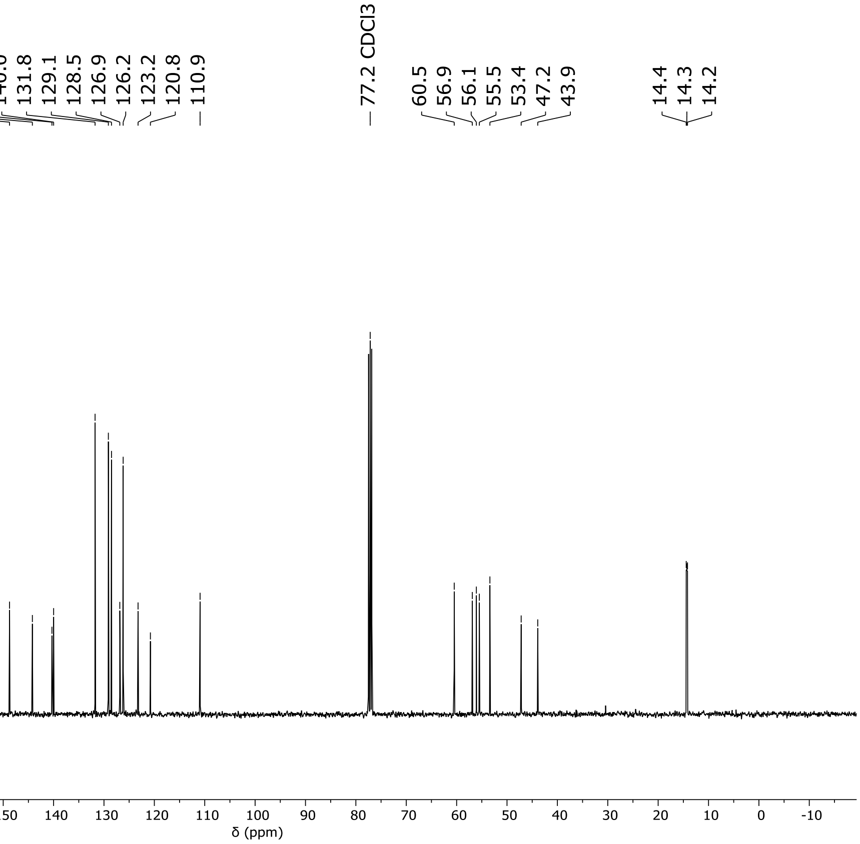

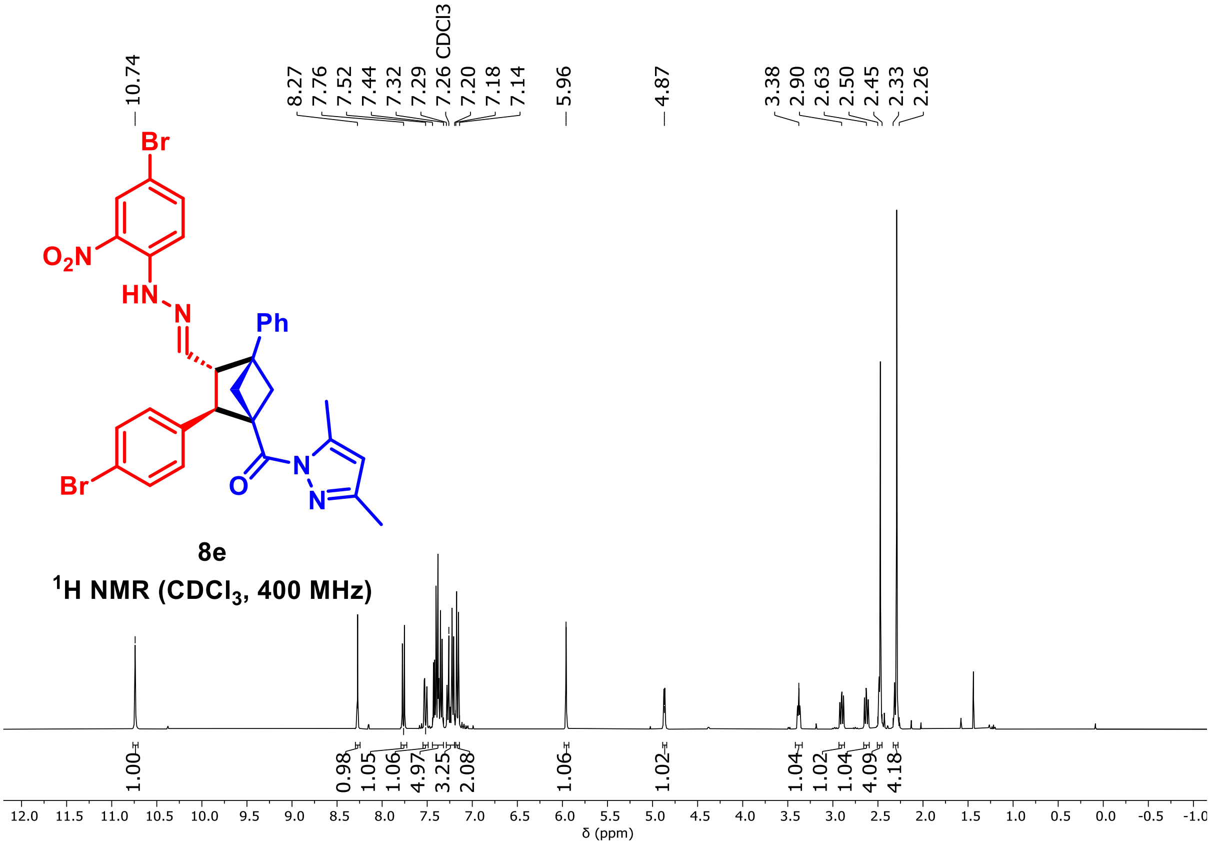

8e
1H NMR (CDCl3, 400 MHz)
10.74
8.27
7.76
7.52
7.44
7.32
7.29
7.26 CDCl3
7.20
7.18
7.14
5.96
4.87
3.38
2.90
2.63
2.50
2.45
2.33
2.26
1.00
0.98
1.05
1.06
4.97
3.25
2.08
1.06
1.02
1.04
1.02
1.04
4.09
4.18
δ (ppm)

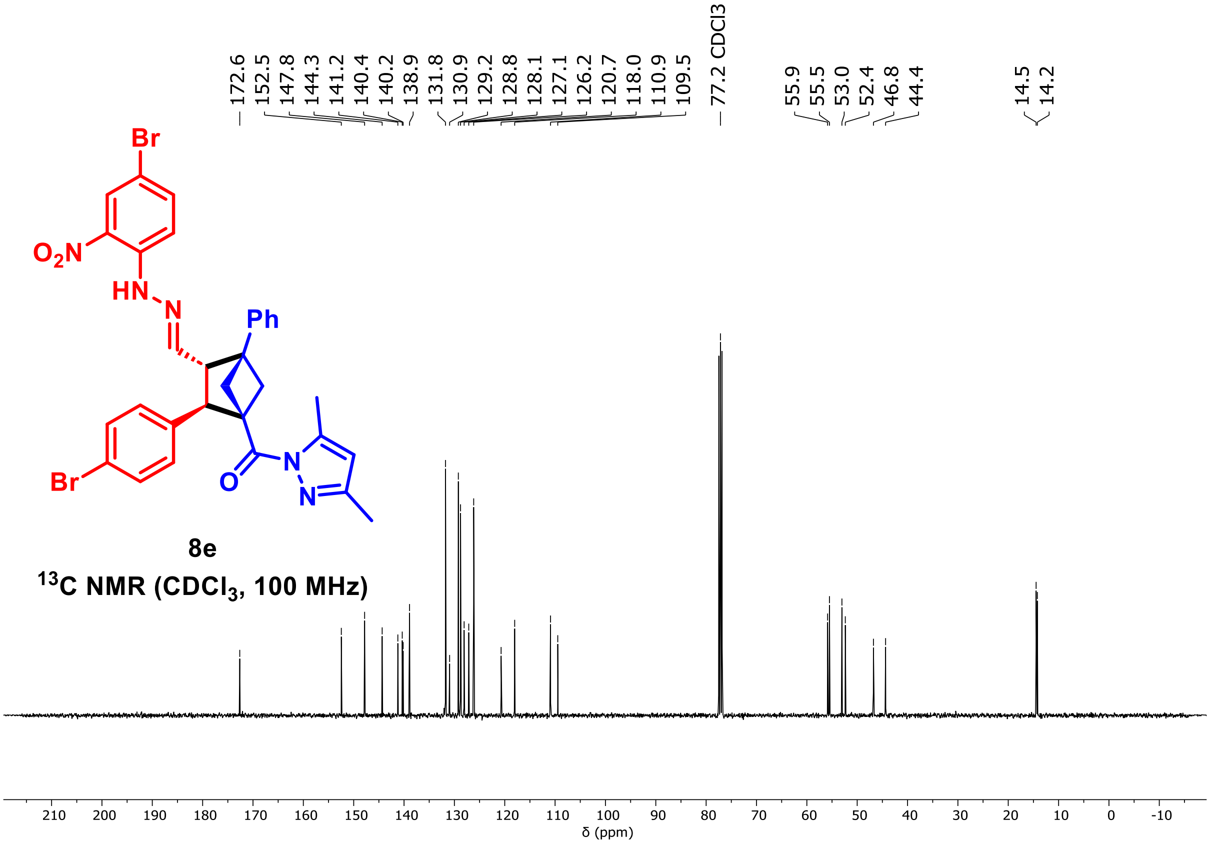

8e
13C NMR (CDCl3, 100 MHz)
172.6
152.5
147.8
144.3
141.2
140.4
140.2
138.9
131.8
130.9
129.2
128.8
128.1
127.1
126.2
120.7
118.0
110.9
109.5
77.2 CDCl3
55.9
55.5
53.0
52.4
46.8
44.4
14.5
14.2
δ (ppm)

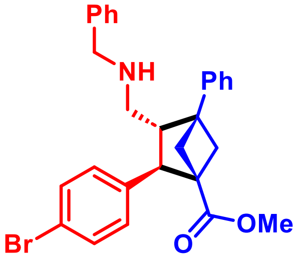

8f

^{1}H NMR ($CDCl_3$, 400 MHz)

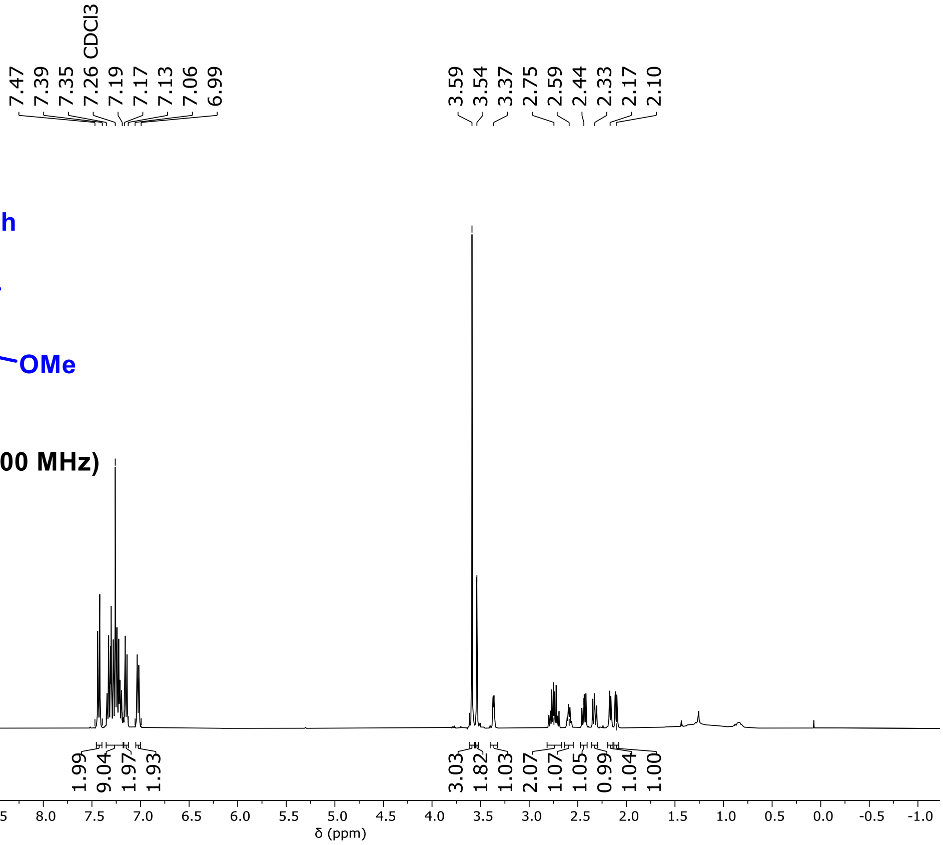

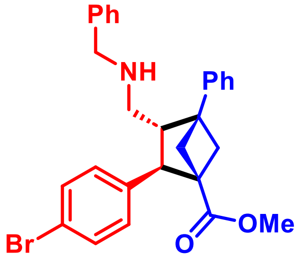

8f

^{13}C NMR ($CDCl_3$, 100 MHz)

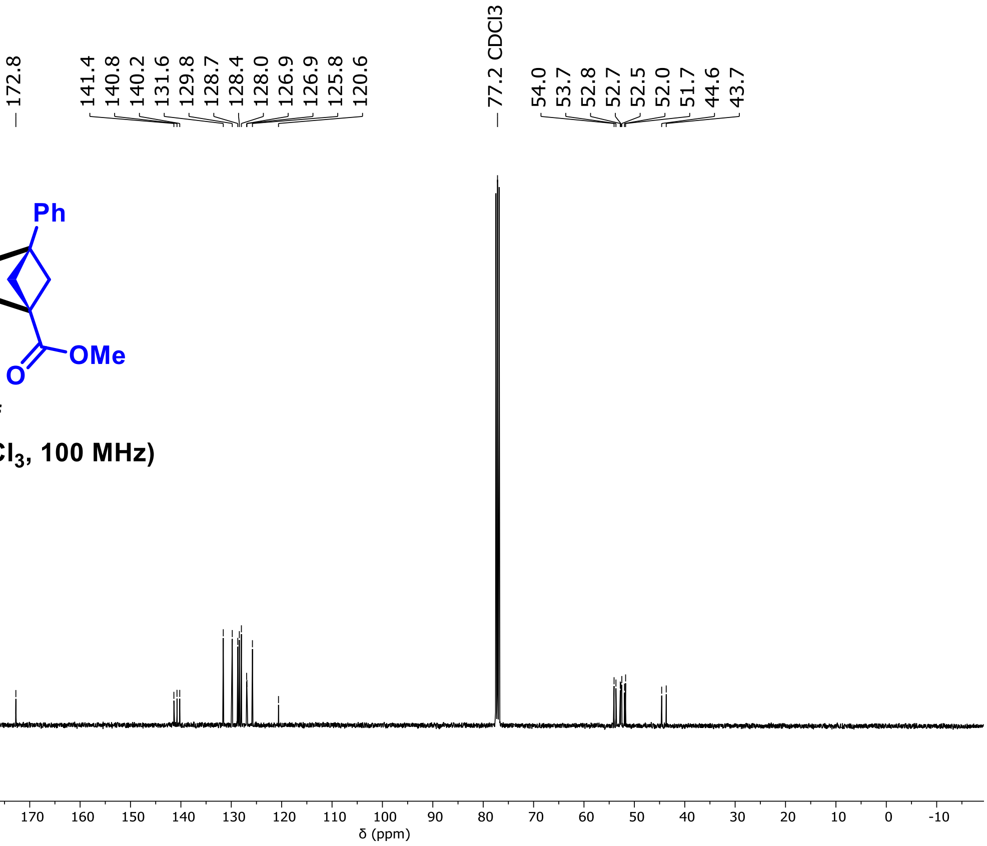

8. UPC2-Chromatograms

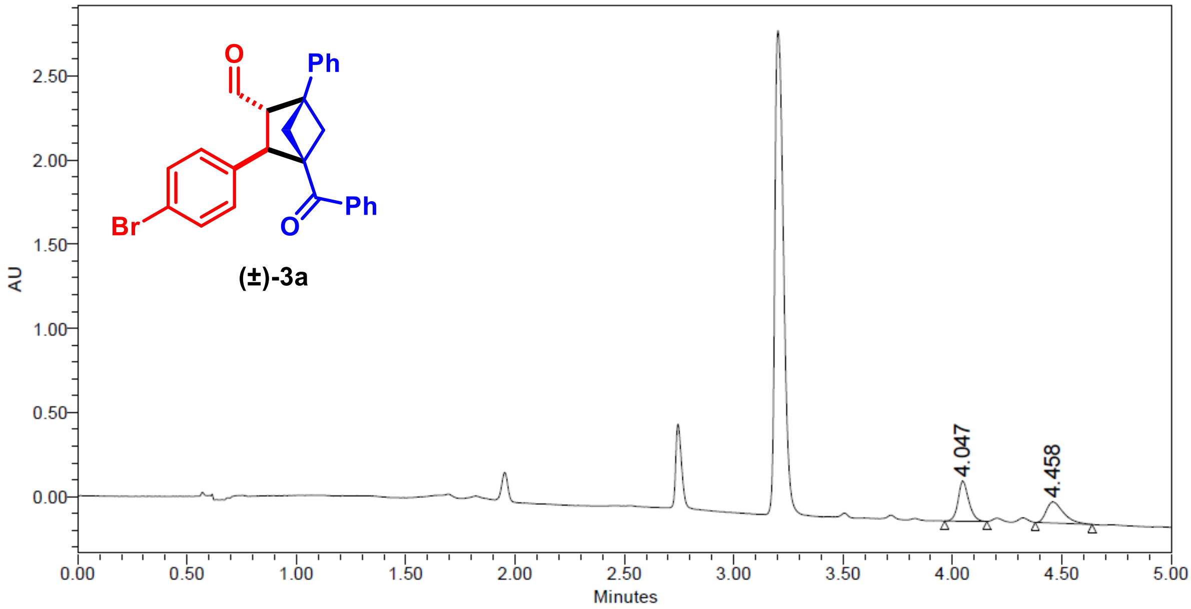

	Retention Time (min)	% Area
1	4.047	53.98
2	4.458	46.02

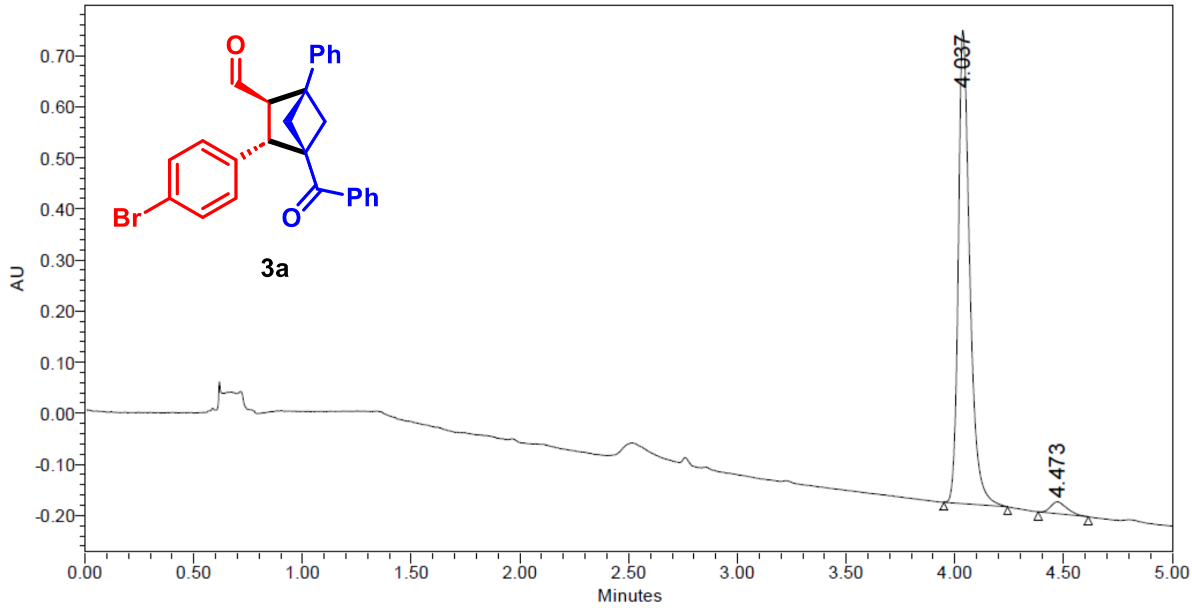

	Retention Time (min)	% Area
1	4.037	96.53
2	4.473	3.47

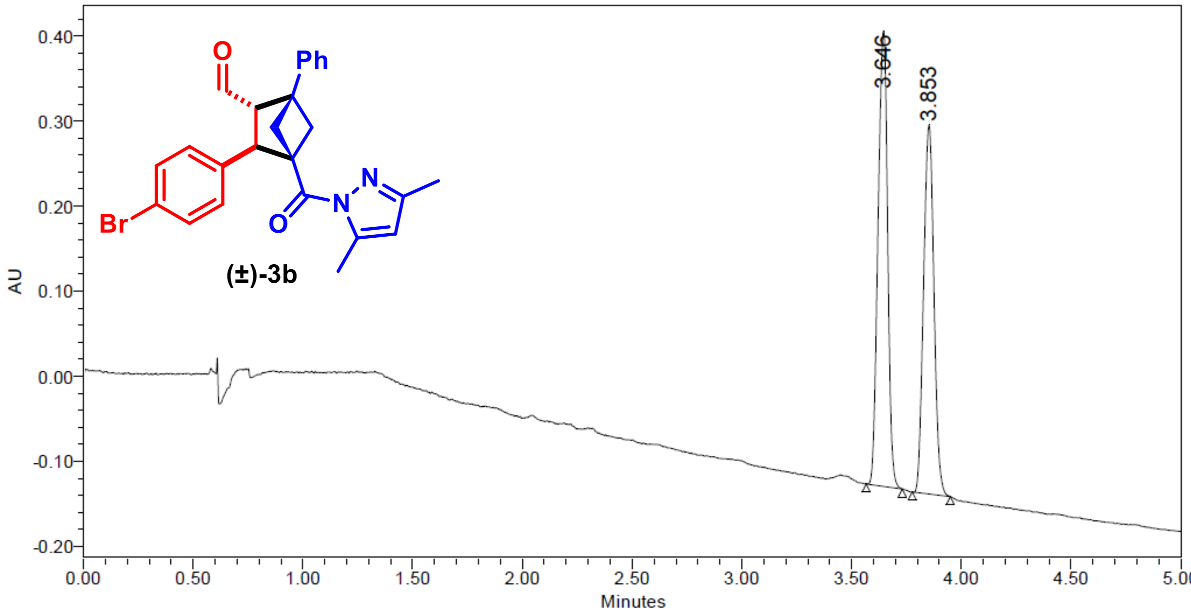

	Retention Time (min)	% Area
1	3.646	53.35
2	3.853	46.65

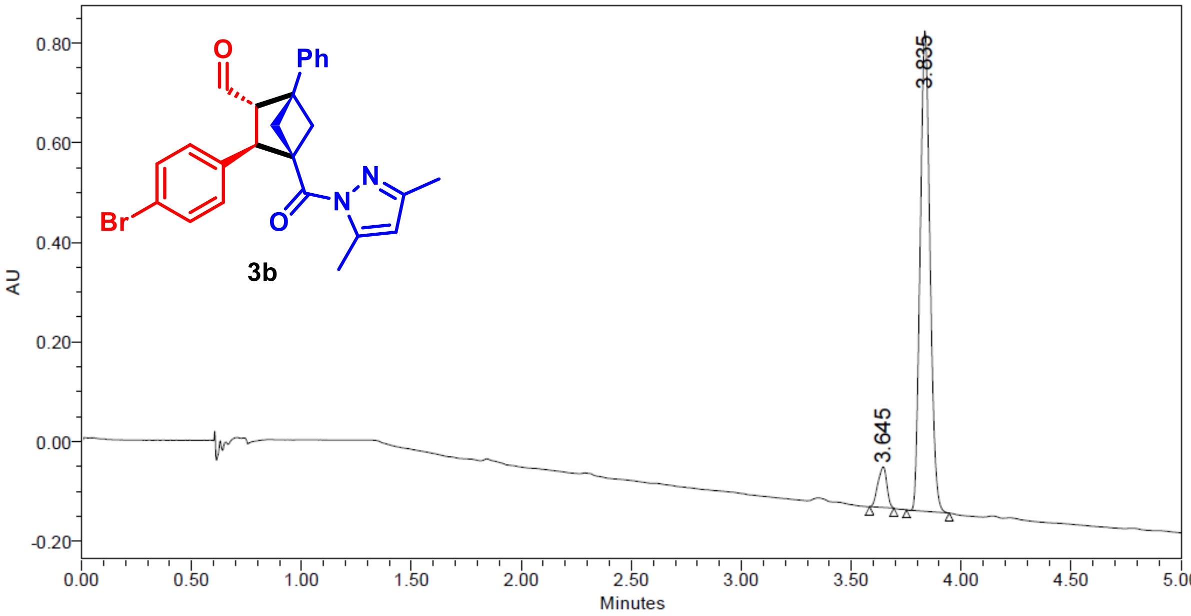

	Retention Time (min)	% Area
1	3.645	7.11
2	3.835	92.89

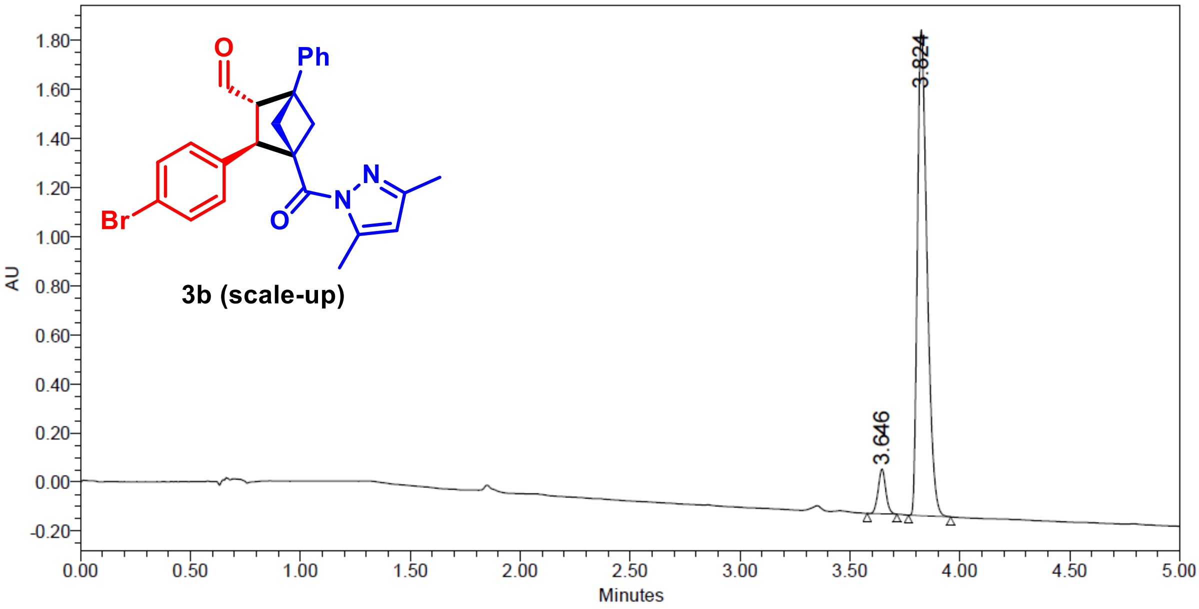

	Retention Time (min)	% Area
1	3.646	6.76
2	3.824	93.24

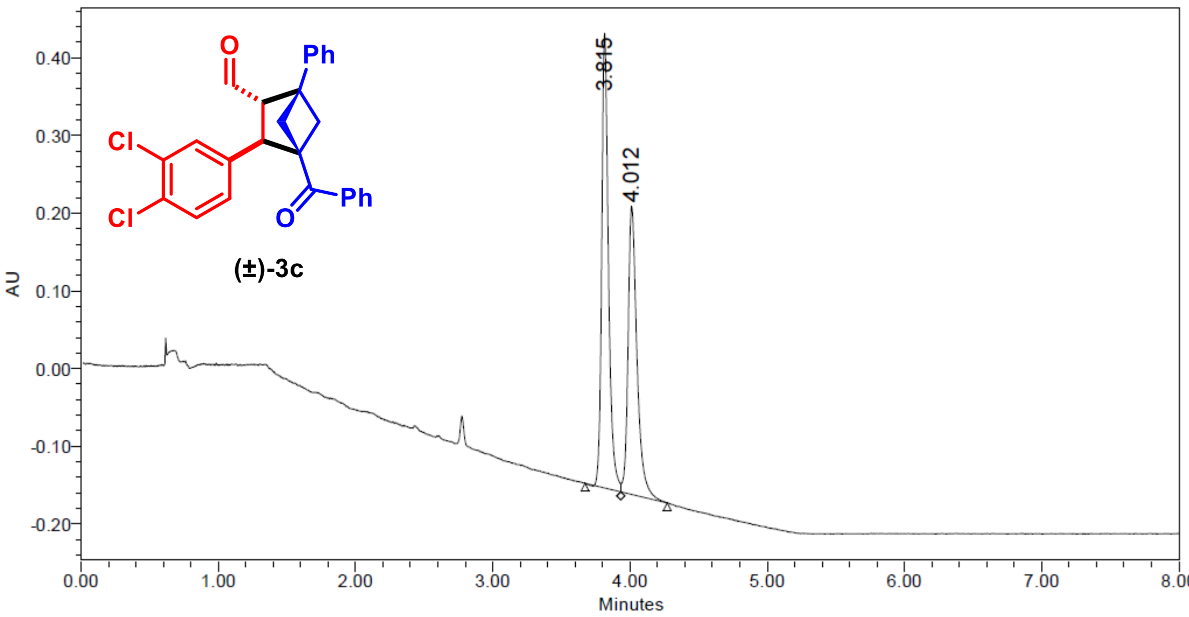

	Retention Time (min)	% Area
1	3.815	54.48
2	4.012	45.52

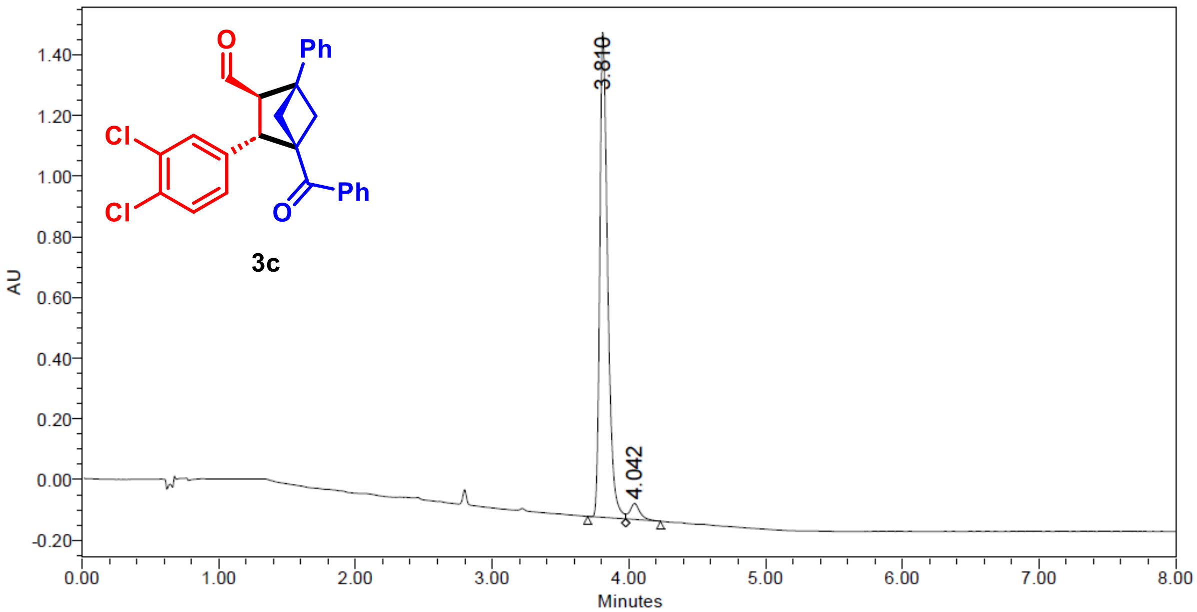

	Retention Time (min)	% Area
1	3.810	95.96
2	4.042	4.04

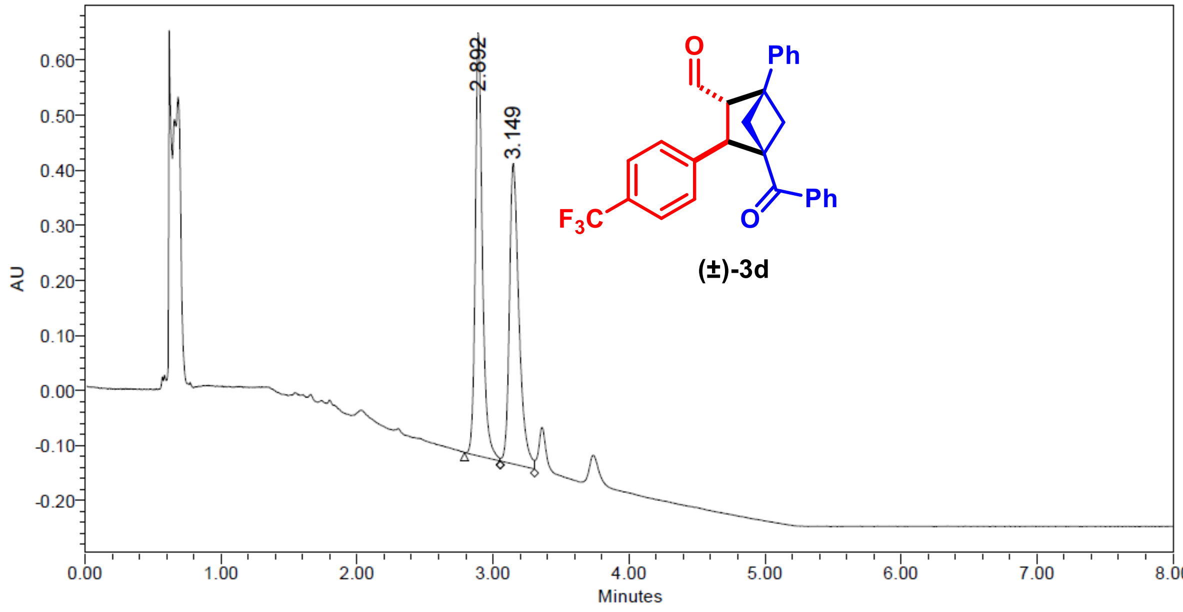

	Retention Time (min)	% Area
1	2.892	50.95
2	3.149	49.05

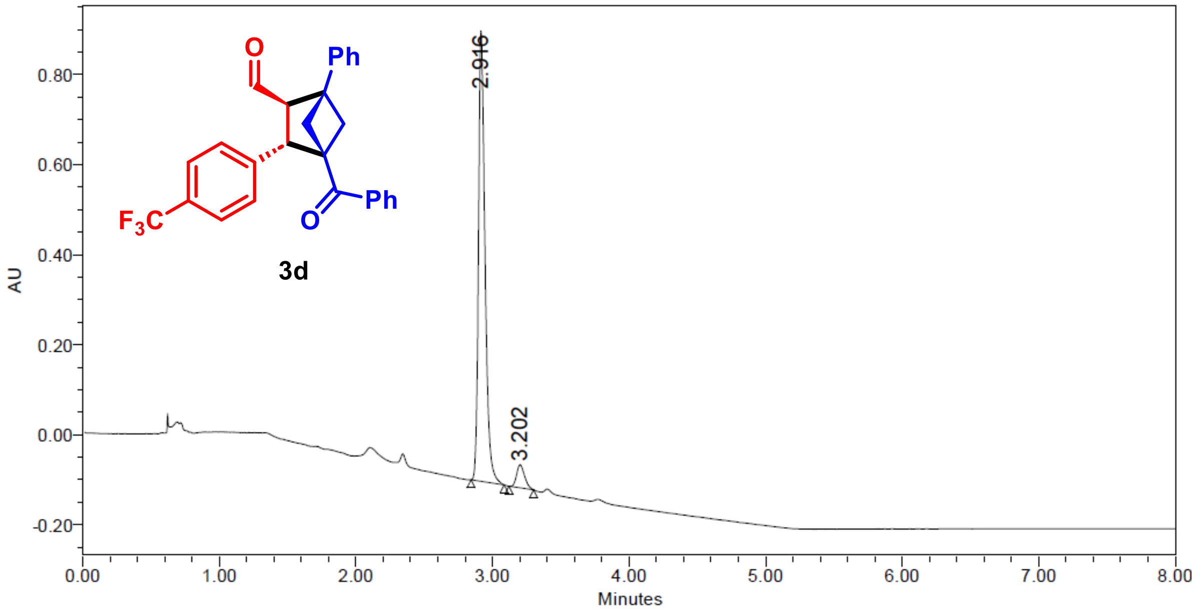

	Retention Time (min)	% Area
1	2.916	94.08
2	3.202	5.92

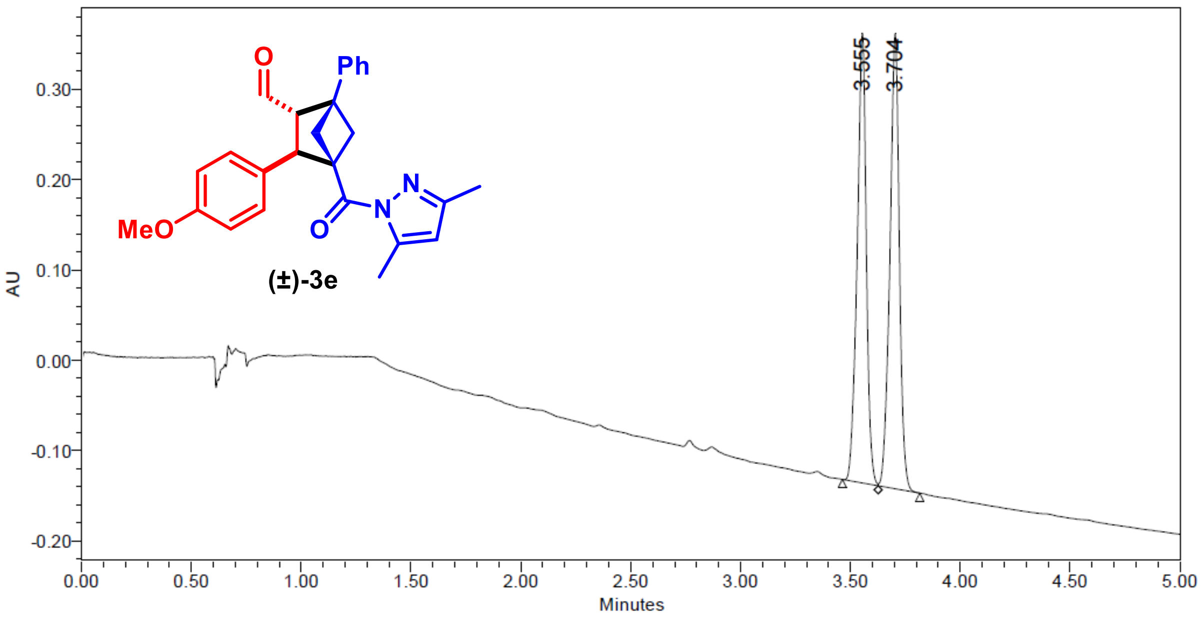

	Retention Time (min)	% Area
1	3.555	48.82
2	3.704	51.18

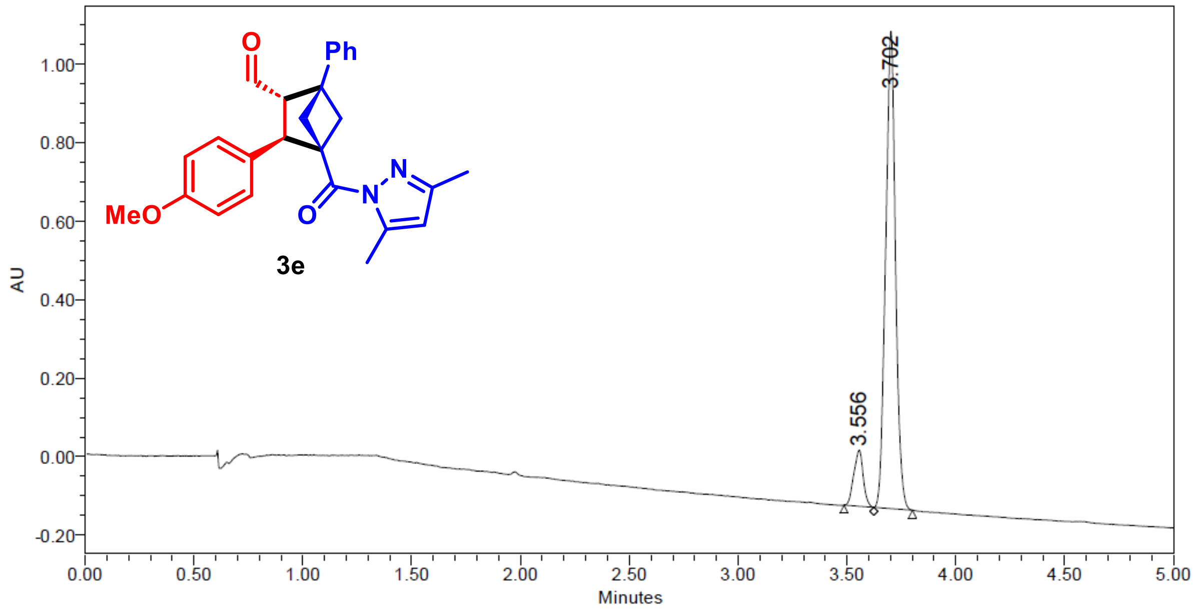

	Retention Time (min)	% Area
1	3.556	10.06
2	3.702	89.94

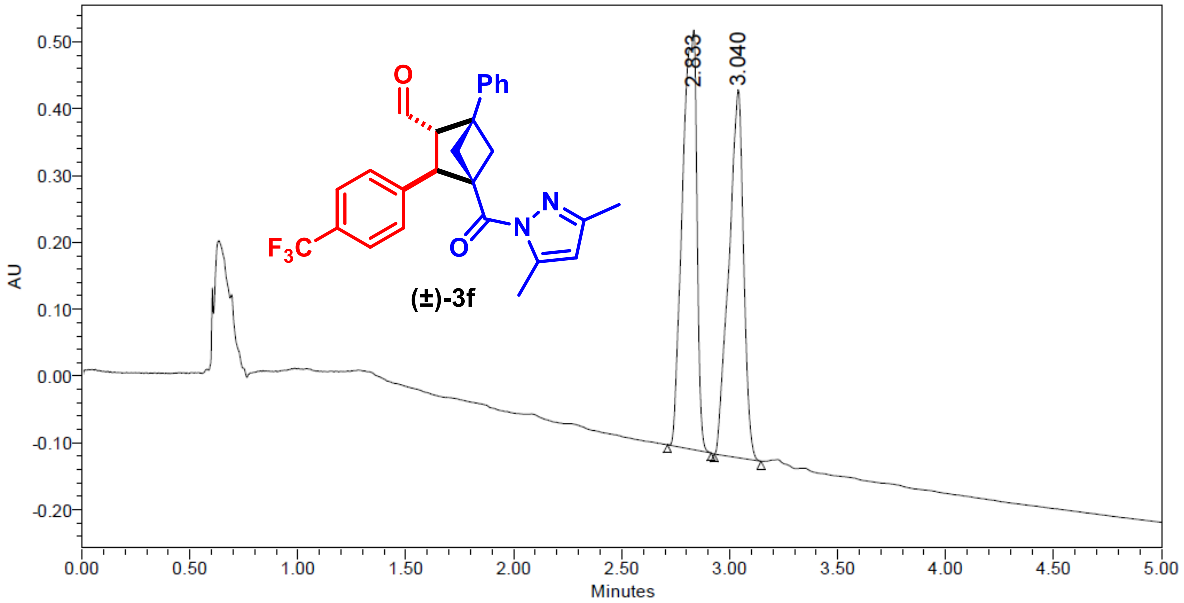

	Retention Time (min)	% Area
1	2.833	52.79
2	3.040	47.21

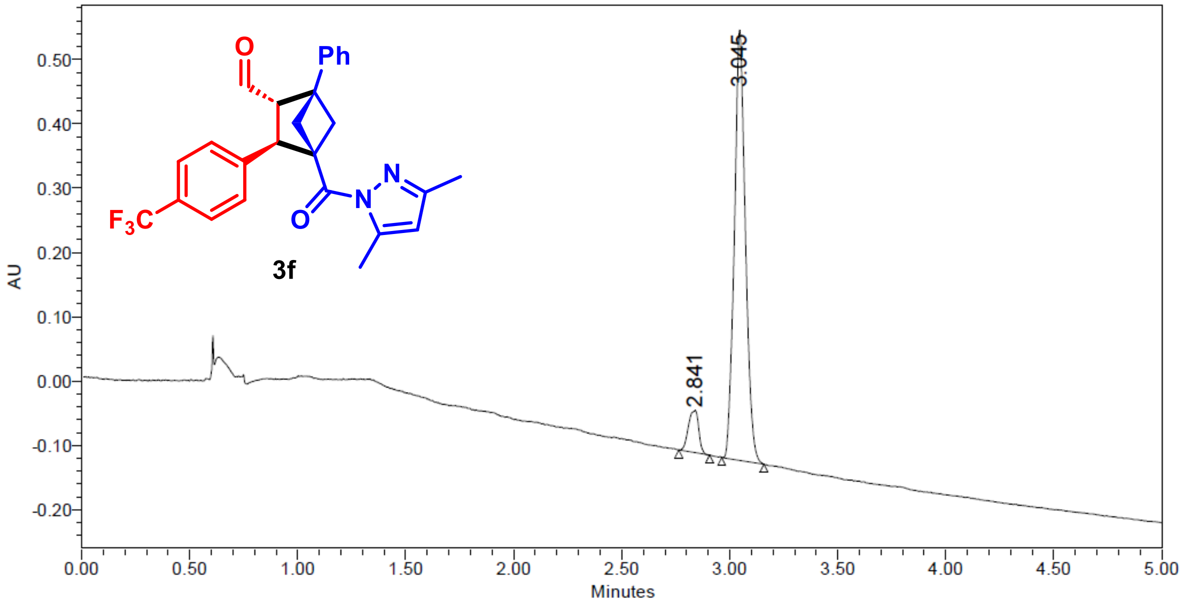

	Retention Time (min)	% Area
1	2.841	8.29
2	3.045	91.71

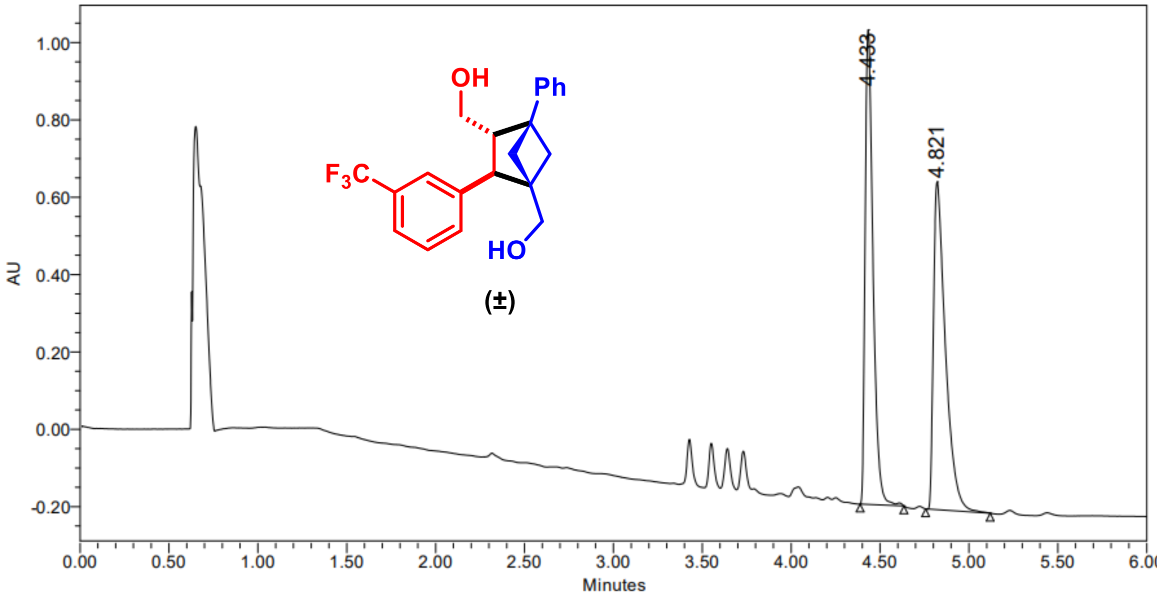

	Retention Time (min)	% Area
1	4.433	49.04
2	4.821	50.96

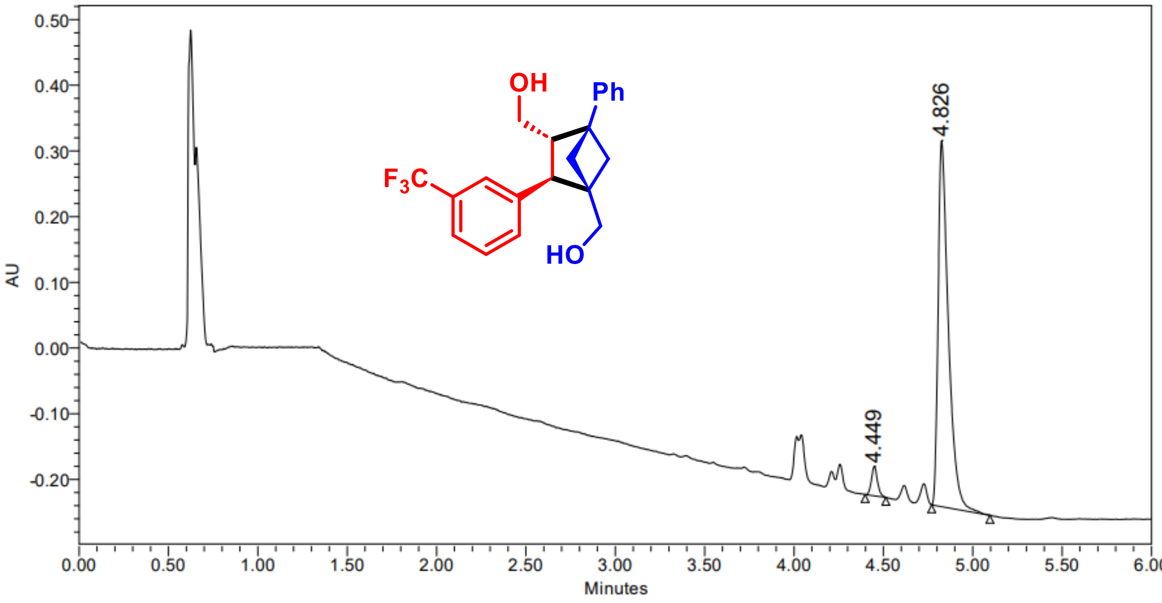

	Retention Time (min)	% Area
1	4.449	4.52
2	4.826	95.48

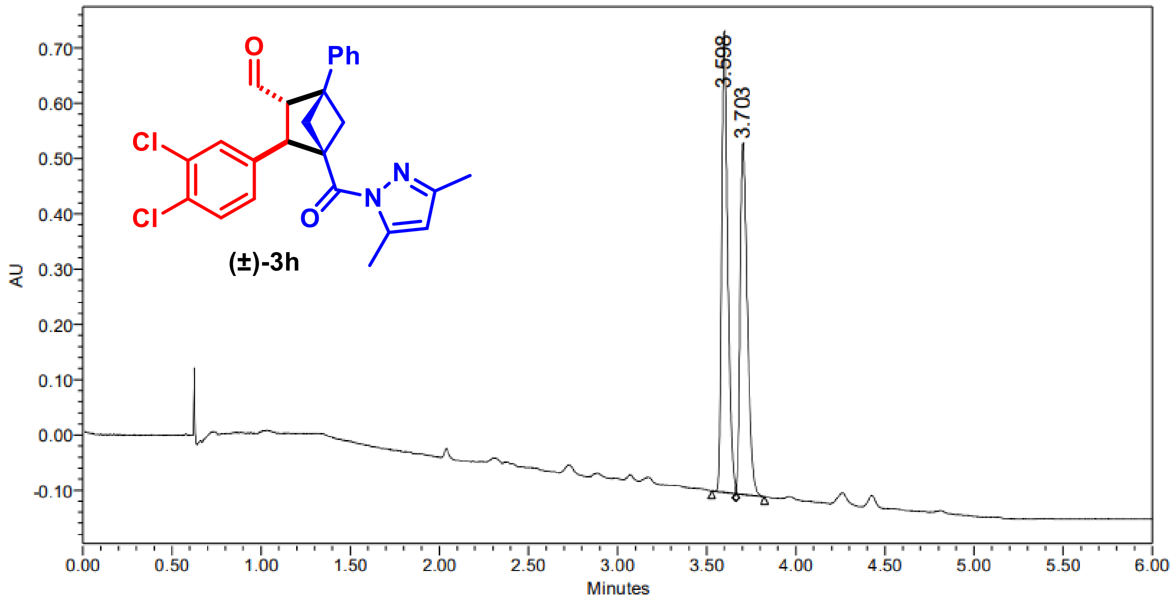

	Retention Time (min)	% Area
1	3.598	53.24
2	3.703	46.76

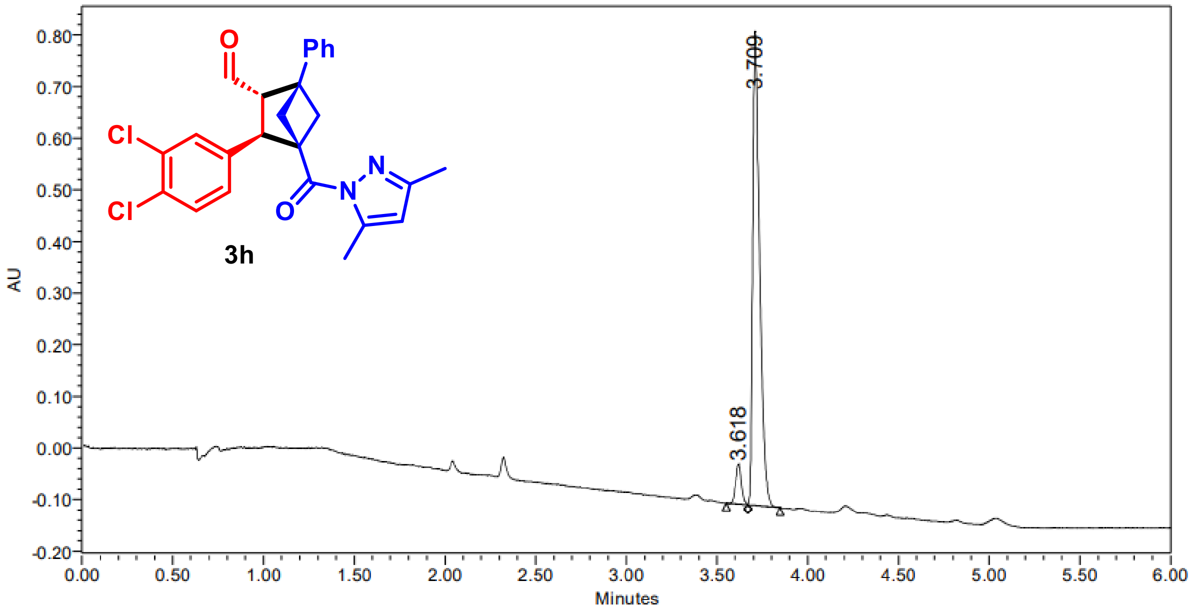

	Retention Time (min)	% Area
1	3.618	6.35
2	3.709	93.65

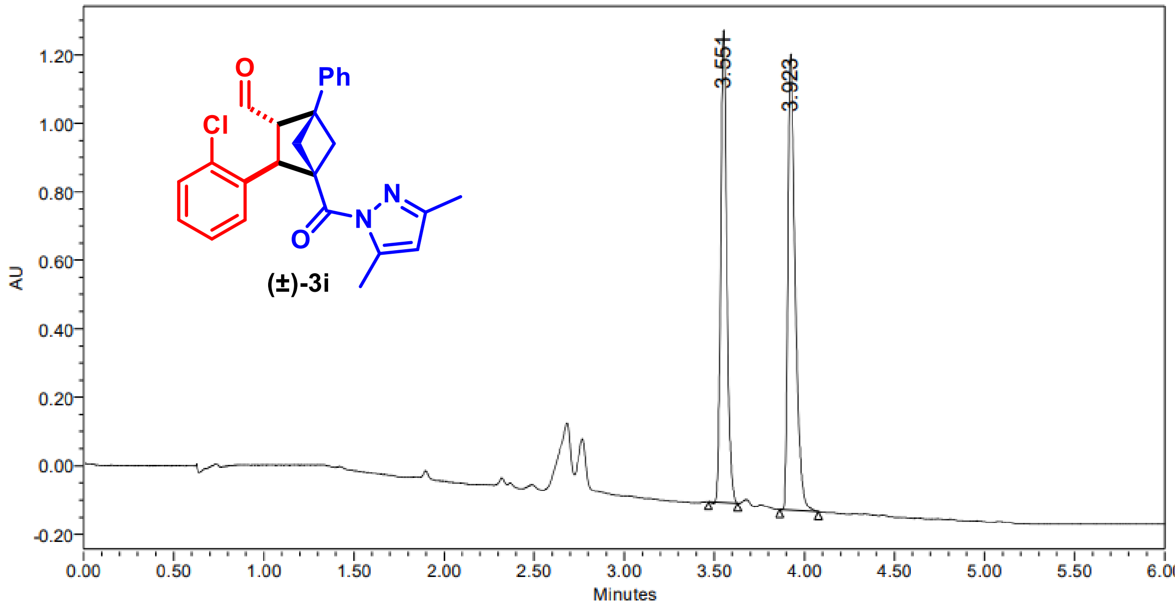

	Retention Time (min)	% Area
1	3.551	45.69
2	3.923	54.31

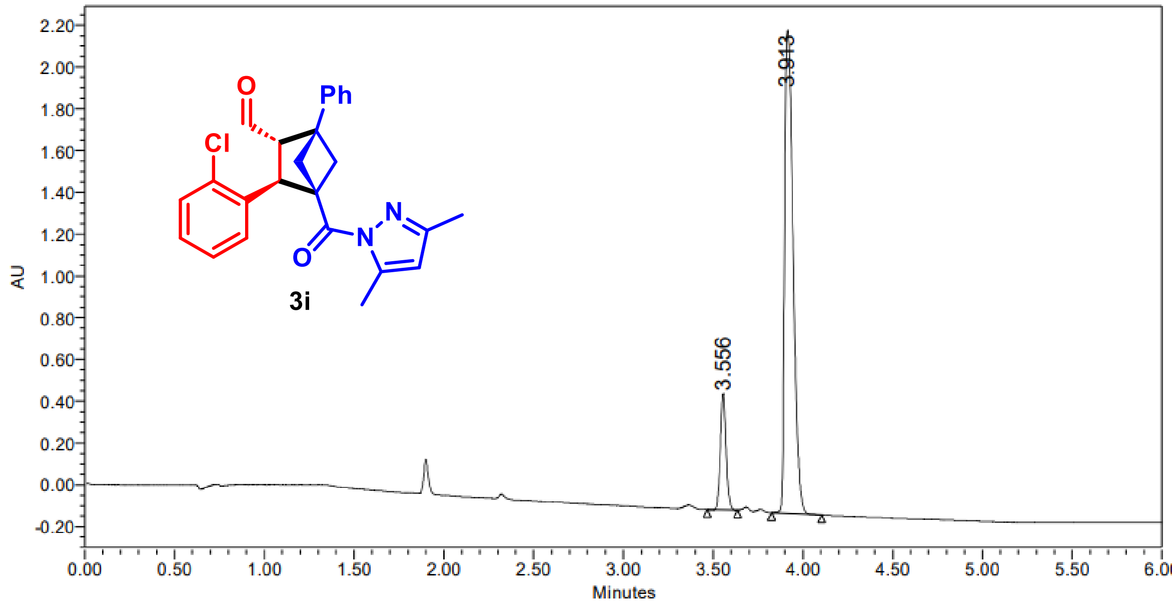

	Retention Time (min)	% Area
1	3.556	13.95
2	3.913	86.05

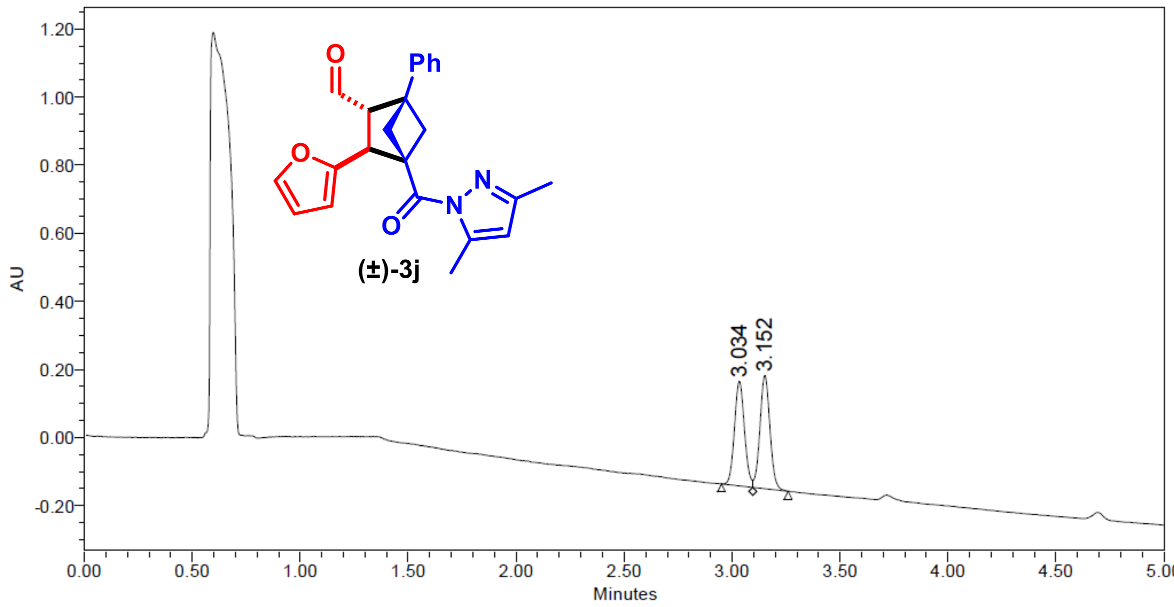

	Retention Time (min)	% Area
1	3.152	51.93
2	3.034	48.07

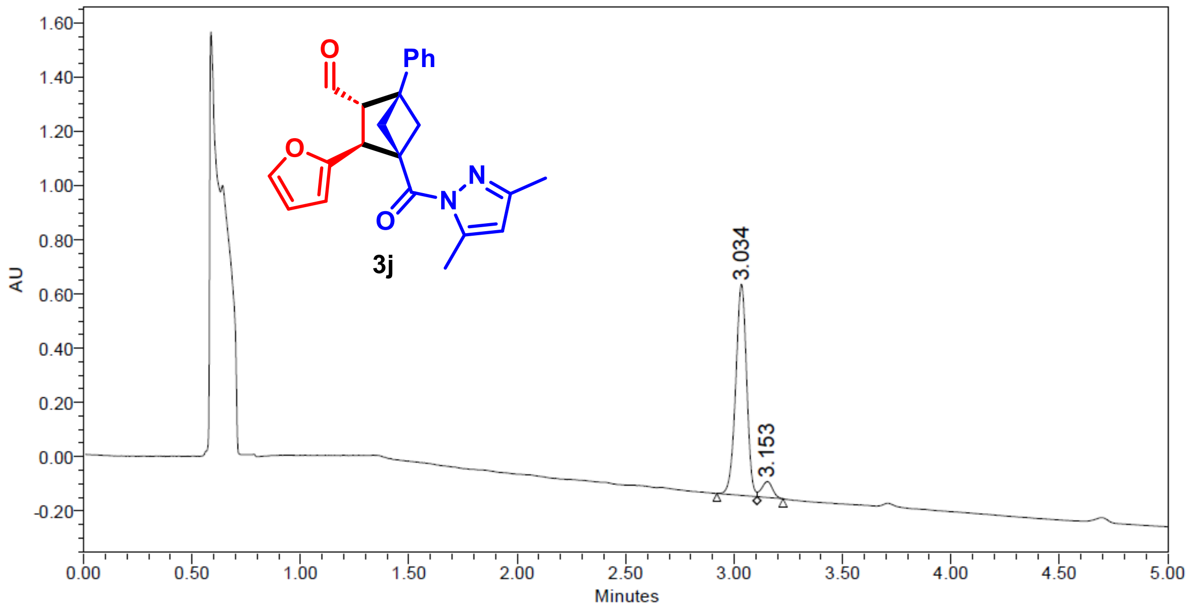

	Retention Time (min)	% Area
1	3.034	93.01
2	3.153	6.99

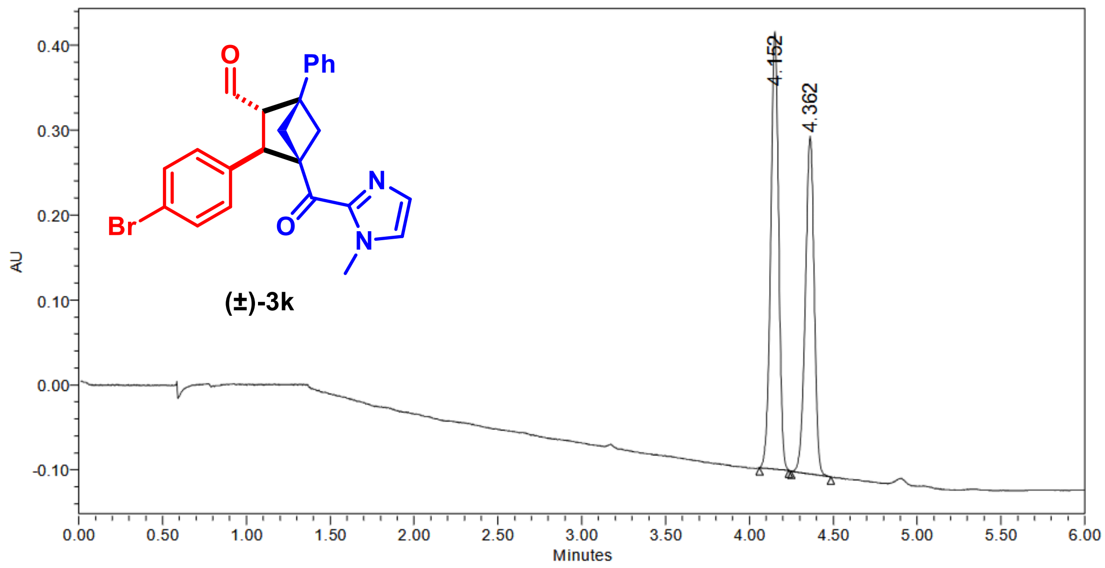

	Retention Time (min)	% Area
1	4.152	53.90
2	4.362	46.10

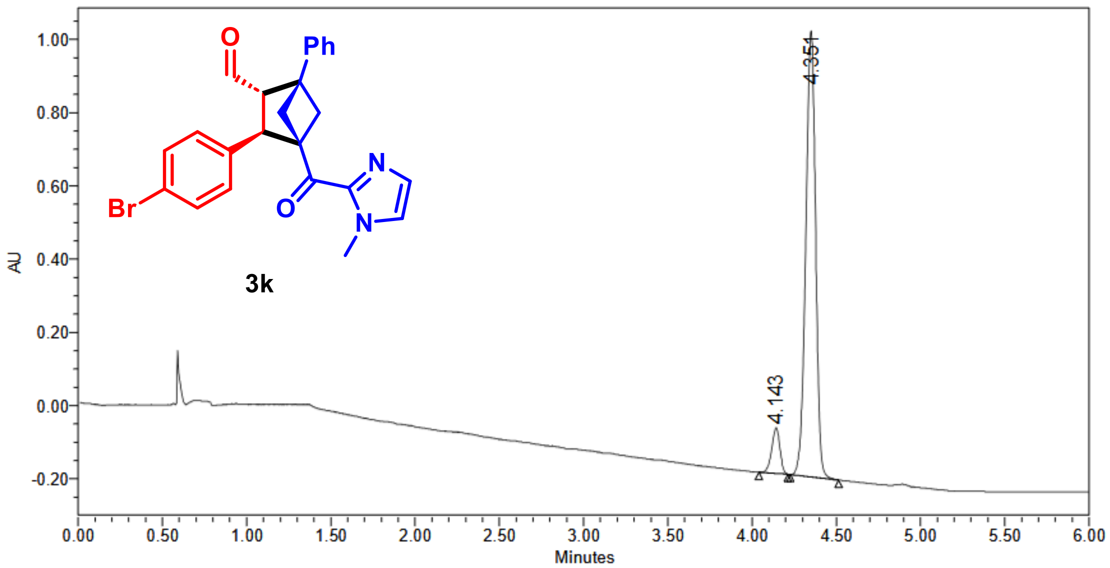

	Retention Time (min)	% Area
1	4.143	8.02
2	4.351	91.98

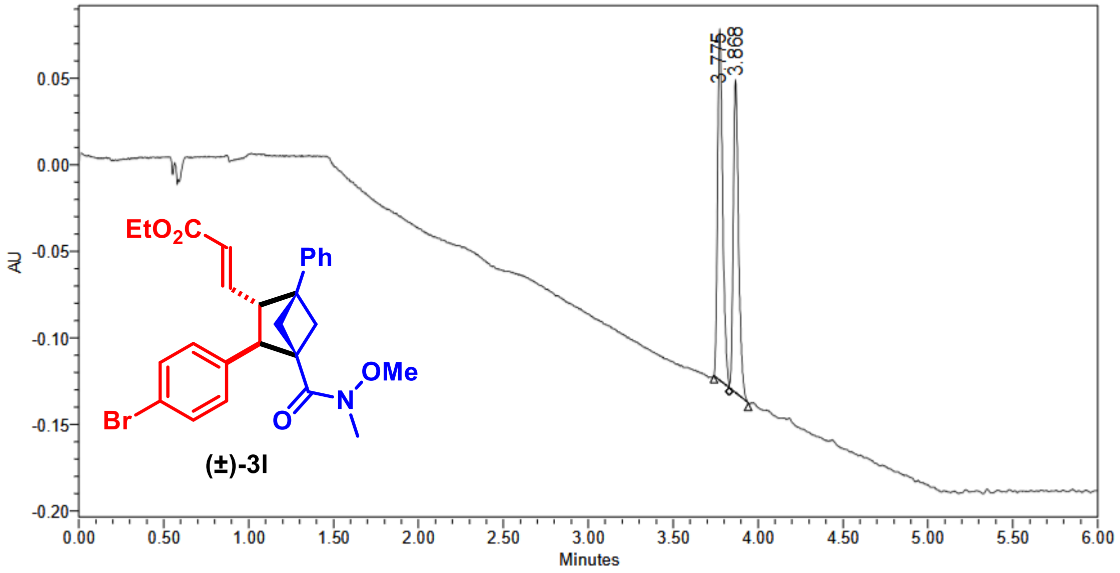

	Retention Time (min)	% Area
1	3.775	51.98
2	3.868	48.02

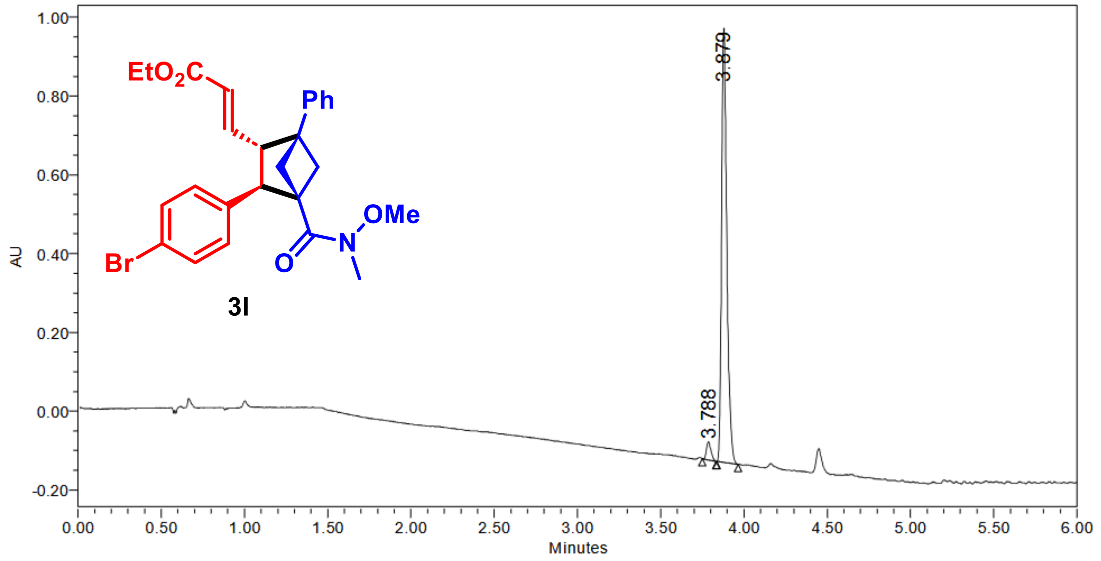

	Retention Time (min)	% Area
1	3.788	3.48
2	3.879	96.52

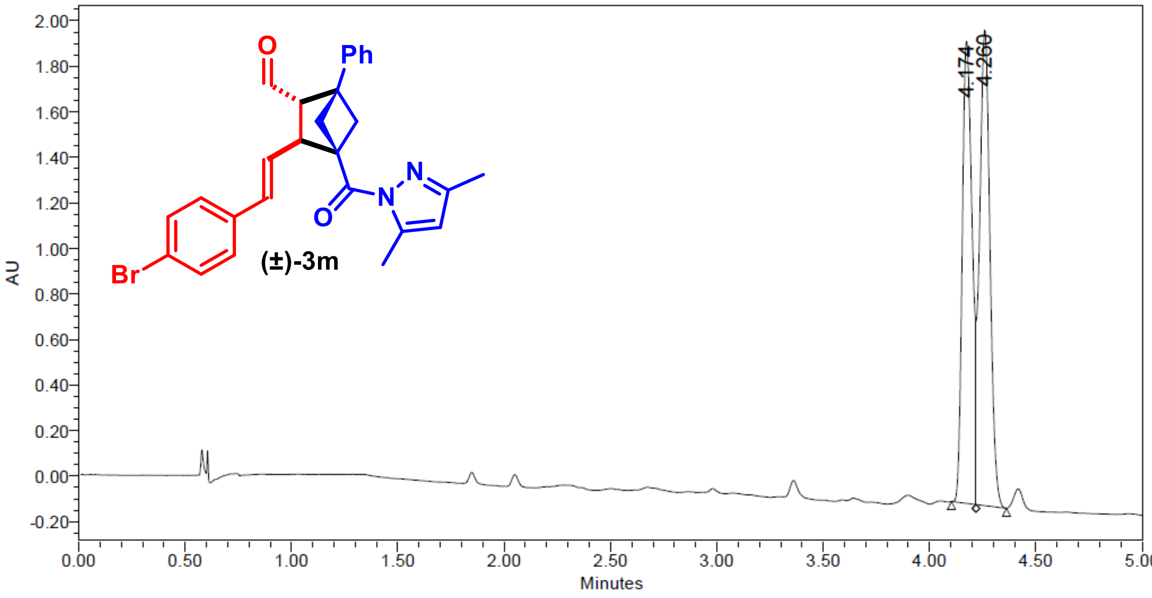

	Retention Time (min)	% Area
1	4.174	47.74
2	4.260	52.26

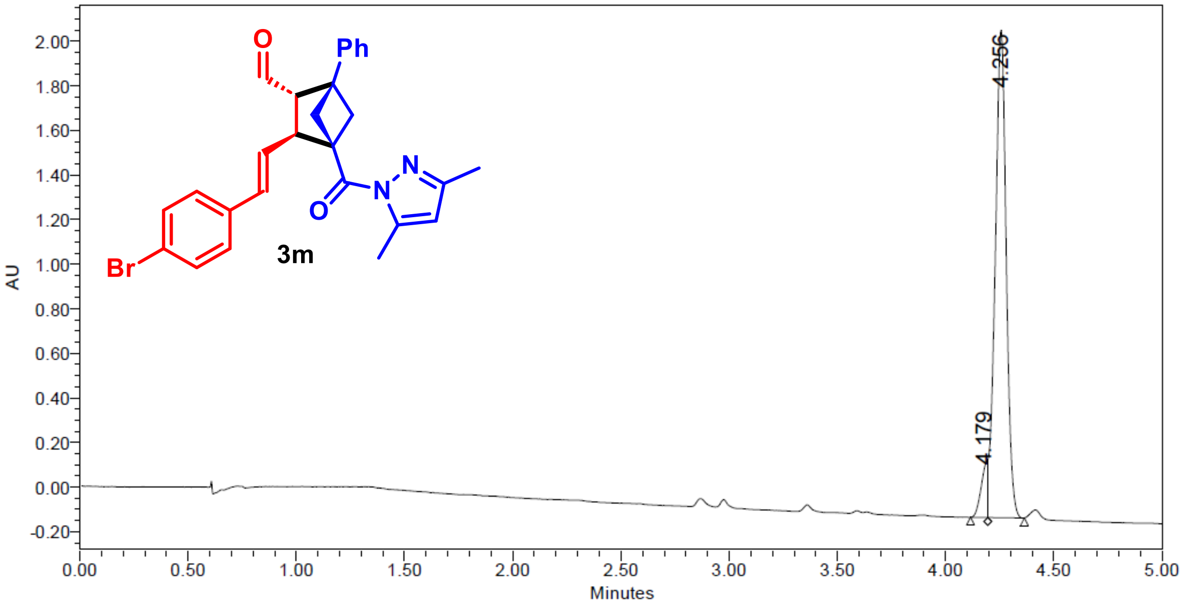

	Retention Time (min)	% Area
1	4.179	6.17
2	4.256	93.83

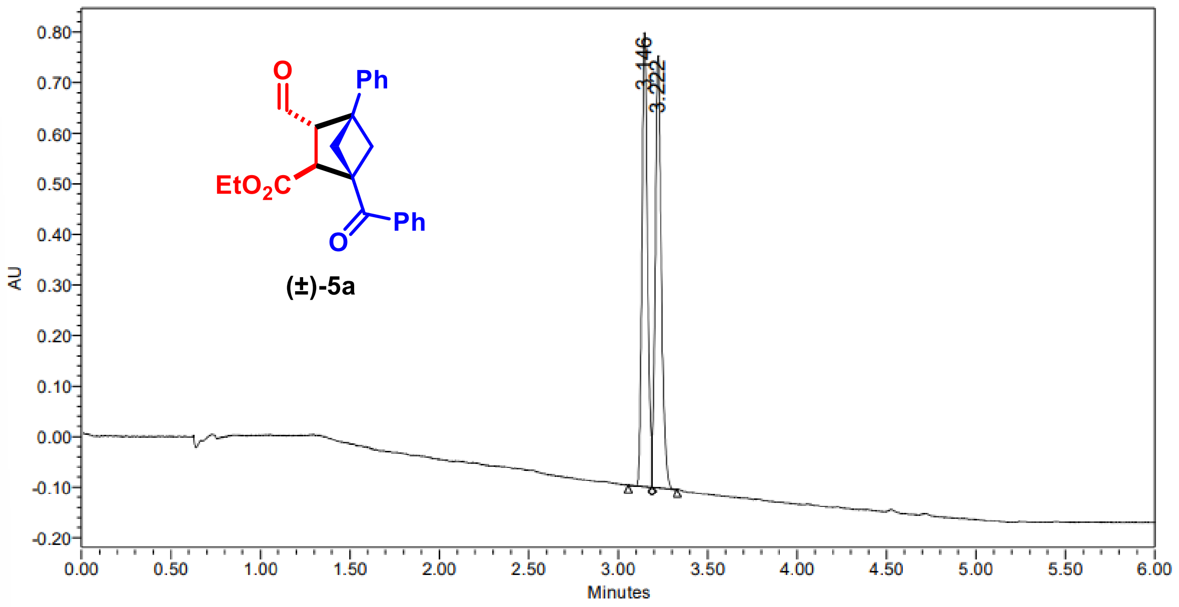

	Retention Time (min)	% Area
1	3.146	49.11
2	3.222	50.89

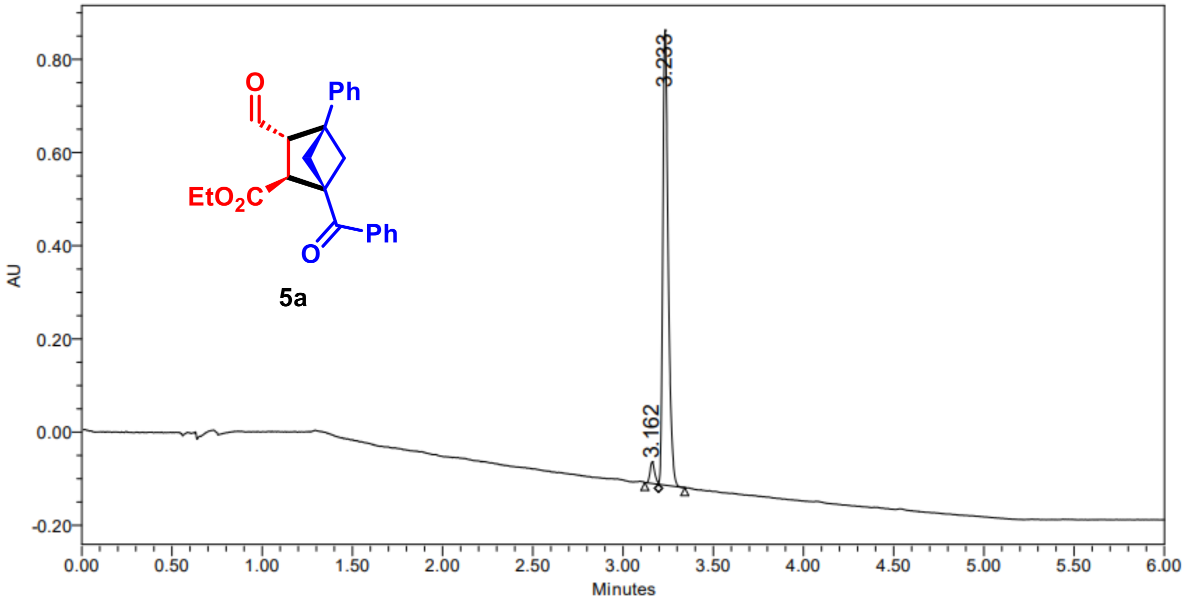

	Retention Time (min)	% Area
1	3.162	3.96
2	3.233	96.04

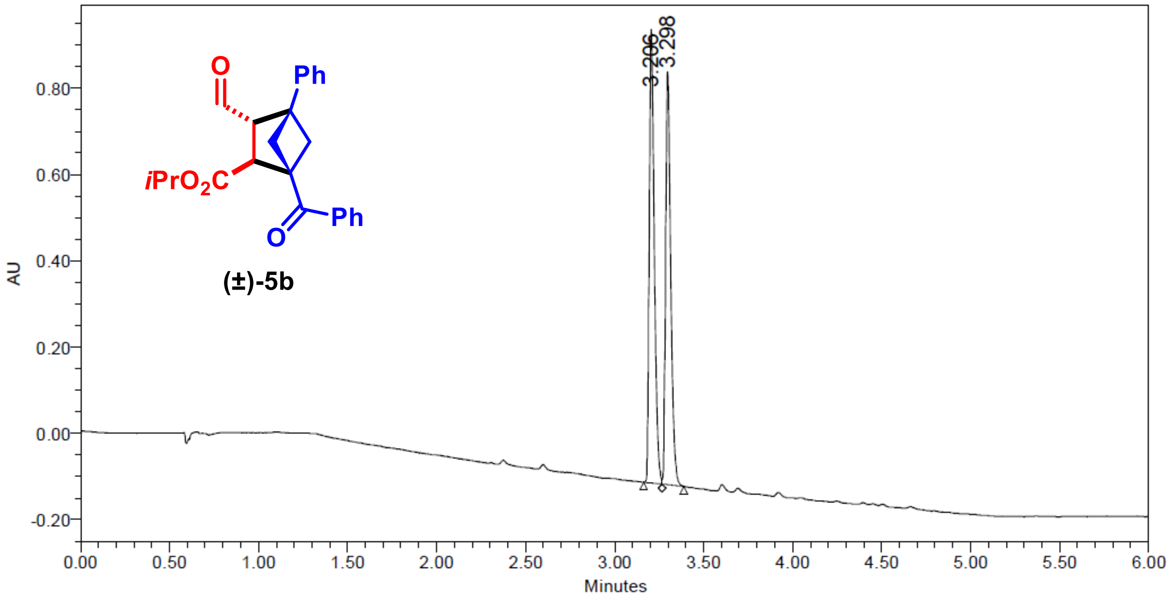

	Retention Time (min)	% Area
1	3.206	52.01
2	3.298	47.99

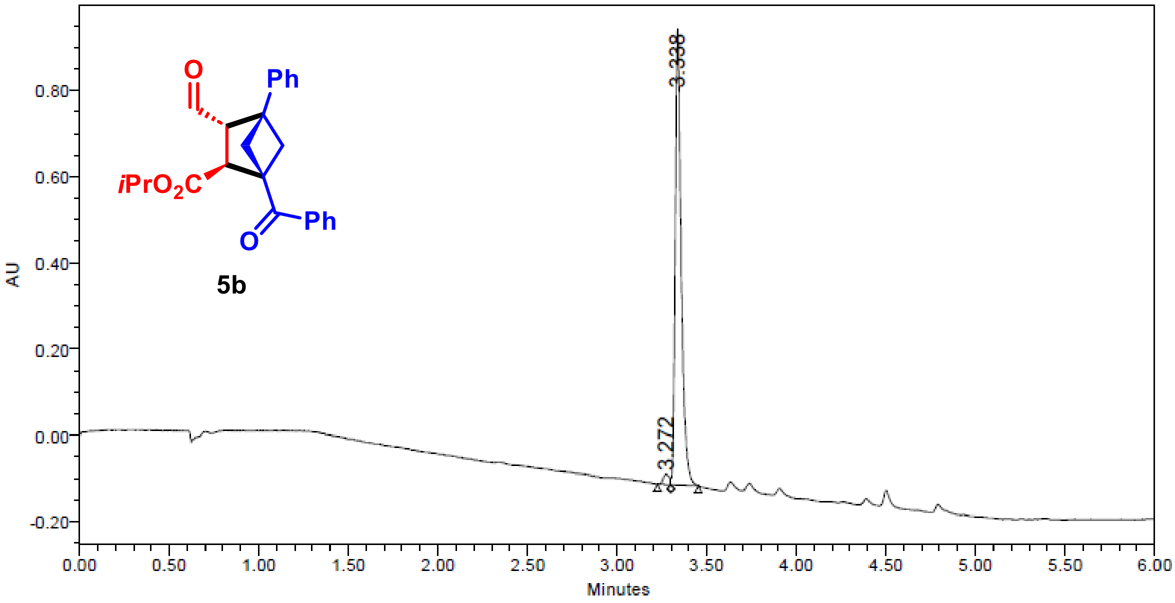

	Retention Time (min)	% Area
1	3.272	2.16
2	3.338	97.84

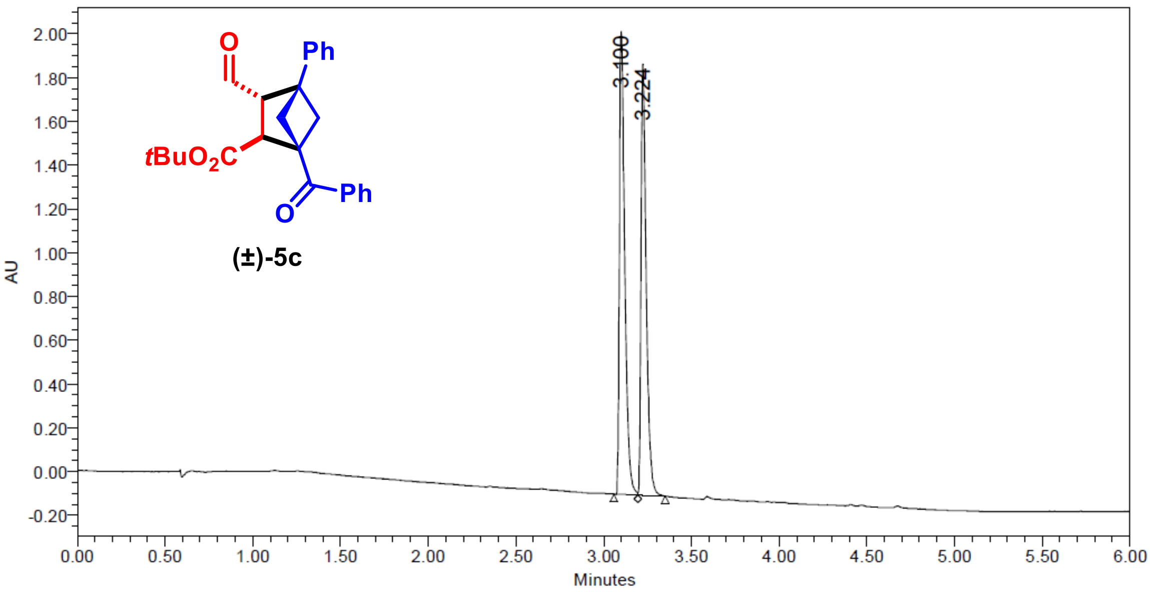

	Retention Time (min)	% Area
1	3.100	52.17
2	3.224	47.83

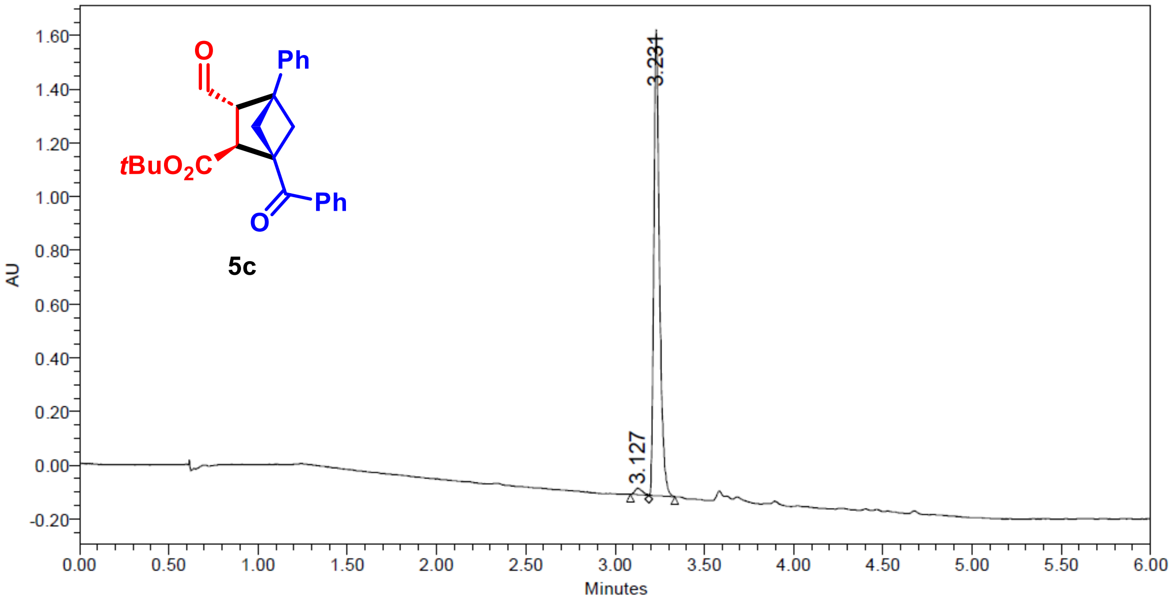

	Retention Time (min)	% Area
1	3.127	1.71
2	3.231	98.29

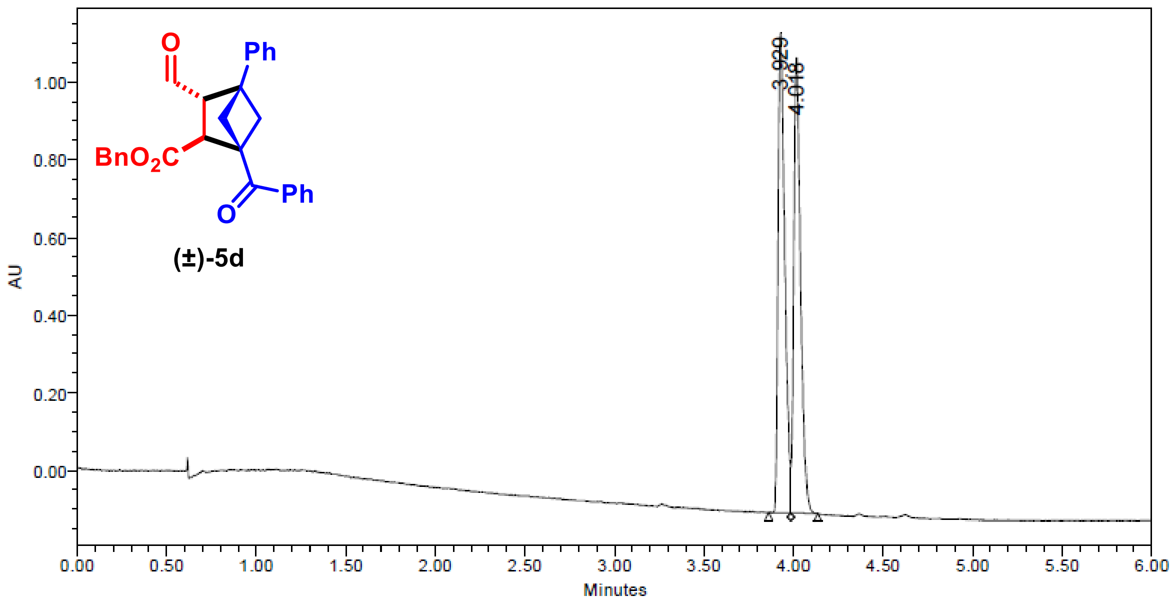

	Retention Time (min)	% Area
1	3.929	51.34
2	4.018	48.66

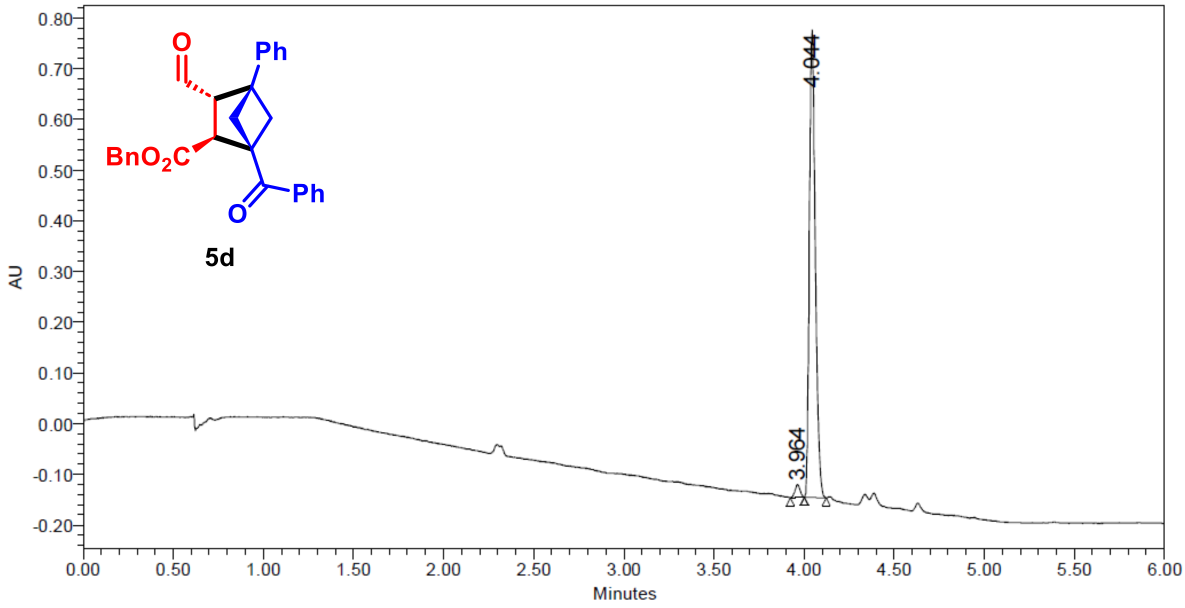

	Retention Time (min)	% Area
1	3.964	2.37
2	4.044	97.63

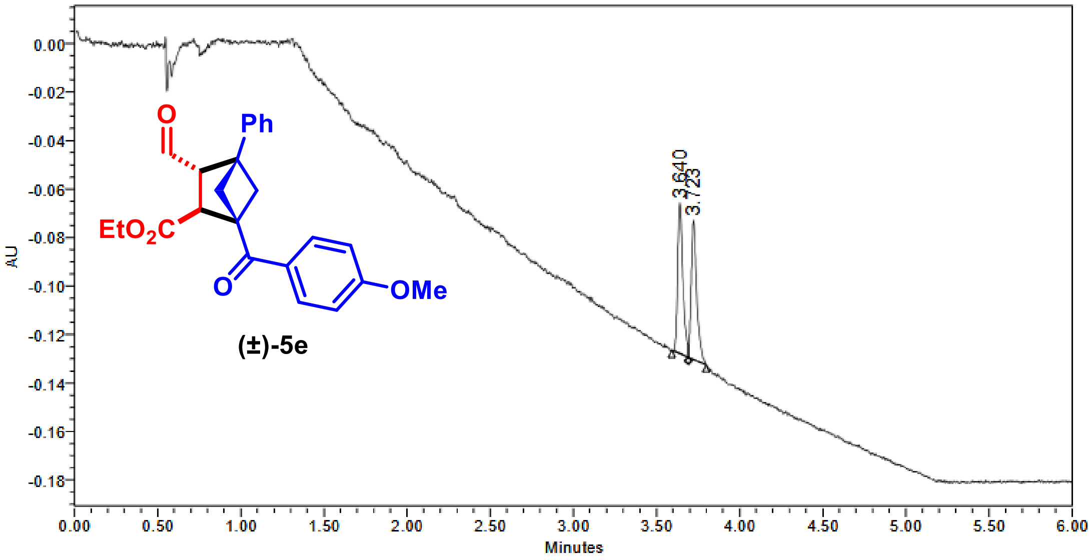

	Retention Time (min)	% Area
1	3.640	50.47
2	3.723	49.53

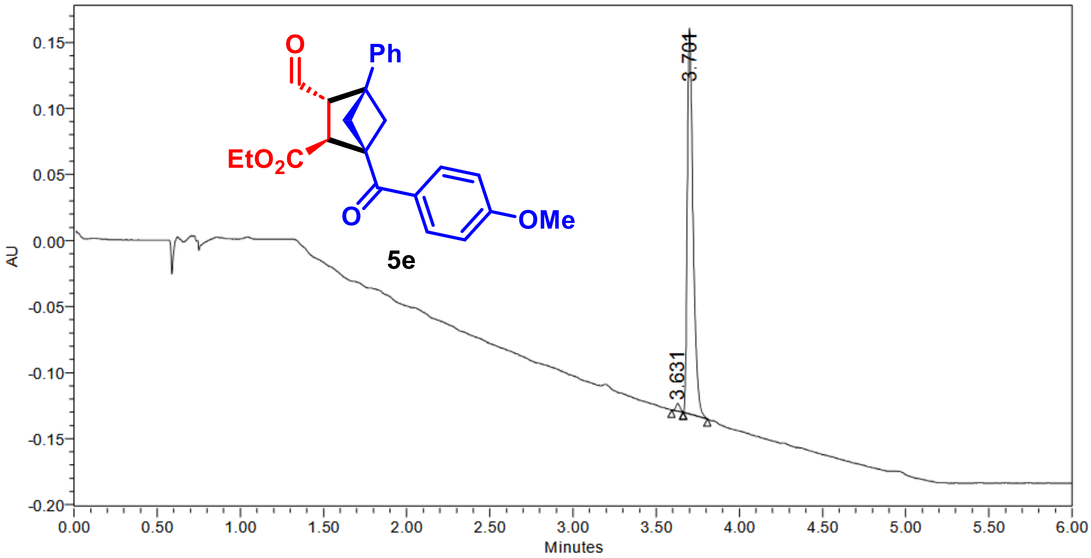

	Retention Time (min)	% Area
1	3.631	1.39
2	3.701	98.61

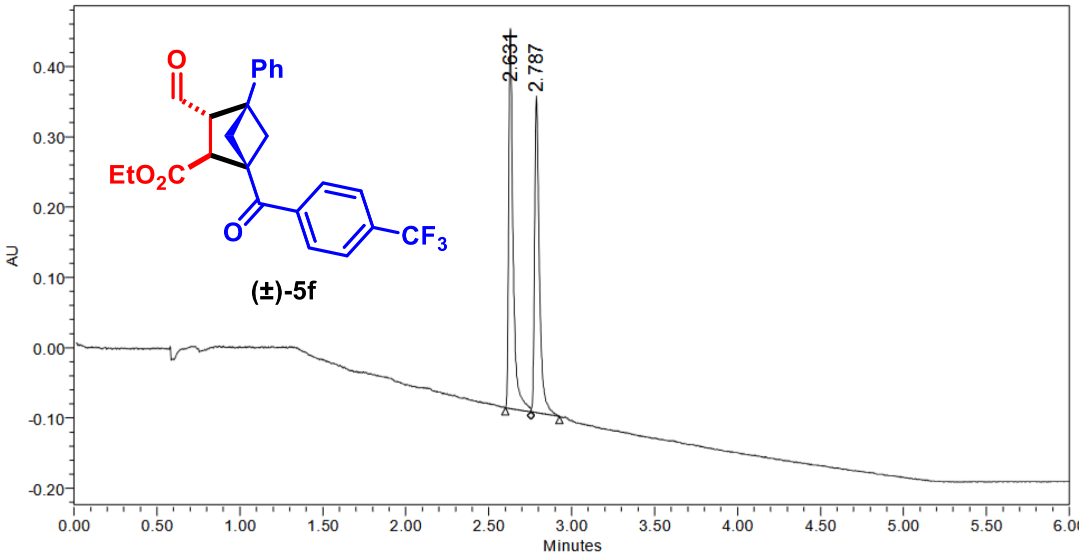

	Retention Time (min)	% Area
1	2.631	51.92
2	2.787	48.08

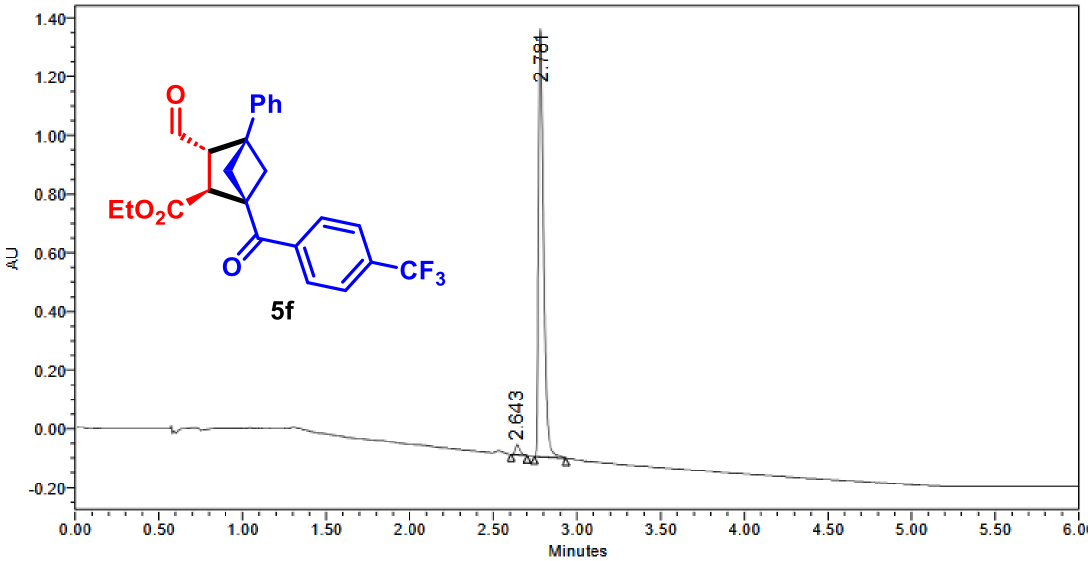

	Retention Time (min)	% Area
1	2.781	97.98
2	2.643	2.02

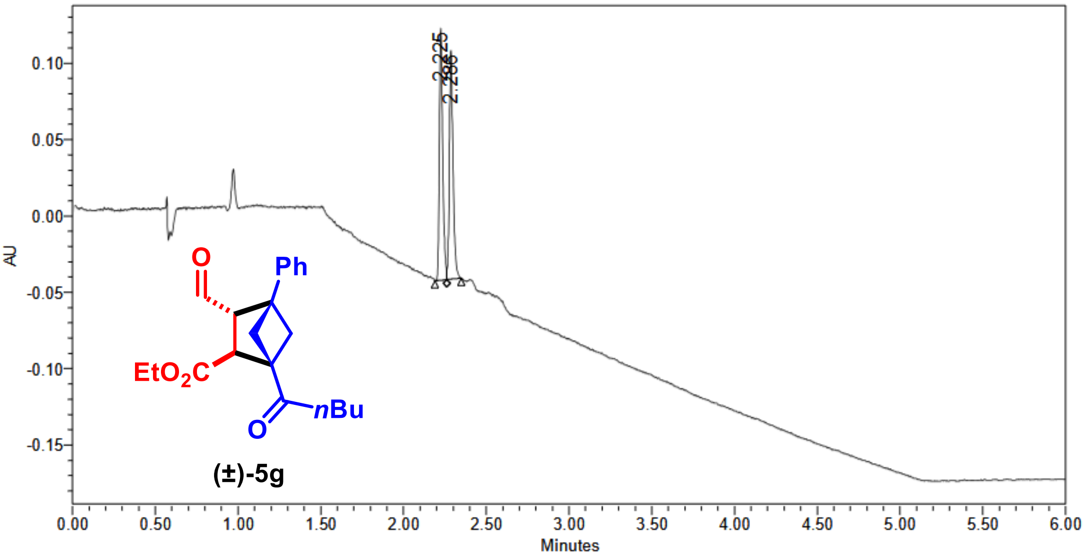

	Retention Time (min)	% Area
1	2.225	50.61
2	2.286	49.39

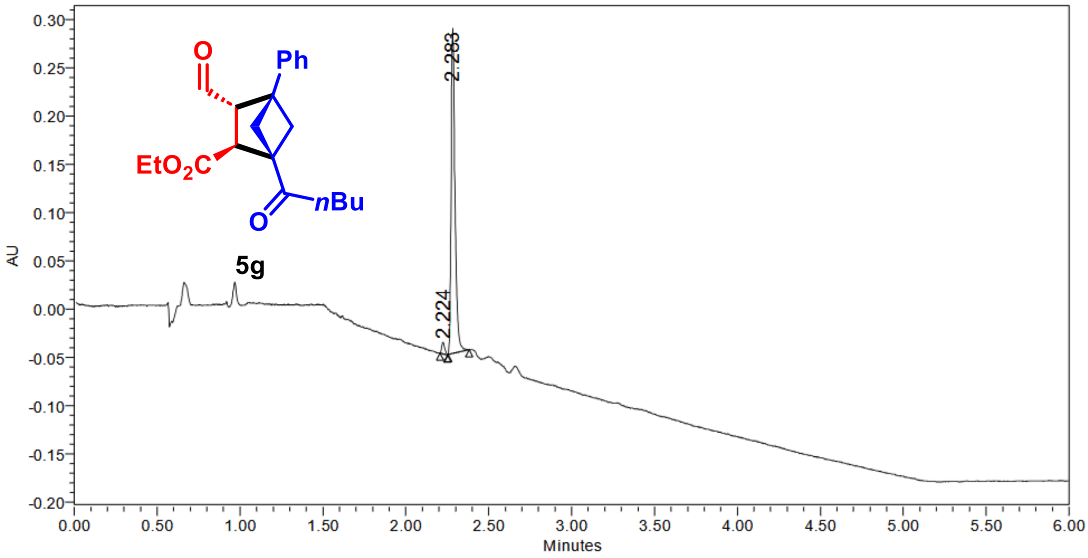

	Retention Time (min)	% Area
1	2.224	2.73
2	2.283	97.27

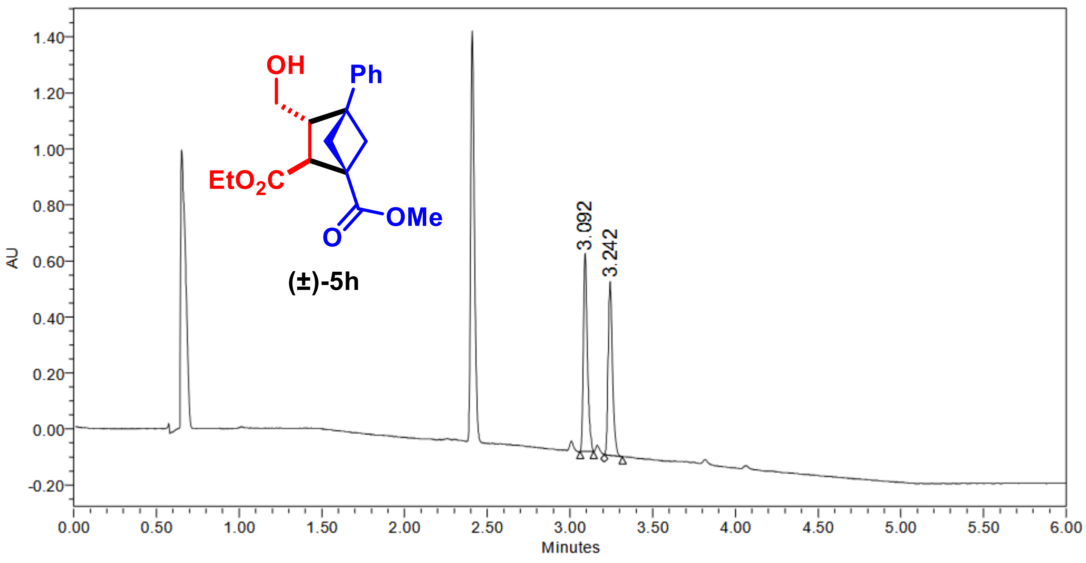

	Retention Time (min)	% Area
1	3.242	48.10
2	3.092	51.90

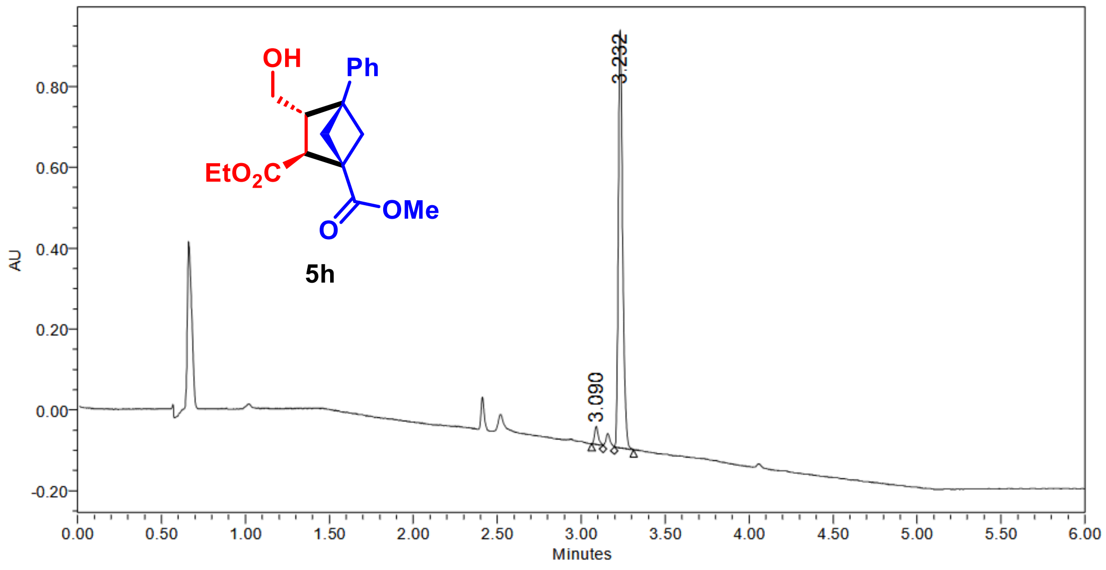

	Retention Time (min)	% Area
1	3.090	3.69
2	3.232	96.31

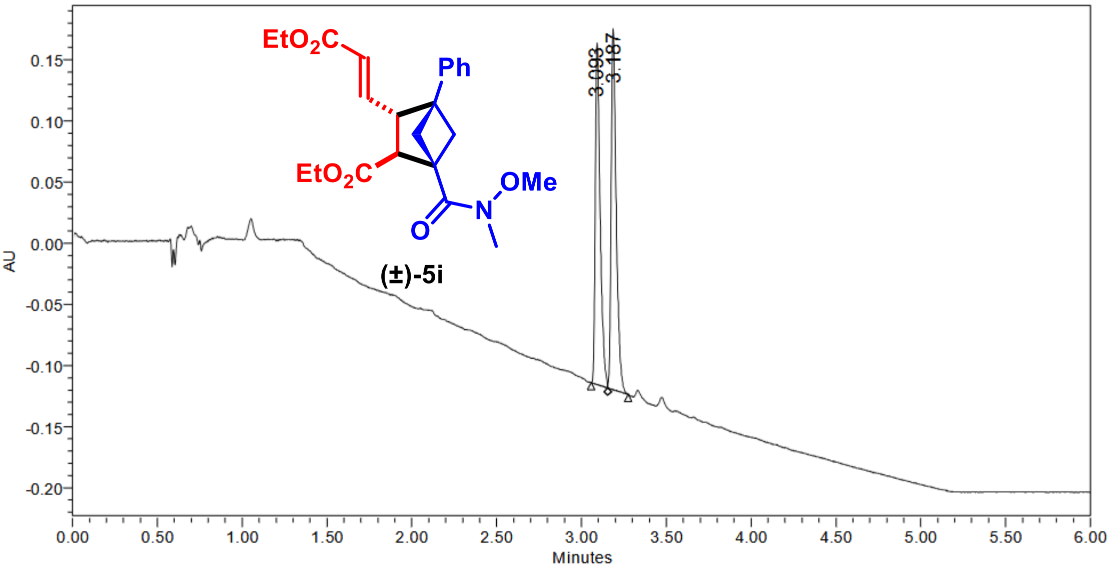

	Retention Time (min)	% Area
1	3.093	47.56
2	3.187	52.44

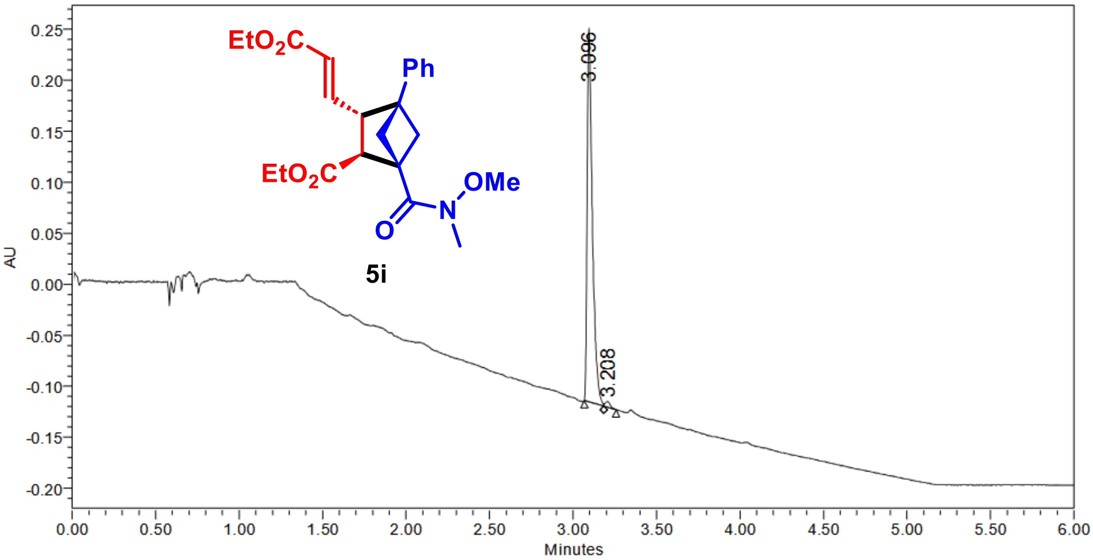

	Retention Time (min)	% Area
1	3.096	98.56
2	3.208	1.44

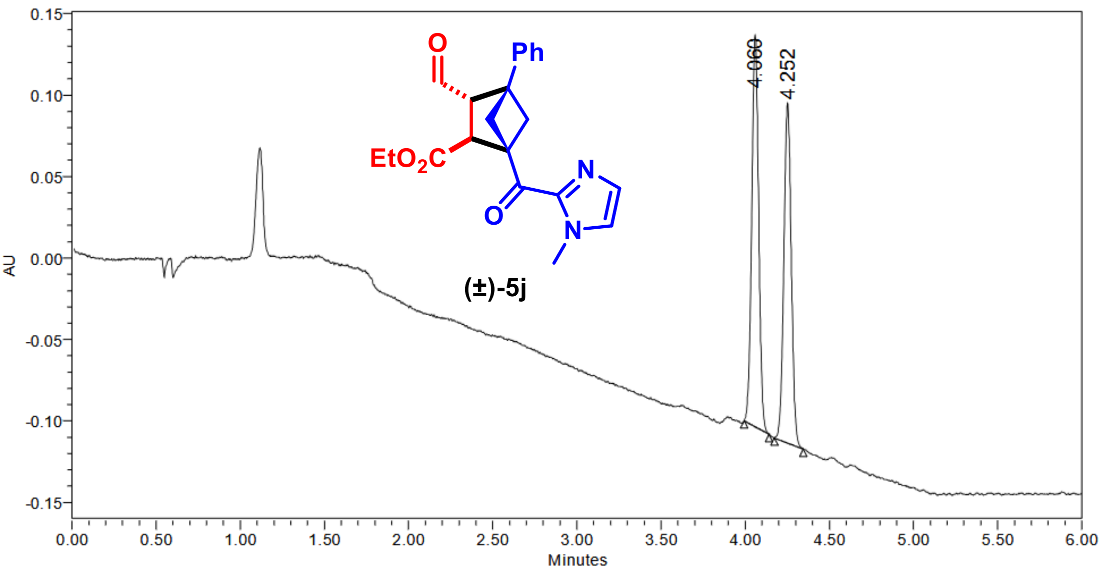

	Retention Time (min)	% Area
1	4.060	51.76
2	4.252	48.24

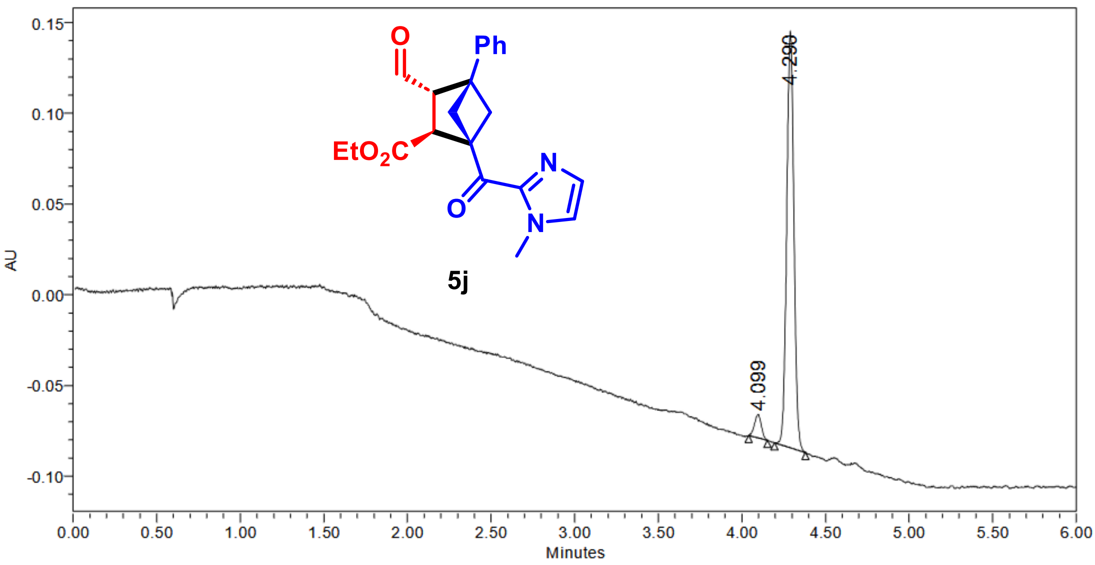

	Retention Time (min)	% Area
1	4.099	5.04
2	4.290	94.96

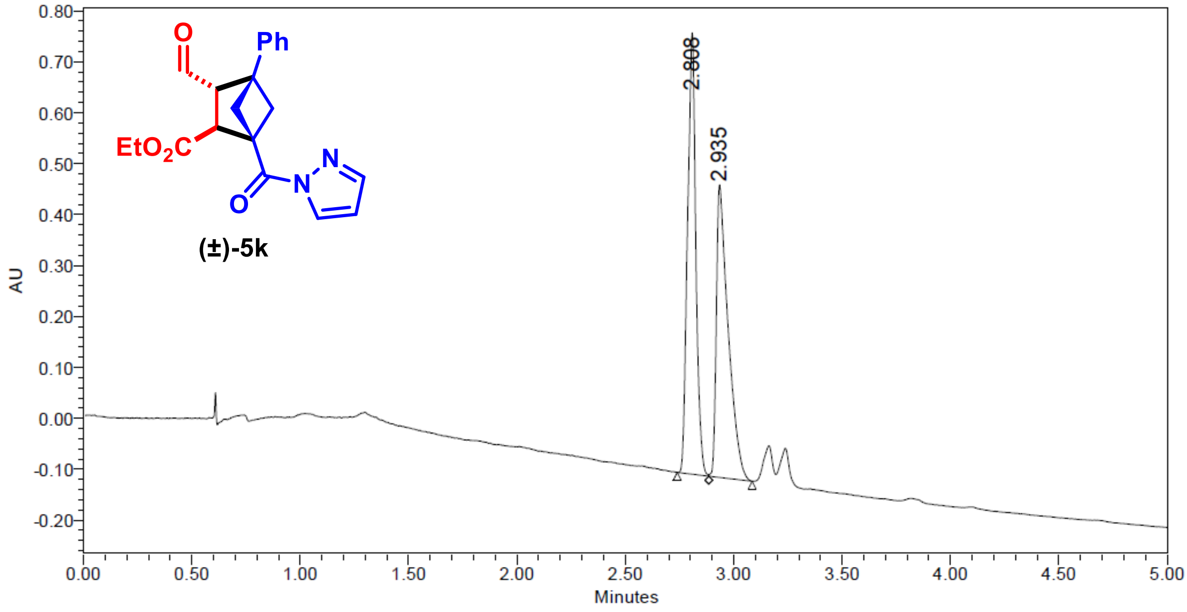

	Retention Time (min)	% Area
1	2.808	51.50
2	2.935	48.50

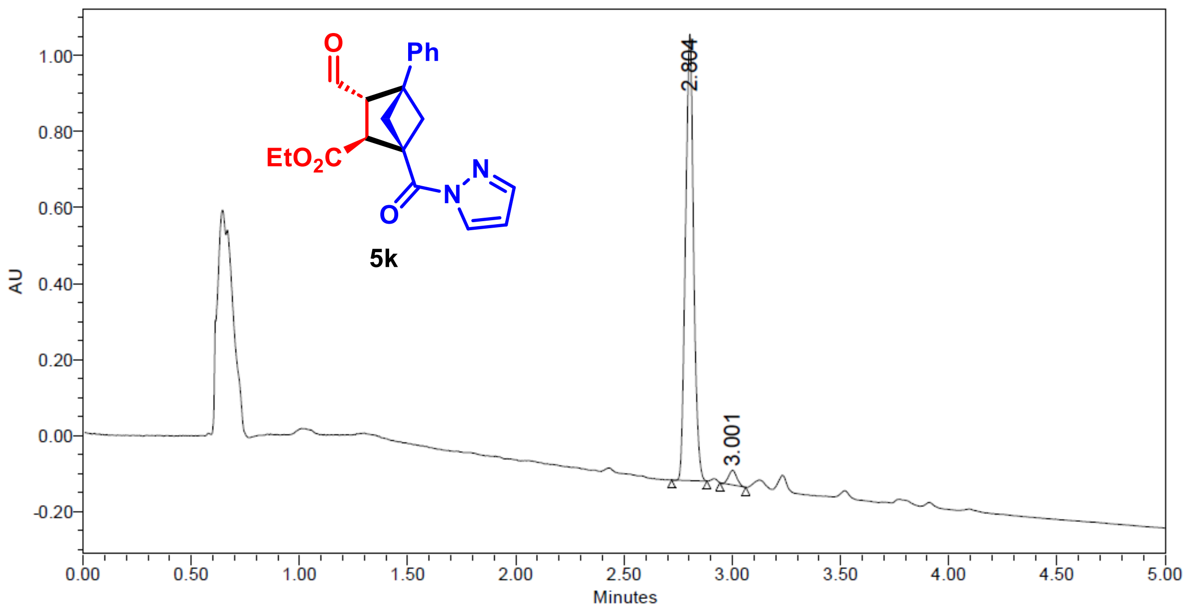

	Retention Time (min)	% Area
1	2.804	96.78
2	3.001	3.22

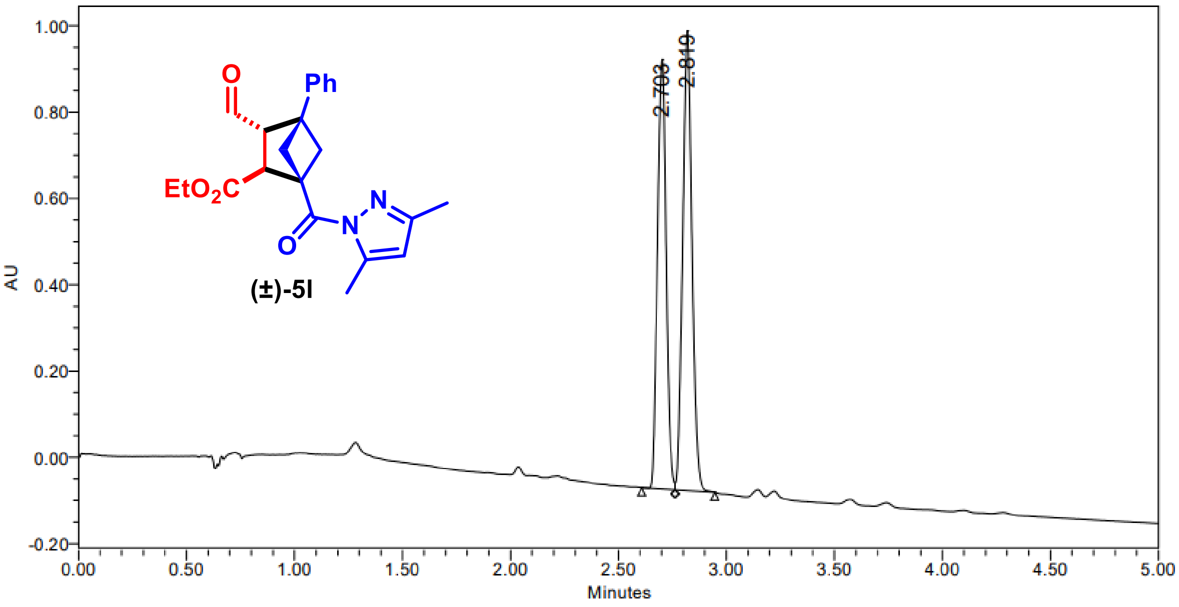

	Retention Time (min)	% Area
1	2.703	46.14
2	2.819	53.86

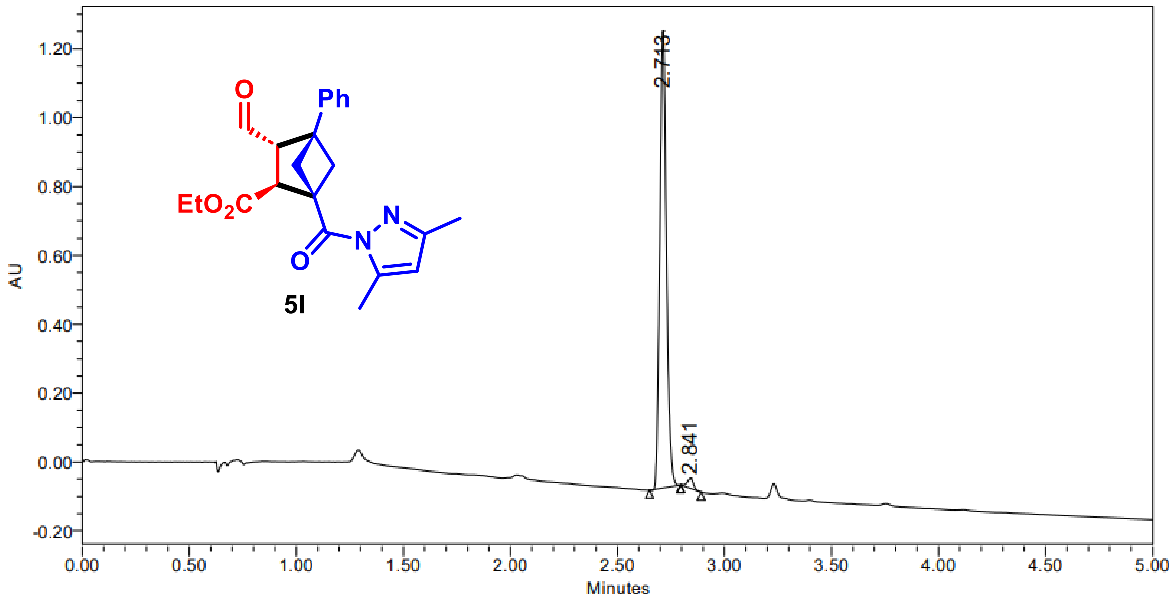

	Retention Time (min)	% Area
1	2.713	98.05
2	2.841	1.95

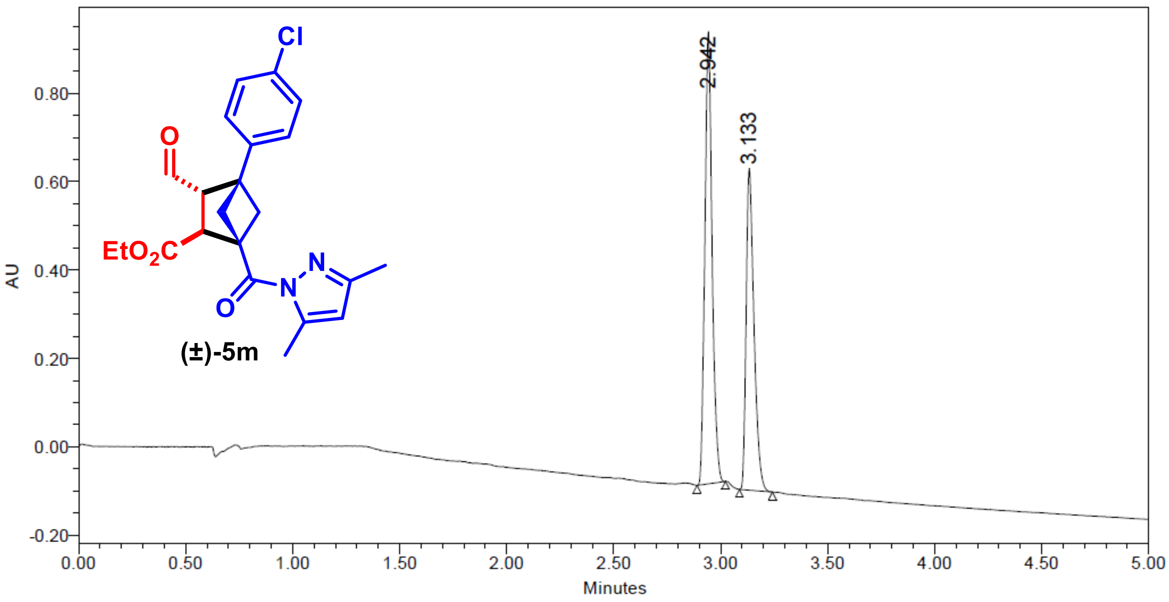

	Retention Time (min)	% Area
1	2.942	56.69
2	3.133	43.31

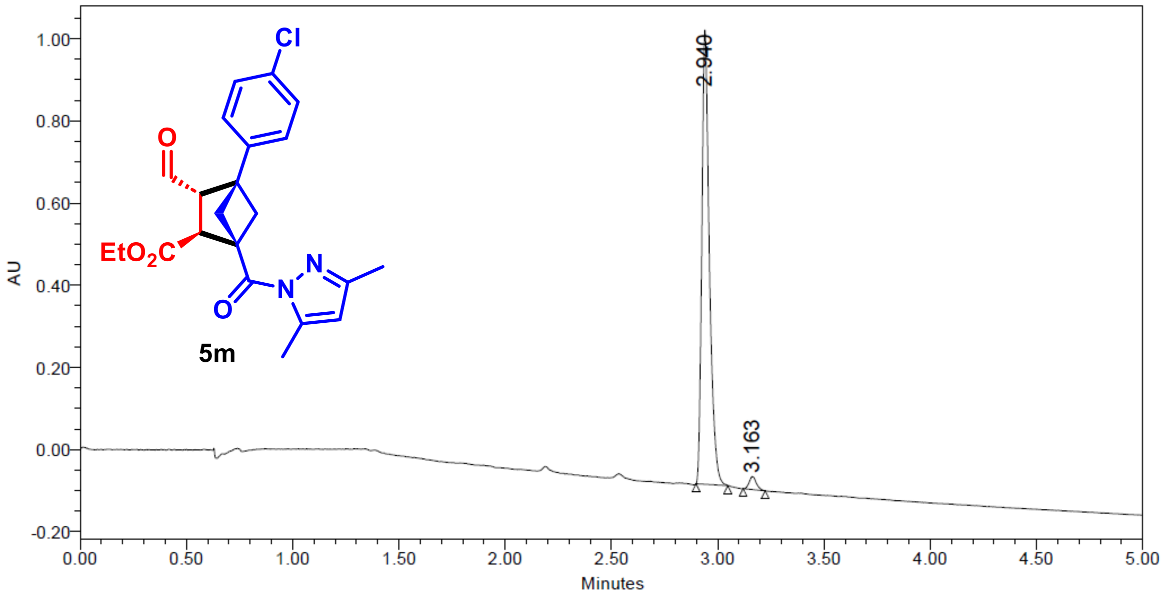

	Retention Time (min)	% Area
1	2.940	97.35
2	3.163	2.65

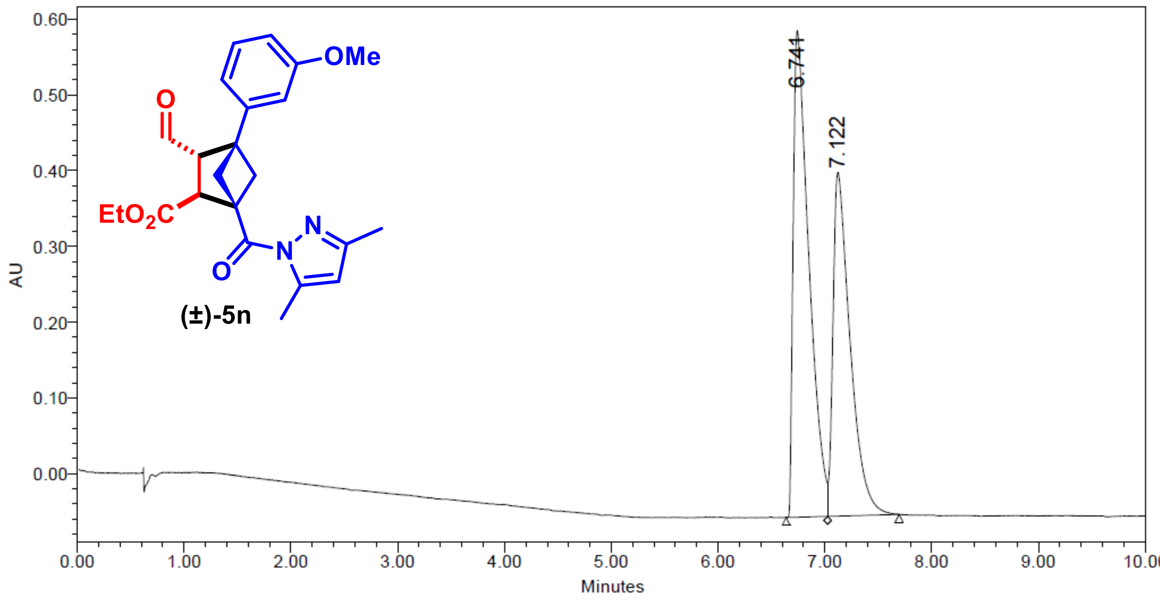

	Retention Time (min)	% Area
1	6.741	56.50
2	7.122	43.50

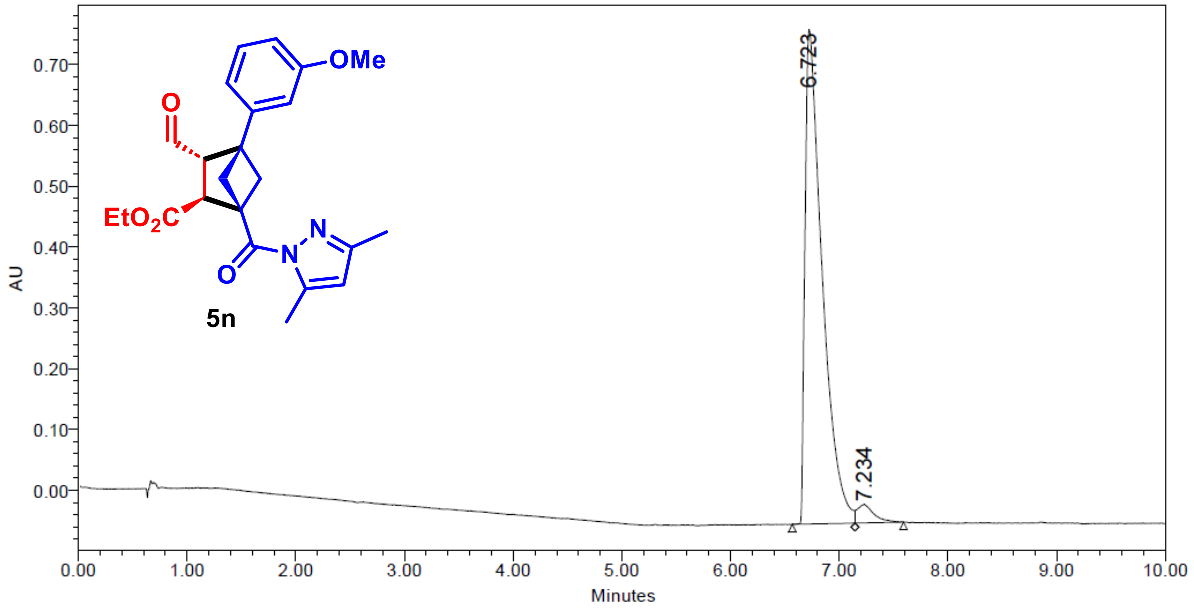

	Retention Time (min)	% Area
1	6.723	96.51
2	7.234	3.49

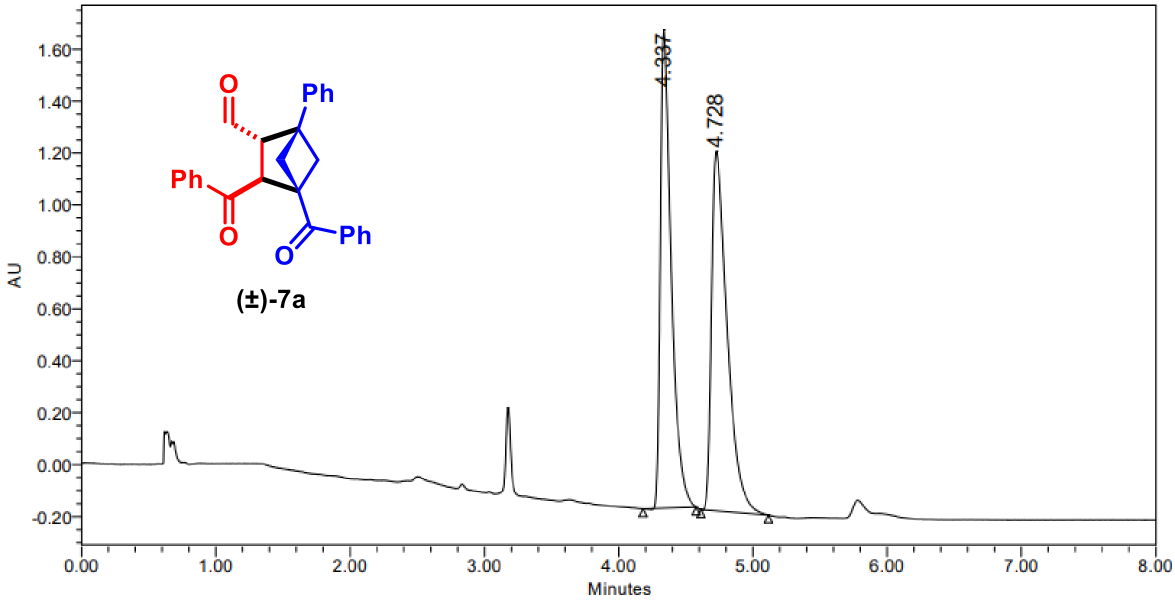

	Retention Time (min)	% Area
1	4.337	48.39
2	4.728	51.61

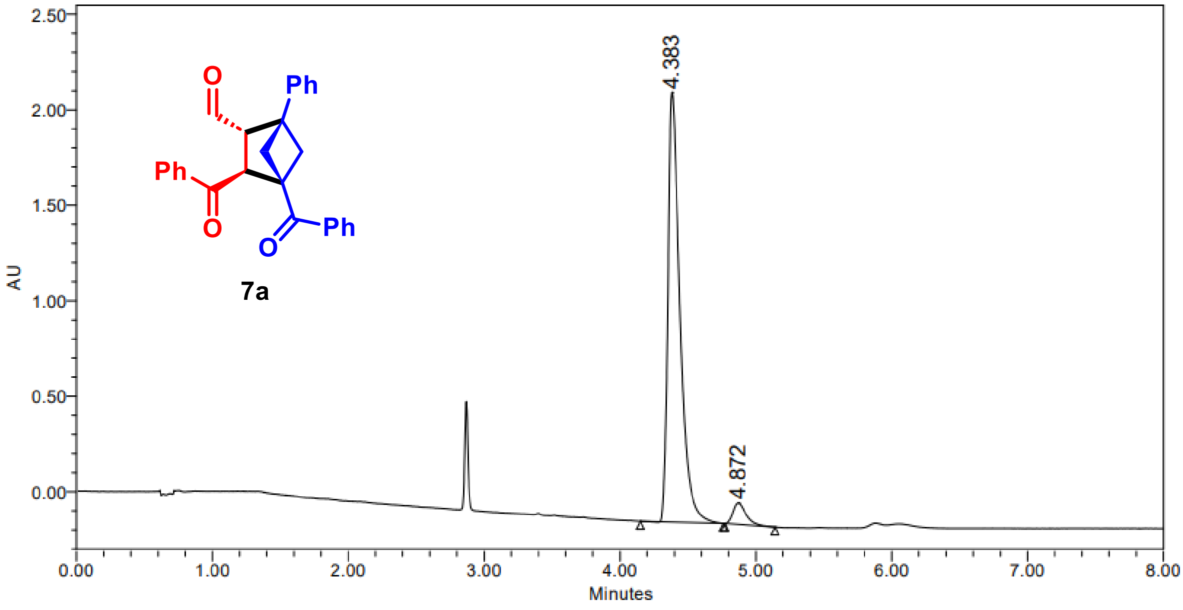

	Retention Time (min)	% Area
1	4.383	94.92
2	4.872	5.08

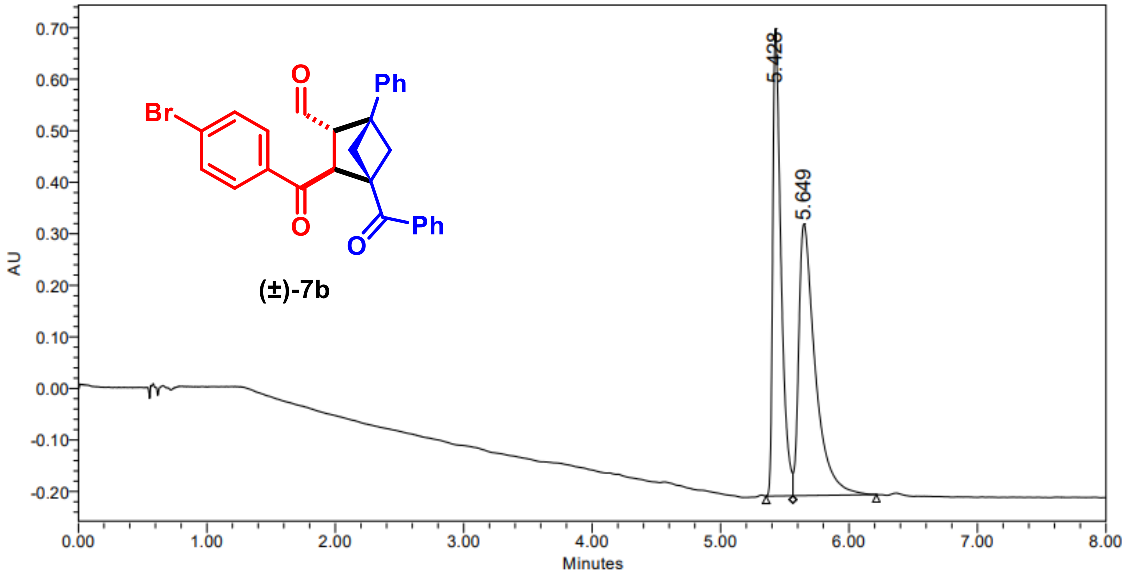

	Retention Time (min)	% Area
1	5.428	46.31
2	5.649	53.69

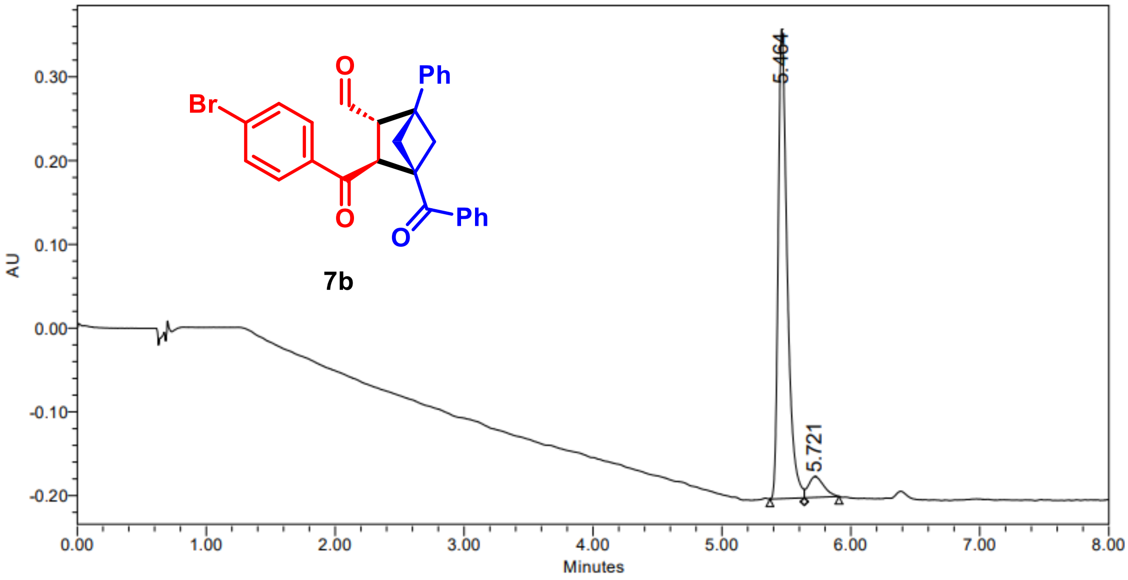

	Retention Time (min)	% Area
1	5.464	93.01
2	5.721	6.99

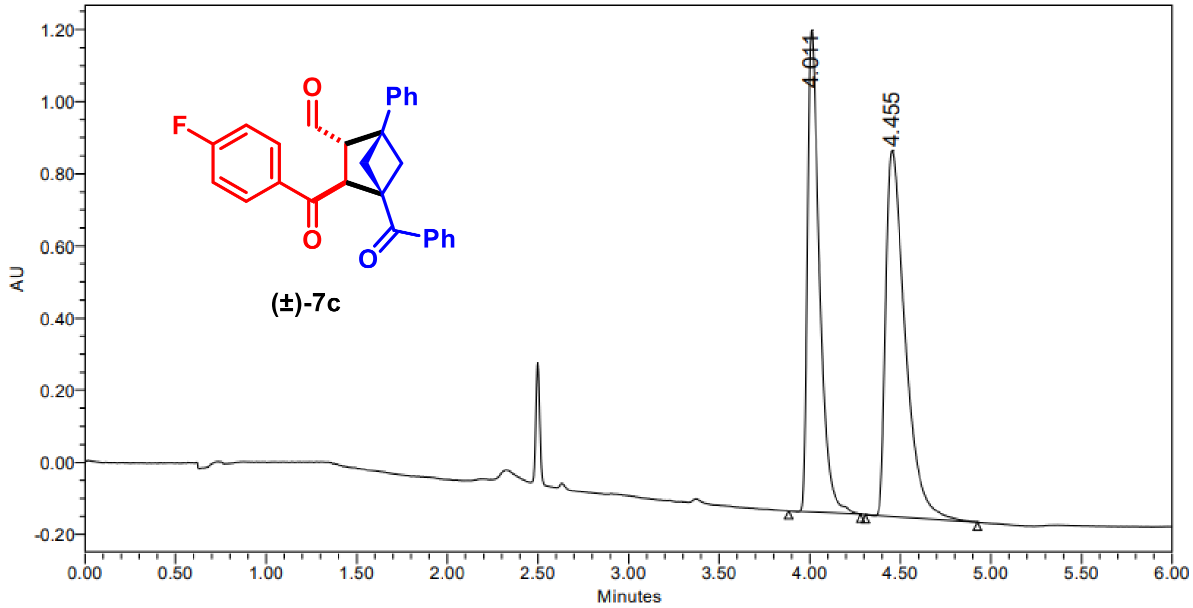

	Retention Time (min)	% Area
1	4.011	45.08
2	4.455	54.92

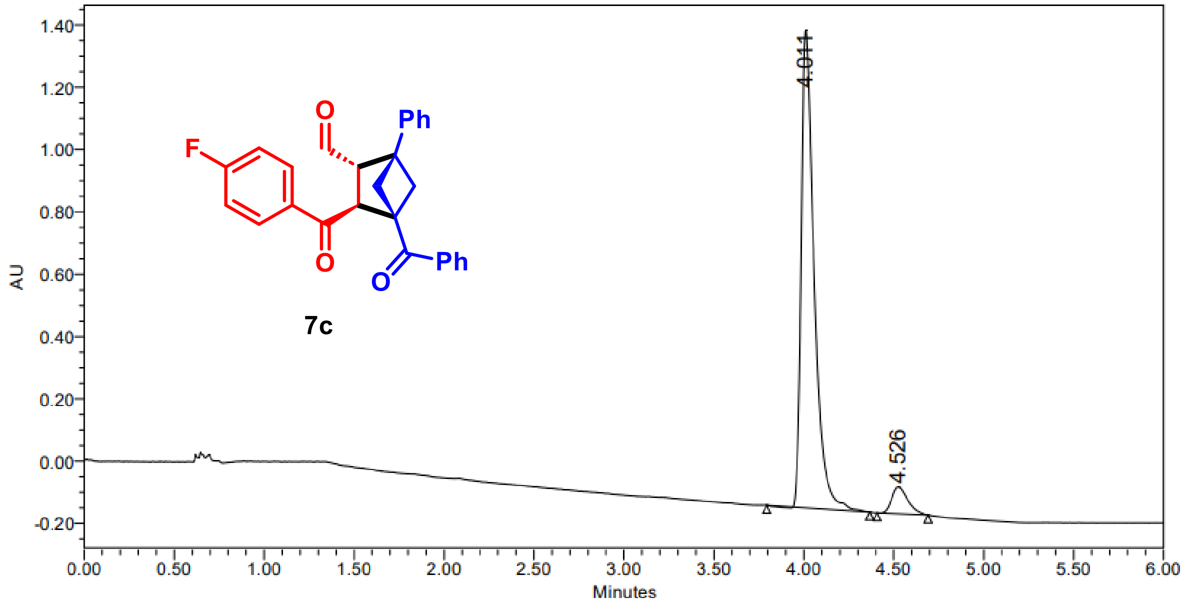

	Retention Time (min)	% Area
1	4.011	93.49
2	4.526	6.51

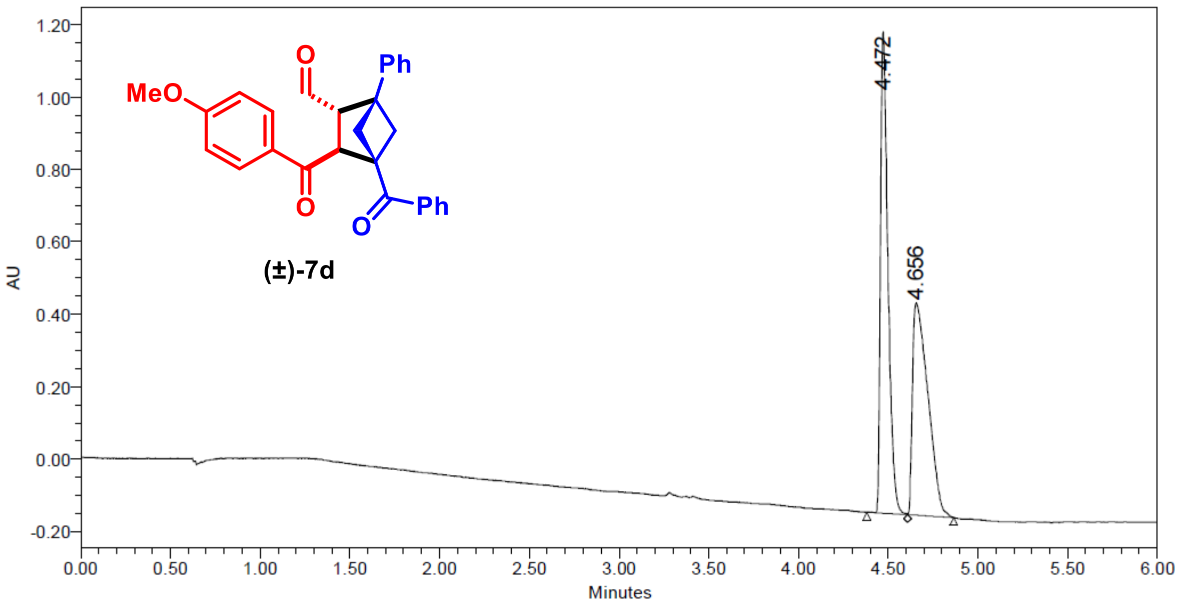

	Retention Time (min)	% Area
1	4.472	52.27
2	4.656	47.73

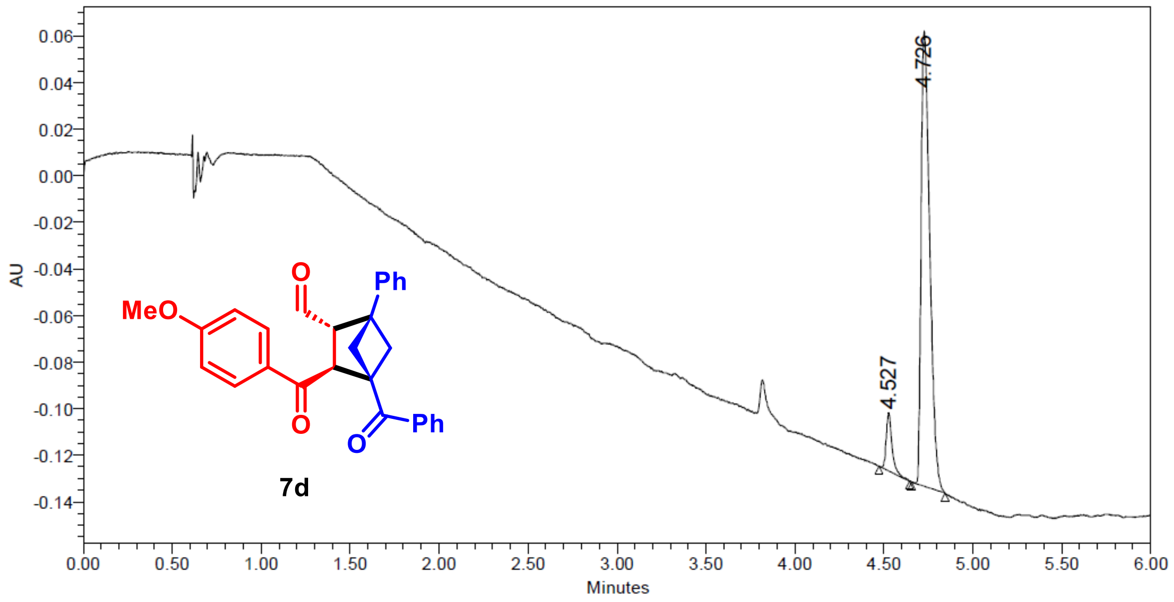

	Retention Time (min)	% Area
1	4.527	7.87
2	4.726	92.13

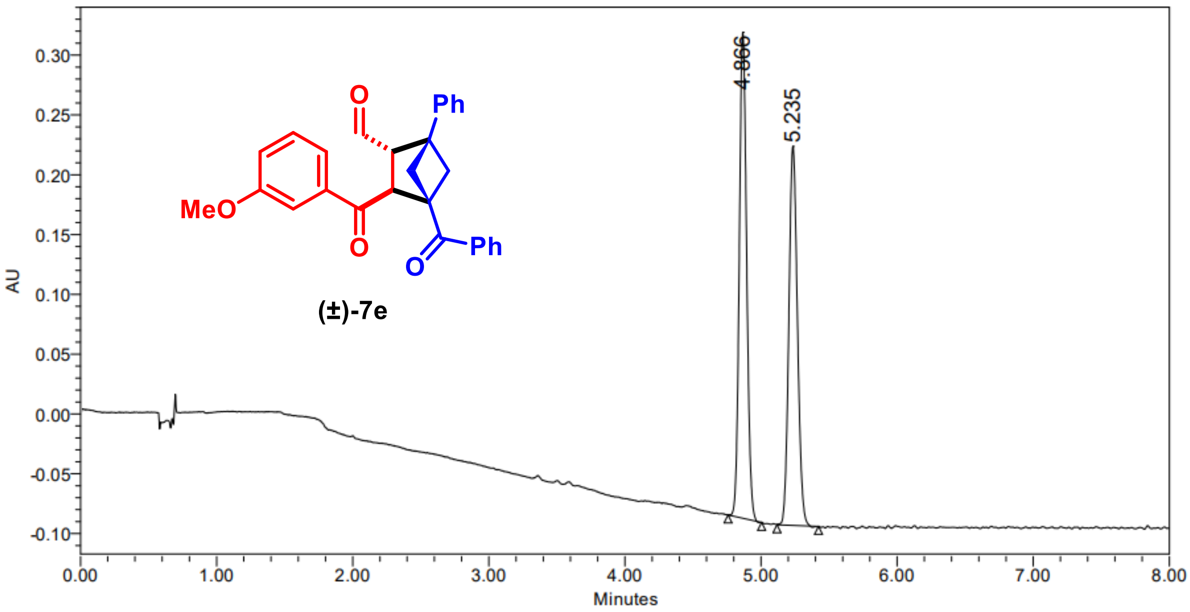

	Retention Time (min)	% Area
1	4.866	52.36
2	5.235	47.64

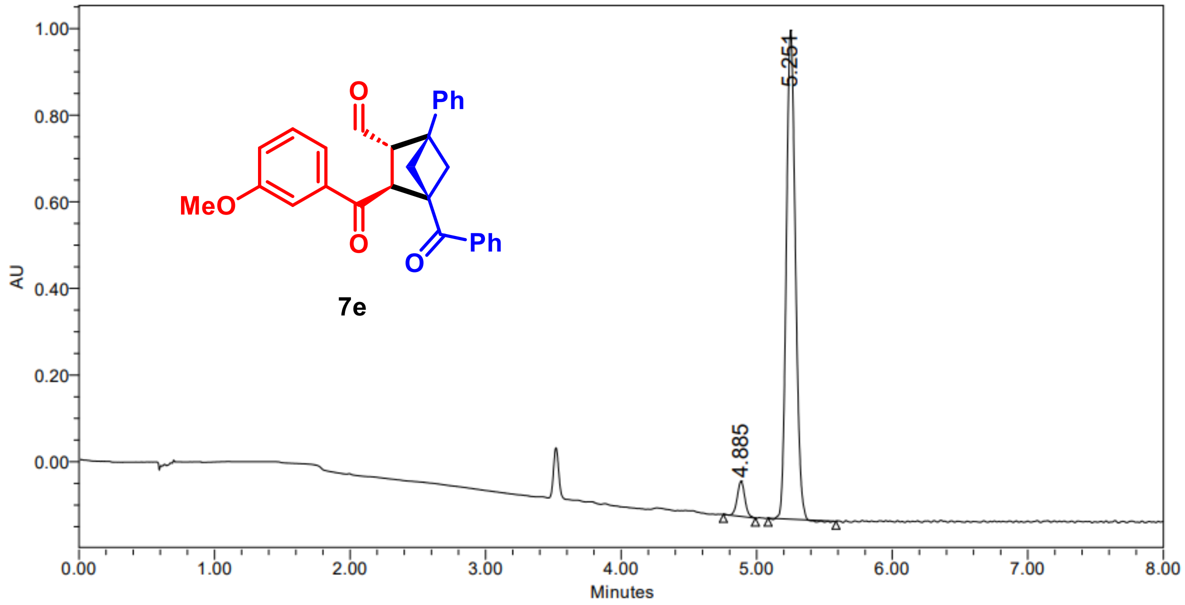

	Retention Time (min)	% Area
1	4.885	5.67
2	5.251	94.33

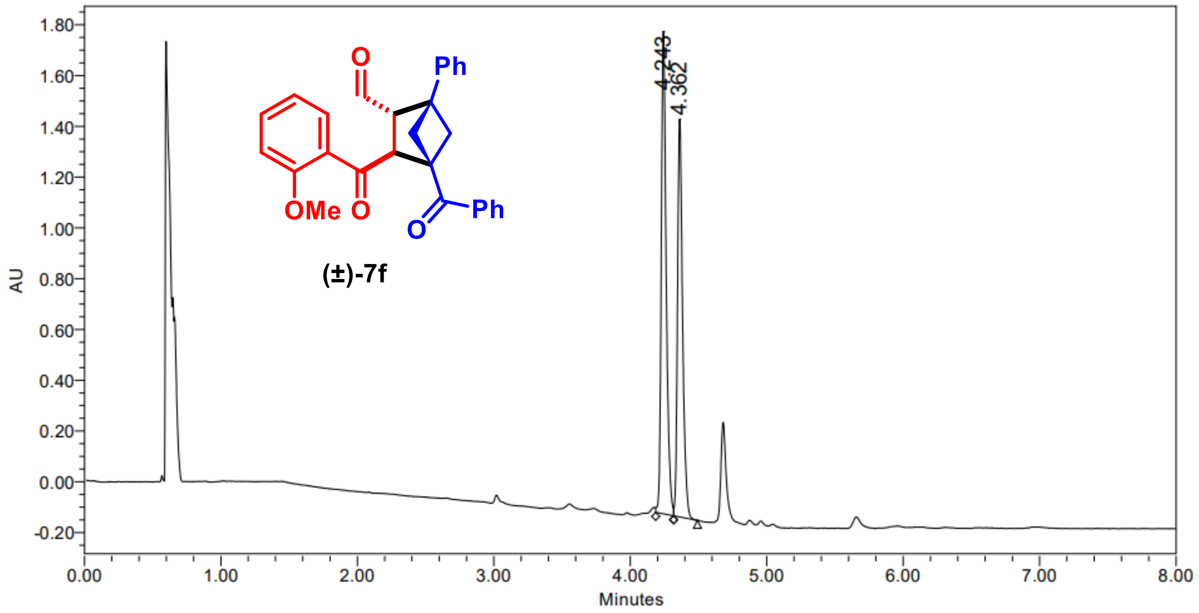

	Retention Time (min)	% Area
1	4.243	54.15
2	4.362	45.85

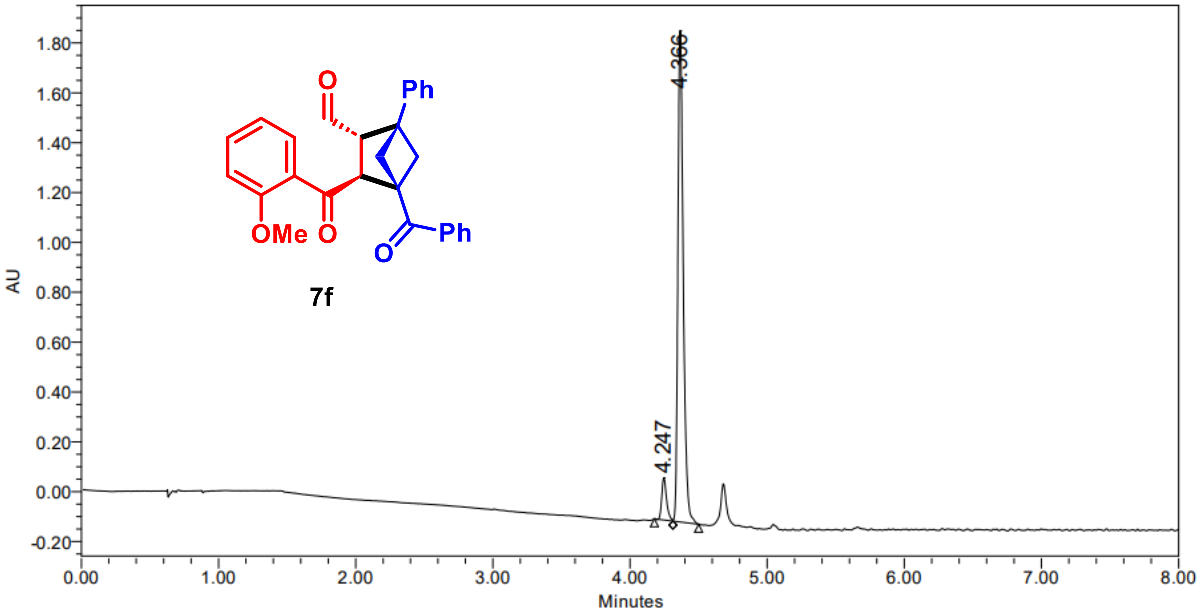

	Retention Time (min)	% Area
1	4.247	7.13
2	4.366	92.87

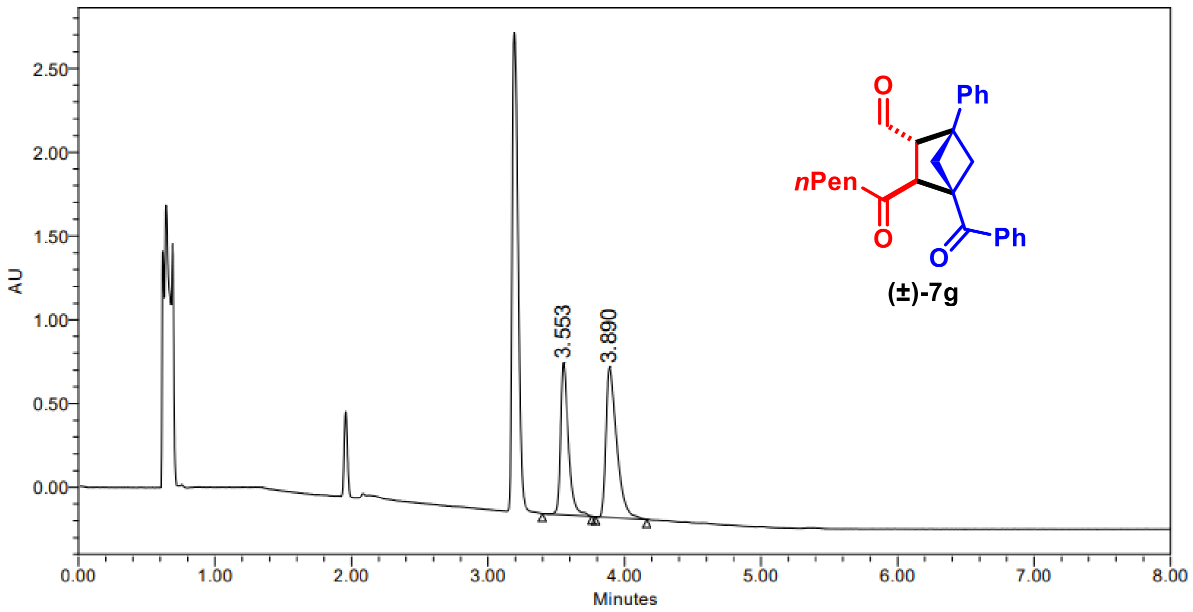

	Retention Time (min)	% Area
1	3.553	42.19
2	3.890	57.81

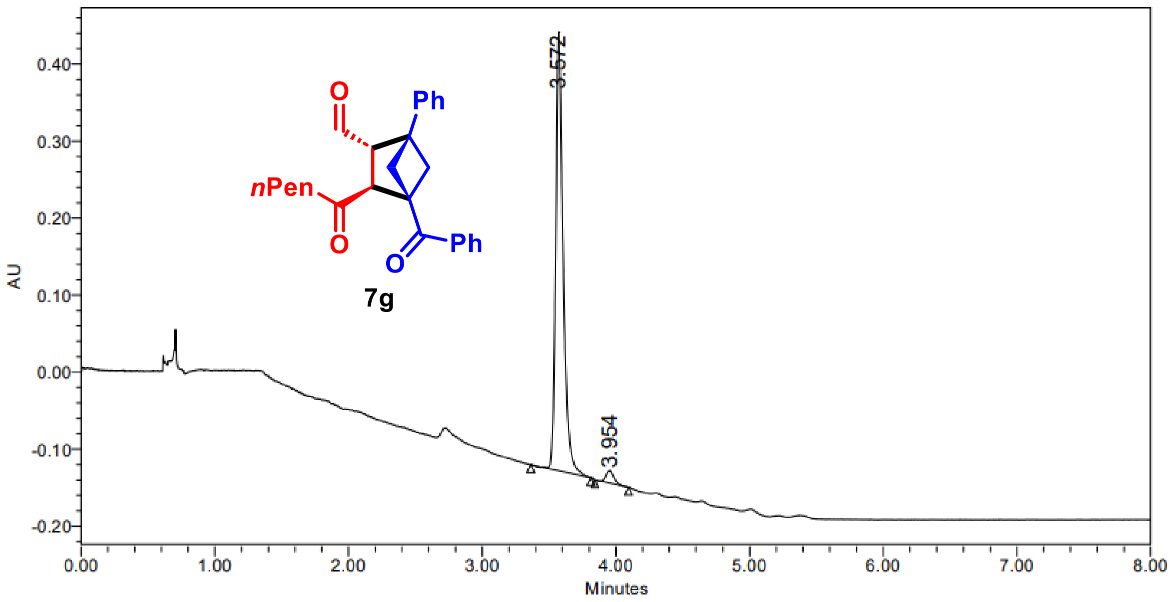

	Retention Time (min)	% Area
1	3.572	97.03
2	3.954	2.97

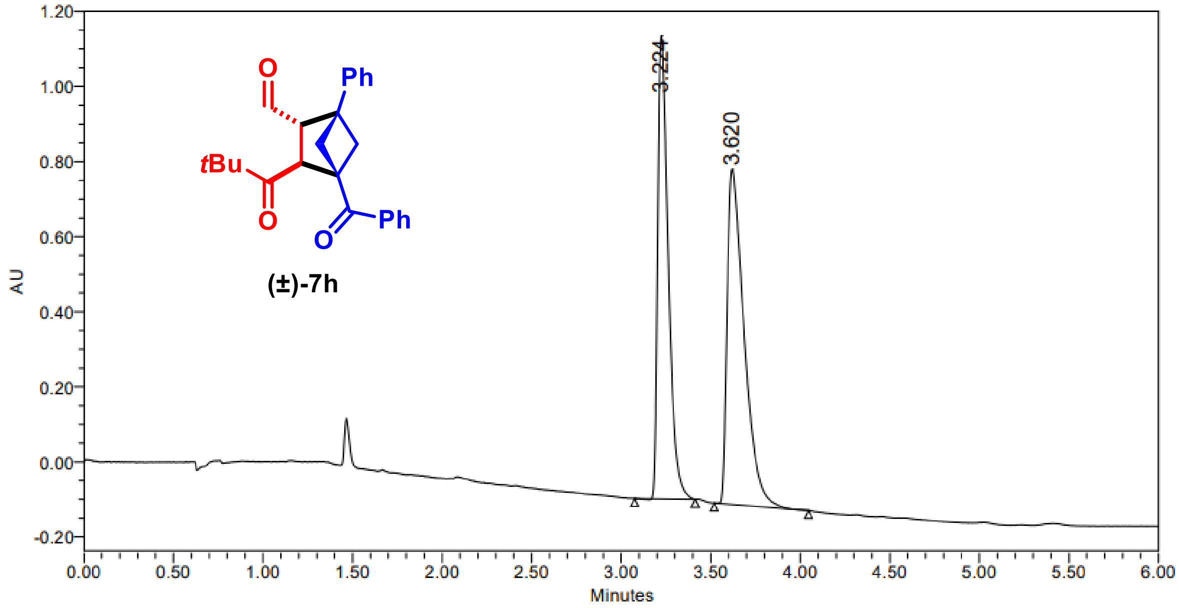

	Retention Time (min)	% Area
1	3.224	46.37
2	3.620	53.63

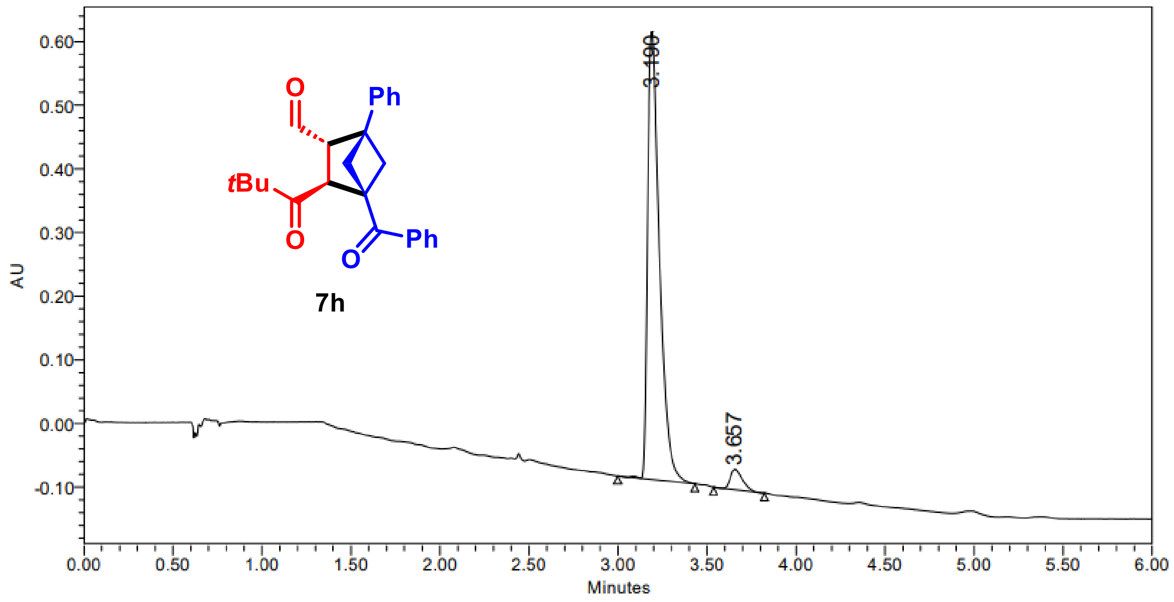

	Retention Time (min)	% Area
1	3.190	95.57
2	3.657	4.43

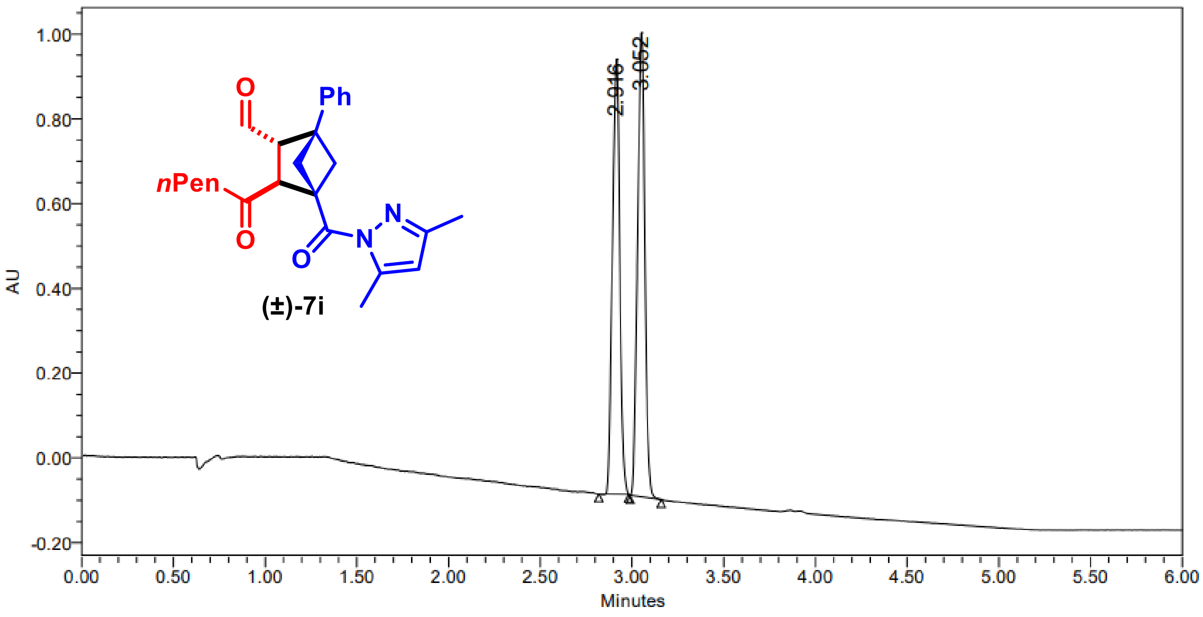

	Retention Time (min)	% Area
1	2.916	48.49
2	3.052	51.51

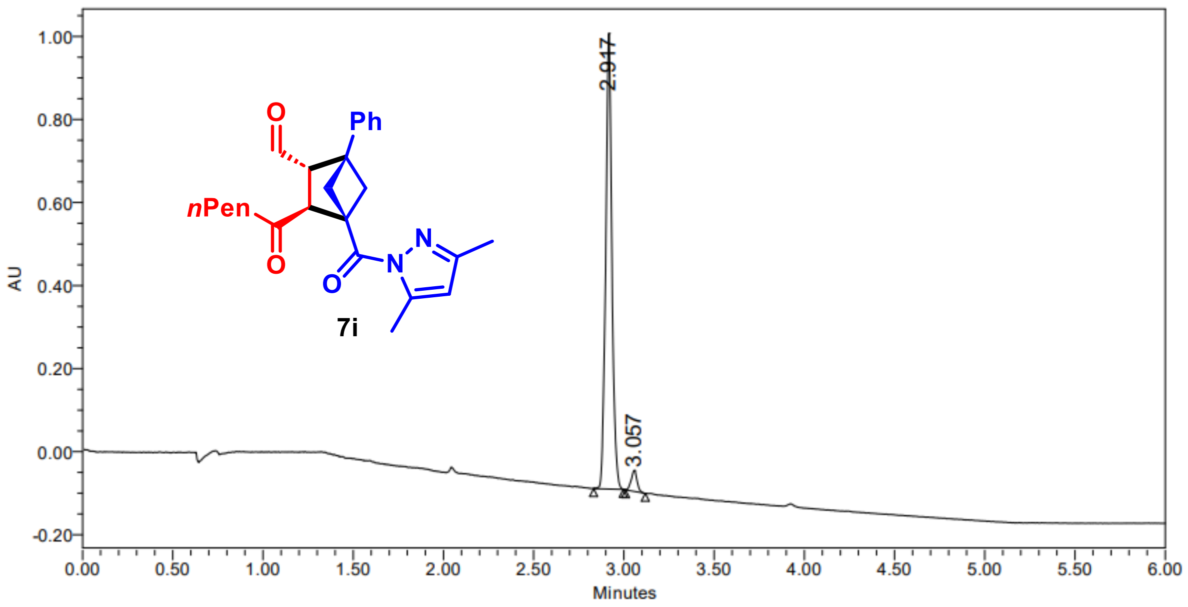

	Retention Time (min)	% Area
1	2.917	95.87
2	3.057	4.13

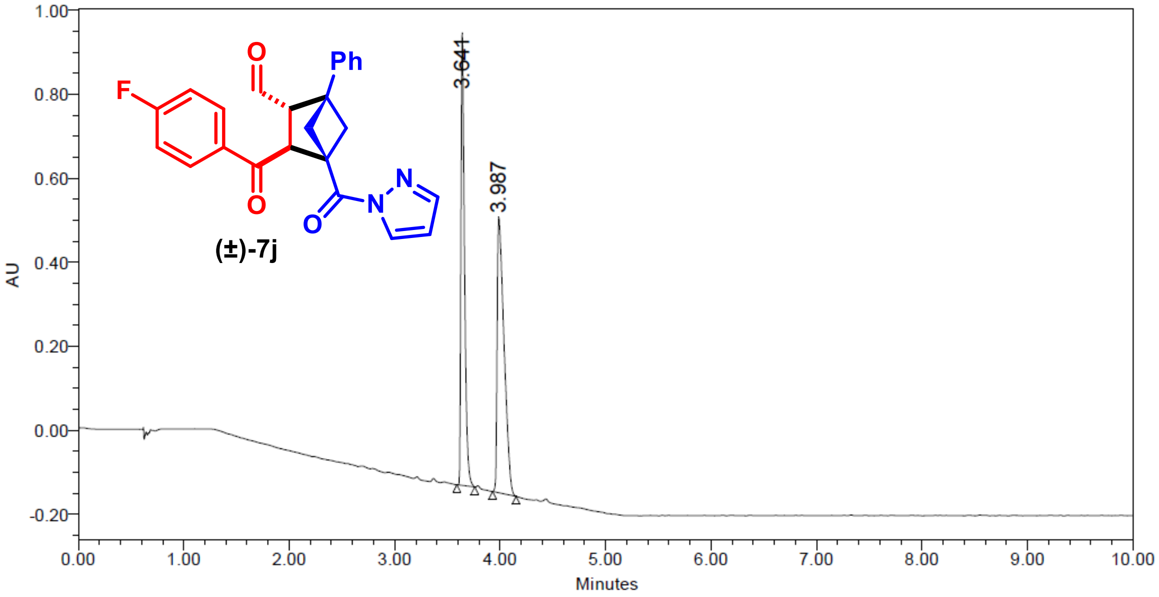

	Retention Time (min)	% Area
1	3.641	46.13
2	3.987	53.87

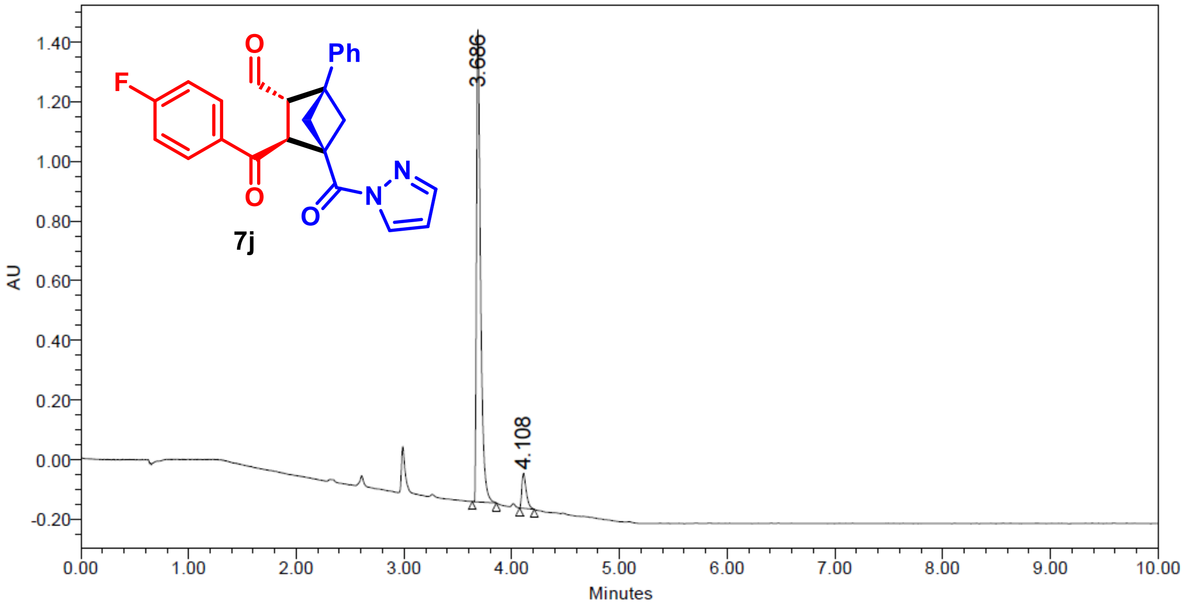

	Retention Time (min)	% Area
1	3.686	92.59
2	4.108	7.41

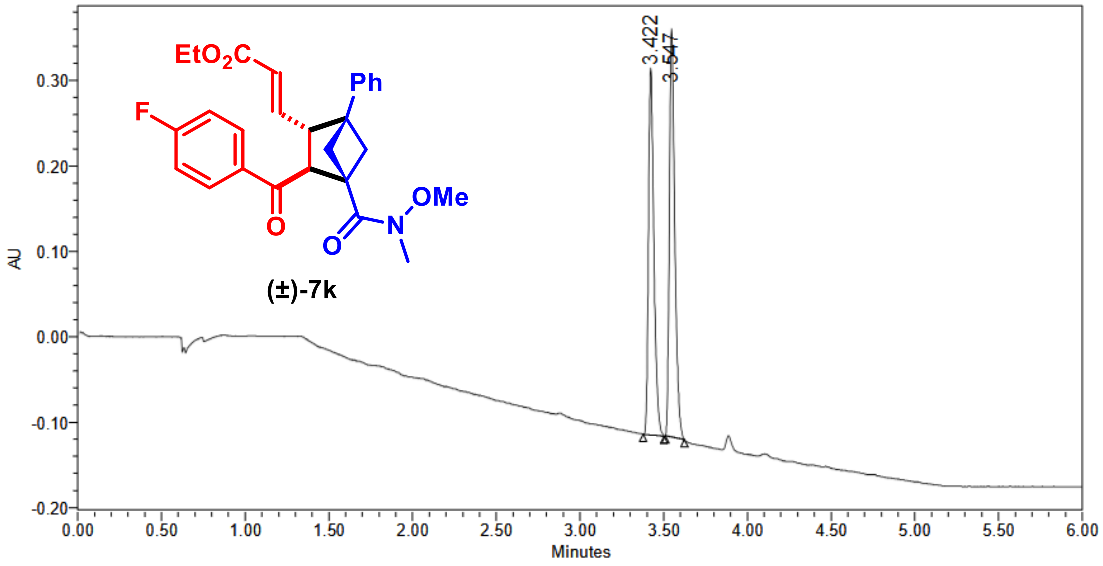

	Retention Time (min)	% Area
1	3.422	46.76
2	3.547	53.24

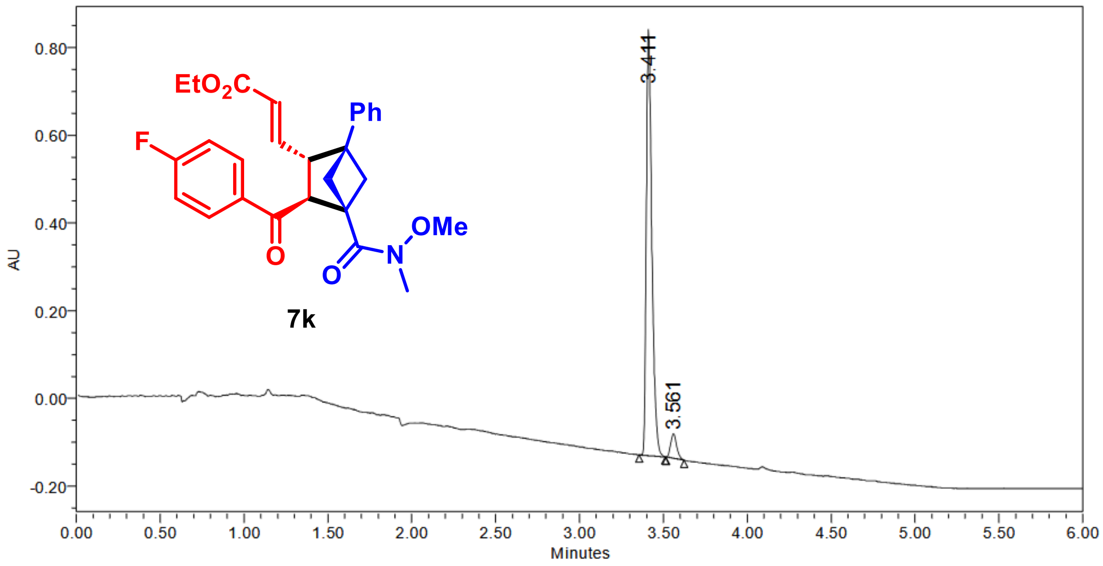

	Retention Time (min)	% Area
1	3.411	93.93
2	3.561	6.07

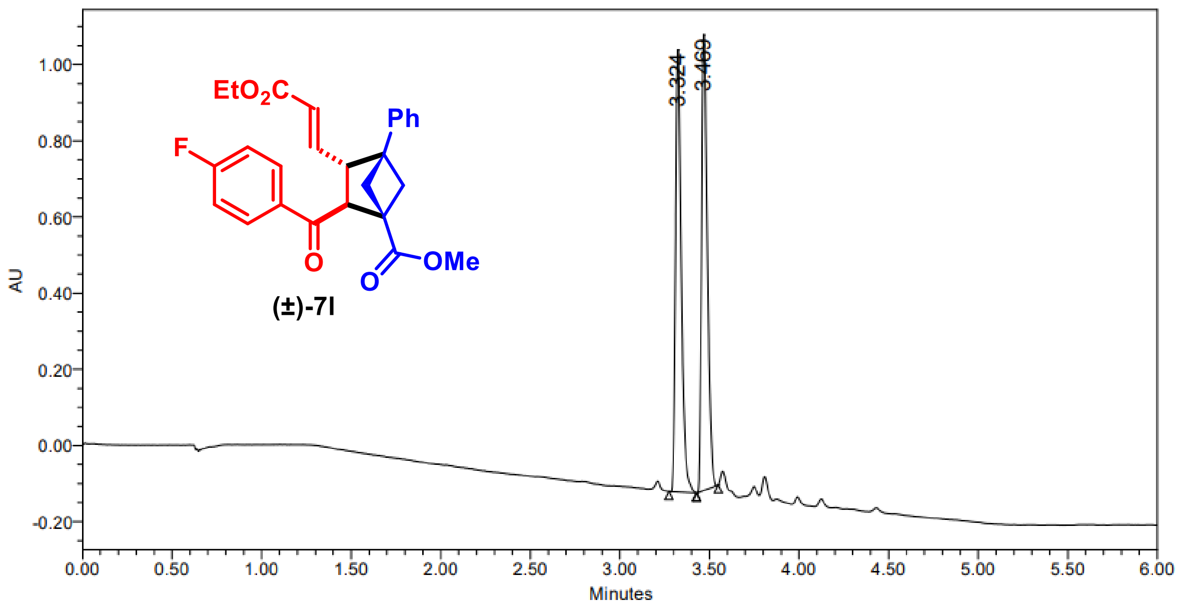

	Retention Time (min)	% Area
1	3.324	48.97
2	3.469	51.03

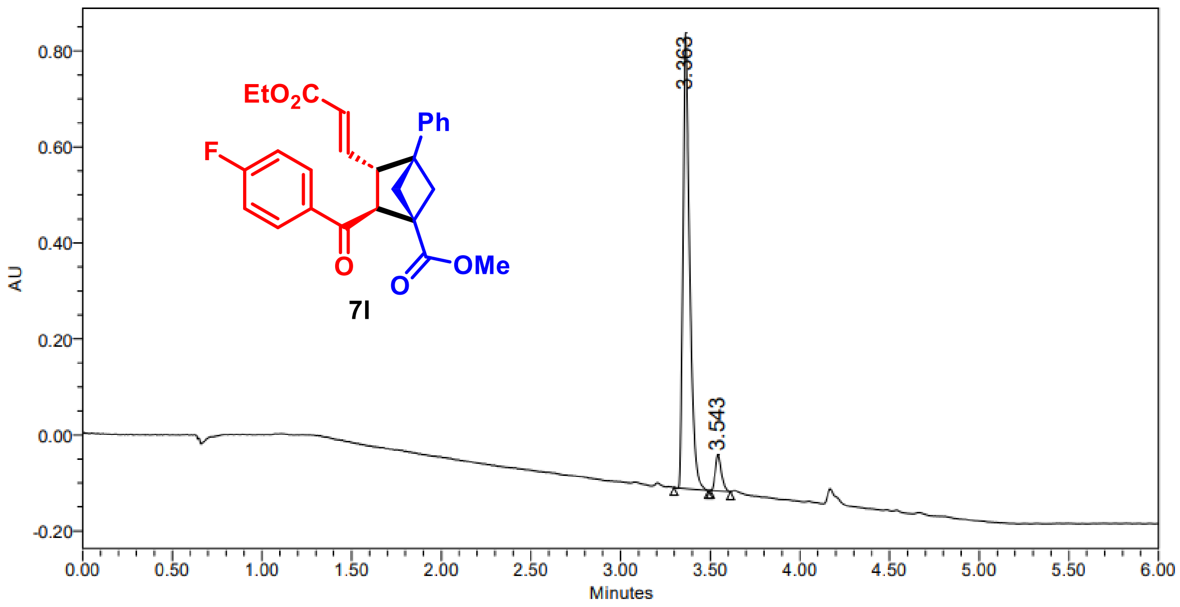

	Retention Time (min)	% Area
1	3.363	93.37
2	3.543	6.63

9. Geometries XYZ

Images were formed using CYLView.[11] Gibbs free energies reported are in units of Hartree.

3b_1

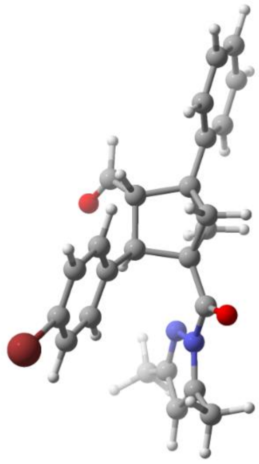

Charge = 0, **Multiplicity** = 1
Gibbs free energy = -3799.4522

No imaginary frequency found

C	0.51409500	0.00226300	0.65443500
C	0.97931500	0.66626800	-0.68117000
C	2.40014300	1.14960900	-0.29017900
C	2.68215300	-0.37592500	-0.26057900
C	1.78107500	-0.83982800	0.92899400
H	0.43159800	0.78224000	1.41456900
H	2.91394400	1.64432200	-1.11832400
H	2.50093100	1.72486700	0.63290100
H	1.62178500	-1.92313300	0.85045800
C	4.05916500	-0.95134300	-0.34495000
C	5.17963300	-0.18926800	-0.01158600
C	4.23796700	-2.27925900	-0.74427100
C	6.45681500	-0.74269400	-0.07425200
H	5.05302400	0.84568400	0.30078900
C	5.51196800	-2.83321300	-0.80926200
H	3.37015300	-2.88217800	-1.00750700
C	6.62700300	-2.06571800	-0.47370900
H	7.32123000	-0.13632000	0.18818700
H	5.63654400	-3.86679800	-1.12557300
H	7.62414100	-2.49765500	-0.52649800
C	-0.82389300	-0.68872600	0.55861200
C	-0.99419400	-2.00158500	0.11501300
C	-1.96788200	0.05444100	0.87077800
C	-2.26323200	-2.55668600	-0.02579100
H	-0.13543100	-2.62119600	-0.12813400
C	-3.24341900	-0.47907900	0.73365500
H	-1.86135300	1.07599200	1.23164000
C	-3.37565000	-1.78541000	0.28027500
H	-2.37141600	-3.58103800	-0.37073000
H	-4.11460200	0.11950100	0.98362700
C	-0.04830100	1.47566900	-1.41597600
C	2.42634700	-0.61760300	2.26795700
H	3.36775300	-1.18255700	2.42574400
O	1.99532400	0.10068000	3.14308200
O	-0.47848800	1.14599200	-2.50077100
C	-1.69720400	3.32458100	-1.07062700
C	-1.80645500	4.27145300	-0.09106900
C	-0.69485900	4.07597400	0.77823100
H	-2.59027900	5.01320300	-0.00121000
N	-0.55395200	2.60772800	-0.76522500
N	0.04872400	3.07093500	0.37237200
Br	-5.12384100	-2.53841000	0.08328200
C	1.67244700	-0.48793800	-1.43099100
C	-2.59734800	3.04252800	-2.22054900
H	-2.08314600	3.18344000	-3.17603100
H	-2.96127700	2.01011100	-2.19225700
H	-3.45153800	3.72353600	-2.17079800
C	-0.34709100	4.85024800	2.00271700
H	-1.17017300	4.81221800	2.72591400
H	0.55373500	4.44279200	2.47106900
H	-0.17352000	5.90435400	1.75690900
H	2.11202600	-0.15671400	-2.37502700
H	1.13065400	-1.42687500	-1.56733000

3b_2

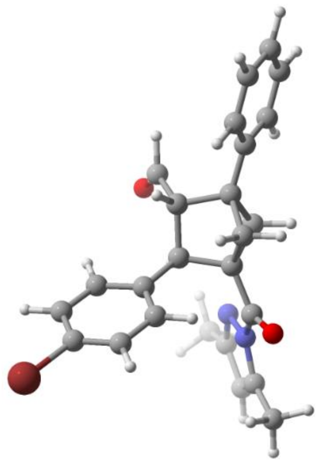

Charge = 0, **Multiplicity** = 1
Gibbs free energy = -3799.4503

No imaginary frequency found

C	0.37615000	0.00542600	0.51784300
C	1.02755800	0.76306500	-0.66625700
C	2.46404600	0.98863700	-0.11409900
C	2.55224700	-0.54720200	-0.28850900
C	1.50336100	-1.03877900	0.75749000
H	0.32872700	0.67249500	1.38153200
H	3.09999400	1.51954700	-0.82709000
H	2.55774100	1.41512000	0.88724200
H	1.19552000	-2.06172700	0.50284300
C	3.85033900	-1.28574100	-0.35553000
C	3.90126600	-2.56179900	-0.92440200
C	5.01970700	-0.73069300	0.16596200
C	5.09832500	-3.26841100	-0.96992800
H	2.99441100	-3.00389800	-1.33419000
C	6.21948100	-1.43778800	0.12338200
H	4.99223700	0.26242100	0.61062000
C	6.26264500	-2.70823100	-0.44500700
H	5.12427400	-4.25943700	-1.41831700
H	7.12317200	-0.99257400	0.53438200
H	7.19947700	-3.26019800	-0.48153800
C	-0.99683800	-0.59084600	0.32496300
C	-1.63203700	-0.70051600	-0.91222100
C	-1.67033700	-1.06256000	1.45672000
C	-2.90233400	-1.26197800	-1.02377200
H	-1.14969100	-0.34827800	-1.81923200
C	-2.93480900	-1.62973800	1.36676500
H	-1.19853900	-0.97990800	2.43404900
C	-3.53957100	-1.72272400	0.11829000
H	-3.37882700	-1.33330500	-1.99730100
H	-3.43799300	-1.98643300	2.26111700
C	0.26587900	1.86055900	-1.35482200
C	2.06159100	-1.09428500	2.15115000
H	2.85883800	-1.85121700	2.29750300
O	1.70990000	-0.39592200	3.07652700
O	0.01586500	1.83443400	-2.54187500
C	-1.02410200	3.95971300	-0.91638300
C	-1.12944400	4.74462400	0.19694000
C	-0.30192000	4.14402200	1.18898300
H	-1.73053700	5.64016500	0.29455500
N	-0.16163000	2.93480900	-0.56581600
N	0.27279400	3.05679400	0.72672800
Br	-5.28434500	-2.49660400	-0.02539700
C	1.64127100	-0.36617900	-1.52763300
C	-1.68636100	4.10289000	-2.24038500
H	-0.95467300	4.26733600	-3.03723500
H	-2.25610900	3.20428400	-2.49808100
H	-2.36697800	4.95789400	-2.19940500
C	-0.06276700	4.61183200	2.58304800
H	-1.00788400	4.67075700	3.13554400
H	0.61067800	3.92697800	3.10698500
H	0.38091600	5.61443800	2.58272100
H	2.19592600	0.02363400	-2.38438700
H	1.00705100	-1.20604400	-1.82254100

3b_3

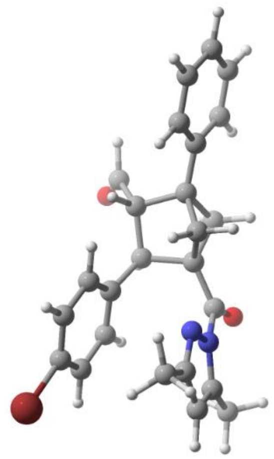

Charge = 0, **Multiplicity** = 1
Gibbs free energy = -3799.4527

No imaginary frequency found

C	0.84142100	0.02182200	1.22388400
C	1.19661500	1.20075200	0.24995400
C	2.66690300	1.47359300	0.63736000
C	2.88527100	0.08312600	-0.00684500
C	2.10503700	-0.83691800	0.98419700
H	0.85567700	0.41066600	2.24721400
H	3.10494500	2.27691900	0.03908500
H	2.90141700	1.60801400	1.69773400
H	1.90252500	-1.79679500	0.49256900
C	4.21543400	-0.39918200	-0.48888600
C	5.40223400	0.17664600	-0.03388300
C	4.28266800	-1.45876400	-1.39886400
C	6.63516900	-0.29674500	-0.47851800
H	5.36311700	1.00218200	0.67435900
C	5.51187300	-1.93121300	-1.84578000
H	3.36218200	-1.91473200	-1.76051400
C	6.69395400	-1.35160900	-1.38541200
H	7.55222200	0.16222100	-0.11498100
H	5.54891200	-2.75391300	-2.55692400
H	7.65594500	-1.71971700	-1.73564100
C	-0.51280100	-0.60111900	0.98316600
C	-0.73781700	-1.66616700	0.11047500
C	-1.61956200	-0.01297400	1.60320300
C	-2.02754500	-2.10541900	-0.17398500
H	0.08983700	-2.16239900	-0.38822300
C	-2.91566800	-0.42774200	1.32650000
H	-1.46838900	0.80773700	2.30183100
C	-3.10392800	-1.46869100	0.42626100
H	-2.18047300	-2.92806900	-0.86639100
H	-3.75963600	0.05916600	1.80614200
C	0.23411000	2.35357700	0.27499100
C	2.90065600	-1.17598100	2.21289300
H	3.84683500	-1.71951100	2.01350600
O	2.57885200	-0.91395900	3.35100200
O	0.41754400	3.31854600	0.98805300
C	-2.11157700	2.96371900	-0.33233400
C	-3.00620000	2.38524100	-1.18759000
C	-2.30777300	1.32758700	-1.83849900
H	-4.03940300	2.67786900	-1.32779700
N	-0.93442200	2.25074000	-0.49003300
N	-1.06798200	1.25143400	-1.41361300
Br	-4.88370800	-2.03383800	0.00705500
C	1.74777300	0.46180600	-0.99133800
C	-2.30853100	4.08944300	0.61966300
H	-2.08634000	3.78408400	1.64724300
H	-1.65693600	4.93530100	0.38071900
H	-3.35102000	4.41501500	0.56304600
C	-2.83237600	0.37568500	-2.85737100
H	-3.17207600	0.90962400	-3.75232200
H	-2.05770000	-0.34073700	-3.14577000
H	-3.69187000	-0.17509100	-2.45729300
H	2.09292100	1.15225300	-1.76519300
H	1.15071500	-0.34050600	-1.42569600

3b_4

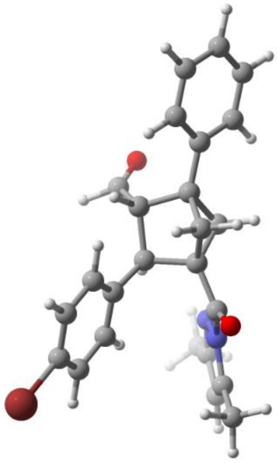

Charge = 0, **Multiplicity** = 1
Gibbs free energy = -3799.4497

No imaginary frequency found

C	0.47423900	0.01361400	0.69180500
C	0.94918800	0.68960000	-0.63240800
C	2.36616700	1.17623000	-0.23043600
C	2.65727500	-0.34821900	-0.20425900
C	1.76282800	-0.83077900	0.95890600
H	0.37523500	0.79584000	1.45015100
H	2.87301000	1.68149200	-1.05650900
H	2.46140800	1.74575000	0.69587100
H	1.55375000	-1.90516600	0.88339700
C	4.03023600	-0.92974600	-0.32592900
C	5.16749800	-0.12180900	-0.34518600
C	4.18677300	-2.31405500	-0.44970700
C	6.43544100	-0.68274900	-0.48089400
H	5.06375700	0.95648900	-0.24526900
C	5.45141800	-2.87723100	-0.58544900

H	3.30819300	-2.95759500	-0.44041400
C	6.58259000	-2.06228000	-0.60175900
H	7.31176500	-0.03778600	-0.49044200
H	5.55488000	-3.95622000	-0.68097200
H	7.57248300	-2.50102900	-0.70828900
C	-0.85865000	-0.68529700	0.58350400
C	-2.00852500	0.04825800	0.89612900
C	-1.01730500	-1.99442300	0.12551500
C	-3.27982700	-0.49117700	0.74364300
H	-1.91106000	1.06641500	1.26894300
C	-2.28190200	-2.55459300	-0.03156600
H	-0.15318700	-2.60688500	-0.11667000
C	-3.40106100	-1.79287000	0.27426900
H	-4.15620500	0.09957600	0.99401000
H	-2.38146400	-3.57574100	-0.38827600
C	-0.07840200	1.50002700	-1.36787000
C	2.26064600	-0.60254300	2.35650500
H	1.54624200	-0.91685100	3.14751700
O	3.32967300	-0.12030900	2.65630600
O	-0.49413400	1.18014800	-2.46101600
C	-1.74201900	3.33541900	-1.02095400
C	-1.86753100	4.27100500	-0.03260500
C	-0.76566500	4.07134900	0.84796100
H	-2.65600900	5.00797500	0.05602200
N	-0.59884000	2.62084100	-0.70966300
N	-0.01227000	3.07432000	0.44031200
Br	-5.14296200	-2.55354100	0.05544200
C	1.65209000	-0.45373900	-1.38558000
C	-2.62672100	3.06165600	-2.18470300
H	-2.10141600	3.21483300	-3.13221600
H	-2.98644500	2.02747000	-2.17160700
H	-3.48440800	3.73846400	-2.13840900
C	-0.43721800	4.83395600	2.08498400
H	-1.26725400	4.78024900	2.79920800
H	0.46257000	4.42936100	2.55773100
H	-0.27067300	5.89233300	1.85317400
H	2.09560500	-0.11235400	-2.32387500
H	1.11695900	-1.39513700	-1.53390200

3b_5

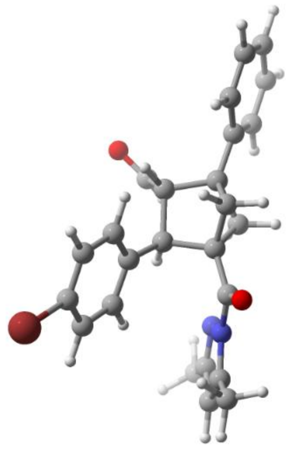

Charge = 0, **Multiplicity** = 1
Gibbs free energy = -3799.4523

No imaginary frequency found

C	0.51368400	0.02745600	0.62816300
C	0.95228100	0.75869900	-0.68157500
C	2.35538100	1.26866400	-0.26799700
C	2.68377900	-0.24949800	-0.30043400
C	1.79927900	-0.82413700	0.85051300
H	0.42483400	0.78143400	1.41604400
H	2.85827100	1.80420400	-1.07717500
H	2.43588700	1.81799000	0.67133400
H	1.64070600	-1.89562900	0.70141800
C	4.07971400	-0.77530400	-0.39489400
C	5.14945000	-0.06589900	0.15463700
C	4.32780000	-2.00552600	-1.00952300
C	6.44413800	-0.57566300	0.09139500
H	4.96901200	0.89498200	0.63369100
C	5.62050200	-2.51583200	-1.07490900
H	3.50137600	-2.56588700	-1.44377600
C	6.68367600	-1.80201600	-0.52378000
H	7.26836000	-0.01060700	0.52167300
H	5.79970100	-3.47282100	-1.56071100
H	7.69512100	-2.19929800	-0.57715400
C	-0.81441800	-0.68160600	0.52651500
C	-0.97226400	-1.96664800	0.00399900
C	-1.96219200	0.01910800	0.91329500
C	-2.23431600	-2.53589500	-0.13964300
H	-0.10898000	-2.55245000	-0.29851800
C	-3.23127700	-0.52948300	0.77434400
H	-1.86461800	1.01784400	1.33509400

C	-3.35179200	-1.80723500	0.24318000
H	-2.33328100	-3.53826400	-0.54624200
H	-4.10615900	0.03551700	1.08283000
C	-0.10573700	1.56300500	-1.37805800
C	2.39766200	-0.62334600	2.21112800
H	2.57470700	0.42960900	2.51479700
O	2.68973100	-1.52856100	2.96231000
O	-0.52385300	1.27063000	-2.47794500
C	-1.82964200	3.32035800	-0.93651800
C	-1.96954800	4.21769500	0.08489400
C	-0.84257300	4.03189500	0.93612400
H	-2.78330100	4.92040500	0.21366500
N	-0.65429700	2.64052100	-0.67208200
N	-0.06095900	3.07815100	0.48051100
Br	-5.09005600	-2.58169700	0.04439000
C	1.68106800	-0.33940300	-1.47853800
C	-2.72777000	3.04912500	-2.09052100
H	-2.22987200	3.25582300	-3.04263500
H	-3.04386400	2.00075700	-2.10565000
H	-3.61148000	3.68728100	-2.00332400
C	-0.51661400	4.76566900	2.19116300
H	-1.32889000	4.65771100	2.91949100
H	0.40711300	4.37970900	2.63231000
H	-0.39383100	5.83652200	1.99149600
H	2.11281900	0.04829600	-2.40437800
H	1.16648400	-1.28607700	-1.65960800

3b_6

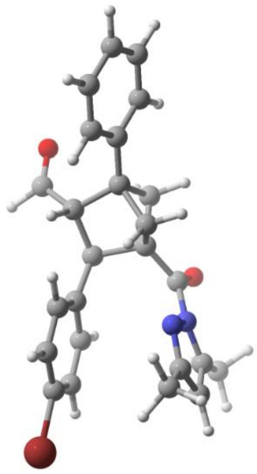

Charge = 0, **Multiplicity** = 1
Gibbs free energy = -3799.4506

No imaginary frequency found

C	-0.79697900	0.02403400	-1.26916400
C	-1.16739200	1.19367800	-0.29285200
C	-2.63535000	1.46618700	-0.68869200
C	-2.85883800	0.06810200	-0.06123400
C	-2.07865800	-0.84026200	-1.03571400
H	-0.79424200	0.42963200	-2.28793300
H	-3.07004200	2.26798000	-0.08603200
H	-2.86339100	1.60864400	-1.74846600
H	-1.82717300	-1.80209400	-0.57262600
C	-4.18066300	-0.41306500	0.44792500
C	-4.25962300	-1.63888500	1.11700400
C	-5.34241800	0.34479800	0.29814600
C	-5.47269600	-2.09822300	1.61929500
H	-3.36092700	-2.24057900	1.24581400
C	-6.55889900	-0.11247300	0.80010500
H	-5.29955700	1.29861000	-0.22308900
C	-6.62913500	-1.33533500	1.46274900
H	-5.51570300	-3.05435700	2.13693700
H	-7.45584500	0.48984000	0.67058800
H	-7.57879100	-1.69307400	1.85501000
C	0.55562200	-0.59978600	-1.02135900
C	1.66555600	-0.00672600	-1.63074800
C	0.77525300	-1.66704600	-0.15032000
C	2.96026100	-0.41968900	-1.34504600
H	1.51830600	0.81627100	-2.32756000
C	2.06356000	-2.10396600	0.14301900
H	-0.05477700	-2.16736500	0.34016000
C	3.14338200	-1.46319000	-0.44676000
H	3.80684000	0.07062500	-1.81647000
H	2.21272200	-2.92858500	0.83384200
C	-0.20565600	2.34845900	-0.30479600
C	-2.73883200	-1.18091100	-2.34054100
H	-2.09340900	-1.76043600	-3.03545700
O	-3.86680300	-0.88190700	-2.66193400
O	-0.38505300	3.31568700	-1.01576100
C	2.13180400	2.96345700	0.32793700
C	3.02070800	2.38231300	1.18719300
C	2.32032200	1.31755700	1.82449100
H	4.05146600	2.67780400	1.33893500
N	0.95576000	2.24504600	0.47017600
N	1.08477000	1.23970800	1.38787700
Br	4.92045200	-2.02720600	-0.01615300
C	-1.72649800	0.44620600	0.93728400

C	2.33311100	4.09663100	-0.61421600
H	2.12068100	3.79803400	-1.64583400
H	1.67673900	4.93864000	-0.37471800
H	3.37396300	4.42525200	-0.54624000
C	2.83918000	0.36068300	2.84159000
H	3.70549400	-0.18285700	2.44634200
H	3.16694300	0.88969200	3.74388700
H	2.06510100	-0.36159100	3.11662000
H	-2.07961100	1.13121100	1.71210500
H	-1.13220900	-0.35791700	1.37294800

3b_7

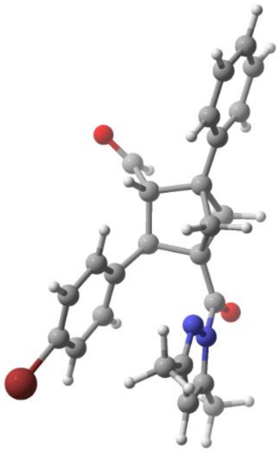

Charge = 0, **Multiplicity** = 1
Gibbs free energy = -3799.4524

No imaginary frequency found

C	-0.82746900	0.00182900	-1.22179500
C	-1.16145400	1.24361800	-0.32076900
C	-2.62075700	1.52124800	-0.73623800
C	-2.87179000	0.17315400	-0.01523500
C	-2.10175600	-0.84196300	-0.91431100
H	-0.84036200	0.33832800	-2.26526400
H	-3.04979700	2.36056300	-0.18248800
H	-2.84163800	1.61116300	-1.80287600
H	-1.89083000	-1.75533600	-0.35308400
C	-4.21890000	-0.25492000	0.47050600
C	-4.32561200	-1.18540400	1.50837700
C	-5.38471500	0.24043600	-0.11561600
C	-5.57340500	-1.60893800	1.95359400
H	-3.42233300	-1.57842000	1.97250200
C	-6.63564100	-0.18375600	0.32775100
H	-5.31429800	0.96648000	-0.92358500
C	-6.73388000	-1.10849900	1.36412600
H	-5.64164400	-2.32968800	2.76588500
H	-7.53595100	0.21286800	-0.13694100
H	-7.71024200	-1.43749300	1.71363700
C	0.52022900	-0.62671300	-0.95801900
C	1.62713400	-0.09425000	-1.62589300
C	0.74058000	-1.63504700	-0.01931600
C	2.92075600	-0.50930200	-1.33724000
H	1.47911700	0.68310400	-2.37313600
C	2.02725900	-2.07365900	0.27805400
H	-0.08720400	-2.08506200	0.52042400
C	3.10516000	-1.49301700	-0.37420400
H	3.76548600	-0.06542900	-1.85567200
H	2.17706400	-2.85104000	1.02148700
C	-0.17593300	2.37364500	-0.40920000
C	-2.85945300	-1.22502200	-2.15144300
H	-3.11621100	-0.39725000	-2.84476400
O	-3.19552500	-2.36000700	-2.41204900
O	-0.34449700	3.30344100	-1.17099000
C	2.18595000	2.96189500	0.14907800
C	3.07154200	2.41511600	1.03383200
C	2.35203400	1.41605200	1.75127200
H	4.11251800	2.68988100	1.15067600
N	0.99324700	2.28844300	0.35563000
N	1.10833100	1.34353500	1.33708800
Br	4.88131500	-2.05690800	0.06091800
C	-1.73581500	0.59071000	0.95669800
C	2.40328800	4.01950100	-0.87393600
H	2.16176100	3.65553700	-1.87785900
H	1.77702400	4.89625400	-0.68371500
H	3.45461900	4.31939300	-0.84761600
C	2.86014300	0.51560200	2.82374700
H	3.70421300	-0.07858900	2.45407400
H	3.21736200	1.09464500	3.68310400
H	2.07076900	-0.16369500	3.15832700
H	-2.07552200	1.33357100	1.68274200
H	-1.15576000	-0.19322400	1.44439000

3b_8

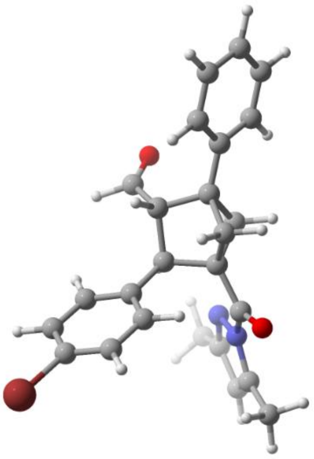

Charge = 0, **Multiplicity** = 1
Gibbs free energy = -3799.4476

No imaginary frequency found

C	0.32162600	0.00067200	0.55367700
C	0.98995600	0.75417600	-0.62165300
C	2.42235900	0.97159900	-0.05935100
C	2.50445500	-0.56404600	-0.23418400
C	1.46013900	-1.06458700	0.78380900
H	0.26906600	0.68049400	1.41054800
H	3.06065900	1.50749700	-0.76964800
H	2.51348700	1.39606300	0.94381300
H	1.09334100	-2.06712500	0.52290000
C	3.79372800	-1.31705000	-0.33549900
C	3.78059300	-2.65694400	-0.73698400
C	5.01868400	-0.71248800	-0.05081000
C	4.96653100	-3.37593900	-0.85008300
H	2.83150100	-3.14188000	-0.96505900
C	6.20724100	-1.43024700	-0.16493200
H	5.04535600	0.32731100	0.27041500
C	6.18557100	-2.76392000	-0.56429000
H	4.93931000	-4.41820700	-1.16440500
H	7.15481400	-0.94388100	0.06195200
H	7.11453000	-3.32490800	-0.65290300
C	-1.05688000	-0.58007800	0.35370300
C	-1.77026000	-0.98496700	1.48696400
C	-1.66014300	-0.73730500	-0.89545200
C	-3.03869600	-1.54244700	1.38674000
H	-1.32678000	-0.85974100	2.47411700
C	-2.93469600	-1.28689300	-1.01600500
H	-1.15081200	-0.42816600	-1.80469300
C	-3.60951300	-1.68853400	0.12751000
H	-3.57278200	-1.85002800	2.28264700
H	-3.38645100	-1.39539300	-1.99909800
C	0.23349300	1.85431600	-1.31090200
C	1.88099400	-1.11001800	2.22379000
H	1.11322300	-1.52480600	2.91339200
O	2.94612500	-0.72929200	2.65273000
O	-0.04238700	1.81698900	-2.49028500
C	-0.93249300	4.03253900	-0.89965200
C	-1.01127500	4.82149400	0.21287100
C	-0.25569800	4.16316200	1.22517700
H	-1.54899800	5.75912400	0.29771100
N	-0.15231600	2.94989400	-0.52719100
N	0.25595500	3.04078600	0.77436900
Br	-5.35592400	-2.44874900	-0.02940800
C	1.60474900	-0.37093900	-1.48201600
C	-1.54010700	4.22765100	-2.24241100
H	-0.77436500	4.30875500	-3.02017900
H	-2.19307000	3.39051700	-2.50976900
H	-2.13139800	5.14824600	-2.22663200
C	-0.02429800	4.61087200	2.62643800
H	0.47648200	5.58593500	2.64250500
H	-0.97666400	4.71963300	3.15838000
H	0.59761100	3.88665400	3.16137700
H	2.16567200	0.02274700	-2.33527800
H	0.97188600	-1.20945700	-1.79135100

3b_9

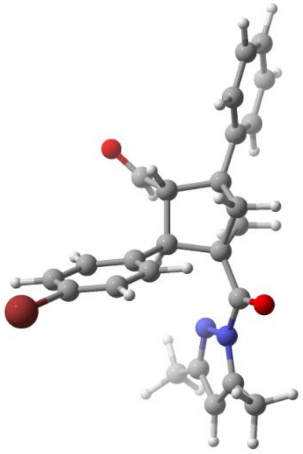

Charge = 0, **Multiplicity** = 1
Gibbs free energy = -3799.4498

No imaginary frequency found

C	0.39999200	0.03755200	0.49760300
C	1.01203500	0.88012500	-0.65039500
C	2.42448100	1.16147300	-0.06749300
C	2.60215100	-0.35973000	-0.30825300
C	1.56854800	-0.99317200	0.66948400
H	0.33807700	0.66899200	1.38847700
H	3.04126900	1.75176900	-0.74988800
H	2.47998700	1.55674800	0.94844600
H	1.28671400	-1.99443200	0.32947700
C	3.94211800	-1.01842400	-0.37893900
C	4.08769100	-2.24554300	-1.03262800
C	5.05683500	-0.43969000	0.22961900
C	5.32489700	-2.87927800	-1.07908000
H	3.22398700	-2.70616400	-1.50954100
C	6.29666200	-1.07392300	0.18583300
H	4.95563300	0.51603400	0.74069000
C	6.43460000	-2.29469300	-0.46939500
H	5.42515100	-3.83227500	-1.59450000
H	7.15729500	-0.61017500	0.66336400
H	7.40288800	-2.78935300	-0.50721000
C	-0.95551400	-0.59178400	0.29784800
C	-1.63770900	-1.05268100	1.42792200
C	-1.56281700	-0.74346100	-0.94893400
C	-2.88555700	-1.65412500	1.32722900
H	-1.18539000	-0.93564900	2.41129800
C	-2.81655500	-1.33800900	-1.07076800
H	-1.07129900	-0.39753600	-1.85352600
C	-3.46338500	-1.78956600	0.07011600
H	-3.39674100	-2.00366000	2.21978900
H	-3.27361300	-1.44168600	-2.05068400
C	0.17947100	1.95117800	-1.29675100
C	2.06056500	-1.08463700	2.08173900
H	2.34622800	-0.12351500	2.55870000
O	2.15847100	-2.12243500	2.70037100
O	-0.05583500	1.96287200	-2.48668300
C	-1.29467700	3.90288800	-0.76932500
C	-1.47778500	4.61780500	0.38060100
C	-0.60173000	4.04835800	1.34879100
H	-2.16111200	5.44672800	0.51751900
N	-0.34179300	2.94681300	-0.46223100
N	0.07405600	3.04357800	0.83847800
Br	-5.18517500	-2.61006200	-0.08813700
C	1.70142800	-0.16813500	-1.55417700
C	-1.96283600	4.04728000	-2.09021300
H	-1.24654700	4.31365600	-2.87331800
H	-2.44782300	3.11344400	-2.39265100
H	-2.71862500	4.83402000	-2.01464600
C	-0.41300900	4.46681300	2.76606700
H	-0.06468100	5.50503500	2.81863000
H	-1.36191400	4.41040700	3.31225200
H	0.31920600	3.82271800	3.26208900
H	2.24504600	0.29715500	-2.37943900
H	1.12106600	-1.02603400	-1.90277600

References

1 Y.-J. Li, Z.-L. Wu, Q.-S. Gu, T. Fan, M.-H. Duan, L. Wu, Y.-T. Wang, J.-P. Wu, F.-L. Fu, F. Sang, A.-T. Peng, Y. Jiang, X.-Y. Liu, J.-S. Lin, *J. Am. Chem. Soc.* 2024, **146**, 34427-34441.

2 Gaussian 16, Revision B.01, M. J. Frisch, G. W. Trucks, H. B. Schlegel, G. E. Scuseria, M. A. Robb, J. R. Cheeseman, G. Scalmani, V. Barone, G. A. Petersson, H. Nakatsuji, X. Li, M. Caricato, A. V. Marenich, J. Bloino, B. G. Janesko, R Gomperts, B. Mennucci, H. P. Hratchian, J. V. Ortiz, A. F. Izmaylov, J. L. Sonnenberg, D. Williams-Young, F. Ding, F. Lipparini, F. Egidi, J. Goings, B. Peng, A. Petrone, T. Henderson, D. Ranasinghe, V. G. Zakrzewski, J. Gao, N. Rega, G. Zheng, W. Liang, M. Hada, M. Ehara, K. Toyota, R. Fukuda, J. Hasegawa, M. Ishida, T. Nakajima, Y. Honda, O. Kitao, H. Nakai, T. Vreven, K. Throssell, J. A. Montgomery, J. E. Peralta, F. Ogliaro, M. J. Bearpark, J. J. Heyd, E. N. Brothers, K. N. Kudin, V. N. Staroverov, T. A. Keith, R. Kobayashi, J. Normand, K. Raghavachari, A. P. Rendell, J. C. Burant, S. S. Iyengar, J. Tomasi, M. Cossi, J. M. Millam, M. Klene, C. Adamo, R. Cammi, J. W. Ochterski, R. L. Martin, K. Morokuma, O. Farkas, J. B. Foresman, D. J. Fox, Gaussian, Inc., Wallingford CT, 2016.

3 Schrödinger Release 2019-1: MacroModel, Schrödinger, LLC, New York, NY, 2019.

4 Schrödinger Release 2019-1: Maestro, Schrödinger, LLC, New York, NY, 2019.

5 J. Chai, M. Head-Gordon *Phys. Chem. Chem. Phys.,* 2008, **10**, 6615-6620.

6 F. Jensen *J. Chem. Theory Comput.* 2014, **10**, 1074-1085.

7 A. V. Manerich, C. J. Cramer, D. G. Truhlar *J. Phys. Chem. B*, 2009, **113**, 6378-6396.

8 G. Luchini, J. V. Alegre-Requena, I. Funes-Ardoiz, R. S. Paton *F1000Research*, 2020, **9**, 291.

9 D. M. McCann, P. J. Stephens *J. Org. Chem.* 2006, **71**, 6074-6098.

10 GaussView, Version 6, R. Dennington, T. A. Keith, J. M. Millam, Semichem Inc., Shawnee Mission, KS, 2016.

11 CYLview, 1.0b; Legault, C. Y. Université de Sherbrooke, 2009.
